# Agent- and Dose-Specific Intestinal Obstruction Safety of GLP-1 Receptor Agonists and SGLT2 Inhibitors: A Network Meta-Analysis of Randomized Trials

**DOI:** 10.3390/ijms27020608

**Published:** 2026-01-07

**Authors:** Jiann-Jy Chen, Chih-Wei Hsu, Chao-Ming Hung, Mein-Woei Suen, Hung-Yu Wang, Wei-Chieh Yang, Brendon Stubbs, Yen-Wen Chen, Tien-Yu Chen, Wei-Te Lei, Andre F. Carvalho, Shih-Pin Hsu, Yow-Ling Shiue, Bing-Yan Zeng, Cheng-Ta Li, Kuan-Pin Su, Chih-Sung Liang, Bing-Syuan Zeng, Ping-Tao Tseng

**Affiliations:** 1 Prospect Clinic for Otorhinolaryngology & Neurology, Kaohsiung 81166, Taiwan; jiannjy@yahoo.com.tw (J.-J.C.); kevinachen0527@gmail.com (Y.-W.C.); 2 Department of Otorhinolaryngology, E-Da Cancer Hospital, I-Shou University, Kaohsiung 82445, Taiwan; 3 Department of Psychiatry, Kaohsiung Chang Gung Memorial Hospital and Chang Gung University College of Medicine, Kaohsiung 833401, Taiwan; harwicacademia@gmail.com; 4 Division of General Surgery, Department of Surgery, E-Da Cancer Hospital, I-Shou University, Kaohsiung 82445, Taiwan; ed100647@edah.org.tw; 5 School of Medicine, College of Medicine, I-Shou University, Kaohsiung 82445, Taiwan; a.pin.hsu@gmail.com; 6 Department of Psychology, College of Medical and Health Science, Asia University, Taichung 41354, Taiwan; blake@asia.edu.tw; 7 Gender Equality Education and Research Center, Asia University, Taichung 41354, Taiwan; 8 Department of Medical Research, Asia University Hospital, Asia University, Taichung 41354, Taiwan; 9 Department of Medical Research, China Medical University Hospital, China Medical University, Taichung 404332, Taiwan; 10 Kaohsiung Municipal Kai-Syuan Psychiatric Hospital, Kaohsiung 80276, Taiwan; hywang1975@gmail.com; 11 Department of Pediatrics, Ping An Medical Clinic, Tainan 708, Taiwan; medarchies@gmail.com; 12 Department of Psychological Medicine, Institute of Psychiatry, Psychology and Neuroscience, King’s College London, London SE5 8AF, UK; brendon.stubbs@kcl.ac.uk; 13 Department of Sport, University of Vienna, 1010 Vienna, Austria; 14 Department of Psychiatry, Tri-Service General Hospital, Taipei 114202, Taiwan; verducciwol@gmail.com; 15 Department of Psychiatry, College of Medicine, National Defense Medical University, Taipei 114202, Taiwan; 16 Division of Pediatric Allergy, Immunology, and Rheumatology, Department of Pediatrics, Hsinchu Municipal MacKay Children’s Hospital, Hsinchu 30068, Taiwan; lazyleisure@gmail.com; 17 Graduate Institute of Clinical Medical Sciences, College of Medicine, Chang Gung University, Taoyuan 333323, Taiwan; 18 Innovation in Mental and Physical Health and Clinical Treatment (IMPACT) Strategic Research Centre, School of Medicine, Barwon Health, Deakin University, Geelong, VIC 3220, Australia; canaldasaudemental@gmail.com; 19 Department of Neurology, E-Da hospital, I-Shou University, Kaohsiung 82445, Taiwan; 20 Institute of Precision Medicine, National Sun Yat-Sen University, Kaohsiung 80424, Taiwan; shirley@imst.nsysu.edu.tw; 21 Institute of Biomedical Sciences, National Sun Yat-Sen University, Kaohsiung 80424, Taiwan; holdinggreat@yahoo.com.tw; 22 Department of Internal Medicine, E-Da Dachang Hospital, I-Shou University, Kaohsiung 82445, Taiwan; 23 Department of Psychiatry, Taipei Veterans General Hospital, Taipei 112201, Taiwan; on5083@msn.com; 24 Division of Psychiatry, School of Medicine, National Yang Ming Chiao Tung University, Taipei 112, Taiwan; 25 Institute of Brain Science and Brain Research Center, School of Medicine, National Yang Ming Chiao Tung University, Taipei 112, Taiwan; 26 Mind-Body Interface Research Center (MBI Lab & Care), China Medical University Hospital, Taichung 40447, Taiwan; cobolsu@gmail.com; 27 Office of Research and Development, Asia University, Taichung 413305, Taiwan; 28 College of Medicine, China Medical University, Taichung 404328, Taiwan; 29 An-Nan Hospital, China Medical University, Tainan 709, Taiwan; 30 Department of Psychiatry, Beitou Branch, Tri-Service General Hospital, School of Medicine, National Defense Medical University, Taipei 114202, Taiwan; lcsyfw@gmail.com; 31 Department of Psychiatry, National Defense Medical University, Taipei 114202, Taiwan; 32 Department of Internal Medicine, E-Da Cancer Hospital, I-Shou University, Kaohsiung 82445, Taiwan

**Keywords:** network meta-analysis, GLP-1 receptor agonist, SGLT2 inhibitor, intestine obstruction, ileus, adverse effect

## Abstract

Glucagon-like peptide-1 (GLP-1) receptor agonists and sodium–glucose cotransporter-2 (SGLT2) inhibitors have reshaped pharmacological management of type 2 diabetes, but emerging safety signals suggest a possible association with intestinal obstruction. Because many candidates for these agents already harbor risk factors for ileus and bowel obstruction, clarifying agent- and dose-specific gastrointestinal safety is clinically important. We aimed to re-evaluate the risk of intestinal obstruction across individual GLP-1 receptor agonists and SGLT2 inhibitors, with particular attention to dose stratification. We systematically searched eight databases through 21 January 2025 to identify randomized controlled trials (RCTs) comparing GLP-1 receptor agonists or SGLT2 inhibitors with placebo or active comparators in adults. The primary outcome was incident intestinal obstruction (small or large bowel). A frequentist random-effects network meta-analysis estimated odds ratios (ORs) with 95% confidence intervals (CIs) across drugs and dose tiers; Bayesian models and surface under the cumulative ranking (SUCRA) metrics were used for sensitivity analyses and treatment ranking. Risk of bias and certainty of evidence were assessed with standard Cochrane and GRADE-adapted tools. Fifty RCTs (47 publications; 192,359 participants) met inclusion criteria. Overall, canagliflozin use was associated with a higher incidence of intestinal obstruction than control therapies (OR 2.56, 95% CI 1.01–6.49), corresponding to an absolute risk difference of 0.15% and a number needed to harm of 658. High-dose canagliflozin (300 mg/day) showed the clearest signal (OR 3.42, 95% CI 1.08–10.76). In contrast, liraglutide was associated with a lower risk of intestinal obstruction (OR 0.44, 95% CI 0.24–0.81), with an absolute risk reduction of 0.34% and a number needed to treat of 295. No other GLP-1 receptor agonist or SGLT2 inhibitor demonstrated a statistically significant increase in obstruction risk. Frequentist and Bayesian analyses yielded concordant estimates and rankings. From a randomized-trial perspective, intestinal obstruction risk is not elevated for most GLP-1 receptor agonists and SGLT2 inhibitors. A dose-dependent safety signal was observed only for high-dose canagliflozin, whereas liraglutide may confer a protective effect. These findings refine gastrointestinal safety profiles for modern antidiabetic agents and may inform perioperative bowel management, drug selection, and dose optimization in patients at risk for ileus or adhesive obstruction.

## 1. Introduction

Glucagon-like peptide-1 (GLP-1) receptor agonists and sodium–glucose cotransporter-2 (SGLT2) inhibitors are widely used “novel” antidiabetic agents whose pharmacological actions differ substantially from those of traditional insulin secretagogues or sensitizers [1]. GLP-1 receptor agonists are peptide-based incretin mimetics that act through a G protein-coupled receptor to enhance glucose-dependent insulin secretion, slow gastric emptying, and modulate appetite, whereas SGLT2 inhibitors are small molecules that block renal tubular glucose reabsorption and increase urinary glucose excretion. As clinical indications have broadened from glycemic control to cardiovascular and renal risk reduction, cumulative exposure in real-world practice has increased, and with it, recognition of previously underappreciated adverse drug reactions [2].

Intestinal obstruction involving either the small or large bowel represents a serious gastrointestinal complication with potential for rapid clinical deterioration. Populations commonly treated with GLP-1 receptor agonists or SGLT2 inhibitors—such as individuals with long-standing diabetes, obesity, prior abdominal surgery, or malignancy—frequently have baseline risk factors for ileus or mechanical obstruction [3,4]. In these patients, drug-related changes in motility, secretion, or intraluminal volume can have outsized consequences. Failure to promptly recognize and manage obstruction can lead to perforation, free intraperitoneal air or fluid, peritonitis, and septic shock, often necessitating emergency surgical intervention [5].

In recent years, several case reports and pharmacovigilance analyses have described intestinal obstruction in temporal association with GLP-1 receptor agonist therapy [6,7,8,9]. These observations have raised concern that sustained GLP-1 receptor activation, through its effects on gastrointestinal motility and secretion, may contribute to bowel stasis in susceptible individuals. A narrative review by Jalleh et al. proposed that GLP-1 receptor agonists may exacerbate delayed gastric emptying and prolong small-bowel transit, thereby increasing the likelihood of luminal distension and obstruction [10]. Reflecting these accumulating data, the U.S. Food and Drug Administration has updated product information for certain GLP-1 receptor agonists to include intestinal obstruction or ileus as potential adverse reactions.

Compared with GLP-1 receptor agonists, the relationship between SGLT2 inhibitors and obstruction risk has been less thoroughly characterized. Some mechanistic hypotheses suggest that enhanced osmotic diuresis and mild volume depletion might indirectly influence perfusion and motility of the gut, whereas other preclinical data point in the opposite direction. For example, Nozu et al. reported that SGLT2 inhibition could attenuate postoperative ileus in a rat model, implying a possible protective modulation of intestinal function [11]. These contrasting pharmacological observations underscore the need for a comprehensive comparative safety assessment.

At the level of randomized controlled trials (RCTs), however, safety signals from case series and regulatory databases have not been consistently reproduced [12,13,14]. Individual trials are often underpowered for rare events such as intestinal obstruction, and safety endpoints may be captured as part of broad adverse-event summaries rather than systematically adjudicated outcomes. Under these circumstances, meta-analytic techniques are essential to aggregate sparse data across trials and to provide more precise estimates of low-frequency harms. Pooled analyses based on diverse trial populations are frequently used as reference points for clinical guidelines and for hypothesis generation in pharmacovigilance research [15,16].

To date, no conventional meta-analysis or network meta-analysis (NMA) has systematically quantified the risk of intestinal obstruction across individual GLP-1 receptor agonists and SGLT2 inhibitors while accounting for dose. NMAs are particularly well suited to this question because they can integrate both direct and indirect comparisons among multiple agents and dosing regimens, treating each drug–dose combination as a distinct node and enabling inference on relative safety rankings [17]. The present study extends our group’s prior NMA work examining neurologic [18], metastatic [19], colorectal [20], and auditory [21] outcomes associated with these drug classes by focusing on intestinal obstruction as a predefined safety endpoint. Our objective was to delineate agent- and dose-specific obstruction risk for GLP-1 receptor agonists and SGLT2 inhibitors using a rigorously conducted, dose-stratified NMA of RCTs.

## 2. Results

### 2.1. Eligibility of the Studies

**Figure 1:**
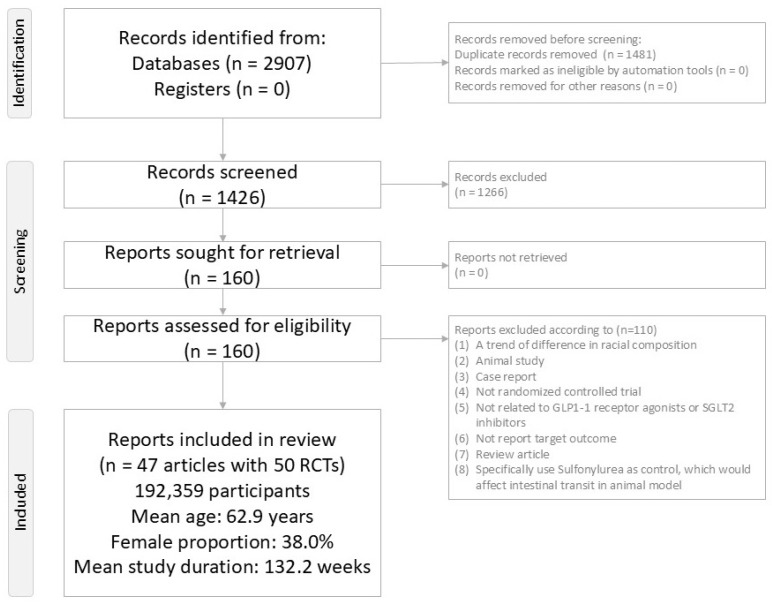
PRISMA2020 Flowchart of current network meta-analysis.

The study selection process is summarized in Figure 1. After screening and full-text review, 110 articles were excluded for reasons such as ineligible design, absence of relevant outcomes, or duplicate reporting (Table S3) [6,7,8,9,10,11,22,23,24,25,26,27,28,29,30,31,32,33,34,35,36,37,38,39,40,41,42,43,44,45,46,47,48,49,50,51,52,53,54,55,56,57,58,59,60,61,62,63,64,65,66,67,68,69,70,71,72,73,74,75,76,77,78,79,80,81,82,83,84,85,86,87,88,89,90,91,92,93,94,95,96,97,98,99,100,101,102,103,104,105,106,107,108,109,110,111,112,113,114,115,116,117,118,119,120,121,122,123,124,125]. Ultimately, 47 publications describing 50 distinct RCTs were included in the NMA (Table S4) [12,13,14,126,127,128,129,130,131,132,133,134,135,136,137,138,139,140,141,142,143,144,145,146,147,148,149,150,151,152,153,154,155,156,157,158,159,160,161,162,163,164,165,166,167,168,169]. Across these trials, 192,359 participants were randomized. The mean age was 62.9 years (range 44.9–71.9 years), and women comprised a mean of 38.0% of participants (range 23.4–81.0%). Mean trial duration was 132.2 weeks, with follow-up ranging from 13 to 281 weeks.

GLP-1 receptor agonists evaluated included tirzepatide, efpeglenatide, liraglutide, albiglutide, dulaglutide, exenatide, semaglutide, and lixisenatide. SGLT2 inhibitors assessed were bexagliflozin, canagliflozin, empagliflozin, ertugliflozin, dapagliflozin, and sotagliflozin. Trials varied in background therapy and baseline cardiovascular risk, but collectively represented contemporary clinical use of these agents in type 2 diabetes and cardiometabolic populations.

### 2.2. Primary Outcome: Intestine Obstruction Events

**Figure 2:**
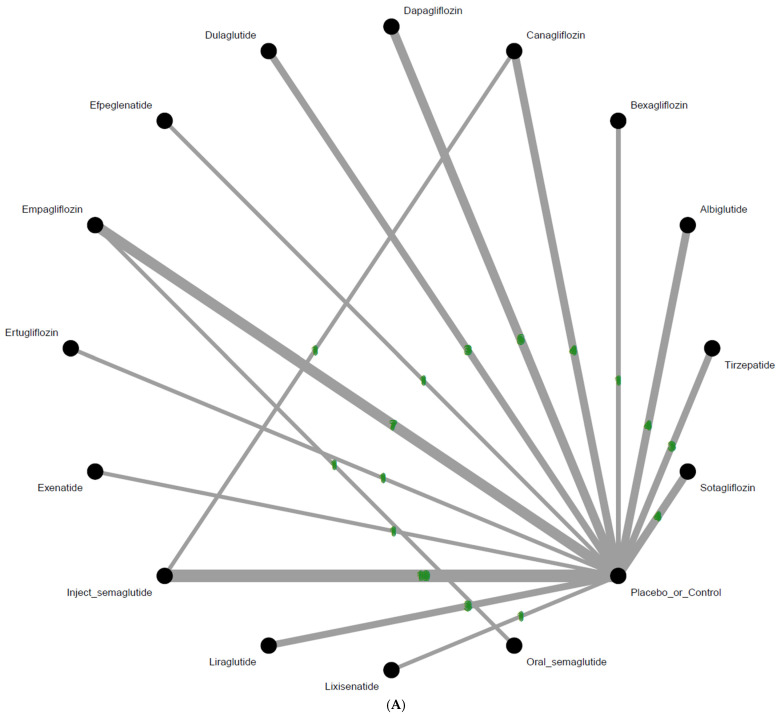

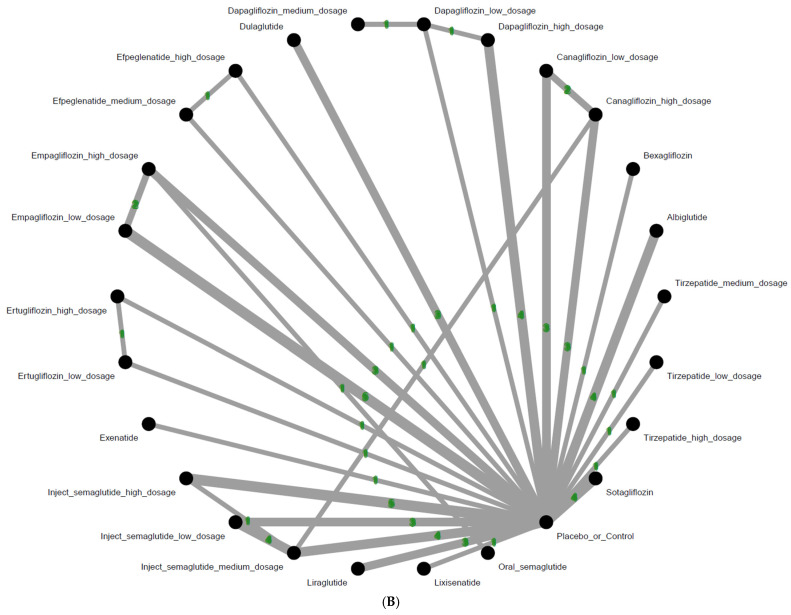
(**A**) Network structure of the primary outcome: intestine obstruction events. The overall structure of the network meta-analysis. The lines between nodes represent direct comparisons from various trials, with the green numbers over the lines indicating the number of trials providing these comparisons for each specific treatment. The thickness of the lines corresponds to the number of trials linked to the network. (**B**) Network structure of the primary outcome: intestine obstruction events in aspect of various dosage subgroup. The overall structure of the network meta-analysis. The lines between nodes represent direct comparisons from various trials, with the green numbers over the lines indicating the number of trials providing these comparisons for each specific treatment. The thickness of the lines corresponds to the number of trials linked to the network.

**Figure 3:**
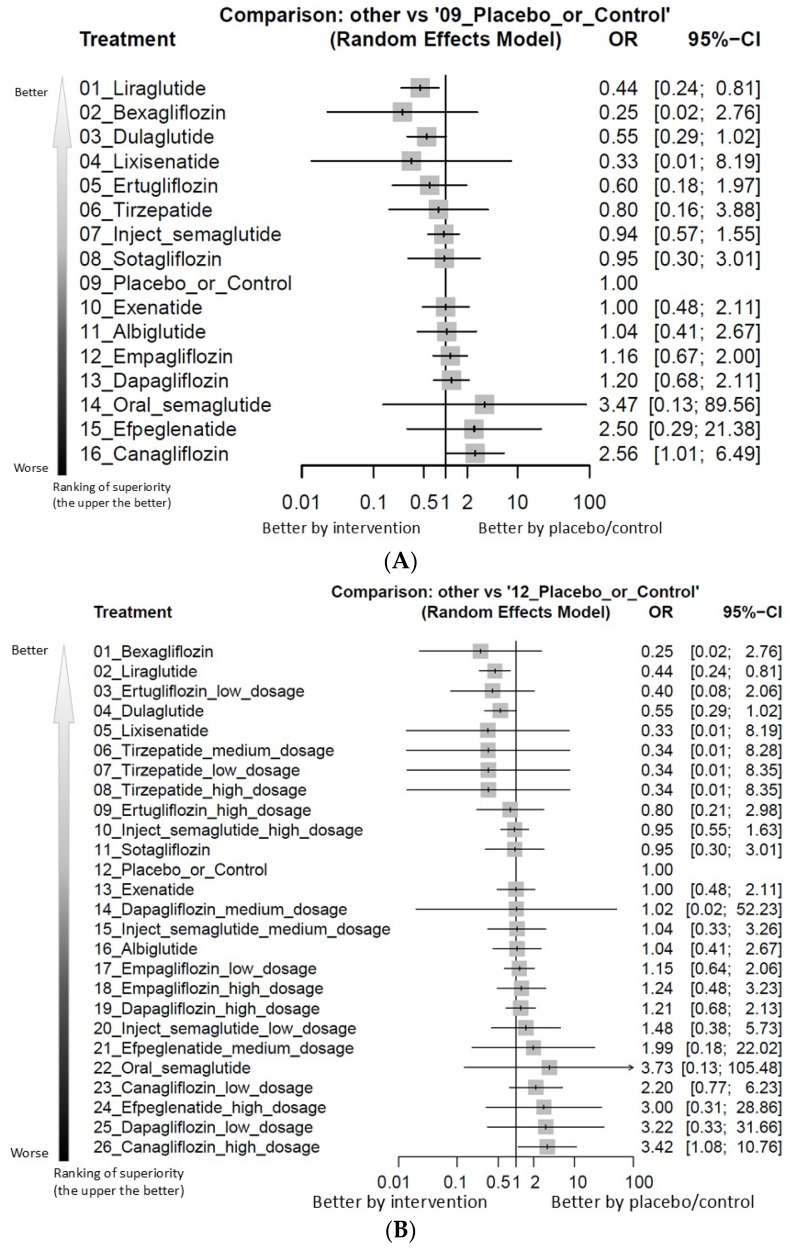
(**A**) Forest plot of primary outcome: intestine obstruction events. When the effect size (expressed as odds ratio) is less than 1, the specified treatment is associated with fewer events of intestine obstruction compared to placebo/controls. (**B**) Forest plot of primary outcome: intestine obstruction events in aspect of various dosage subgroup. When the effect size (expressed as odds ratio) is less than 1, the specified treatment is associated with fewer events of intestine obstruction compared to placebo/controls.

In the primary NMA, canagliflozin use was associated with a statistically significant increase in intestinal obstruction compared with pooled control treatments (OR 2.56, 95% CI 1.01–6.49). This corresponds to an absolute risk difference of 0.15% and a number needed to harm of approximately 658. Among all regimens, canagliflozin occupied the highest rank in terms of obstruction risk (Figure 2A and Figure 3A, Figure S3A, and Table 1). As addressed in the introduction, patients with diabetes mellitus often have multiple risk factors for intestine obstruction (for example, autonomic nervous system dysfunction and obesity). Although we intended to perform subgroup analysis focusing on RCTs with specific comorbidity (such as autonomic nervous system dysfunction and obesity), the analytic process could not be performed due to the sparse numbers of RCTs focusing such specific comorbidity.

Liraglutide, in contrast, was the only regimen associated with a statistically significant reduction in intestinal obstruction. Compared with controls, liraglutide yielded an OR of 0.44 (95% CI 0.24–0.81), translating into an absolute risk difference of −0.34% and a number needed to treat of 294.7. For all other GLP-1 receptor agonists and SGLT2 inhibitors, point estimates for obstruction were generally close to unity and did not reach conventional thresholds for statistical significance.

### 2.3. Dose-Stratified Analyses of Intestinal Obstruction

Subgroup analyses stratified by dosing tier highlighted a pronounced dose–response signal for canagliflozin. High-dose canagliflozin (300 mg/day) was associated with a significantly increased risk of intestinal obstruction relative to control (OR 3.42, 95% CI 1.08–10.76), with an absolute risk difference of 0.11% and a number needed to harm of 933.39. Across all evaluated dose nodes, high-dose canagliflozin consistently ranked as the regimen with the greatest obstruction risk (Figure 2B and Figure 3B, Figure S3B, and Table 2). In contrast, lower canagliflozin doses and dose tiers for other agents did not demonstrate comparable elevations in risk.

### 2.4. Acceptability: Drop-Out Rate

We used all-cause study withdrawal as a global indicator of treatment acceptability. Several agents exhibited significantly lower discontinuation rates than their respective control arms. These regimens included tirzepatide (OR 0.44, 95% CI 0.35–0.55), oral semaglutide (OR 0.40, 95% CI 0.19–0.85), a secondary tirzepatide comparison (OR 0.56, 95% CI 0.42–0.74), canagliflozin (OR 0.66, 95% CI 0.55–0.80), albiglutide (OR 0.71, 95% CI 0.58–0.86), liraglutide (OR 0.76, 95% CI 0.66–0.89), dapagliflozin (OR 0.79, 95% CI 0.62–0.99), injectable semaglutide (OR 0.81, 95% CI 0.70–0.94), sotagliflozin (OR 0.86, 95% CI 0.74–0.99), and empagliflozin (OR 0.87, 95% CI 0.79–0.95). Overall, tirzepatide ranked most favorably in terms of retention and global acceptability (Figures S1, S2 and S3C, and Table S5).

### 2.5. Sensitivity Analyses Using Bayesian NMA

Bayesian NMA models produced effect estimates and treatment rankings that were highly consistent with those obtained from frequentist analyses (Figure S4A–C). SUCRA-based rankings derived from Bayesian models are presented in Table S6A–C and Figure S5A–F. Deviation-model diagnostics did not reveal major incoherence or instability across treatment comparisons, suggesting that the network structure was well supported by the underlying trial data (Figure S6A–I).

### 2.6. Risk of Bias, Consistency, and Certainty of Evidence

Across all included trials, 81.4% (285 of 350 items) of risk-of-bias judgments were categorized as low risk, 15.4% (54 items) as unclear, and 3.2% (11 items) as high risk (Figure S7A,B). Network-wide consistency checks did not identify statistically significant discrepancies between direct and indirect estimates for key comparisons (Table S7A–C). According to GRADE assessments adapted for NMA, the certainty of evidence for most agent-level comparisons ranged from moderate to high, supporting the reliability of the main conclusions (Table S8A–C).

## 3. Discussion

To our knowledge, this is the first network meta-analysis to systematically evaluate intestinal obstruction risk across individual GLP-1 receptor agonists and SGLT2 inhibitors, incorporating dose-stratified nodes within each agent. By leveraging RCT data, we provide a randomized-trial-based perspective on a safety signal that was thus far superior to the case reports and spontaneous pharmacovigilance observations. The principal findings are that high-dose canagliflozin (300 mg/day) is associated with an increased risk of intestinal obstruction, whereas liraglutide appears to confer a reduced risk, and no other GLP-1 receptor agonist or SGLT2 inhibitor was linked to a statistically significant increase in obstruction events.

The most clinically consequential observation is the dose-dependent safety signal identified for canagliflozin. At 300 mg/day, canagliflozin showed the highest ranking for obstruction risk in both overall and dose-stratified analyses. This pattern suggests that intestinal obstruction may be related not merely to class-level SGLT2 inhibition, but to specific pharmacological properties of canagliflozin at higher exposures. Preclinical work has indicated that canagliflozin can promote intestinal adenomatosis through complex non-cell-autonomous signaling mechanisms [170], although analogous tumorigenic effects have not been demonstrated in human trials. Canagliflozin had been found to exert effects of postprandial glucose reduction by delaying intestinal glucose absorption [171]. Pharmacodynamic studies indicate that canagliflozin slows glucose absorption along the intestinal tract [172], potentially increasing intraluminal osmolar load and prolonging overall transit time [173]. At high doses, partial inhibition of intestinal SGLT1 in addition to SGLT2 blockade may further alter luminal glucose handling and fluid dynamics, creating conditions conducive to distension and stasis. The previous animal study had demonstrated the conversely increased GLP-1 and peptide YY (PYY) levels related to SGLT1 inhibition by LX4211 prescription, a dual SGLT1 and SGLT2 inhibitor [174]. The elevated plasma GLP-1 [175] and PYY levels [176] would lead to intestine motility inhibition. Therefore canagliflozin, which regimen had higher SGLT1 inhibitory effects than the other SGLT2 inhibitors [177], would have a possibility of intestine obstruction beyond a class-level effect. Nevertheless, although we detected statistical significance in our NMA, the absolute risk difference (0.11%) and number needed to harm (933.39) were both clinically less relevant. This statistical/clinical discrepancy should be particularly important when we took risk–benefit considerations in patients with high cardiovascular risk. Further, since the causality cannot be firmly established from trial-level data alone, the mechanistic human studies and prospective pharmacovigilance focused on high-dose canagliflozin should be warranted to clarify whether the observed association reflects a true drug effect, a high-risk clinical phenotype, or residual confounding.

In contrast, our pooled RCT results do not corroborate a generalized increase in obstruction risk for GLP-1 receptor agonists. None of the GLP-1 receptor agonists in the network were associated with excess obstruction events, and liraglutide was linked to a statistically significant reduction in risk. Wegeberg et al. reported that liraglutide reduced colonic transit time and improved intestinal motility indices in a controlled human study [178], which may help explain the protective association observed in our analysis. Although GLP-1 receptor activation is often viewed as inhibitory to gastric motility, particularly in the proximal stomach, prior research suggests that GLP-1 receptor agonists function more as modulators than simple suppressors of gut motility. In patients with diabetic neuropathy and substantially delayed gastric emptying, GLP-1 receptor agonists did not further impair motility and, in some cases, may normalize abnormal patterns [179].

From a mechanistic standpoint, GLP-1 receptor agonists bind to GLP-1 receptors, which are class B G protein-coupled receptors, leading to activation of adenylate cyclase, increased intracellular cyclic AMP, and downstream signaling via protein kinase A and EPAC [180]. GLP-1 receptors are expressed on myenteric plexus neurons and other components of the enteric nervous system [181], where GPCR activation can modulate both mucosal secretion and smooth-muscle contractility [182]. These pharmacodynamic effects appear to be region- and context-dependent, with the potential to either slow or normalize transit depending on baseline motility status. Our findings support the concept that, when used at standard doses in contemporary RCT populations, GLP-1 receptor agonists as a class do not materially increase intestinal obstruction risk and that liraglutide in particular may exert net favorable effects on motility-related outcomes.

### Strengths and Limitations

This study has several strengths that enhance confidence in the conclusions. First, the network meta-analytic framework allowed us to integrate data from multiple active and placebo comparators and to compare individual agents and dose tiers within a single coherent model. This design is particularly valuable in the context of modern antidiabetic therapy, where head-to-head trials of all clinically relevant regimens are not feasible. Second, we restricted analyses to RCTs and excluded participants with pre-existing obstruction, thereby focusing on incident events and improving internal validity. Third, dose-stratified modeling enabled us to separate class effects from dose-specific pharmacodynamic signals, an important consideration for agents such as canagliflozin where high-dose regimens may have distinct intestinal exposures compared with lower doses. Fourth, we corroborated frequentist results using Bayesian NMA and SUCRA-based rankings, which yielded concordant patterns and support the stability of our findings.

Several limitations should also be acknowledged. First, we could not consistently distinguish small bowel from large bowel obstruction, or mechanical obstruction from functional ileus, because such distinctions were rarely reported in the source trials. This lack of granularity limited our ability to examine specific pathophysiological subtypes. Second, by focusing on RCTs, we may have excluded valuable information from large observational cohorts that capture longer exposure durations and broader patient populations. Nonetheless, RCT-based estimates provide an essential anchor for causality and reduce confounding by indication. Third, heterogeneity in diagnostic criteria, imaging practices, and adverse-event coding across international trials may have introduced variability in outcome ascertainment and contributed to imprecision in some estimates. As addressed in the methodology section, the current NMA did not make distinction between mechanical obstruction and functional ileus, nor between small-bowel and large-bowel obstruction. In fact, the most frequently recorded “intestine obstruction events” among the included RCTs were just remarked as “intestine obstruction” without any other specifiers. Therefore, it would be practically difficult to distinguish them. We surely recognized that the process of merging these site-specific and mechanism-specific intestine obstruction into one group would impose potential bias in the main result. However, meta-analyses of large datasets remain a cornerstone for safety signal detection and are frequently used to generate hypotheses for regulatory and pharmacoepidemiologic follow-up [15,16,183,184]. Fourth, concomitant medications that influence gastrointestinal motility (e.g., opioids, anticholinergics) were not systematically accounted for in the trial reports. This limitation was the natural result that the most RCTs were not initially designed to evaluate intestine obstruction, which needed specific information regarding baseline medication affecting gastrointestinal motility. Although randomization should mitigate systematic imbalances, residual confounding cannot be completely excluded. Therefore, future large-scale RCTs specifically designed for exploring gastrointestinal motility related to such medications should be warranted. Fifth, since some of the intestine obstruction events of the included RCTs came from spontaneous reporting systems and none of the included RCTs were primarily designed to evaluate intestinal obstruction or ileus, the possibility of underreporting and inconsistent capture of rare events might raise the possibility of underestimation of intestine obstruction. In fact, the lack of large-scale RCTs directly investigating intestine obstruction related to such medication prescription might limit the certainty of the evidence. Specifically, evidence from RCTs with direct investigation of intestine obstruction adverse event data might provide more reliable evidence than post-marketing reports. Therefore, dedicated prospective studies, ideally with predefined gastrointestinal safety endpoints and standardized adjudication, will be necessary to fully characterize the mechanistic links between these agents and obstructive complications. In addition, there was still a gap for the linkage between animal study and statistical evidence in our NMA so that clinicians should pay special attention when applied our result in clinical practice. Finally, as addressed in the introduction, patients with diabetes mellitus often have multiple risk factors for intestine obstruction (for example, autonomic nervous system dysfunction and obesity). Despite our intention to perform subgroup analysis to exclude the potential confounding effects, the analytic process could not be performed due to the sparse data. Therefore, future large-scale RCTs focusing specific comorbidity should be warranted.

## 4. Materials and Methods

This NMA was designed as a confirmatory safety analysis targeting intestinal obstruction as a prespecified adverse outcome of interest. The study was conducted following the Cochrane Handbook guidance for safety-focused systematic reviews [185] and adhered to the Preferred Reporting Items for Systematic Reviews and Meta-Analyses (PRISMA) statement, including the network meta-analysis extension (PRISMA-NMA) [186] (Table S1). The protocol was prospectively registered in PROSPERO (CRD42025641396), and the project received approval from the Institutional Review Board of Tri-Service General Hospital, National Defense Medical Center (TSGHIRB E202516007).

### 4.1. Database Searches and Study Identification

A comprehensive search strategy was implemented across eight electronic databases: PubMed, Embase, ClinicalKey, Cochrane CENTRAL, ProQuest, ScienceDirect, Web of Science, and ClinicalTrials.gov, from inception through 21 January 2025 (Table S2). Search terms combined controlled vocabulary and free-text terms related to GLP-1 receptor agonists, SGLT2 inhibitors, randomized trials, and intestinal obstruction or ileus. Two investigators (PT Tseng and BY Zeng) independently screened titles and abstracts, followed by full-text review of potentially eligible articles. Discrepancies at any stage were resolved through discussion and, when necessary, consultation with a third reviewer. Reference lists of relevant narrative reviews and meta-analyses were screened manually to identify additional RCTs not captured in the initial database search [9,10,22]. No language restrictions were imposed.

### 4.2. Inclusion and Exclusion Criteria

Eligibility criteria were predefined using the Population–Intervention–Comparator–Outcome–Study design (PICOS) framework. Eligible populations were adults without baseline intestinal obstruction. Interventions included any GLP-1 receptor agonist or SGLT2 inhibitor administered at a therapeutic dose approved or under investigation for type 2 diabetes or related cardiometabolic indications. Comparators comprised placebo, standard-of-care therapy, or alternative active treatments. The primary outcome was incident intestinal obstruction (small or large bowel) reported during trial follow-up. Only randomized controlled trials with at least one GLP-1 receptor agonist or SGLT2 inhibitor arm and an eligible comparator arm were included.

To minimize selective reporting and under-ascertainment of harms, we restricted inclusion to RCTs that incorporated systematic adverse-event monitoring or explicitly listed intestinal obstruction, ileus, or related terms as part of safety reporting [187]. Trials exclusively enrolling patients with pre-existing obstruction were excluded to maintain focus on incident or drug-attributable events. Additional exclusion criteria were non-randomized designs, animal or preclinical studies, trials without relevant comparator arms, and studies lacking sufficient data on intestinal obstruction to contribute to the network.

### 4.3. Methodological Quality Appraisal

Two reviewers independently evaluated risk of bias for each included RCT using the original Cochrane Risk of Bias Tool (version 1.0) [188]. The domains assessed included random sequence generation, allocation concealment, blinding of participants and personnel, blinding of outcome assessment, completeness of outcome data, selective reporting, and other potential sources of bias. Any disagreements in domain-level judgments were resolved through consensus with a third senior investigator.

### 4.4. Outcome Definition

For the purposes of this NMA, “intestinal obstruction” encompassed both small bowel obstruction and large bowel obstruction (including colonic and rectal involvement), as recorded in individual trial reports. Given that these clinical entities commonly form a continuum and are often aggregated in adverse-event tables [189], we pooled them into a composite endpoint. Most source trials did not clearly distinguish mechanical obstruction from functional ileus, nor did they provide consistent radiologic or operative confirmation; therefore, these subtypes were not analyzed separately. All-cause study withdrawal, regardless of reason, was extracted as a secondary safety endpoint reflecting overall treatment acceptability.

To explore potential dose–response relationships, we categorized treatment arms according to dosing tiers defined in the original trials and aligned with typical clinical use. Dose nodes were specified a priori as follows:

- Canagliflozin: low 100 mg; high 300 mg.
- Efpeglenatide: low 2 mg; medium 4 mg; high 6 mg.
- Ertugliflozin: low 5 mg; high 15 mg.
- Dapagliflozin: low 2.5 mg; medium 5 mg; high 10 mg.
- Injectable semaglutide: low 0.5 mg; medium 1.0 mg; high 2.4 mg.
- Empagliflozin: low 1–10 mg; high 25–50 mg.

These dose categories were modeled as separate nodes in the network to allow explicit assessment of dose stratification within each molecule.

### 4.5. Data Extraction, Management and Conversion

Data extraction was performed independently by two investigators (PT Tseng and BY Zeng), who used a standardized electronic form to record trial characteristics (design, phase, follow-up duration), participant demographics, baseline risk factors, treatment regimens (agent, dose, route, and frequency), and outcome data for intestinal obstruction and study discontinuation. When necessary information (e.g., number of obstruction events or exact dose) was missing from published reports, attempts were made to contact corresponding authors. Extraction procedures followed recommendations from the Cochrane Handbook for Systematic Reviews of Interventions and best practices in evidence-based pharmacotherapy research [190].

### 4.6. Statistical Analyses

We conducted a frequentist random-effects NMA to integrate direct and indirect evidence across all eligible comparisons, treating each drug–dose combination as an individual treatment node [191]. Analyses were performed using MetaInsight (version 4.0.2, Complex Reviews Support Unit, National Institute for Health Research, London, UK), which implements the netmeta package (version 4.0.2) in R for graph-theoretical NMA estimation [192].

For dichotomous outcomes, continuity corrections were applied to trials with zero events in a single arm, whereas studies with zero events in all arms were excluded from the primary analysis to avoid biased effect estimates [193,194]. Odds ratios (ORs) with 95% confidence intervals (CIs) were calculated and displayed using forest plots for pairwise and network estimates [195]. Treatment ranking probabilities and relative safety hierarchies were derived from the network model. Consistency between direct and indirect evidence was examined using node-splitting approaches, which are especially useful when synthesizing multi-arm pharmacologic trials [192,196]. A two-sided *p* value < 0.05 was considered statistically significant.

### 4.7. Sensitivity Analyses

To test robustness of the primary findings, we repeated the analyses using Bayesian random-effects NMA models with non-informative priors. From these models, we derived SUCRA (surface under the cumulative ranking) values, presented using Litmus Rank-O-Gram and radial SUCRA visualizations, to summarize the probability that each regimen occupies a given rank in terms of intestinal obstruction risk and treatment acceptability [197]. Additional model diagnostics, including deviation-based ranking analyses, were used to identify any influential nodes or incoherent loops in the network [198]. Overall certainty of evidence for each comparison was graded using the GRADE framework adapted for NMA, incorporating risk of bias, indirectness, inconsistency, imprecision, and publication bias [199].

## 5. Conclusions

This network meta-analysis refines the gastrointestinal safety profile of GLP-1 receptor agonists and SGLT2 inhibitors with respect to intestinal obstruction. From the standpoint of randomized clinical trial evidence, most agents within these classes do not appear to increase obstruction risk. A notable exception is high-dose canagliflozin (300 mg/day), which was associated with a modest but statistically significant elevation in obstruction events, whereas liraglutide was linked to a reduced risk.

These agent- and dose-specific findings are relevant for clinicians managing perioperative care, postoperative ileus, and chronic pharmacotherapy in patients with elevated baseline risk for bowel obstruction. Surgeons, endocrinologists, and clinical pharmacologists should consider the potential obstruction signal of high-dose canagliflozin when selecting and titrating SGLT2 inhibitor therapy, particularly in individuals with prior abdominal surgery, adhesions, or known motility disorders. At the same time, the absence of a harmful signal for other GLP-1 receptor agonists and SGLT2 inhibitors, and the potentially protective association for liraglutide, provide reassurance that these agents can generally be used without substantially increasing intestinal obstruction risk. Future work should integrate mechanistic pharmacology, formulation science, and prospective clinical data to optimize glucose-lowering regimens that balance metabolic benefits with gastrointestinal safety.

## Figures and Tables

**Table 1:** League table of the primary outcome: intestine obstruction events.

Liraglutide								* 0.44 [0.24; 0.81]							
1.76 [0.15; 21.01]	Bexagliflozin							0.25 [0.02; 2.76]							
0.81 [0.34; 1.92]	0.46 [0.04; 5.47]	Dulaglutide						0.55 [0.29; 1.02]							
1.32 [0.05; 34.35]	0.75 [0.01; 41.01]	1.64 [0.06; 42.72]	Lixisenatide					0.33 [0.01; 8.19]							
0.74 [0.19; 2.79]	0.42 [0.03; 6.08]	0.91 [0.24; 3.48]	0.56 [0.02; 16.91]	Ertugliflozin				0.60 [0.18; 1.97]							
0.55 [0.10; 3.00]	0.31 [0.02; 5.55]	0.68 [0.13; 3.74]	0.42 [0.01; 14.83]	0.75 [0.10; 5.42]	Tirzepatide			0.80 [0.16; 3.88]							
0.47 [0.21; 1.03]	0.27 [0.02; 3.09]	0.58 [0.26; 1.29]	0.35 [0.01; 9.05]	0.64 [0.18; 2.31]	0.85 [0.16; 4.45]	Inject_ semaglutide		0.94 [0.57; 1.56]							0.33 [0.01; 8.23]
0.46 [0.13; 1.69]	0.26 [0.02; 3.75]	0.57 [0.16; 2.11]	0.35 [0.01; 10.46]	0.63 [0.12; 3.27]	0.84 [0.12; 5.89]	0.98 [0.28; 3.44]	Sotagliflozin	0.95 [0.30; 3.01]							
* 0.44 [0.24; 0.81]	0.25 [0.02; 2.76]	0.55 [0.29; 1.02]	0.33 [0.01; 8.19]	0.60 [0.18; 1.97]	0.80 [0.16; 3.88]	0.94 [0.57; 1.55]	0.95 [0.30; 3.01]	Placebo_ or_Control	1.00 [0.47; 2.09]	0.96 [0.37; 2.45]	0.86 [0.50; 1.49]	0.83 [0.47; 1.46]		0.40 [0.05; 3.43]	0.39 [0.15; 1.04]
0.44 [0.17; 1.14]	0.25 [0.02; 3.08]	0.54 [0.21; 1.43]	0.33 [0.01; 8.88]	0.60 [0.15; 2.42]	0.80 [0.14; 4.56]	0.94 [0.38; 2.29]	0.95 [0.24; 3.73]	1.00 [0.47; 2.09]	Exenatide						
0.42 [0.14; 1.29]	0.24 [0.02; 3.16]	0.52 [0.17; 1.62]	0.32 [0.01; 8.98]	0.57 [0.13; 2.61]	0.77 [0.12; 4.82]	0.90 [0.31; 2.61]	0.92 [0.21; 4.04]	0.96 [0.37; 2.45]	0.96 [0.29; 3.19]	Albiglutide					
* 0.38 [0.17; 0.86]	0.22 [0.02; 2.53]	0.47 [0.21; 1.08]	0.29 [0.01; 7.40]	0.52 [0.14; 1.91]	0.69 [0.13; 3.67]	0.81 [0.39; 1.70]	0.82 [0.23; 2.94]	0.86 [0.50; 1.49]	0.87 [0.35; 2.17]	0.90 [0.30; 2.67]	Empagliflozin		0.33 [0.01; 8.21]		
* 0.37 [0.16; 0.84]	0.21 [0.02; 2.45]	0.45 [0.20; 1.05]	0.28 [0.01; 7.15]	0.50 [0.13; 1.86]	0.66 [0.12; 3.56]	0.78 [0.37; 1.66]	0.79 [0.22; 2.85]	0.83 [0.47; 1.46]	0.83 [0.33; 2.12]	0.87 [0.29; 2.60]	0.96 [0.44; 2.11]	Dapagliflozin			
0.13 [0.00; 3.46]	0.07 [0.00; 4.09]	0.16 [0.01; 4.30]	0.10 [0.00; 9.18]	0.17 [0.01; 5.49]	0.23 [0.01; 8.52]	0.27 [0.01; 7.24]	0.27 [0.01; 8.62]	0.29 [0.01; 7.42]	0.29 [0.01; 8.10]	0.30 [0.01; 8.84]	0.33 [0.01; 8.21]	0.35 [0.01; 9.36]	Oral_ semaglutide		
0.18 [0.02; 1.64]	0.10 [0.00; 2.51]	0.22 [0.02; 2.05]	0.13 [0.00; 6.31]	0.24 [0.02; 2.80]	0.32 [0.02; 4.61]	0.38 [0.04; 3.42]	0.38 [0.03; 4.37]	0.40 [0.05; 3.43]	0.40 [0.04; 3.90]	0.42 [0.04; 4.36]	0.46 [0.05; 4.26]	0.48 [0.05; 4.44]	1.39 [0.03; 68.46]	Efpeglenatide	
* 0.17 [0.06; 0.52]	0.10 [0.01; 1.29]	* 0.21 [0.07; 0.66]	0.13 [0.00; 3.66]	0.23 [0.05; 1.06]	0.31 [0.05; 1.96]	0.37 [0.13; 1.04]	0.37 [0.09; 1.64]	* 0.39 [0.15; 0.99]	0.39 [0.12; 1.29]	0.41 [0.11; 1.53]	0.45 [0.15; 1.33]	0.47 [0.16; 1.40]	1.36 [0.05; 39.95]	0.98 [0.09; 10.15]	Canagliflozin

Data present as OR [95%CIs]. Pairwise (upper-right portion) and network (lower-left portion) meta-analysis results are presented as estimate effect sizes for the outcome of events of intestine obstruction. The gray background indicated the regimens of comparison. Interventions are reported in order of mean ranking of beneficially prophylactic effect on events of intestine obstruction, and outcomes are expressed as odds ratio (OR) (95% confidence intervals) (95%CIs). For the pairwise meta-analyses, OR of less than 1 indicate that the treatment specified in the row had a more beneficial effect than that specified in the column. For the network meta-analysis (NMA), OR of less than 1 indicate that the treatment specified in the column had a more beneficial effect than that specified in the row. Bold results marked with * indicate statistical significance. Abbreviation: 95%CIs: 95% confidence intervals; NMA: network meta-analysis; OR: odds ratio.

**Table 2:** League table of the primary outcome: intestine obstruction events in aspect of various dosage subgroup.

Bexagliflozin											0.25 [0.02; 2.76]														
0.57 [0.05; 6.76]	Liraglutide										* 0.44 [0.24; 0.81]														
0.63 [0.03; 11.47]	1.10 [0.19; 6.34]	Ertugliflozin_ low_dosage						0.50 [0.09; 2.73]			0.40 [0.08; 2.06]														
0.46 [0.04; 5.47]	0.81 [0.34; 1.92]	0.73 [0.13; 4.23]	Dulaglutide								0.55 [0.29; 1.02]														
0.75 [0.01; 41.01]	1.32 [0.05; 34.35]	1.20 [0.03; 43.72]	1.64 [0.06; 42.72]	Lixisenatide							0.33 [0.01; 8.19]														
0.74 [0.01; 40.67]	1.31 [0.05; 34.08]	1.19 [0.03; 43.37]	1.62 [0.06; 42.39]	0.99 [0.01; 91.70]	Tirzepatide_ medium_dosage						0.34 [0.01; 8.28]														
0.74 [0.01; 40.29]	1.30 [0.05; 33.76]	1.18 [0.03; 42.96]	1.61 [0.06; 41.99]	0.98 [0.01; 90.84]	0.99 [0.01; 91.79]	Tirzepatide_ low_dosage					0.34 [0.01; 8.35]														
0.74 [0.01; 40.29]	1.30 [0.05; 33.76]	1.18 [0.03; 42.96]	1.61 [0.06; 41.99]	0.98 [0.01; 90.84]	0.99 [0.01; 91.79]	1.00 [0.01; 92.67]	Tirzepatide_ high_dosage				0.34 [0.01; 8.35]														
0.31 [0.02; 4.84]	0.55 [0.13; 2.35]	0.50 [0.09; 2.73]	0.68 [0.16; 2.93]	0.42 [0.01; 13.28]	0.42 [0.01; 13.43]	0.43 [0.01; 13.56]	0.43 [0.01; 13.56]	Ertugliflozin_ high_dosage			0.80 [0.21; 2.98]														
0.26 [0.02; 3.09]	0.46 [0.21; 1.05]	0.42 [0.07; 2.37]	0.57 [0.25; 1.31]	0.35 [0.01; 9.02]	0.35 [0.01; 9.12]	0.36 [0.01; 9.20]	0.36 [0.01; 9.20]	0.84 [0.20; 3.49]	Inject_ semaglutide_ high_dosage		0.92 [0.53; 1.59]			3.00 [0.12; 73.86]											
0.26 [0.02; 3.75]	0.46 [0.13; 1.69]	0.42 [0.06; 3.10]	0.57 [0.16; 2.11]	0.35 [0.01; 10.46]	0.35 [0.01; 10.58]	0.36 [0.01; 10.68]	0.36 [0.01; 10.68]	0.84 [0.15; 4.79]	0.99 [0.28; 3.54]	Sotagliflozin	0.95 [0.30; 3.01]			.											
0.25 [0.02; 2.76]	* 0.44 [0.24; 0.81]	0.40 [0.08; 2.06]	0.55 [0.29; 1.02]	0.33 [0.01; 8.19]	0.34 [0.01; 8.28]	0.34 [0.01; 8.35]	0.34 [0.01; 8.35]	0.80 [0.21; 2.98]	0.95 [0.55; 1.63]	0.95 [0.30; 3.01]	Placebo_ or_Control	1.00 [0.47; 2.09]		0.79 [0.18; 3.44]	0.96 [0.37; 2.45]	0.89 [0.50; 1.61]	0.91 [0.32; 2.61]	0.83 [0.47; 1.46]	0.67 [0.13; 3.49]	0.50 [0.05; 5.54]	.	0.40 [0.13; 1.23]	0.33 [0.03; 3.21]	0.34 [0.01; 8.40]	0.34 [0.08; 1.48]
0.25 [0.02; 3.08]	0.44 [0.17; 1.14]	0.40 [0.07; 2.41]	0.54 [0.21; 1.43]	0.33 [0.01; 8.88]	0.34 [0.01; 8.97]	0.34 [0.01; 9.06]	0.34 [0.01; 9.06]	0.80 [0.18; 3.61]	0.95 [0.38; 2.37]	0.95 [0.24; 3.73]	1.00 [0.47; 2.09]	Exenatide													
0.25 [0.00; 24.71]	0.43 [0.01; 23.23]	0.39 [0.01; 27.92]	0.54 [0.01; 28.88]	0.33 [0.00; 52.33]	0.33 [0.00; 52.87]	0.33 [0.00; 53.37]	0.33 [0.00; 53.37]	0.78 [0.01; 49.84]	0.93 [0.02; 49.65]	0.94 [0.02; 56.64]	0.98 [0.02; 50.35]	0.99 [0.02; 54.17]	Dapagliflozin_ medium_dosage											0.32 [0.01; 7.80]	
0.24 [0.02; 3.42]	0.42 [0.12; 1.53]	0.38 [0.05; 2.82]	0.52 [0.14; 1.91]	0.32 [0.01; 9.54]	0.32 [0.01; 9.65]	0.33 [0.01; 9.74]	0.33 [0.01; 9.74]	0.77 [0.13; 4.36]	0.91 [0.27; 3.12]	0.91 [0.18; 4.61]	0.96 [0.31; 2.99]	0.96 [0.25; 3.74]	0.98 [0.02; 58.79]	Inject_ semaglutide_ medium_dosage					0.78 [0.17; 3.62]						0.33 [0.01; 8.23]
0.24 [0.02; 3.16]	0.42 [0.14; 1.29]	0.38 [0.06; 2.54]	0.52 [0.17; 1.62]	0.32 [0.01; 8.98]	0.32 [0.01; 9.08]	0.33 [0.01; 9.17]	0.33 [0.01; 9.17]	0.77 [0.15; 3.86]	0.91 [0.31; 2.70]	0.92 [0.21; 4.04]	0.96 [0.37; 2.45]	0.96 [0.29; 3.19]	0.98 [0.02; 55.94]	1.00 [0.23; 4.38]	Albiglutide										
0.22 [0.02; 2.57]	* 0.38 [0.17; 0.89]	0.35 [0.06; 1.98]	0.47 [0.20; 1.11]	0.29 [0.01; 7.50]	0.29 [0.01; 7.58]	0.30 [0.01; 7.66]	0.30 [0.01; 7.66]	0.69 [0.16; 2.93]	0.83 [0.37; 1.83]	0.83 [0.23; 3.01]	0.87 [0.48; 1.56]	0.87 [0.34; 2.24]	0.89 [0.02; 47.40]	0.91 [0.25; 3.26]	0.91 [0.30; 2.74]	Empagliflozin_ low_dosage	0.83 [0.25; 2.82]								
0.20 [0.02; 2.67]	0.35 [0.11; 1.10]	0.32 [0.05; 2.15]	0.44 [0.14; 1.38]	0.27 [0.01; 7.58]	0.27 [0.01; 7.66]	0.27 [0.01; 7.73]	0.27 [0.01; 7.73]	0.64 [0.13; 3.27]	0.76 [0.25; 2.30]	0.77 [0.17; 3.42]	0.81 [0.31; 2.09]	0.81 [0.24; 2.71]	0.82 [0.01; 47.14]	0.84 [0.19; 3.72]	0.84 [0.22; 3.21]	0.93 [0.34; 2.51]	Empagliflozin_ high_dosage				0.33 [0.01; 8.21]				
0.21 [0.02; 2.44]	* 0.36 [0.16; 0.84]	0.33 [0.06; 1.88]	0.45 [0.20; 1.05]	0.28 [0.01; 7.13]	0.28 [0.01; 7.21]	0.28 [0.01; 7.28]	0.28 [0.01; 7.28]	0.66 [0.16; 2.77]	0.79 [0.36; 1.73]	0.79 [0.22; 2.85]	0.83 [0.47; 1.46]	0.83 [0.33; 2.12]	0.84 [0.02; 43.28]	0.86 [0.24; 3.09]	0.86 [0.29; 2.59]	0.95 [0.42; 2.15]	1.03 [0.34; 3.13]	Dapagliflozin_high_dosage						0.34 [0.01; 8.44]	
0.17 [0.01; 2.65]	0.30 [0.07; 1.30]	0.27 [0.03; 2.25]	0.37 [0.08; 1.63]	0.22 [0.01; 7.24]	0.23 [0.01; 7.32]	0.23 [0.01; 7.39]	0.23 [0.01; 7.39]	0.54 [0.08; 3.55]	0.64 [0.15; 2.70]	0.64 [0.11; 3.78]	0.67 [0.17; 2.60]	0.68 [0.14; 3.15]	0.69 [0.01; 44.05]	0.70 [0.19; 2.65]	0.70 [0.14; 3.64]	0.77 [0.18; 3.37]	0.84 [0.16; 4.37]	0.81 [0.19; 3.51]	Inject_ semaglutide_ low_dosage						
0.13 [0.00; 3.74]	0.22 [0.02; 2.63]	0.20 [0.01; 3.67]	0.27 [0.02; 3.27]	0.17 [0.00; 9.14]	0.17 [0.00; 9.24]	0.17 [0.00; 9.33]	0.17 [0.00; 9.33]	0.40 [0.03; 6.20]	0.48 [0.04; 5.59]	0.48 [0.03; 6.86]	0.50 [0.05; 5.54]	0.50 [0.04; 6.22]	0.51 [0.01; 51.42]	0.52 [0.04; 7.47]	0.52 [0.04; 6.90]	0.58 [0.05; 6.83]	0.62 [0.05; 8.26]	0.61 [0.05; 7.14]	0.74 [0.05; 11.71]	Efpeglenatide_ medium_dosage			0.67 [0.11; 3.99]		
0.07 [0.00; 4.11]	0.12 [0.00; 3.53]	0.11 [0.00; 4.44]	0.15 [0.00; 4.39]	0.09 [0.00; 9.16]	0.09 [0.00; 9.25]	0.09 [0.00; 9.34]	0.09 [0.00; 9.34]	0.21 [0.01; 7.79]	0.25 [0.01; 7.54]	0.26 [0.01; 8.78]	0.27 [0.01; 7.60]	0.27 [0.01; 8.27]	0.27 [0.00; 47.85]	0.28 [0.01; 9.57]	0.28 [0.01; 9.02]	0.31 [0.01; 8.84]	0.33 [0.01; 8.21]	0.32 [0.01; 9.62]	0.40 [0.01; 14.66]	0.54 [0.01; 32.82]	Oral_ semaglutide				
0.11 [0.01; 1.56]	* 0.20 [0.06; 0.67]	0.18 [0.03; 1.27]	* 0.25 [0.07; 0.84]	0.15 [0.01; 4.40]	0.15 [0.01; 4.45]	0.15 [0.01; 4.49]	0.15 [0.01; 4.49]	0.36 [0.07; 1.95]	0.43 [0.13; 1.40]	0.43 [0.09; 2.05]	0.46 [0.16; 1.29]	0.46 [0.13; 1.64]	0.46 [0.01; 27.24]	0.48 [0.11; 2.12]	0.47 [0.12; 1.93]	0.52 [0.16; 1.73]	0.57 [0.14; 2.33]	0.55 [0.17; 1.80]	0.68 [0.13; 3.64]	0.91 [0.07; 12.45]	1.70 [0.05; 56.30]	Canagliflozin_ low_dosage			0.53 [0.15; 1.96]
0.08 [0.00; 2.26]	0.15 [0.01; 1.53]	0.13 [0.01; 2.18]	0.18 [0.02; 1.91]	0.11 [0.00; 5.61]	0.11 [0.00; 5.67]	0.11 [0.00; 5.72]	0.11 [0.00; 5.72]	0.27 [0.02; 3.66]	0.32 [0.03; 3.25]	0.32 [0.03; 4.03]	0.33 [0.03; 3.21]	0.33 [0.03; 3.63]	0.34 [0.00; 31.89]	0.35 [0.03; 4.39]	0.35 [0.03; 4.04]	0.38 [0.04; 3.98]	0.41 [0.04; 4.84]	0.40 [0.04; 4.16]	0.50 [0.04; 6.92]	0.67 [0.11; 3.99]	1.24 [0.02; 70.48]	0.73 [0.06; 8.86]	Efpeglenatide_ high_dosage		
0.08 [0.00; 2.14]	0.14 [0.01; 1.45]	0.12 [0.01; 2.07]	0.17 [0.02; 1.81]	0.10 [0.00; 5.28]	0.10 [0.00; 5.34]	0.11 [0.00; 5.39]	0.11 [0.00; 5.39]	0.25 [0.02; 3.46]	0.29 [0.03; 3.09]	0.30 [0.02; 3.82]	0.31 [0.03; 3.05]	0.31 [0.03; 3.44]	0.32 [0.01; 7.80]	0.32 [0.03; 4.16]	0.32 [0.03; 3.83]	0.36 [0.03; 3.78]	0.39 [0.03; 4.59]	0.37 [0.04; 3.68]	0.46 [0.03; 6.55]	0.62 [0.02; 17.03]	1.16 [0.02; 66.34]	0.68 [0.06; 8.40]	0.93 [0.04; 23.22]	Dapagliflozin_ low_dosage	
0.07 [0.01; 1.05]	* 0.13 [0.04; 0.47]	* 0.12 [0.02; 0.87]	* 0.16 [0.04; 0.59]	0.10 [0.00; 2.92]	0.10 [0.00; 2.96]	0.10 [0.00; 2.98]	0.10 [0.00; 2.98]	0.23 [0.04; 1.34]	* 0.28 [0.08; 0.99]	0.28 [0.06; 1.42]	* 0.29 [0.09; 0.92]	0.29 [0.07; 1.15]	0.30 [0.00; 18.00]	0.31 [0.07; 1.39]	0.31 [0.07; 1.35]	0.34 [0.09; 1.22]	0.36 [0.08; 1.62]	0.35 [0.10; 1.27]	0.43 [0.08; 2.43]	0.58 [0.04; 8.36]	1.09 [0.03; 37.38]	0.64 [0.21; 1.97]	0.88 [0.07; 11.11]	0.94 [0.07; 12.16]	Canagliflozin_ high_dosage

Data present as OR [95%CIs]. Pairwise (upper-right portion) and network (lower-left portion) meta-analysis results are presented as estimate effect sizes for the outcome of events of intestine obstruction. The gray background indicated the regimens of comparison. Interventions are reported in order of mean ranking of beneficially prophylactic effect on events of intestine obstruction, and outcomes are expressed as odds ratio (OR) (95% confidence intervals) (95%CIs). For the pairwise meta-analyses, OR of less than 1 indicate that the treatment specified in the row got more beneficial effect than that specified in the column. For the network meta-analysis (NMA), OR of less than 1 indicate that the treatment specified in the column got more beneficial effect than that specified in the row. Bold results marked with * indicate statistical significance. Abbreviation: 95%CIs: 95% confidence intervals; NMA: network meta-analysis; OR: odds ratio.

## Data Availability

The original contributions presented in this study are included in the article/Supplementary Materials. Further inquiries can be directed to the corresponding authors.

## Supplementary Materials

The following supporting information can be downloaded at: https://www.mdpi.com/article/10.3390/ijms27020608/s1. Reference [200] is cited in Supplementary Materials.
