# Supplementary material for: Agent- and Dose-Specific Intestinal Obstruction Safety of GLP-1 Receptor Agonists and SGLT2 Inhibitors: A Network Meta-Analysis of Randomized Trials
Source: Int J Mol Sci. 2026 Jan 7;27(2):608. doi: 10.3390/ijms27020608 (PMC12841245; doi:10.3390/ijms27020608)
Supplement: Supplementary file 1 [file ijms-27-00608-s001.zip › ijms-4077317-supplementary.pdf]

<u>List of content in supplement materials</u>

Agent- and Dose-Specific Intestinal Obstruction Safety of GLP-1 Receptor Agonists and SGLT2 Inhibitors

A Network Meta-analysis of Randomized Trials

Jiann-Jy Chen, et al.

Figure S1	Network structure of NMA of acceptability: drop-out rate
Figure S2	Forest plot of NMA of acceptability: drop-out rate
Figure S3	(A) Individual study result of primary outcome: intestine obstruction events
	(B) Individual study result of primary outcome: intestine obstruction events in aspect of various dosage subgroup
	(C) Individual study result of acceptability: drop-out rate
Figure S4	(A) Bayesian-based forest plot of NMA of primary outcome: intestine obstruction events
	(B) Bayesian-based forest plot of NMA of primary outcome: intestine obstruction events in aspect of various dosage subgroup
	(C) Bayesian-based forest plot of NMA of acceptability: drop-out rate
Figure S5	(A) Bayesian-based Litmus Rank-O-Gram rank plot of primary outcome: intestine obstruction events
	(B) Bayesian-based radial surface under the cumulative ranking of primary outcome: intestine obstruction events
	(C) Bayesian-based Litmus Rank-O-Gram rank plot of primary outcome: intestine obstruction events in aspect of various dosage subgroup
	(D) Bayesian-based radial surface under the cumulative ranking of primary outcome: intestine obstruction events in aspect of various dosage subgroup
	(E) Bayesian-based Litmus Rank-O-Gram rank plot of acceptability: drop-out rate
	(F) Bayesian-based radial surface under the cumulative ranking of acceptability: drop-out rate
Figure S6	(A) Bayesian-based residual deviance NMA/UME model of primary outcome: intestine obstruction events
	(B) Bayesian-based per-arm residual deviance of primary outcome: intestine obstruction events
	(C) Bayesian-based leverage plot of primary outcome: intestine obstruction events
	(D) Bayesian-based residual deviance NMA/UME model of primary outcome: intestine obstruction events in aspect of various dosage subgroup

	(E) Bayesian-based per-arm residual deviance of primary outcome: intestine obstruction events in aspect of various dosage subgroup
	(F) Bayesian-based leverage plot of primary outcome: intestine obstruction events in aspect of various dosage subgroup
	(G) Bayesian-based residual deviance NMA/UME model of acceptability: drop-out rate
	(H) Bayesian-based per-arm residual deviance of acceptability: drop-out rate
	(I) Bayesian-based leverage plot of acceptability: drop-out rate
Figure S7	(A) Overview of risk of bias
	(B) Detailed risk of bias in each study
Table S1	PRISMA 2020 checklist of the current network meta-analysis
Table S2	Keyword used in each database and search results
Table S3	Excluded studies and reason
Table S4	Characteristics of the included studies
Table S5	League table of NMA of acceptability: drop-out rate
Table S6	(A) SUCRA (Surface under the cumulative ranking) of primary outcome: intestine obstruction events
	(B) SUCRA (Surface under the cumulative ranking) of primary outcome: intestine obstruction events in aspect of various dosage subgroup
	(C) SUCRA (Surface under the cumulative ranking) of acceptability: drop-out rate
Table S7	(A) Inconsistency within the network meta-analysis of primary outcome: intestine obstruction events
	(B) Inconsistency within the network meta-analysis of primary outcome: intestine obstruction events in aspect of various dosage subgroup
	(C) Inconsistency within the network meta-analysis of acceptability: drop-out rate
Table S8	(A) GRADE of primary outcome: intestine obstruction events
	(B) GRADE of primary outcome: intestine obstruction events in aspect of various dosage subgroup
	(C) GRADE of acceptability: drop-out rate

Figure S1 network structure of NMA of acceptability: drop-out rate

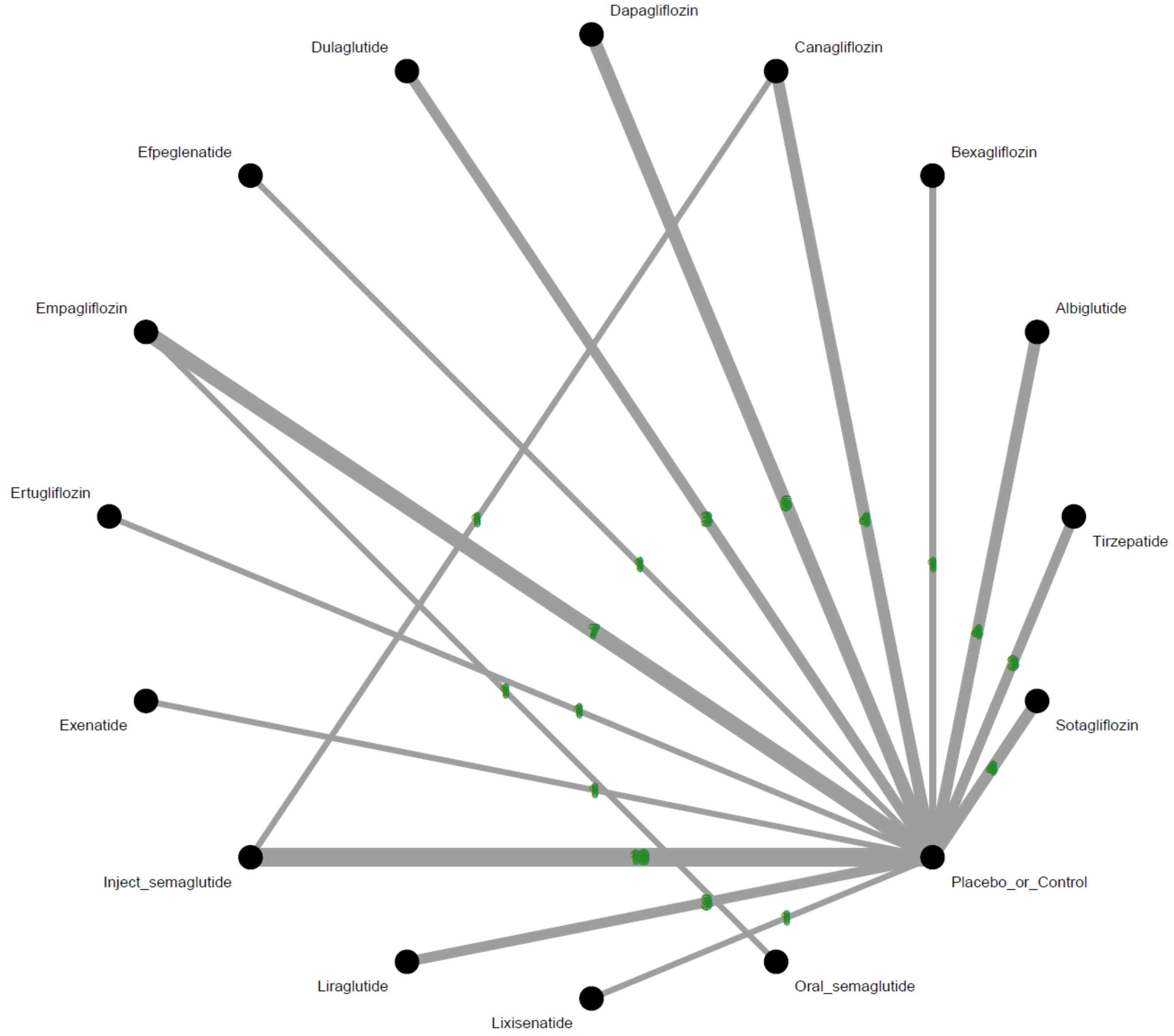

Figure legend of Figure S1

The structure of the network meta-analysis. The lines between nodes represent direct comparisons from various trials, with the numbers over the lines indicating the number of trials providing these comparisons for each specific treatment. The thickness of the lines corresponds to the number of trials linked to the network.

Abbreviation for Figure S1:

95%CIs: 95% confidence intervals; GLP-1 agonist: glucagon-like peptide-1 agonist; NMA: network meta-analysis; OR: odds ratio; RCT: randomized controlled trial; SGLT2 inhibitor: sodium–glucose cotransporter 2 inhibitor

Figure S2 forest plot of NMA of acceptability: drop-out rate

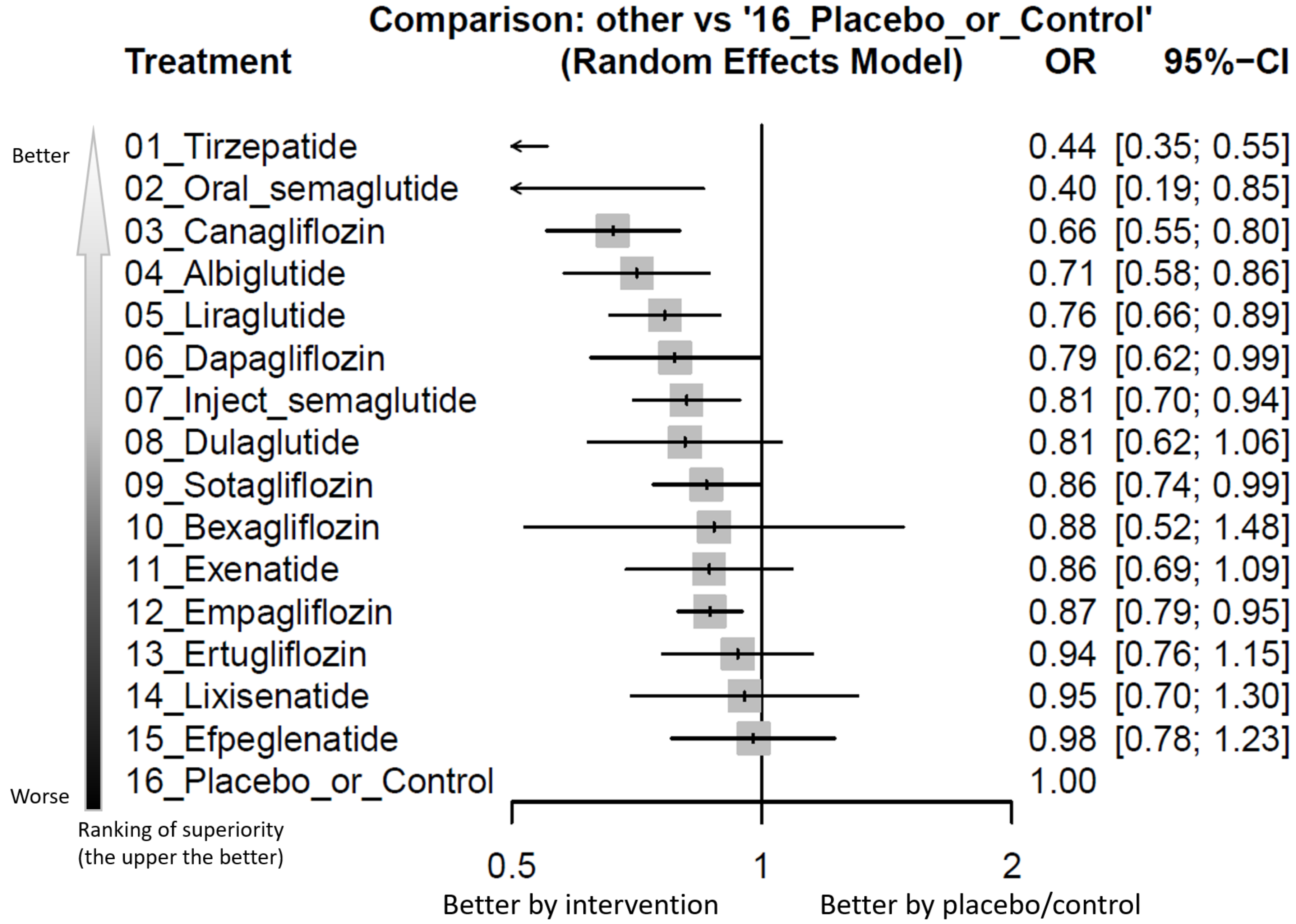

Abbreviation for Figure S2:

95%CIs: 95% confidence intervals; GLP-1 agonist: glucagon-like peptide-1 agonist; NMA: network meta-analysis; OR: odds ratio; RCT: randomized controlled trial; SGLT2 inhibitor: sodium–glucose cotransporter 2 inhibitor

Figure S3A Individual study result of primary outcome: intestine obstruction events

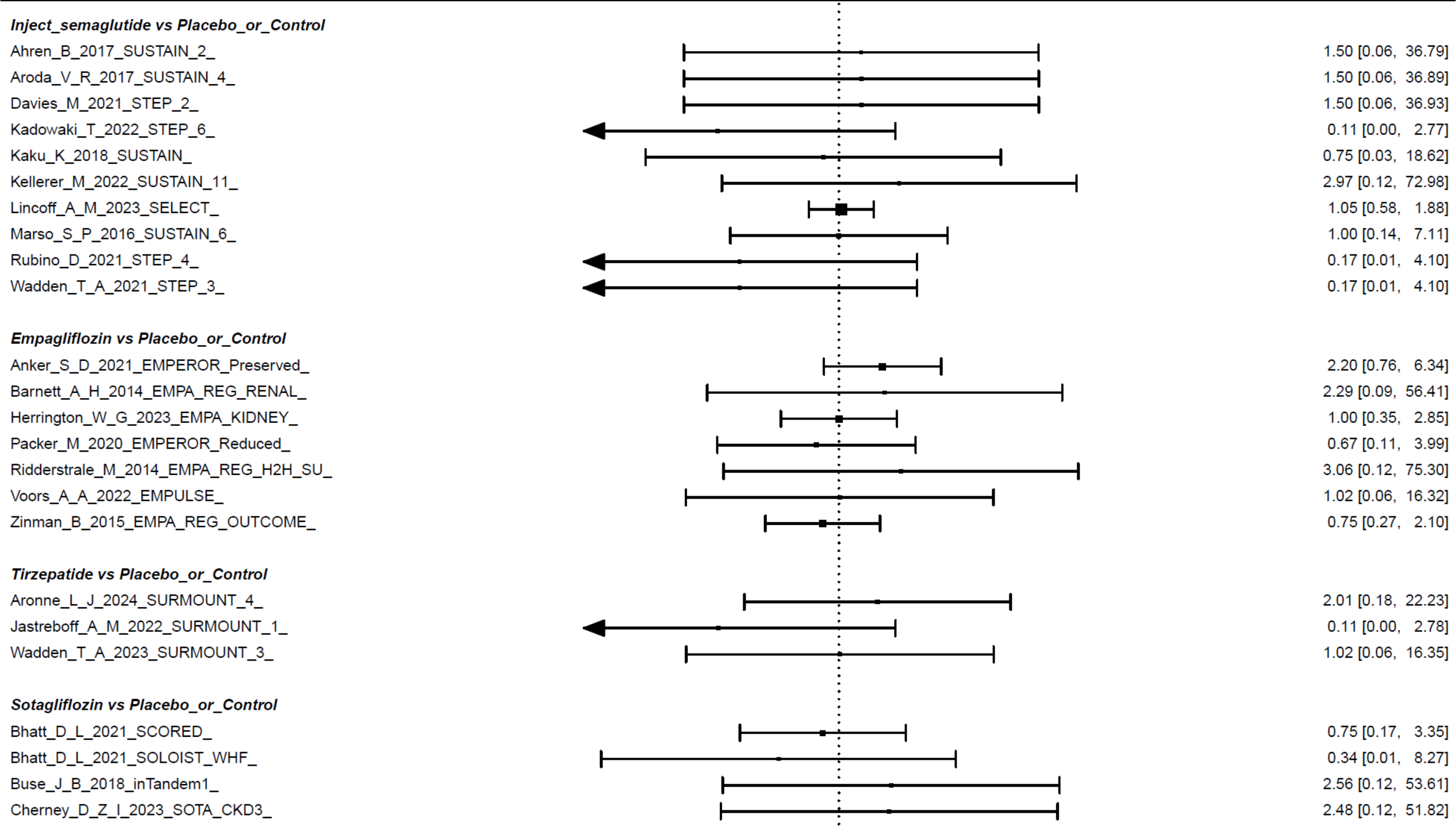

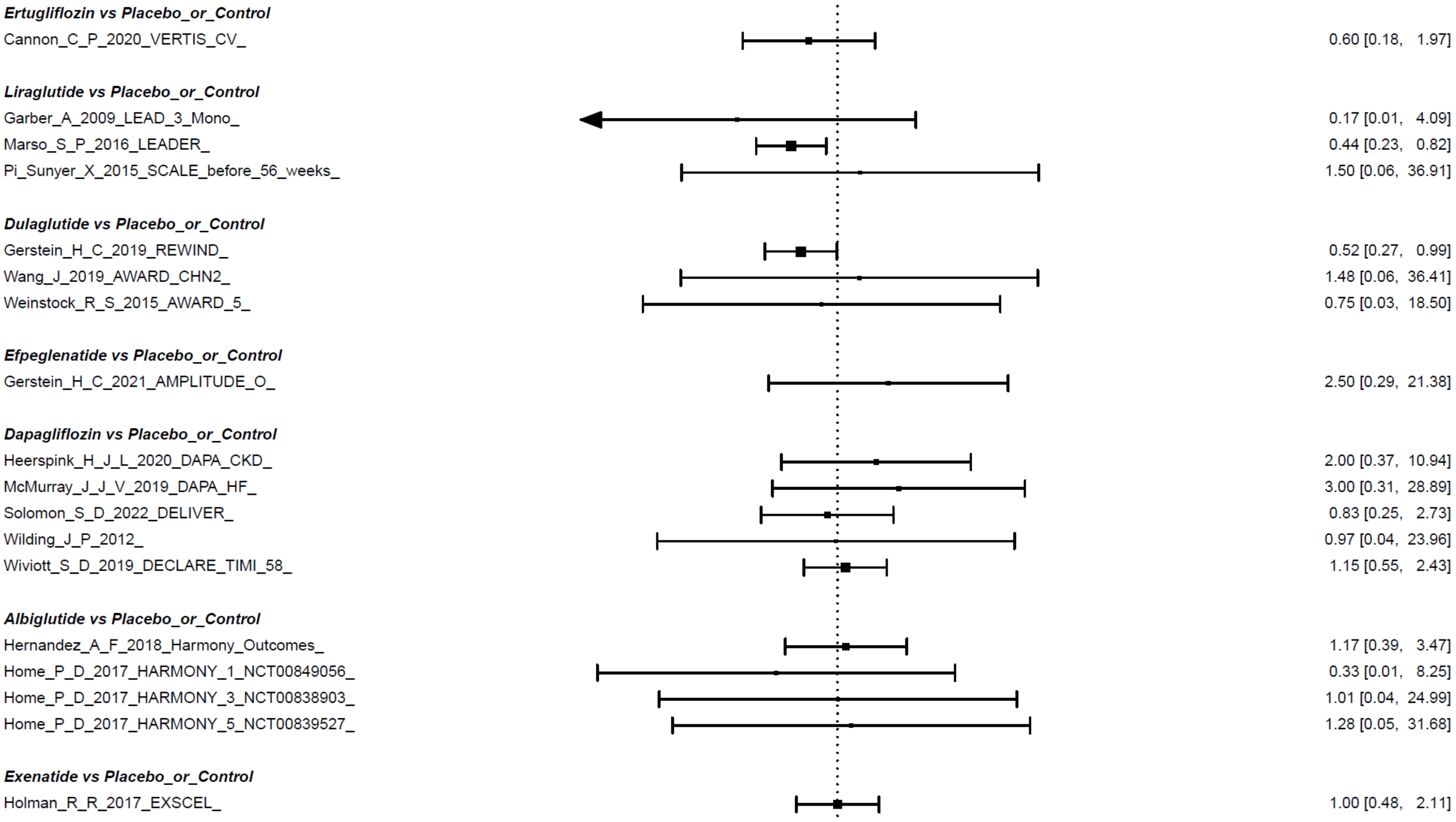
Ertugliflozin vs Placebo_or_Control
Cannon_C_P_2020_VERTIS_CV_ 0.60 [0.18, 1.97]
Liraglutide vs Placebo_or_Control
Garber_A_2009_LEAD_3_Mono_ 0.17 [0.01, 4.09]
Marso_S_P_2016_LEADER_ 0.44 [0.23, 0.82]
Pi_Sunyer_X_2015_SCALE_before_56_weeks_ 1.50 [0.06, 36.91]
Dulaglutide vs Placebo_or_Control
Gerstein_H_C_2019_REWIND_ 0.52 [0.27, 0.99]
Wang_J_2019_AWARD_CHN2_ 1.48 [0.06, 36.41]
Weinstock_R_S_2015_AWARD_5_ 0.75 [0.03, 18.50]
Efpeglenatide vs Placebo_or_Control
Gerstein_H_C_2021_AMPLITUDE_O_ 2.50 [0.29, 21.38]
Dapagliflozin vs Placebo_or_Control
Heerspink_H_J_L_2020_DAPA_CKD_ 2.00 [0.37, 10.94]
McMurray_J_J_V_2019_DAPA_HF_ 3.00 [0.31, 28.89]
Solomon_S_D_2022_DELIVER_ 0.83 [0.25, 2.73]
Wilding_J_P_2012_ 0.97 [0.04, 23.96]
Wiviott_S_D_2019_DECLARE_TIMI_58_ 1.15 [0.55, 2.43]
Albiglutide vs Placebo_or_Control
Hernandez_A_F_2018_Harmony_Outcomes_ 1.17 [0.39, 3.47]
Home_P_D_2017_HARMONY_1_NCT00849056_ 0.33 [0.01, 8.25]
Home_P_D_2017_HARMONY_3_NCT00838903_ 1.01 [0.04, 24.99]
Home_P_D_2017_HARMONY_5_NCT00839527_ 1.28 [0.05, 31.68]
Exenatide vs Placebo_or_Control
Holman_R_R_2017_EXSCEL_ 1.00 [0.48, 2.11]

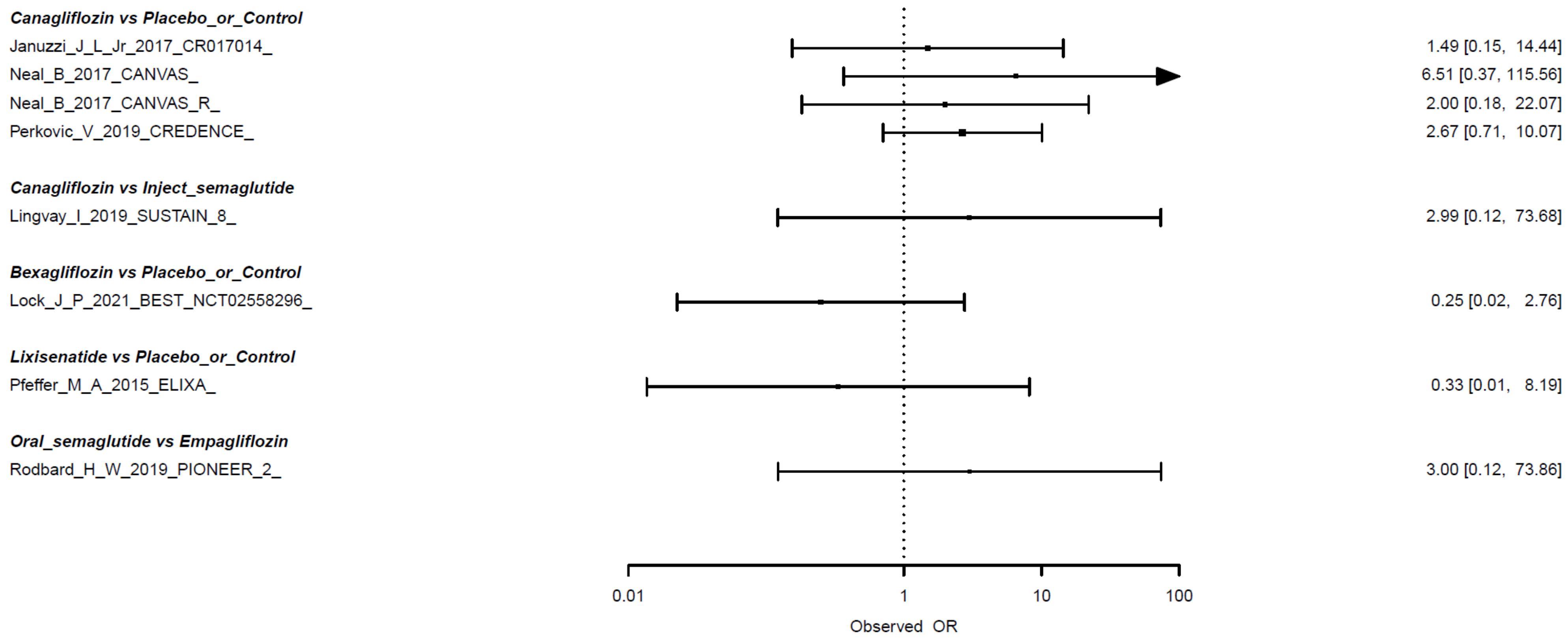

Canagliflozin vs Placebo_or_Control
Januzzi_J_L_Jr_2017_CR017014_
1.49 [0.15, 14.44]
Neal_B_2017_CANVAS_
6.51 [0.37, 115.56]
Neal_B_2017_CANVAS_R_
2.00 [0.18, 22.07]
Perkovic_V_2019_CREDENCE_
2.67 [0.71, 10.07]
Canagliflozin vs Inject_semaglutide
Lingvay_I_2019_SUSTAIN_8_
2.99 [0.12, 73.68]
Bexagliflozin vs Placebo_or_Control
Lock_J_P_2021_BEST_NCT02558296_
0.25 [0.02, 2.76]
Lixisenatide vs Placebo_or_Control
Pfeffer_M_A_2015_ELIXA_
0.33 [0.01, 8.19]
Oral_semaglutide vs Empagliflozin
Rodbard_H_W_2019_PIONEER_2_
3.00 [0.12, 73.86]
0.01
1
10
100
Observed OR

Figure S3B Individual study result of primary outcome: intestine obstruction events in aspect of various dosage subgroup

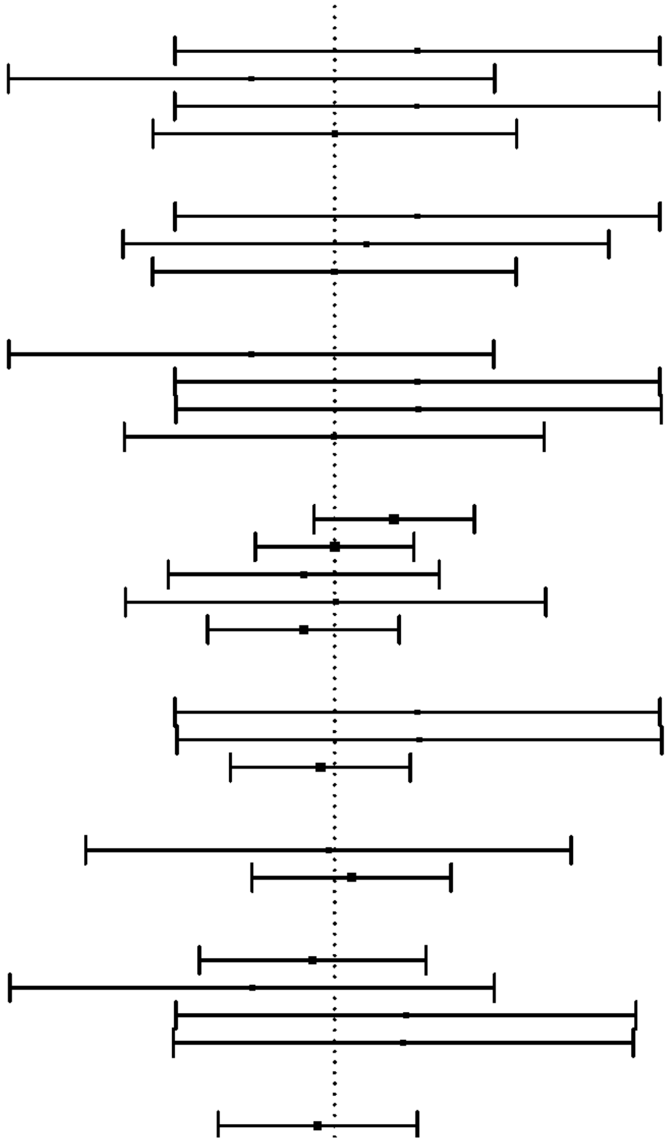

Inject_semaglutide_medium_dosage vs Placebo_or_Control	
Ahren_B_2017_SUSTAIN_2_2	2.99 [0.12, 73.68]
Kadowaki_T_2022_STEP_6_2	0.33 [0.01, 8.28]
Kellerer_M_2022_SUSTAIN_11_	2.97 [0.12, 72.98]
Marso_S_P_2016_SUSTAIN_6_	1.00 [0.09, 11.08]
Inject_semaglutide_low_dosage vs Placebo_or_Control	
Aroda_V_R_2017_SUSTAIN_4_2	2.99 [0.12, 73.68]
Kaku_K_2018_SUSTAIN_2	1.52 [0.06, 37.49]
Marso_S_P_2016_SUSTAIN_6_	1.00 [0.09, 11.02]
Inject_semaglutide_low_dosage vs Inject_semaglutide_medium_dosage	
Ahren_B_2017_SUSTAIN_2_1	0.33 [0.01, 8.19]
Aroda_V_R_2017_SUSTAIN_4_1	2.99 [0.12, 73.68]
Kaku_K_2018_SUSTAIN_1	3.04 [0.12, 74.94]
Marso_S_P_2016_SUSTAIN_6_	1.00 [0.06, 15.94]
Empagliflozin_low_dosage vs Placebo_or_Control	
Anker_S_D_2021_EMPEROR_Preserved_	2.20 [0.76, 6.34]
Herrington_W_G_2023_EMPA_KIDNEY_	1.00 [0.35, 2.85]
Packer_M_2020_EMPEROR_Reduced_	0.67 [0.11, 3.99]
Voors_A_A_2022_EMPULSE_	1.02 [0.06, 16.32]
Zinman_B_2015_EMPA_REG_OUTCOME_	0.66 [0.19, 2.35]
Empagliflozin_high_dosage vs Placebo_or_Control	
Barnett_A_H_2014_EMPA_REG_RENAL_2	2.99 [0.12, 73.69]
Ridderstrale_M_2014_EMPA_REG_H2H_SU_	3.06 [0.12, 75.30]
Zinman_B_2015_EMPA_REG_OUTCOME_	0.83 [0.25, 2.72]
Empagliflozin_high_dosage vs Empagliflozin_low_dosage	
Barnett_A_H_2014_EMPA_REG_RENAL_1	0.92 [0.04, 22.81]
Zinman_B_2015_EMPA_REG_OUTCOME_	1.25 [0.34, 4.67]
Sotagliflozin vs Placebo_or_Control	
Bhatt_D_L_2021_SCORED_	0.75 [0.17, 3.35]
Bhatt_D_L_2021_SOLOIST_WHF_	0.34 [0.01, 8.27]
Buse_J_B_2018_inTandem1_	2.56 [0.12, 53.61]
Cherney_D_Z_I_2023_SOTA_CKD3_	2.48 [0.12, 51.82]
Ertugliflozin_high_dosage vs Placebo_or_Control	
Cannon_C_P_2020_VERTIS_CV_	0.80 [0.21, 2.98]

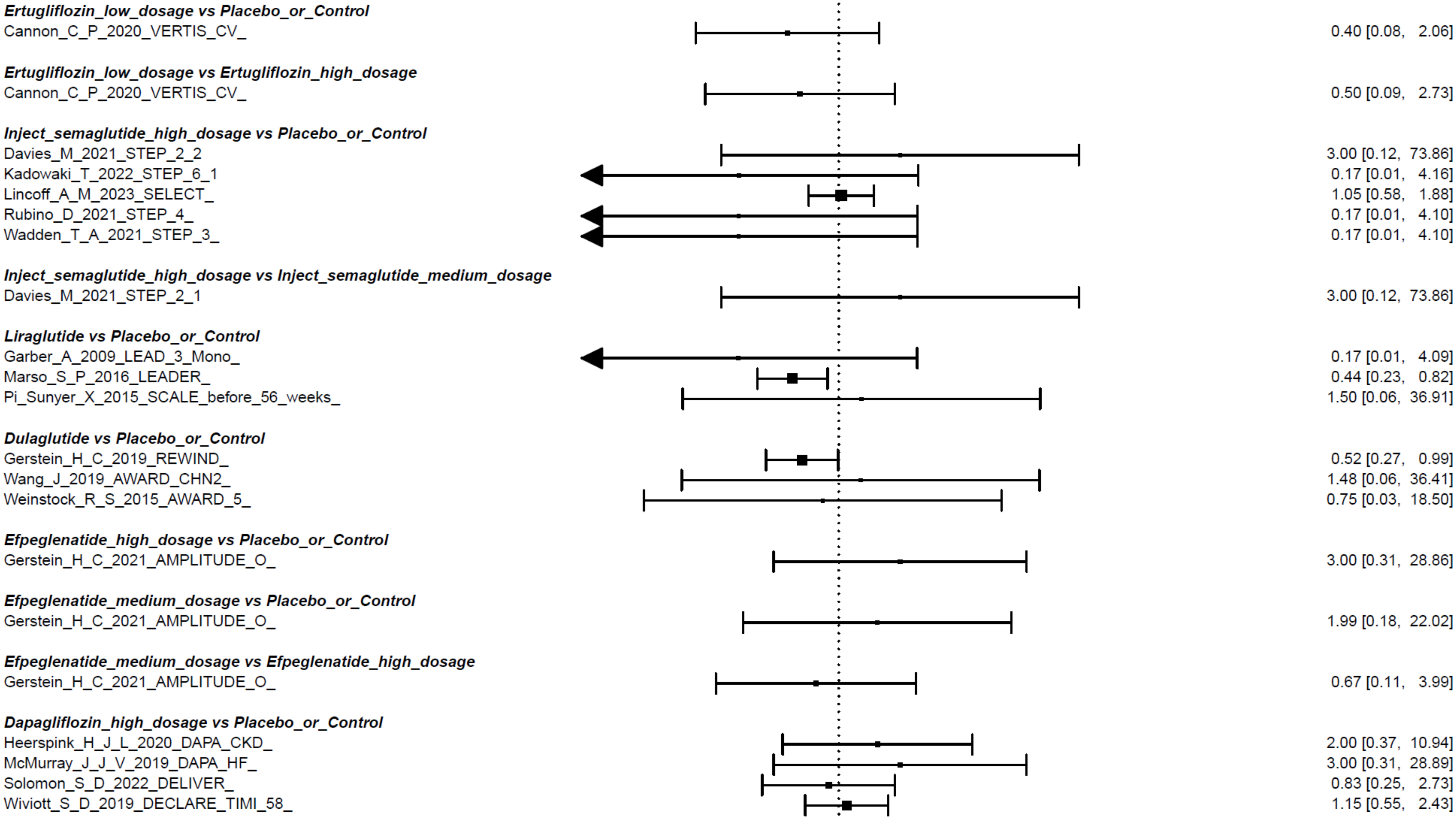
Ertugliflozin_low_dosage vs Placebo_or_Control
Cannon_C_P_2020_VERTIS_CV_
0.40 [0.08, 2.06]
Ertugliflozin_low_dosage vs Ertugliflozin_high_dosage
Cannon_C_P_2020_VERTIS_CV_
0.50 [0.09, 2.73]
Inject_semaglutide_high_dosage vs Placebo_or_Control
Davies_M_2021_STEP_2_2
3.00 [0.12, 73.86]
Kadowaki_T_2022_STEP_6_1
0.17 [0.01, 4.16]
Lincoff_A_M_2023_SELECT_
1.05 [0.58, 1.88]
Rubino_D_2021_STEP_4_
0.17 [0.01, 4.10]
Wadden_T_A_2021_STEP_3_
0.17 [0.01, 4.10]
Inject_semaglutide_high_dosage vs Inject_semaglutide_medium_dosage
Davies_M_2021_STEP_2_1
3.00 [0.12, 73.86]
Liraglutide vs Placebo_or_Control
Garber_A_2009_LEAD_3_Mono_
0.17 [0.01, 4.09]
Marso_S_P_2016_LEADER_
0.44 [0.23, 0.82]
Pi_Sunyer_X_2015_SCALE_before_56_weeks_
1.50 [0.06, 36.91]
Dulaglutide vs Placebo_or_Control
Gerstein_H_C_2019_REWIND_
0.52 [0.27, 0.99]
Wang_J_2019_AWARD_CHN2_
1.48 [0.06, 36.41]
Weinstock_R_S_2015_AWARD_5_
0.75 [0.03, 18.50]
Efpeglenatide_high_dosage vs Placebo_or_Control
Gerstein_H_C_2021_AMPLITUDE_O_
3.00 [0.31, 28.86]
Efpeglenatide_medium_dosage vs Placebo_or_Control
Gerstein_H_C_2021_AMPLITUDE_O_
1.99 [0.18, 22.02]
Efpeglenatide_medium_dosage vs Efpeglenatide_high_dosage
Gerstein_H_C_2021_AMPLITUDE_O_
0.67 [0.11, 3.99]
Dapagliflozin_high_dosage vs Placebo_or_Control
Heerspink_H_J_L_2020_DAPA_CKD_
2.00 [0.37, 10.94]
McMurray_J_J_V_2019_DAPA_HF_
3.00 [0.31, 28.89]
Solomon_S_D_2022_DELIVER_
0.83 [0.25, 2.73]
Wiviott_S_D_2019_DECLARE_TIMI_58_
1.15 [0.55, 2.43]

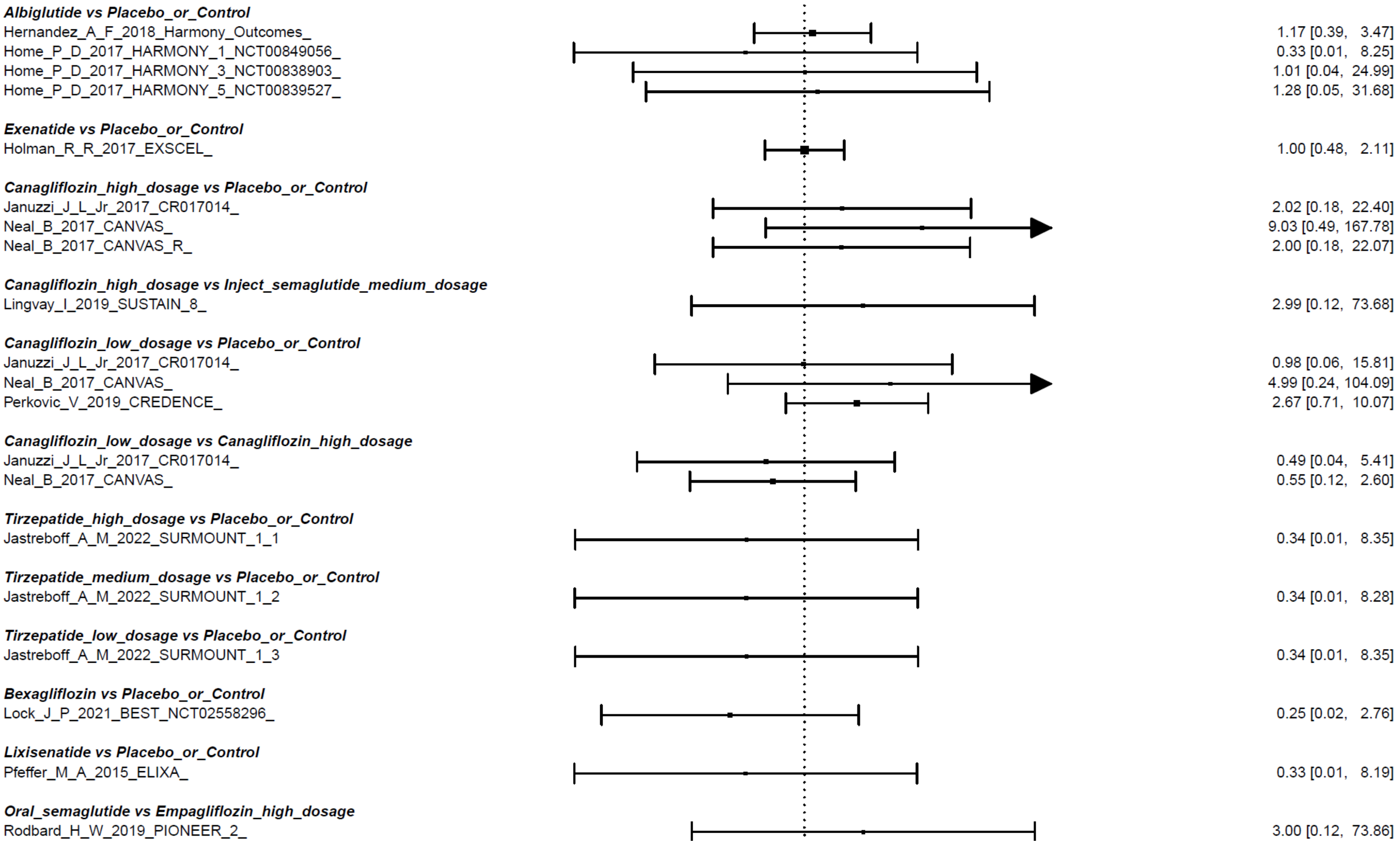
Albiglutide vs Placebo_or_Control
Hernandez_A_F_2018_Harmony_Outcomes_ 1.17 [0.39, 3.47]
Home_P_D_2017_HARMONY_1_NCT00849056_ 0.33 [0.01, 8.25]
Home_P_D_2017_HARMONY_3_NCT00838903_ 1.01 [0.04, 24.99]
Home_P_D_2017_HARMONY_5_NCT00839527_ 1.28 [0.05, 31.68]
Exenatide vs Placebo_or_Control
Holman_R_R_2017_EXSCEL_ 1.00 [0.48, 2.11]
Canagliflozin_high_dosage vs Placebo_or_Control
Januzzi_J_L_Jr_2017_CR017014_ 2.02 [0.18, 22.40]
Neal_B_2017_CANVAS_ 9.03 [0.49, 167.78]
Neal_B_2017_CANVAS_R_ 2.00 [0.18, 22.07]
Canagliflozin_high_dosage vs Inject_semaglutide_medium_dosage
Lingvay_I_2019_SUSTAIN_8_ 2.99 [0.12, 73.68]
Canagliflozin_low_dosage vs Placebo_or_Control
Januzzi_J_L_Jr_2017_CR017014_ 0.98 [0.06, 15.81]
Neal_B_2017_CANVAS_ 4.99 [0.24, 104.09]
Perkovic_V_2019_CREDENCE_ 2.67 [0.71, 10.07]
Canagliflozin_low_dosage vs Canagliflozin_high_dosage
Januzzi_J_L_Jr_2017_CR017014_ 0.49 [0.04, 5.41]
Neal_B_2017_CANVAS_ 0.55 [0.12, 2.60]
Tirzepatide_high_dosage vs Placebo_or_Control
Jastreboff_A_M_2022_SURMOUNT_1_1 0.34 [0.01, 8.35]
Tirzepatide_medium_dosage vs Placebo_or_Control
Jastreboff_A_M_2022_SURMOUNT_1_2 0.34 [0.01, 8.28]
Tirzepatide_low_dosage vs Placebo_or_Control
Jastreboff_A_M_2022_SURMOUNT_1_3 0.34 [0.01, 8.35]
Bexagliflozin vs Placebo_or_Control
Lock_J_P_2021_BEST_NCT02558296_ 0.25 [0.02, 2.76]
Lixisenatide vs Placebo_or_Control
Pfeffer_M_A_2015_ELIXA_ 0.33 [0.01, 8.19]
Oral_semaglutide vs Empagliflozin_high_dosage
Rodbard_H_W_2019_PIONEER_2_ 3.00 [0.12, 73.86]

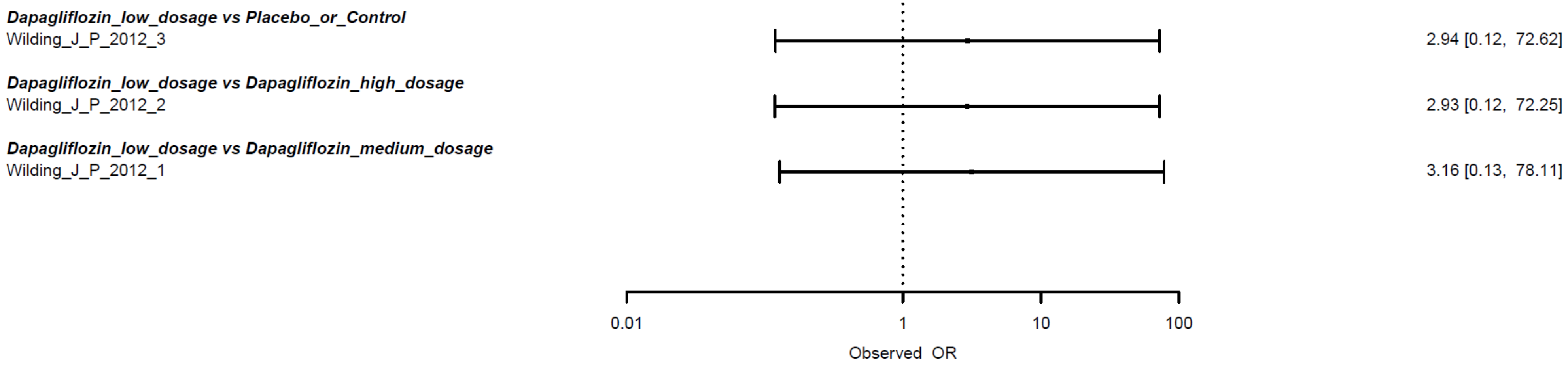
Dapagliflozin_low_dosage vs Placebo_or_Control
Wilding_J_P_2012_3
2.94 [0.12, 72.62]
Dapagliflozin_low_dosage vs Dapagliflozin_high_dosage
Wilding_J_P_2012_2
2.93 [0.12, 72.25]
Dapagliflozin_low_dosage vs Dapagliflozin_medium_dosage
Wilding_J_P_2012_1
3.16 [0.13, 78.11]
0.01
1
10
100
Observed OR

Figure S3C Individual study result of acceptability: drop-out rate

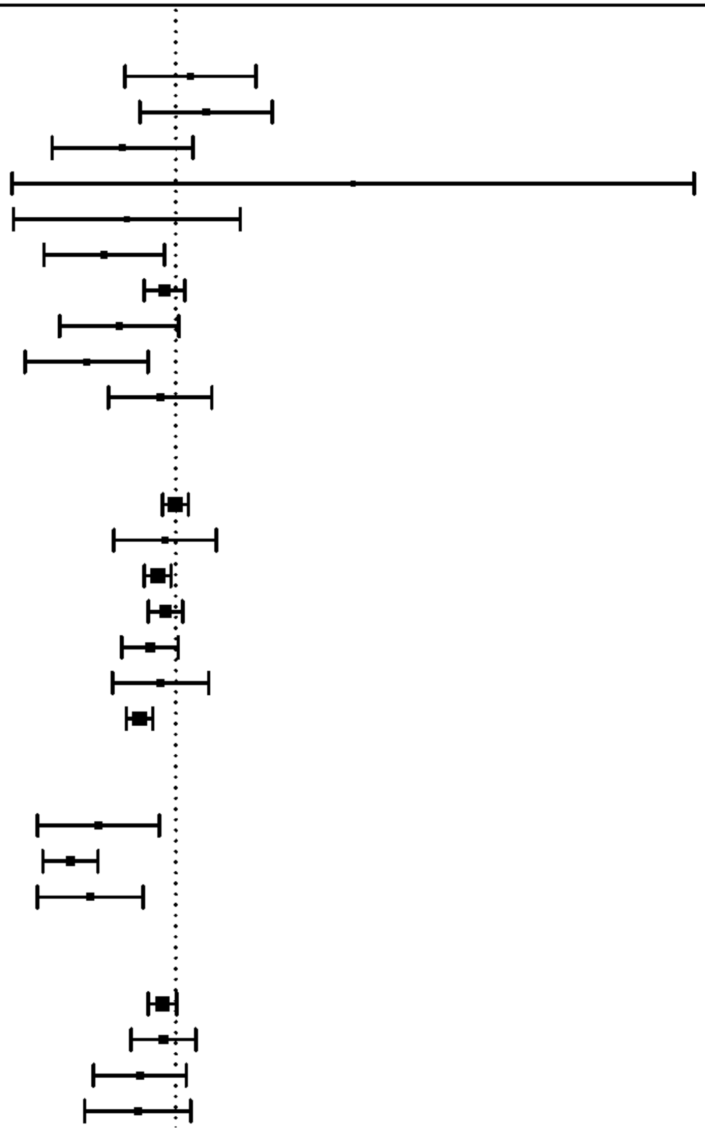

Study	Estimate [95% CI]
Inject_semaglutide vs Placebo_or_Control	
Ahren_B_2017_SUSTAIN_2_	1.13 [0.65, 1.97]
Aroda_V_R_2017_SUSTAIN_4_	1.29 [0.74, 2.26]
Davies_M_2021_STEP_2_	0.64 [0.35, 1.16]
Kadowaki_T_2022_STEP_6_	4.48 [0.25, 80.24]
Kaku_K_2018_SUSTAIN_	0.66 [0.25, 1.73]
Kellerer_M_2022_SUSTAIN_11_	0.55 [0.33, 0.91]
Lincoff_A_M_2023_SELECT_	0.91 [0.77, 1.08]
Marso_S_P_2016_SUSTAIN_6_	0.62 [0.37, 1.03]
Rubino_D_2021_STEP_4_	0.47 [0.28, 0.79]
Wadden_T_A_2021_STEP_3_	0.88 [0.57, 1.36]
Empagliflozin vs Placebo_or_Control	
Anker_S_D_2021_EMPEROR_Preserved_	1.00 [0.90, 1.11]
Barnett_A_H_2014_EMPA_REG_RENAL_	0.91 [0.59, 1.41]
Herrington_W_G_2023_EMPA_KIDNEY_	0.86 [0.77, 0.96]
Packer_M_2020_EMPEROR_Reduced_	0.92 [0.79, 1.06]
Ridderstrale_M_2014_EMPA_REG_H2H_SU_	0.80 [0.63, 1.02]
Voors_A_A_2022_EMPULSE_	0.88 [0.58, 1.32]
Zinman_B_2015_EMPA_REG_OUTCOME_	0.74 [0.66, 0.82]
Tirzepatide vs Placebo_or_Control	
Aronne_L_J_2024_SURMOUNT_4_	0.52 [0.31, 0.87]
Jastreboff_A_M_2022_SURMOUNT_1_	0.41 [0.32, 0.52]
Wadden_T_A_2023_SURMOUNT_3_	0.49 [0.31, 0.76]
Sotagliflozin vs Placebo_or_Control	
Bhatt_D_L_2021_SCORED_	0.89 [0.79, 1.01]
Bhatt_D_L_2021_SOLOIST_WHF_	0.90 [0.69, 1.18]
Buse_J_B_2018_inTandem1_	0.74 [0.50, 1.09]
Cherney_D_Z_I_2023_SOTA_CKD3_	0.73 [0.46, 1.14]

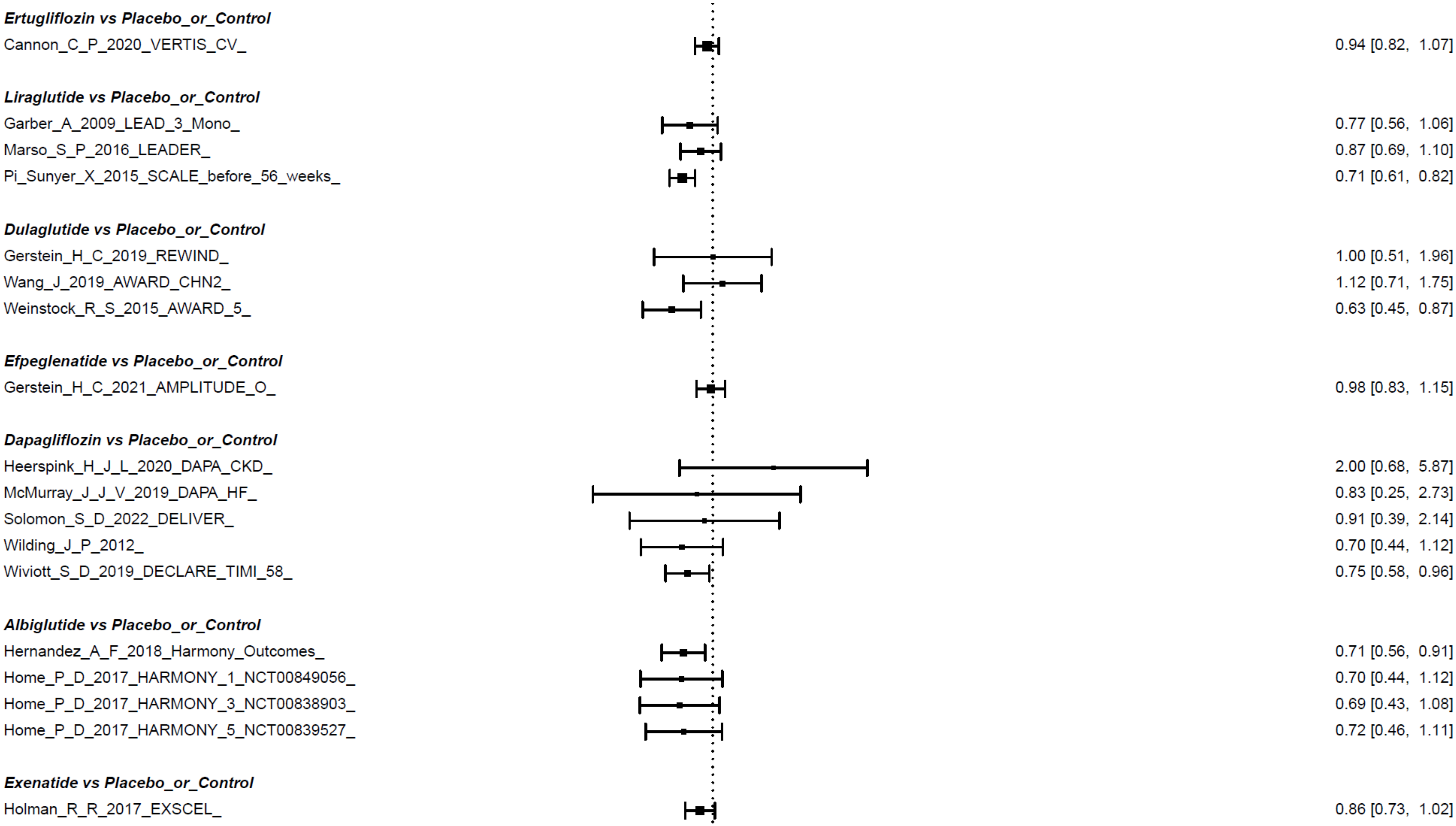

Ertugliflozin vs Placebo_or_Control
Cannon_C_P_2020_VERTIS_CV_ 0.94 [0.82, 1.07]
Liraglutide vs Placebo_or_Control
Garber_A_2009_LEAD_3_Mono_ 0.77 [0.56, 1.06]
Marso_S_P_2016_LEADER_ 0.87 [0.69, 1.10]
Pi_Sunyer_X_2015_SCALE_before_56_weeks_ 0.71 [0.61, 0.82]
Dulaglutide vs Placebo_or_Control
Gerstein_H_C_2019_REWIND_ 1.00 [0.51, 1.96]
Wang_J_2019_AWARD_CHN2_ 1.12 [0.71, 1.75]
Weinstock_R_S_2015_AWARD_5_ 0.63 [0.45, 0.87]
Efpeglenatide vs Placebo_or_Control
Gerstein_H_C_2021_AMPLITUDE_O_ 0.98 [0.83, 1.15]
Dapagliflozin vs Placebo_or_Control
Heerspink_H_J_L_2020_DAPA_CKD_ 2.00 [0.68, 5.87]
McMurray_J_J_V_2019_DAPA_HF_ 0.83 [0.25, 2.73]
Solomon_S_D_2022_DELIVER_ 0.91 [0.39, 2.14]
Wilding_J_P_2012_ 0.70 [0.44, 1.12]
Wiviott_S_D_2019_DECLARE_TIMI_58_ 0.75 [0.58, 0.96]
Albiglutide vs Placebo_or_Control
Hernandez_A_F_2018_Harmony_Outcomes_ 0.71 [0.56, 0.91]
Home_P_D_2017_HARMONY_1_NCT00849056_ 0.70 [0.44, 1.12]
Home_P_D_2017_HARMONY_3_NCT00838903_ 0.69 [0.43, 1.08]
Home_P_D_2017_HARMONY_5_NCT00839527_ 0.72 [0.46, 1.11]
Exenatide vs Placebo_or_Control
Holman_R_R_2017_EXSCEL_ 0.86 [0.73, 1.02]

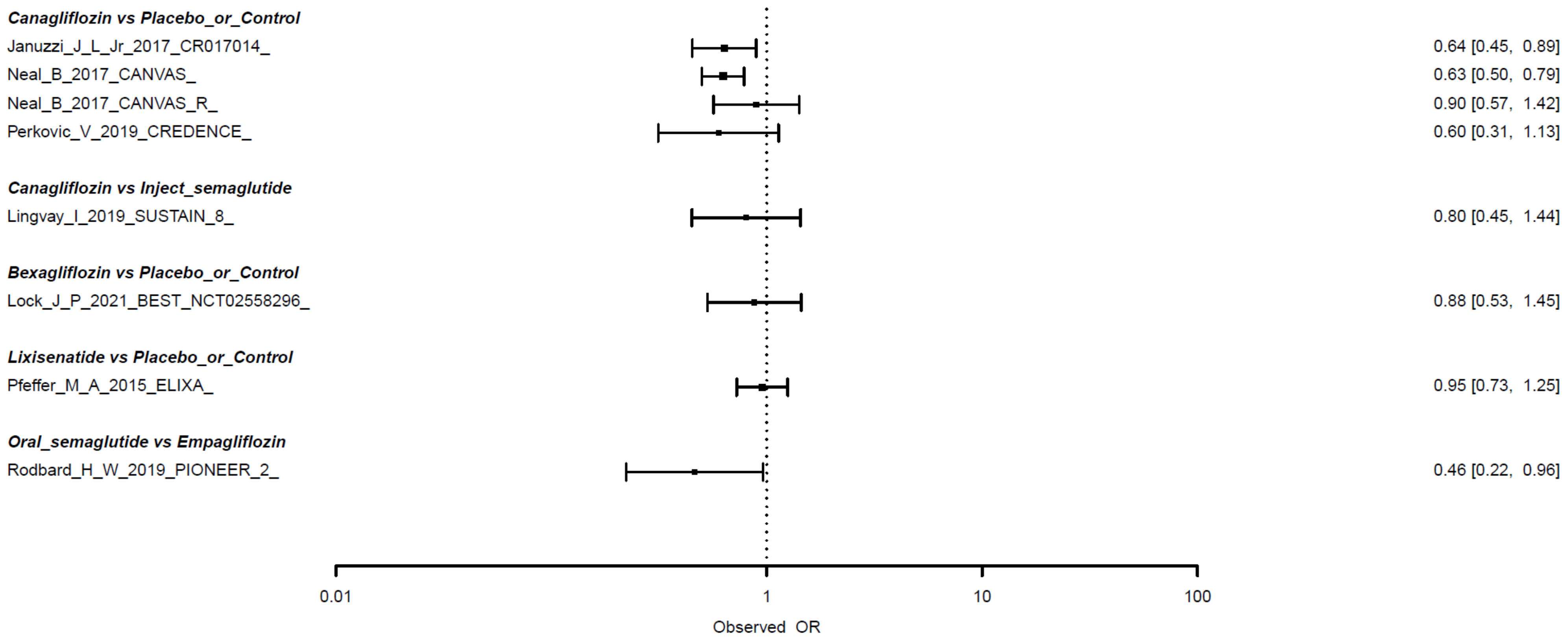

Canagliflozin vs Placebo_or_Control
Januzzi_J_L_Jr_2017_CR017014_ 0.64 [0.45, 0.89]
Neal_B_2017_CANVAS_ 0.63 [0.50, 0.79]
Neal_B_2017_CANVAS_R_ 0.90 [0.57, 1.42]
Perkovic_V_2019_CREDENCE_ 0.60 [0.31, 1.13]
Canagliflozin vs Inject_semaglutide
Lingvay_I_2019_SUSTAIN_8_ 0.80 [0.45, 1.44]
Bexagliflozin vs Placebo_or_Control
Lock_J_P_2021_BEST_NCT02558296_ 0.88 [0.53, 1.45]
Lixisenatide vs Placebo_or_Control
Pfeffer_M_A_2015_ELIXA_ 0.95 [0.73, 1.25]
Oral_semaglutide vs Empagliflozin
Rodbard_H_W_2019_PIONEER_2_ 0.46 [0.22, 0.96]
0.01
1
10
100
Observed OR

Abbreviation for Figure S3A-3C:

95%CIs: 95% confidence intervals; GLP-1 agonist: glucagon-like peptide-1 agonist; NMA: network meta-analysis; OR: odds ratio; RCT: randomized controlled trial; SGLT2 inhibitor: sodium–glucose cotransporter 2 inhibitor

Figure S4A Bayesian-based forest plot of NMA of primary outcome: intestine obstruction events

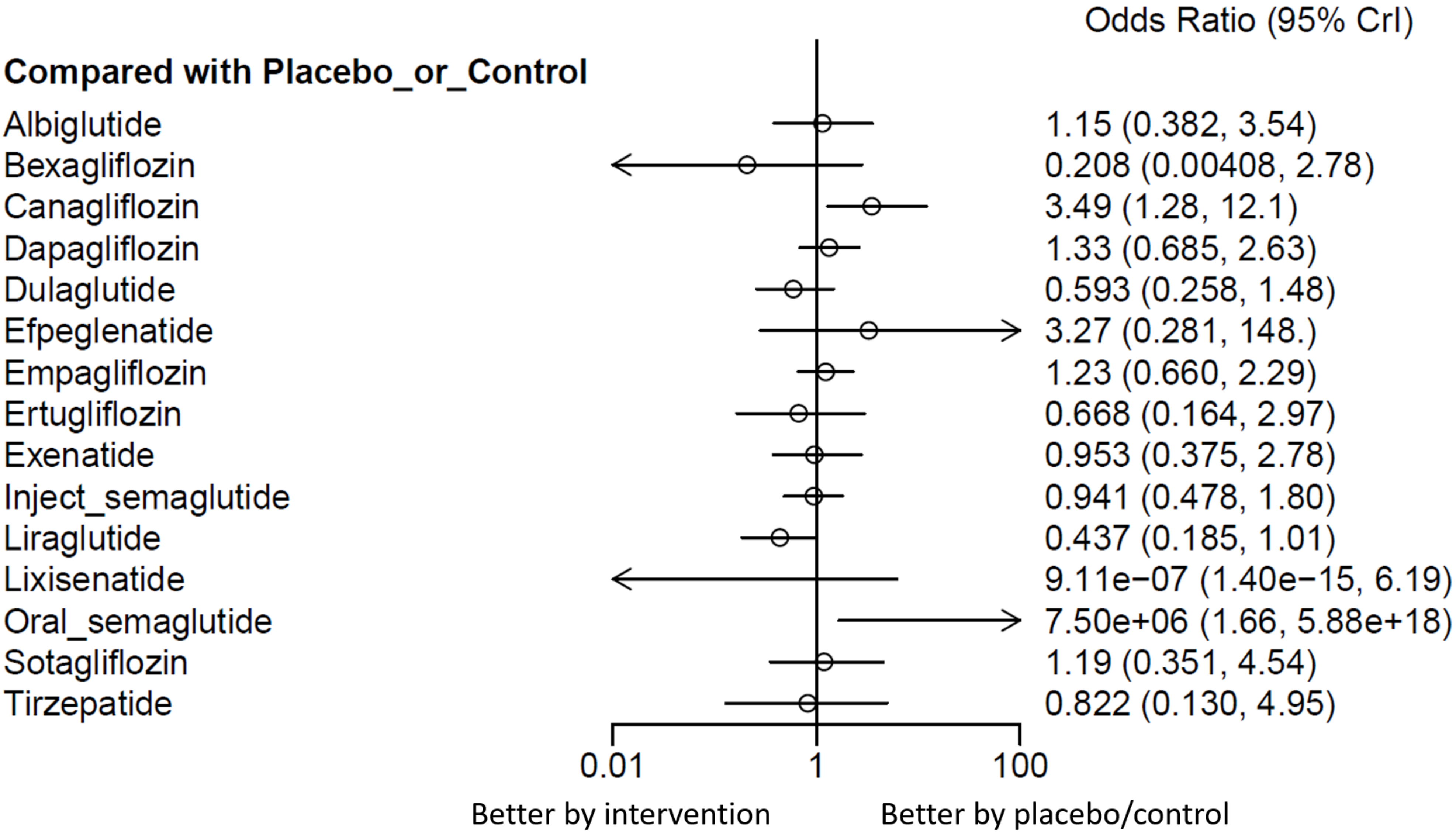

Figure S4B Bayesian-based forest plot of NMA of primary outcome: intestine obstruction events in aspect of various dosage subgroup

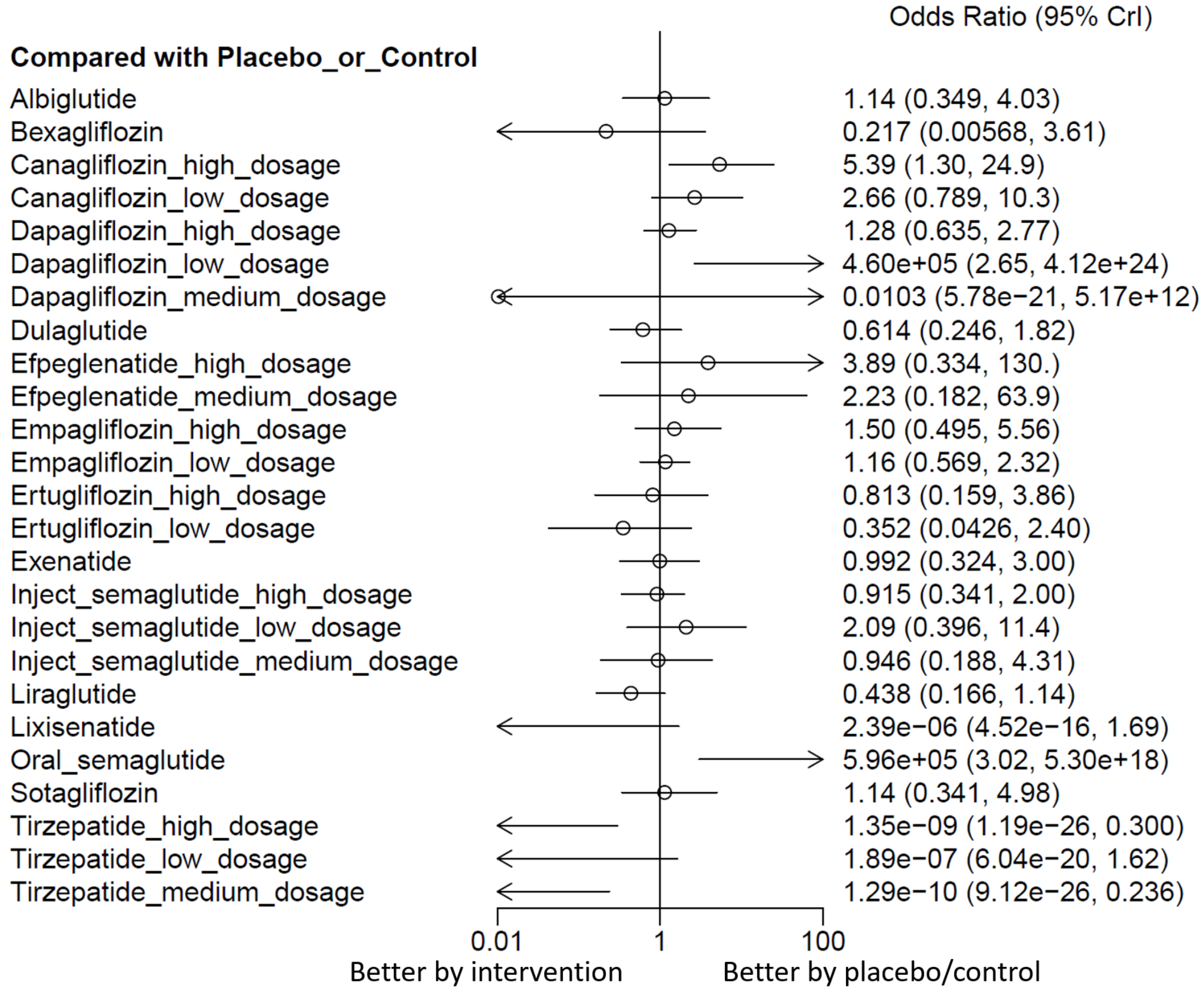

Figure S4C Bayesian-based forest plot of NMA of acceptability: drop-out rate

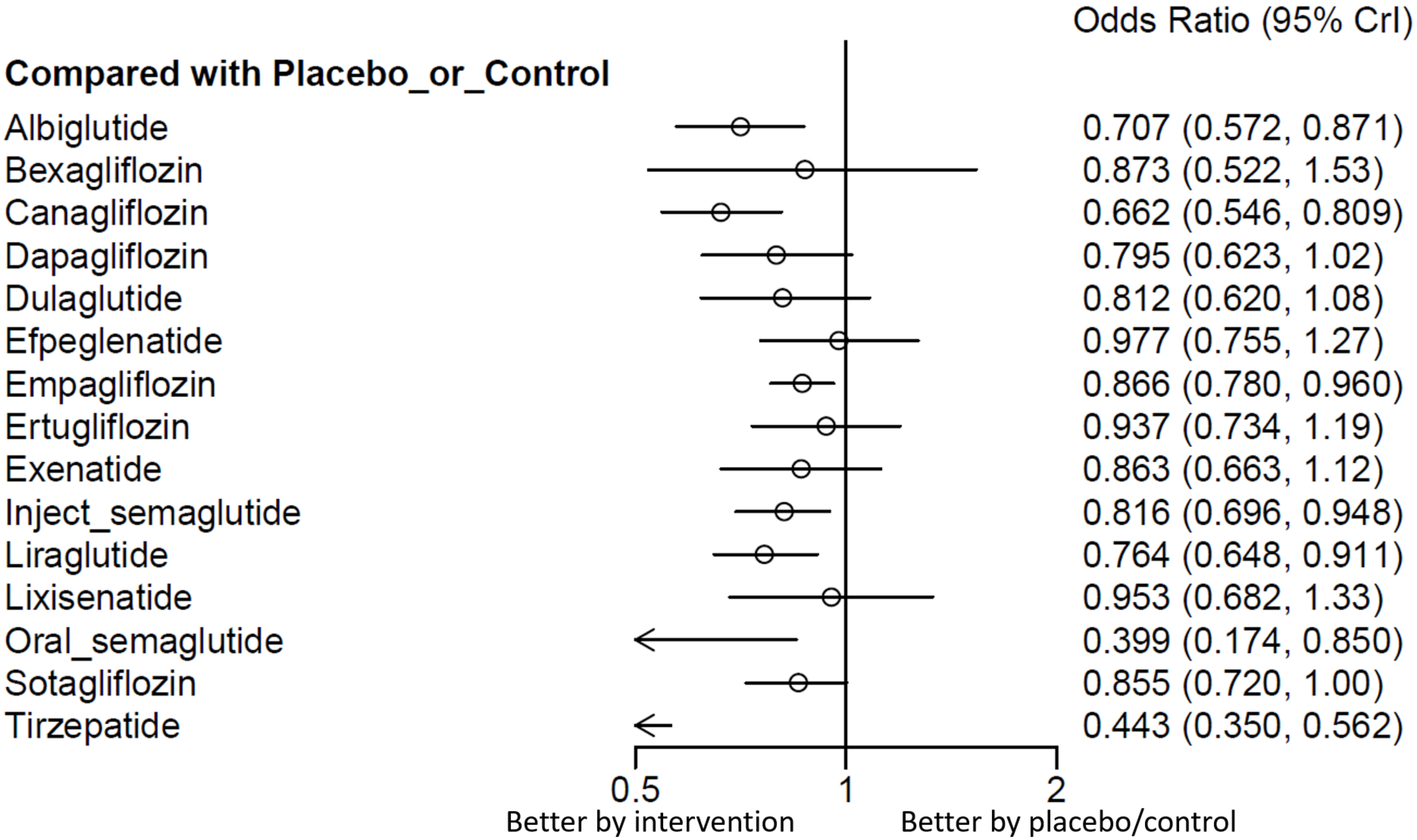

Abbreviation for Figure S4A-4C:

95%CIs: 95% confidence intervals; GLP-1 agonist: glucagon-like peptide-1 agonist; NMA: network meta-analysis; OR: odds ratio; RCT: randomized controlled trial; SGLT2 inhibitor: sodium–glucose cotransporter 2 inhibitor

Figure S5A Bayesian-based Litmus Rank-O-Gram rank plot of primary outcome: intestine obstruction events

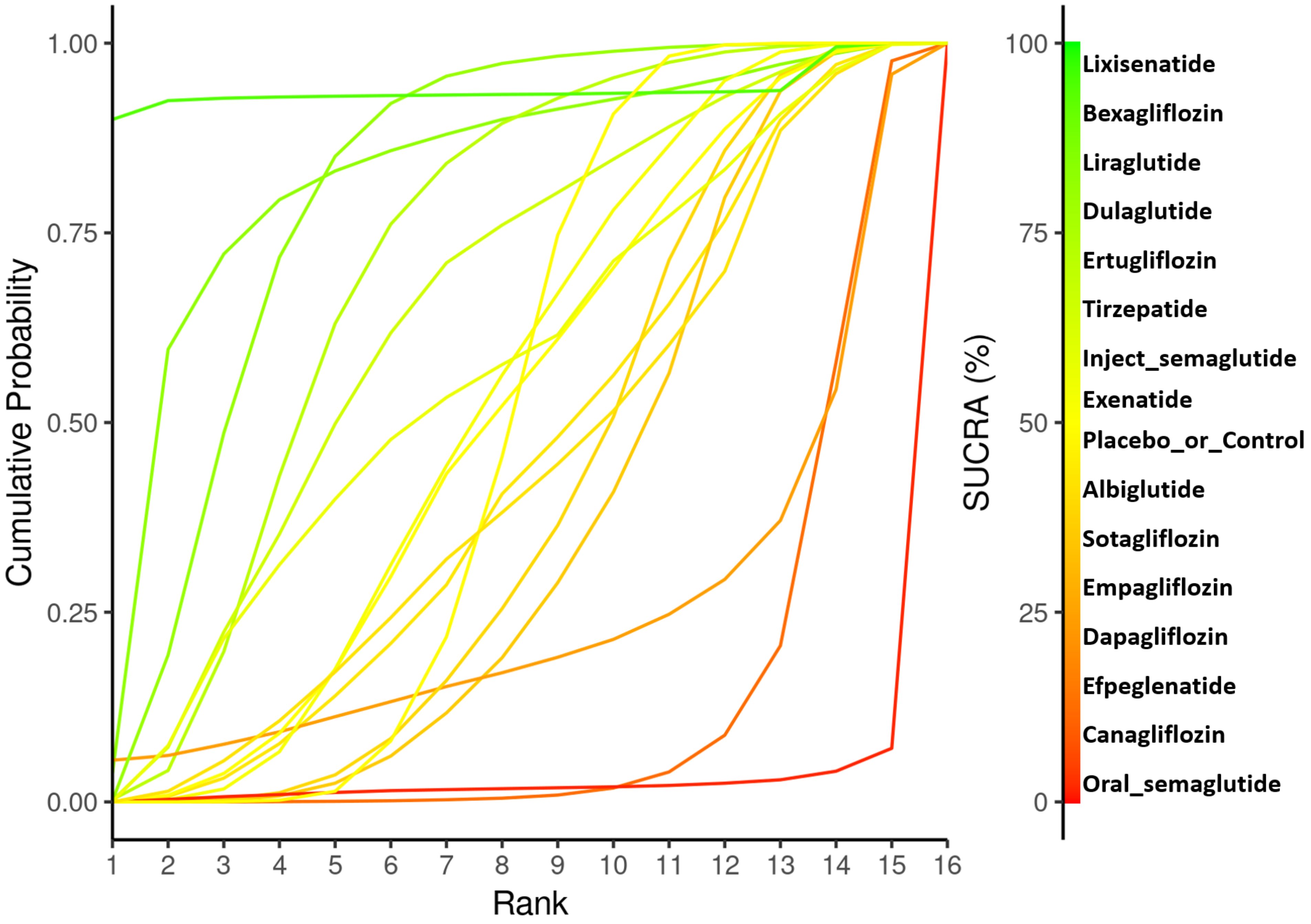

Figure S5B Bayesian-based radial surface under the cumulative ranking of primary outcome: intestine obstruction events

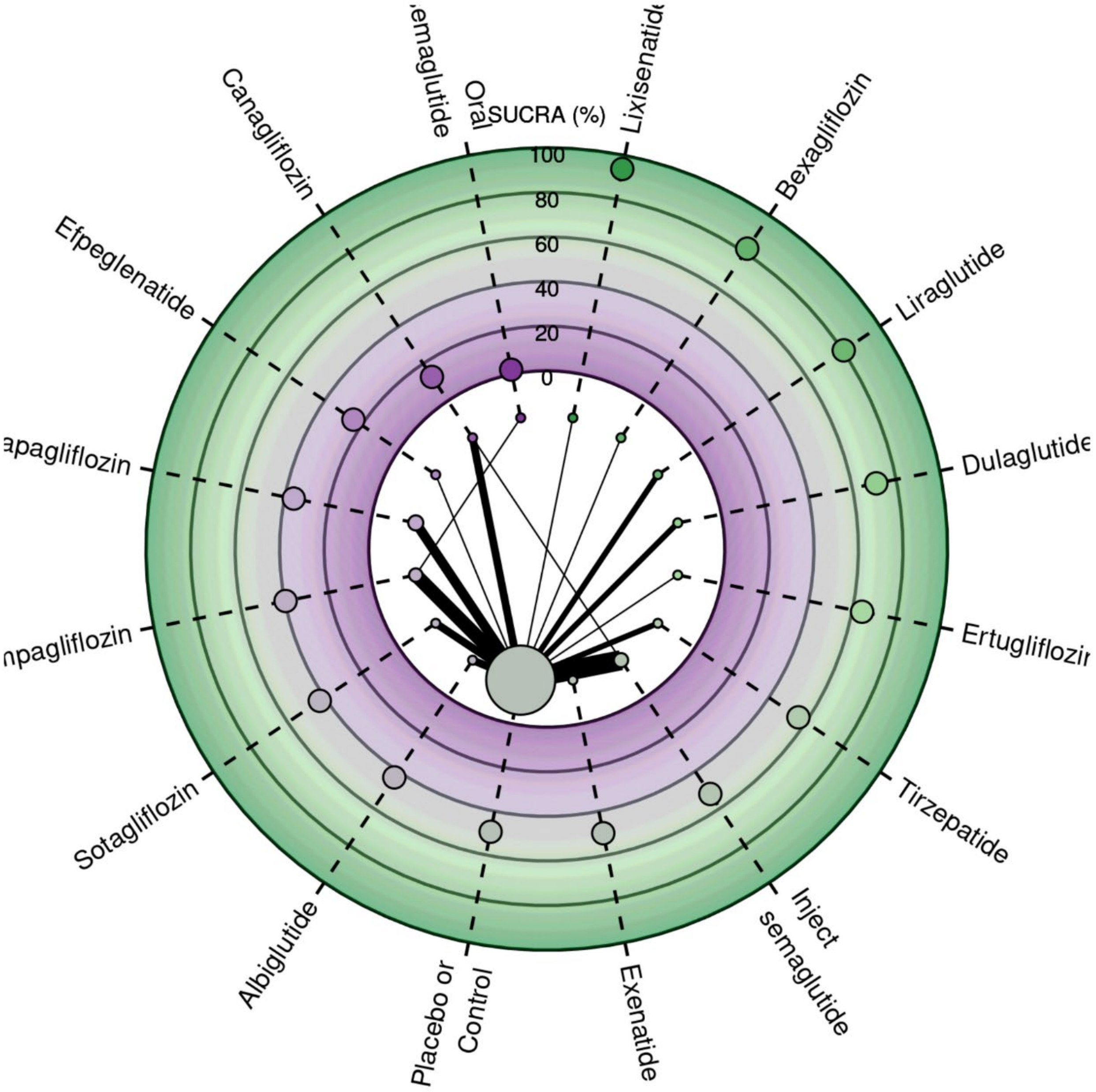

Figure S5C Bayesian-based Litmus Rank-O-Gram rank plot of primary outcome: intestine obstruction events in aspect of various dosage subgroup

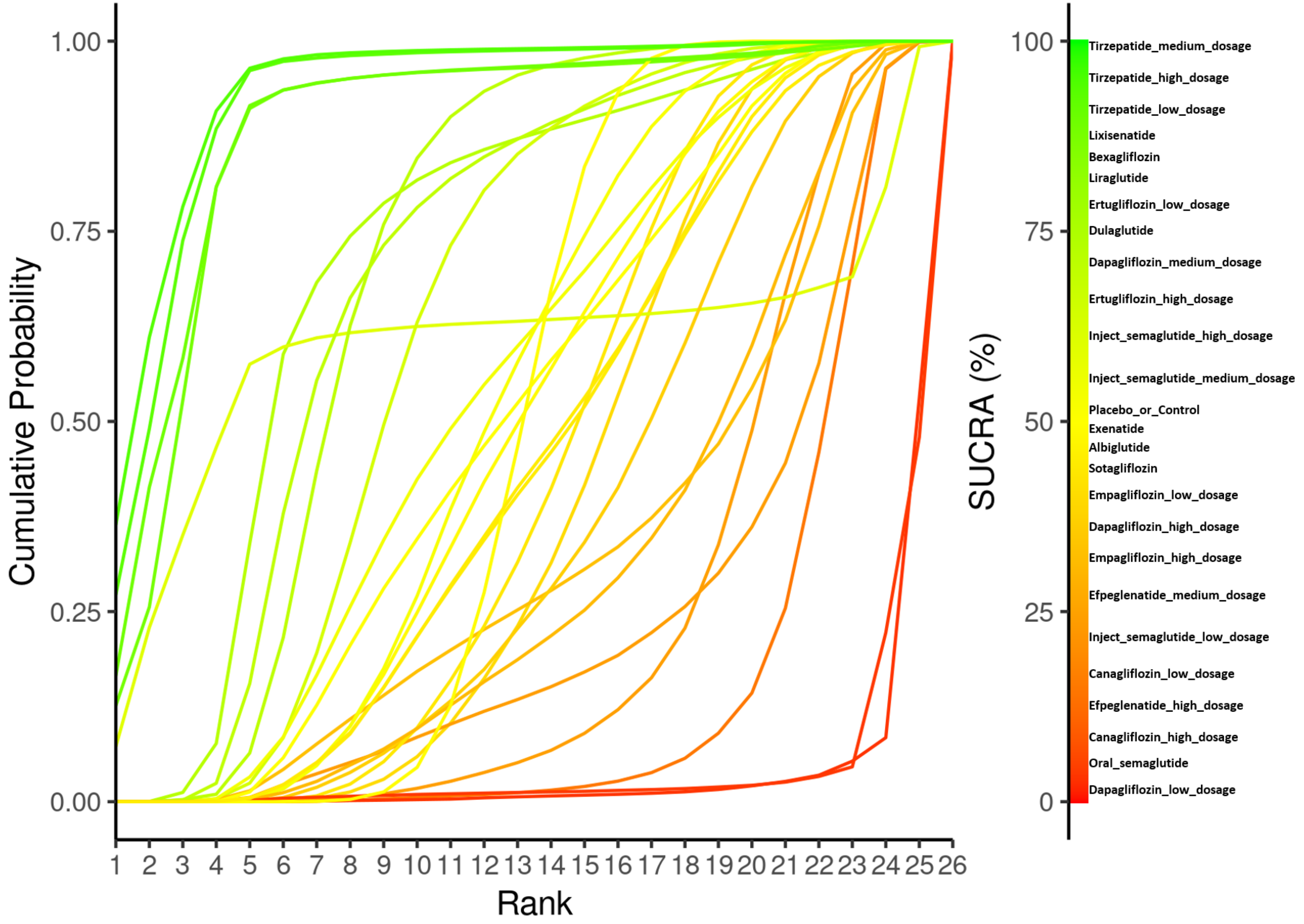

Figure S5D Bayesian-based radial surface under the cumulative ranking of primary outcome: intestine obstruction events in aspect of various dosage subgroup

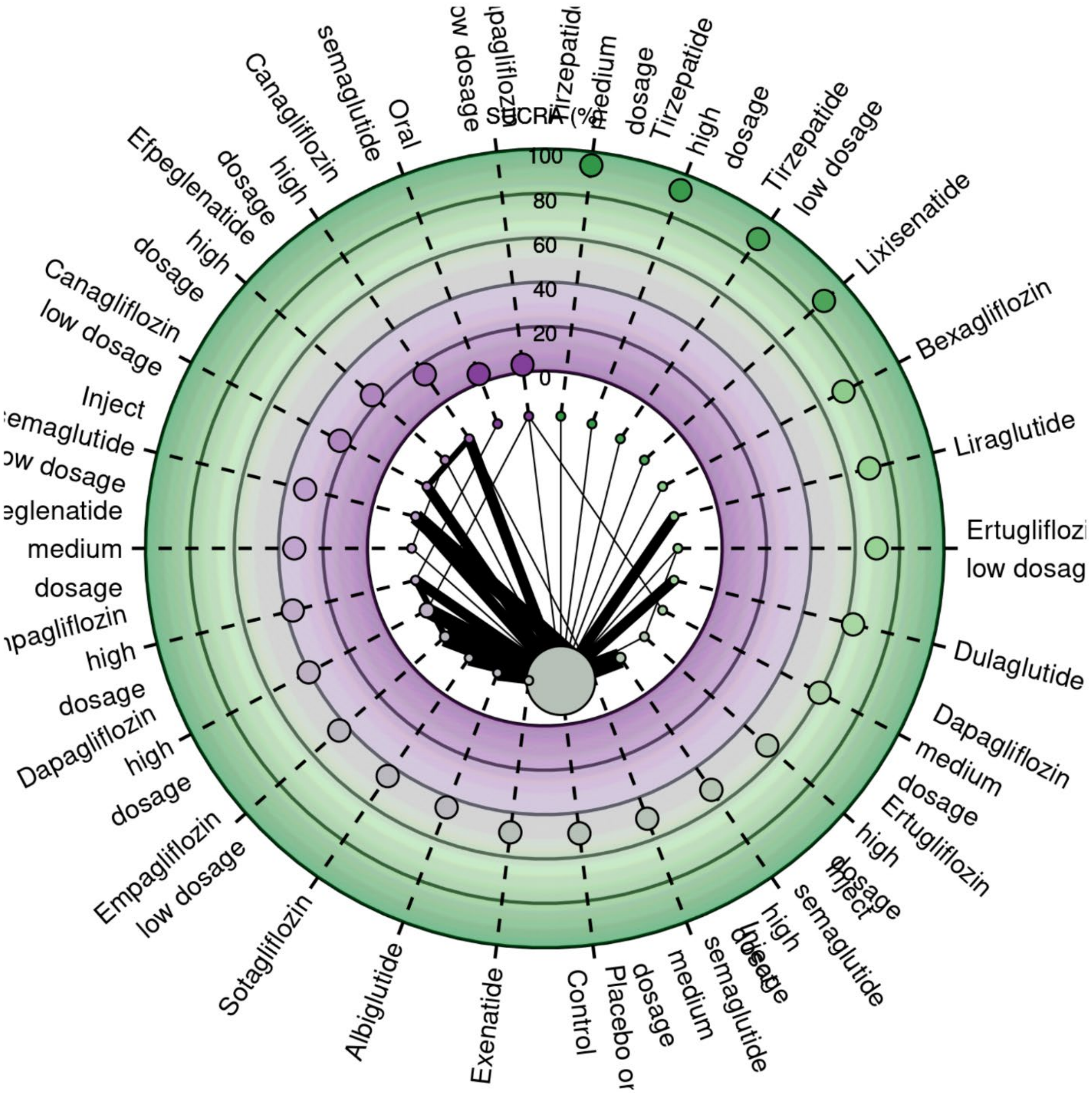

Figure S5E Bayesian-based Litmus Rank-O-Gram rank plot of acceptability: drop-out rate

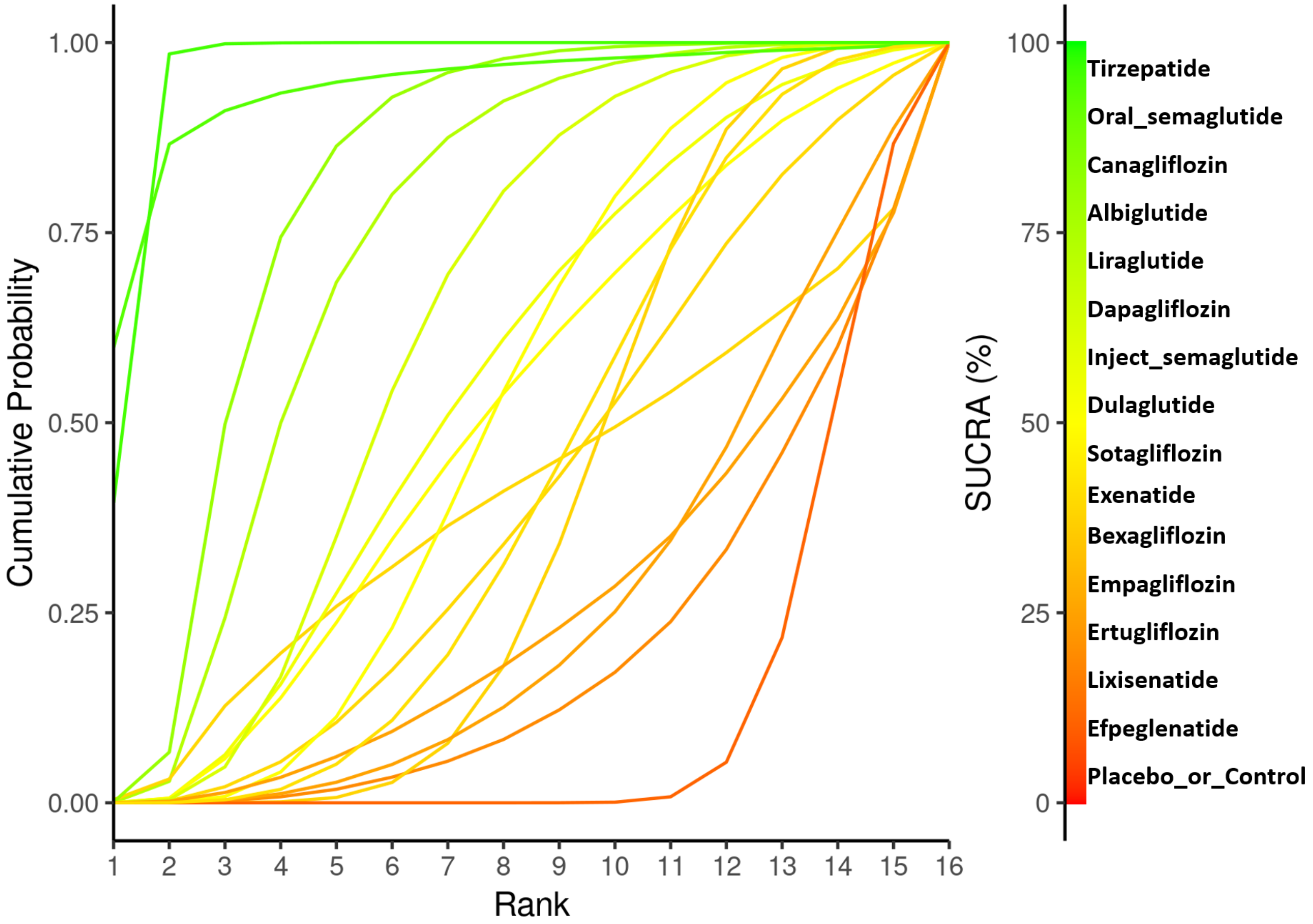

Figure S5F Bayesian-based radial surface under the cumulative ranking of acceptability: drop-out rate

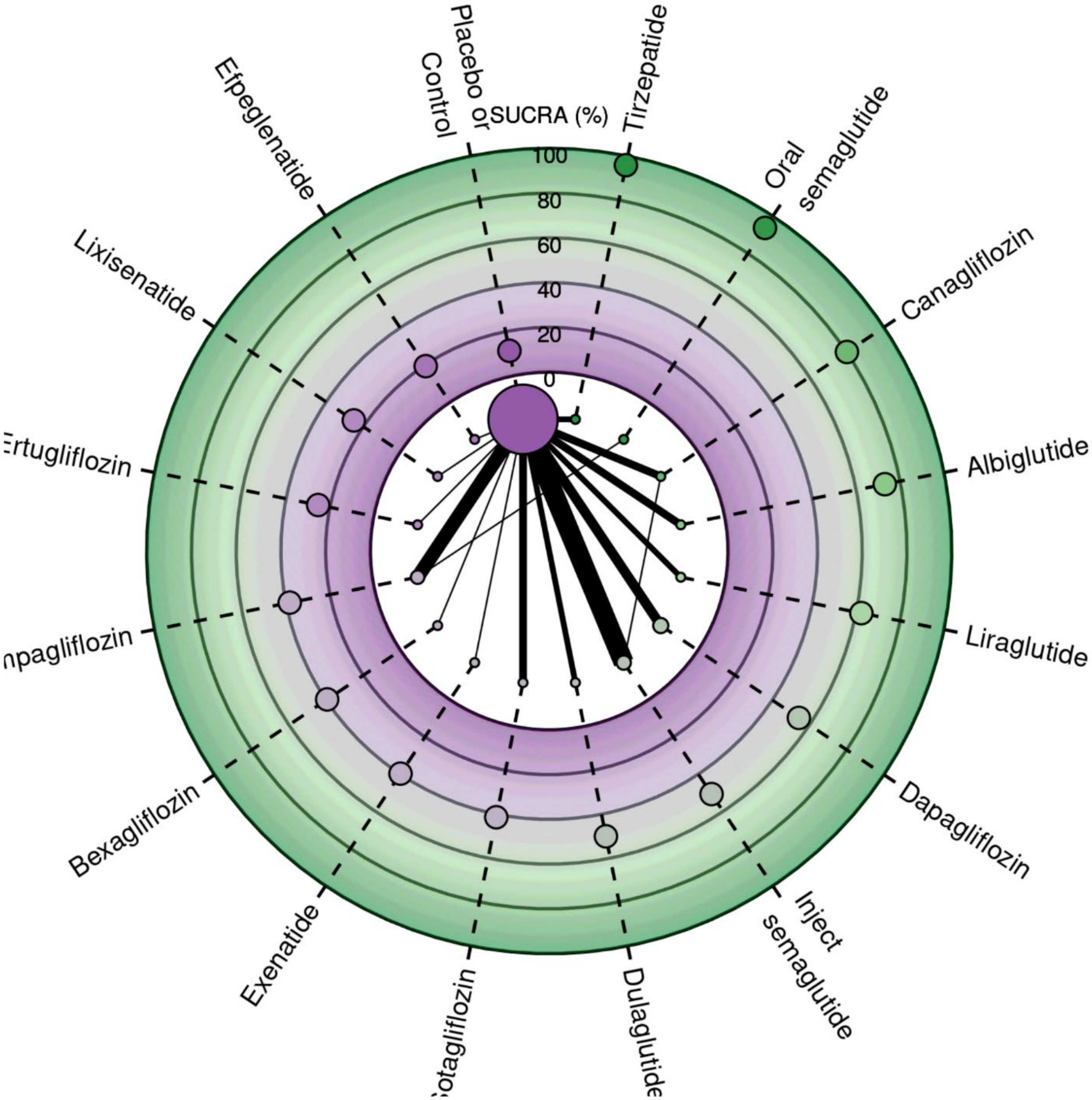

Abbreviation for Figure S5A-5F:

95%CIs: 95% confidence intervals; GLP-1 agonist: glucagon-like peptide-1 agonist; NMA: network meta-analysis; OR: odds ratio; RCT: randomized controlled trial; SGLT2 inhibitor: sodium–glucose cotransporter 2 inhibitor

Figure S6A Bayesian-based residual deviance NMA/UME model of primary outcome: intestine obstruction events

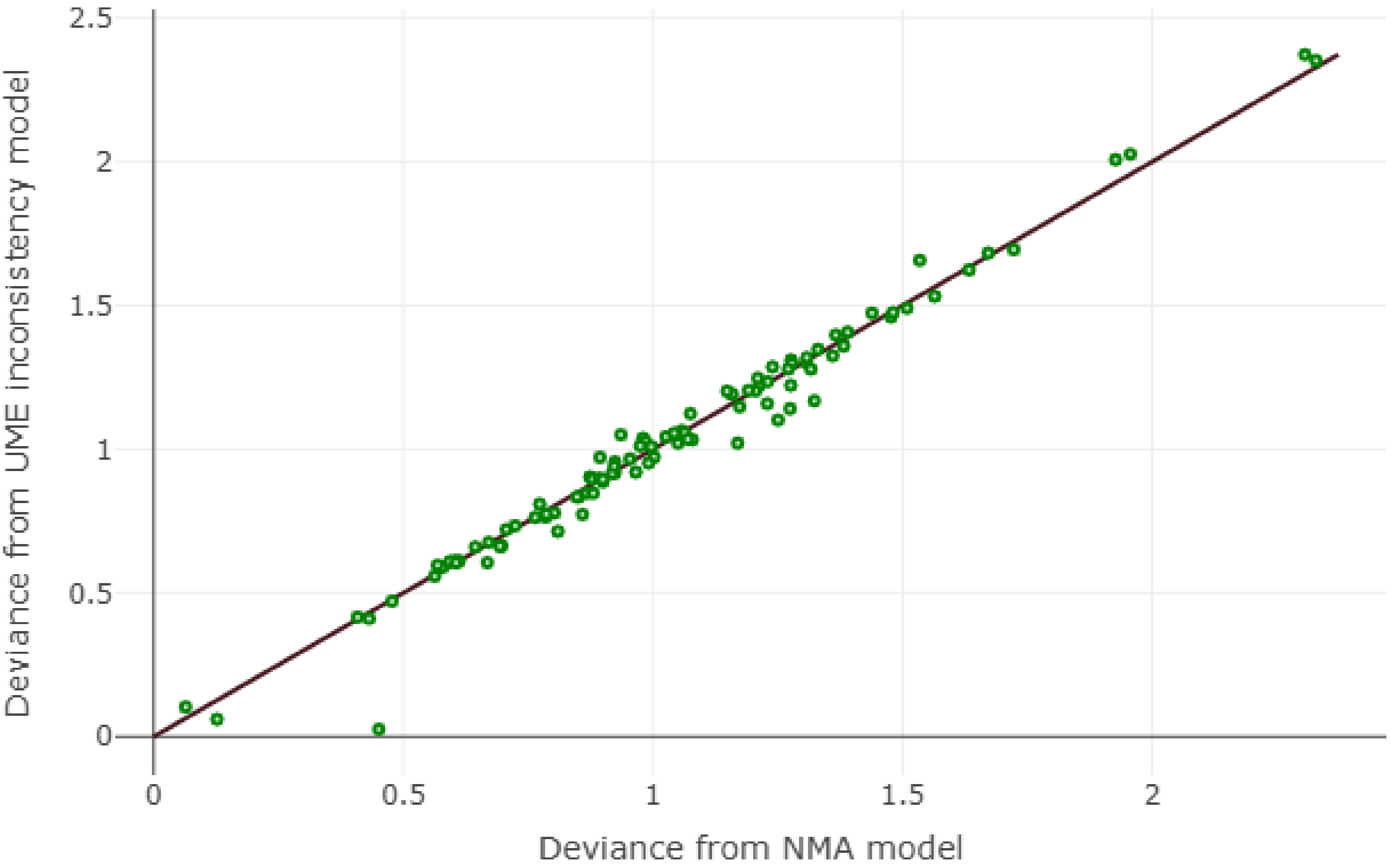

Figure S6B Bayesian-based per-arm residual deviance of primary outcome: intestine obstruction events

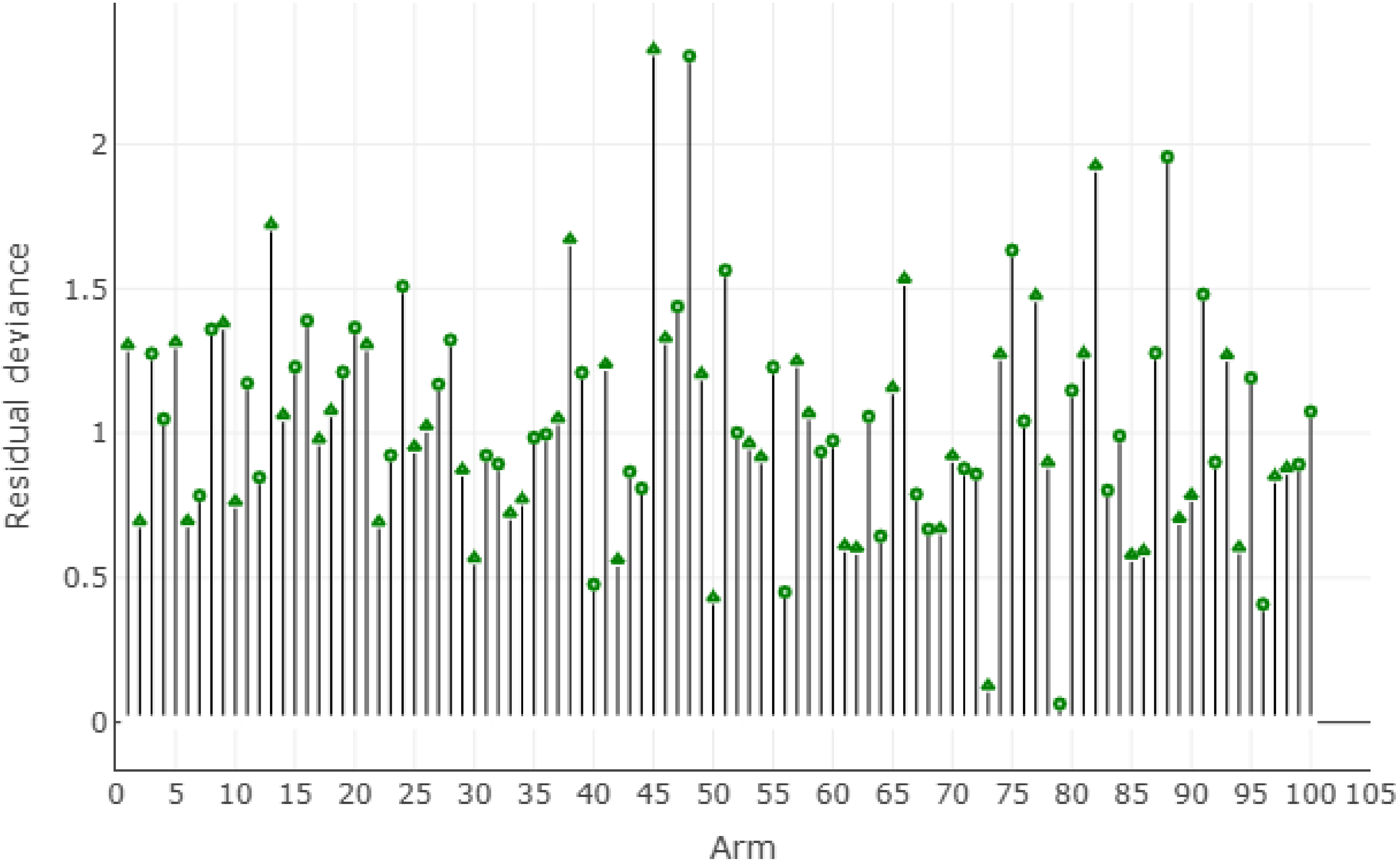

Figure S6C Bayesian-based leverage plot of primary outcome: intestine obstruction events

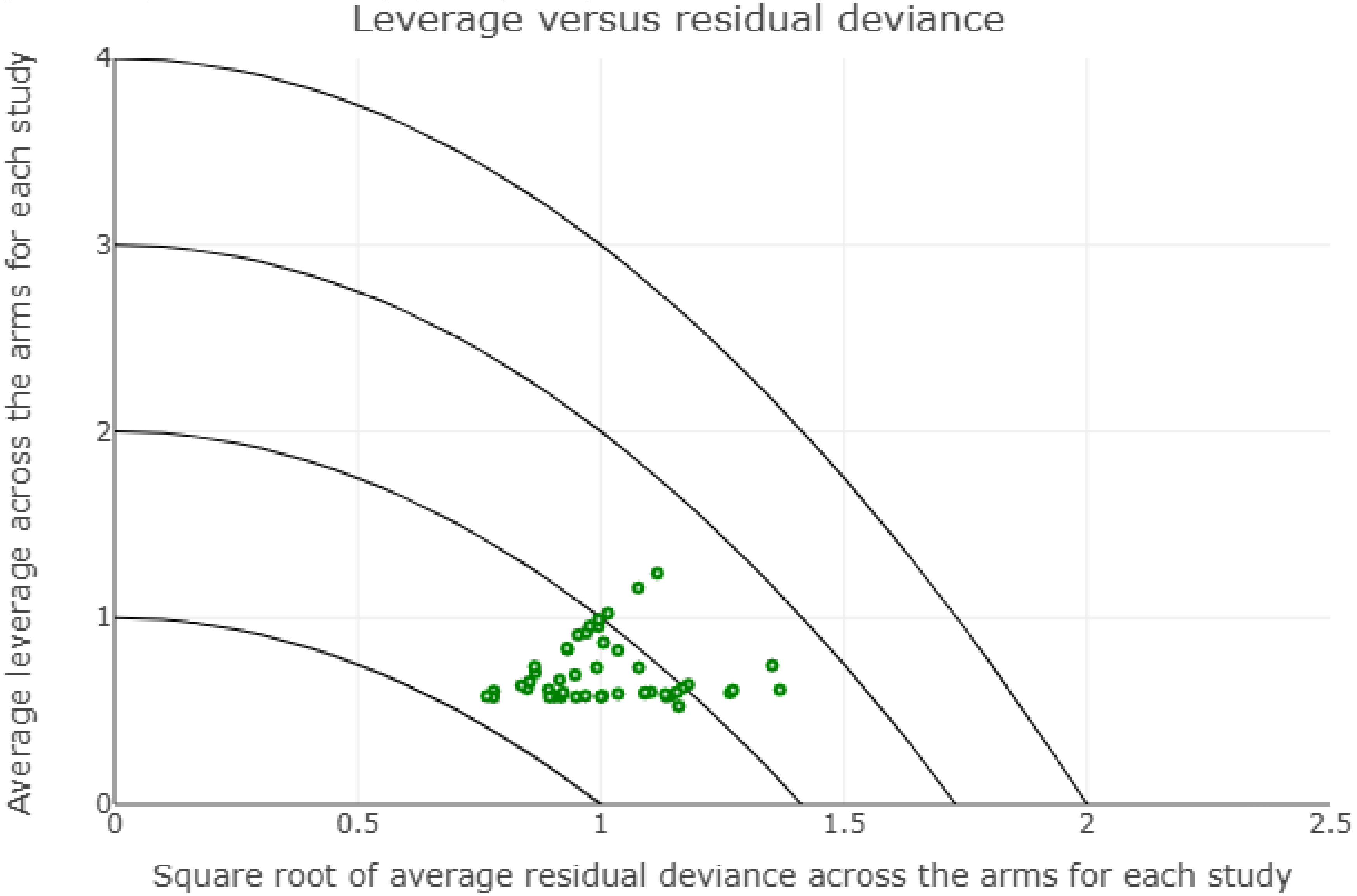

Figure S6D Bayesian-based residual deviance NMA/UME model of primary outcome: intestine obstruction events in aspect of various dosage subgroup

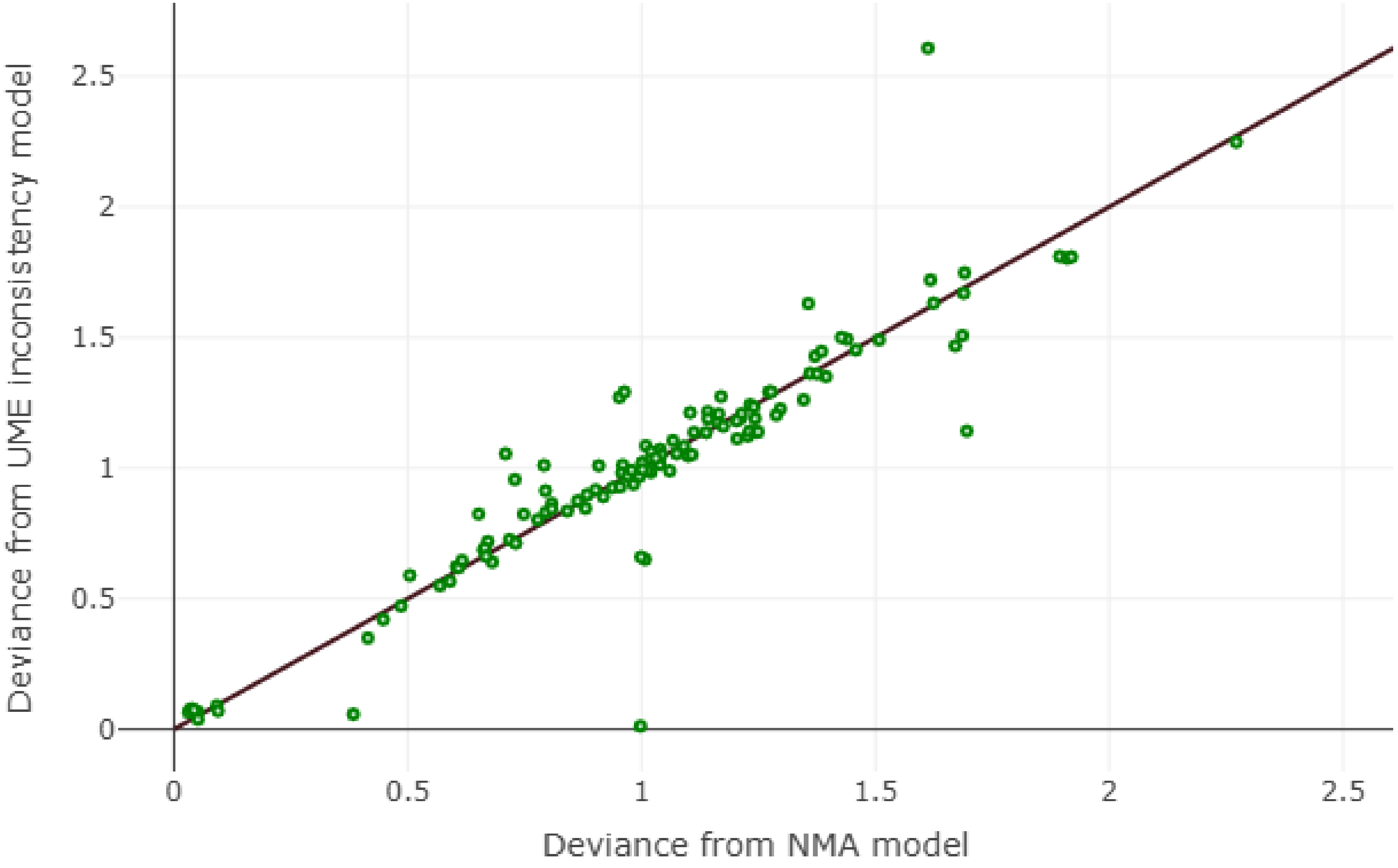

Figure S6E Bayesian-based per-arm residual deviance of primary outcome: intestine obstruction events in aspect of various dosage subgroup

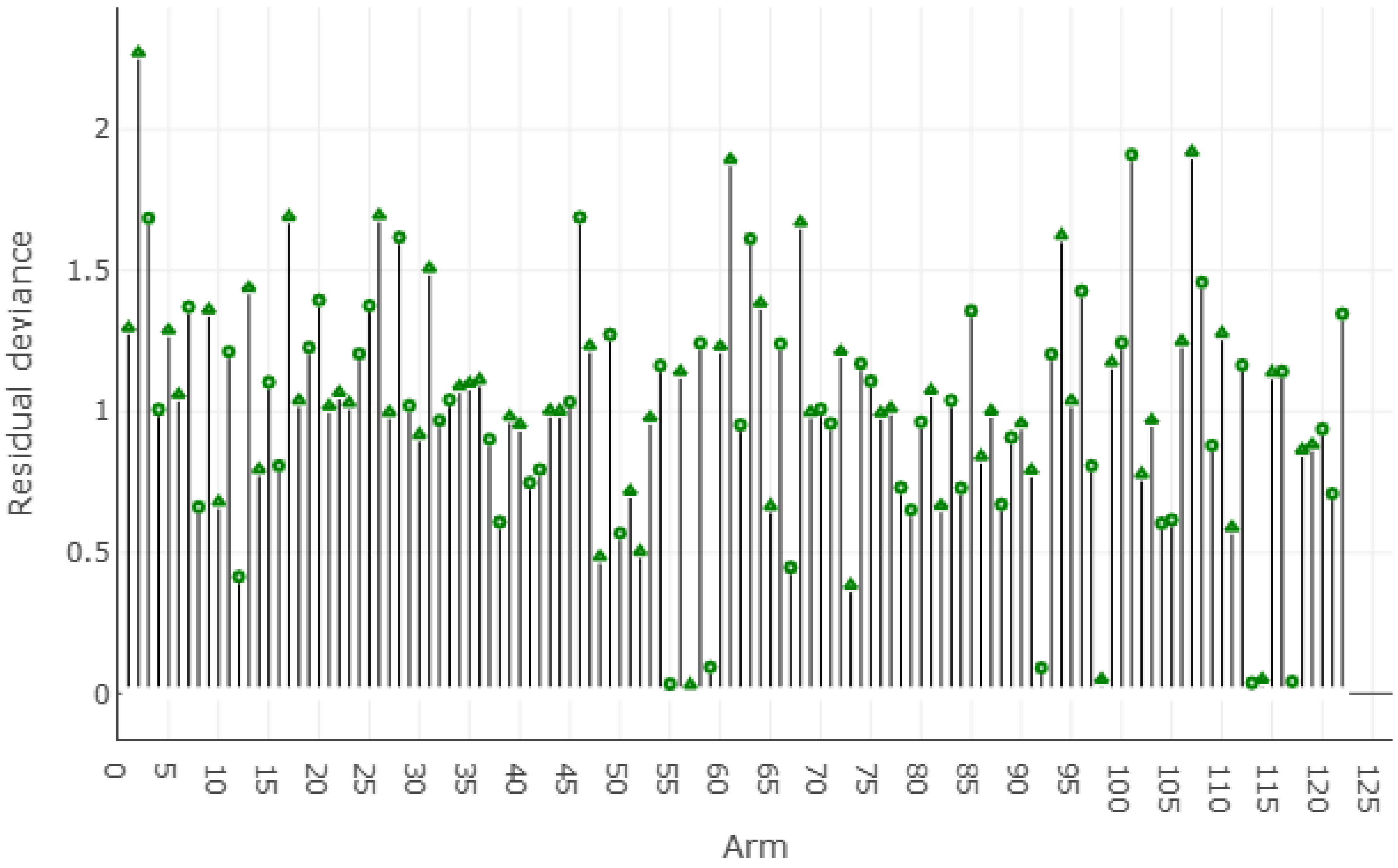

Figure S6F Bayesian-based leverage plot of primary outcome: intestine obstruction events in aspect of various dosage subgroup

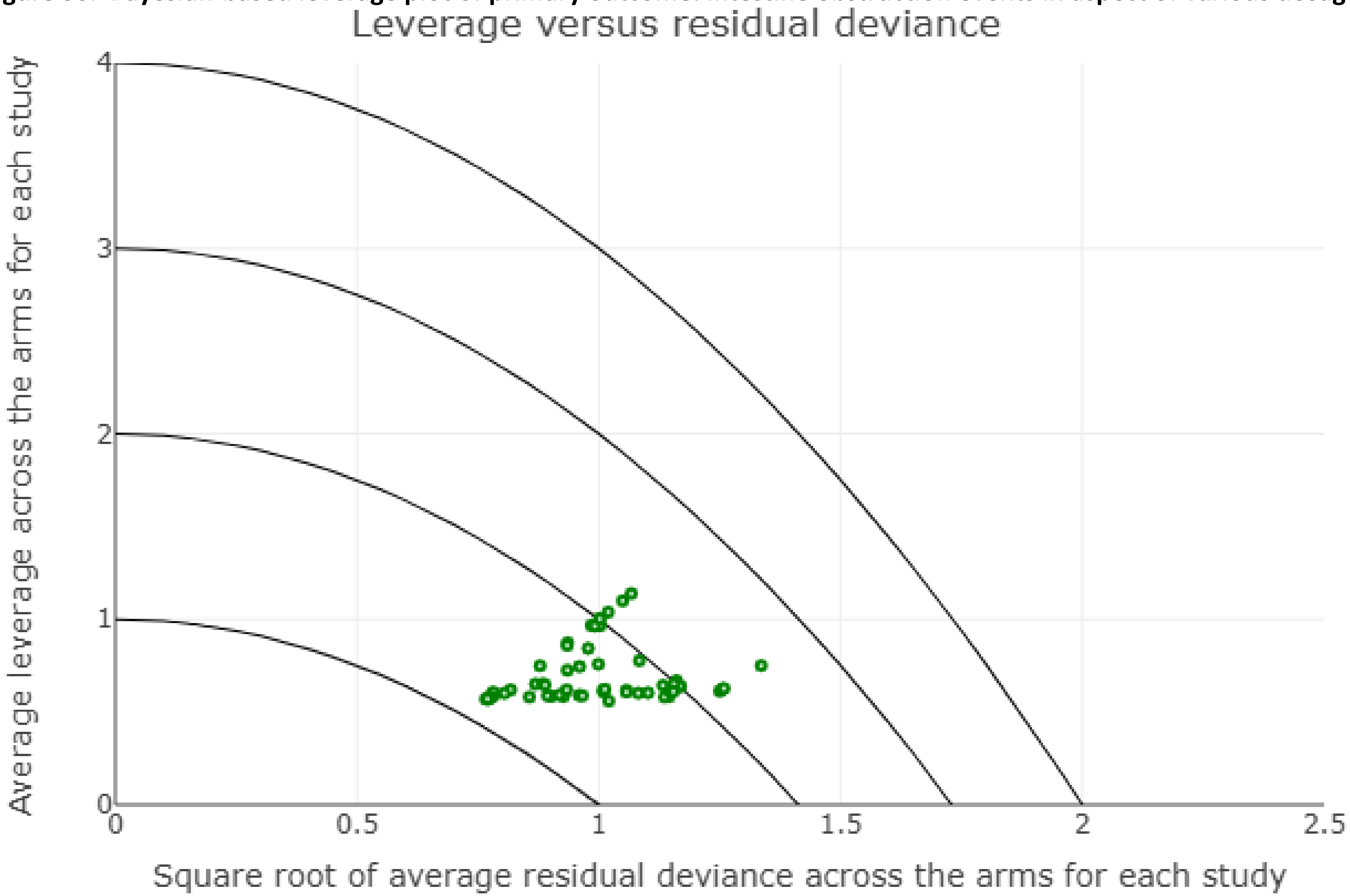

Figure S6G Bayesian-based residual deviance NMA/UME model of acceptability: drop-out rate

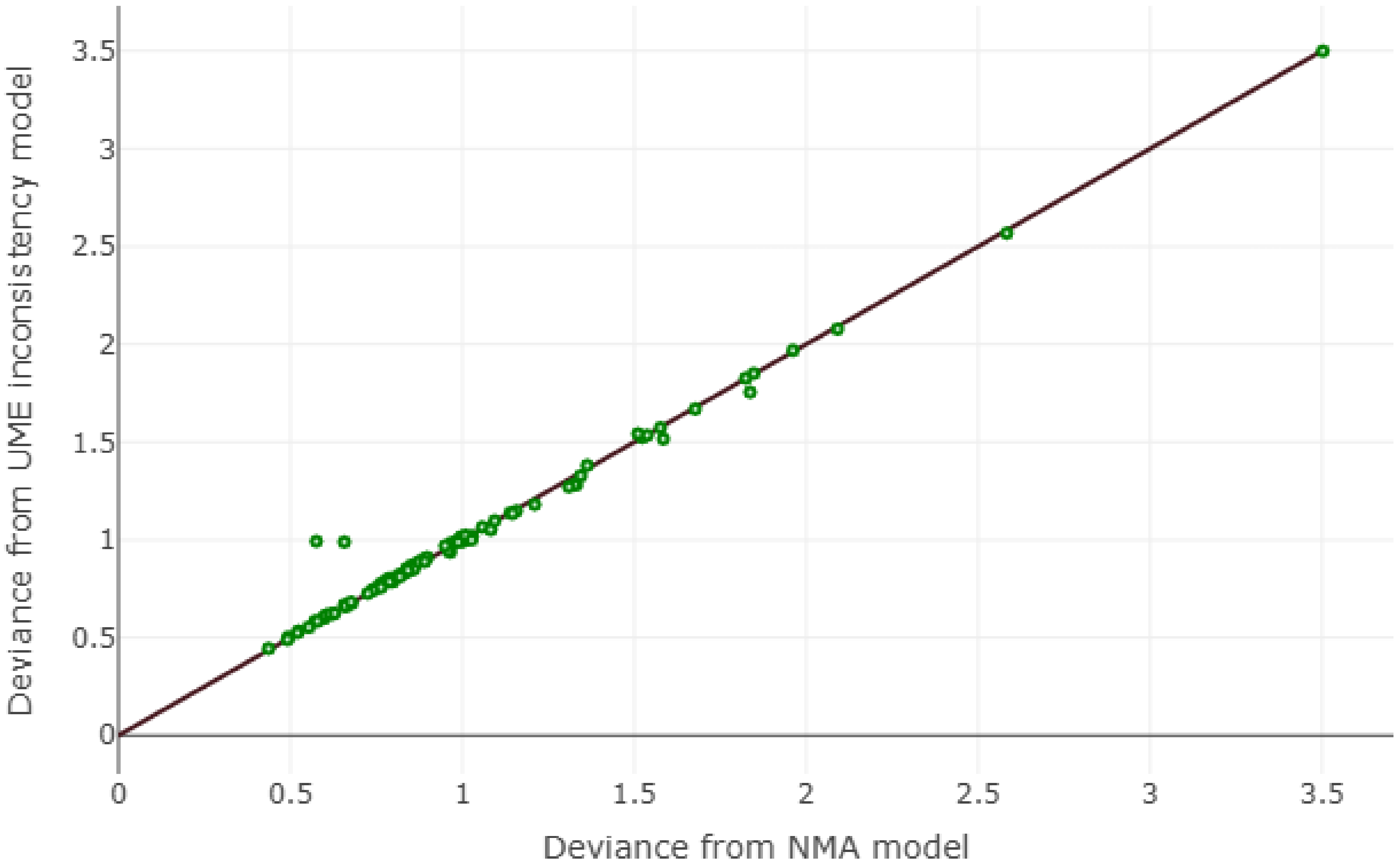

Figure S6H Bayesian-based per-arm residual deviance of acceptability: drop-out rate

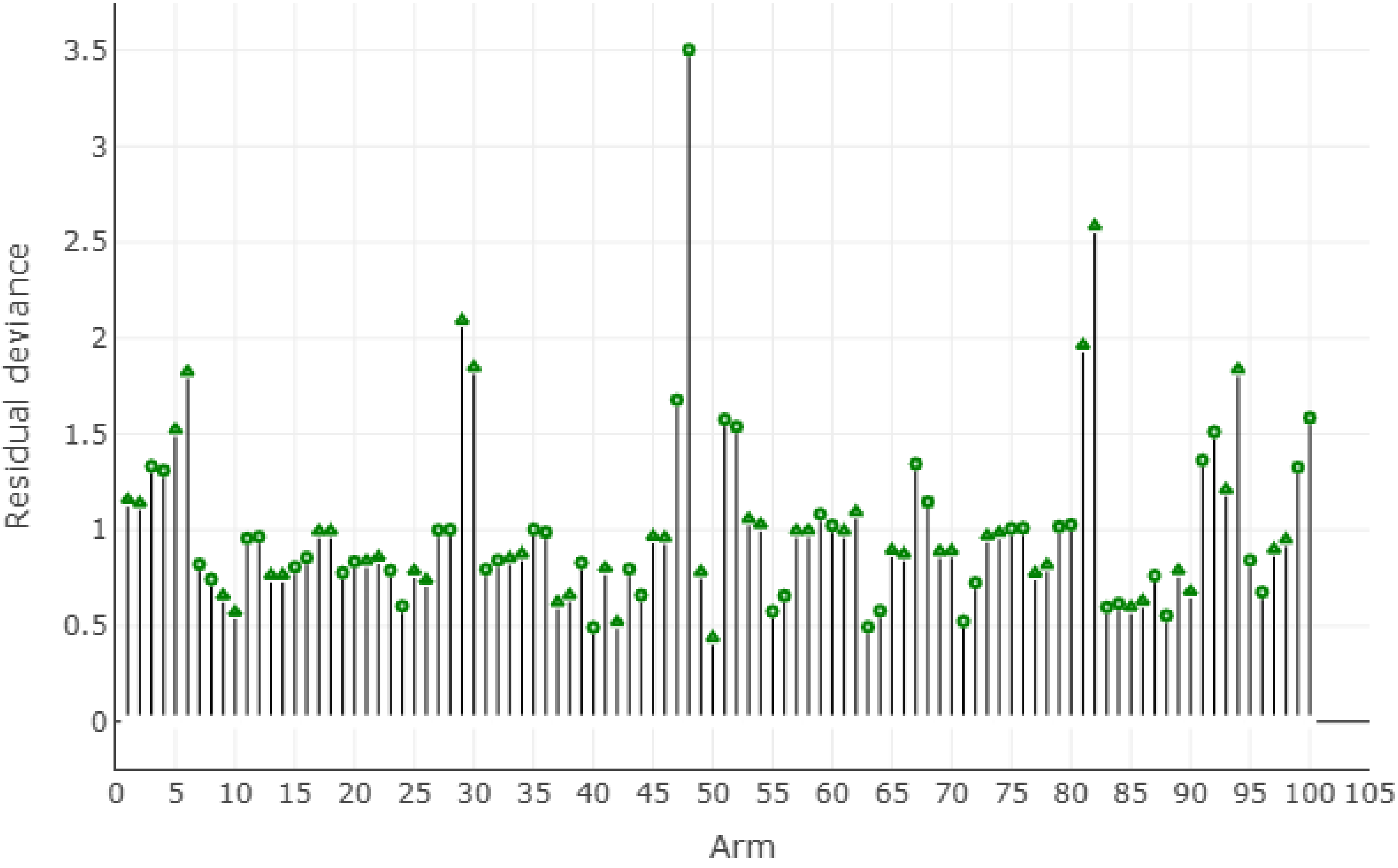

Figure S6I Bayesian-based leverage plot of acceptability: drop-out rate

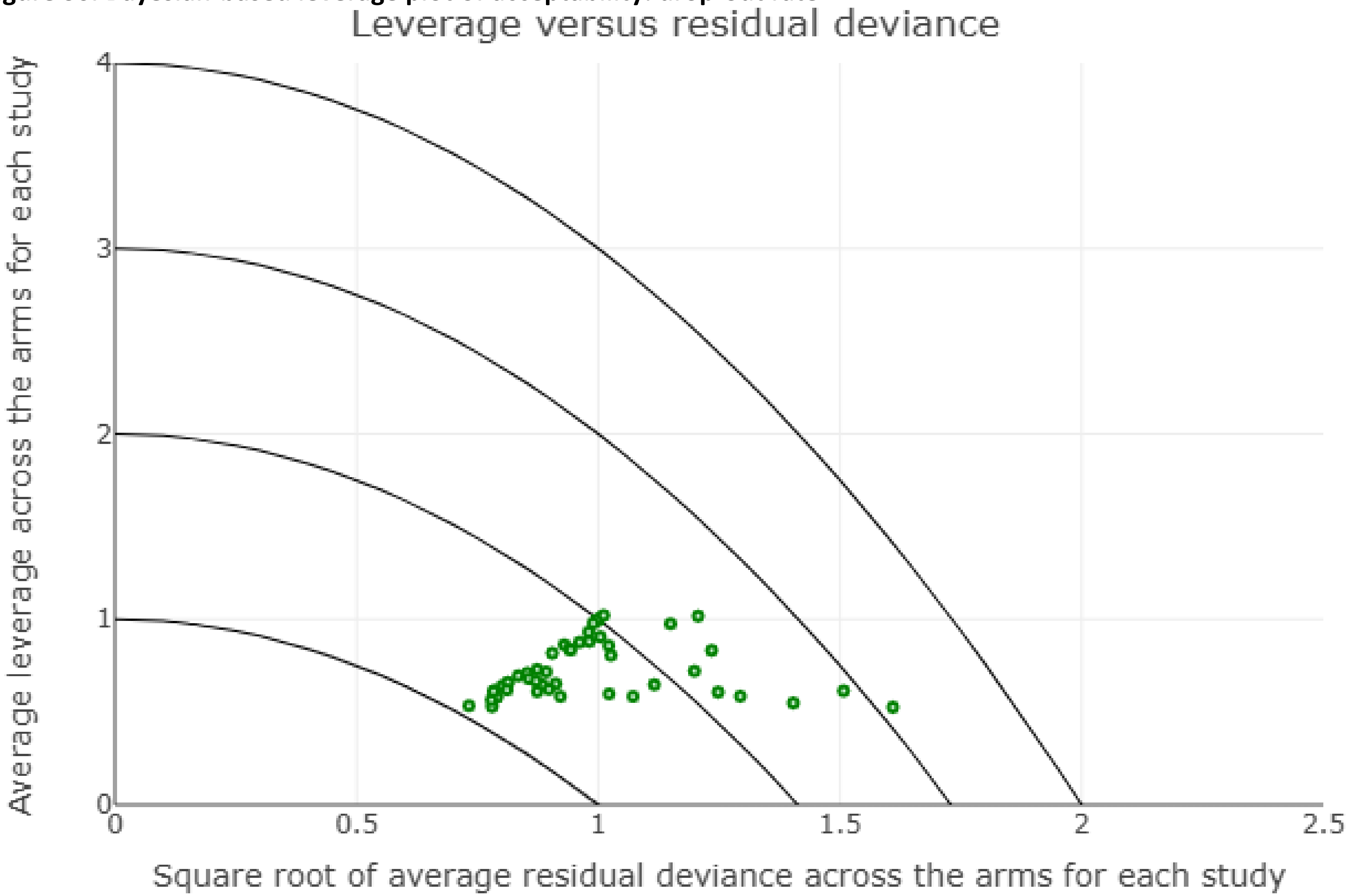

Abbreviation for Figure S6A-6I:

95%CIs: 95% confidence intervals; GLP-1 agonist: glucagon-like peptide-1 agonist; NMA: network meta-analysis; OR: odds ratio; RCT: randomized controlled trial; SGLT2 inhibitor: sodium–glucose cotransporter 2 inhibitor

Figure S7A overview of risk of bias

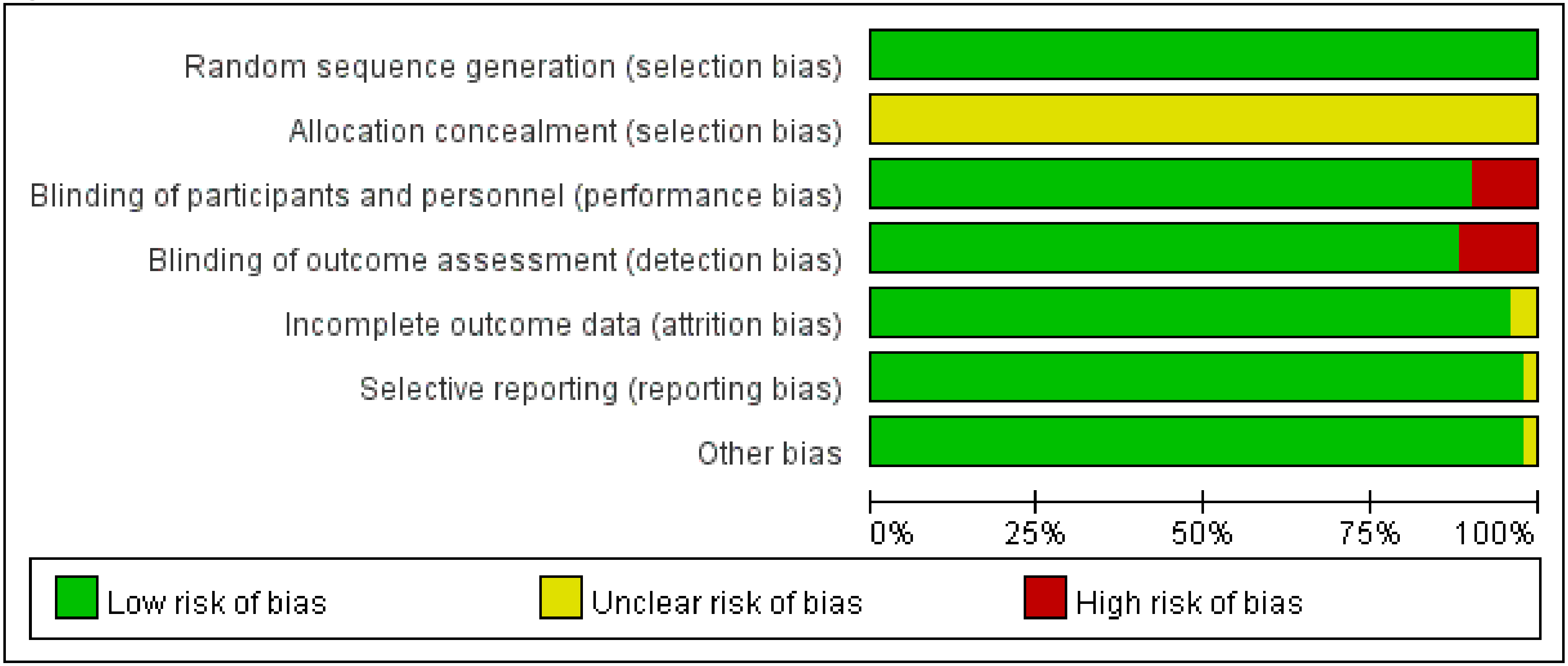

Figure S7B detailed risk of bias in each study

Study	Random sequence generation (selection bias)	Allocation concealment (selection bias)	Blinding of participants and personnel (performance bias)	Blinding of outcome assessment (detection bias)	Incomplete outcome data (attrition bias)	Selective reporting (reporting bias)	Other bias
Ahren, B. (2017) (SUSTAIN 2)	+	?	+	+	+	+	+
Anker, S.D. (2021) (EMPEROR-Preserved)	+	?	+	+	+	+	+
Aroda, V.R. (2017) (SUSTAIN 4)	+	?	–	–	+	+	+
Aronne, L.J. (2024) (SURMOUNT-4)	+	?	–	–	+	+	+
Barnett, A.H. (2014) (EMPA-REG RENAL)	+	?	+	+	+	+	+
Bhatt, D.L. (2021) (SCORED)	+	?	+	+	+	+	+
Bhatt, D.L. (2021) (SOLOIST-WHF)	+	?	+	+	+	+	+
Buse, J.B. (2018) (inTandem1)	+	?	+	+	+	+	+
Cannon, C.P. (2020) (VERTIS CV)	+	?	+	+	+	+	+
Cherney, D.Z.I.(2023) (SOTA-CKD3)	+	?	+	+	+	+	+
Davies, M. (2021) (STEP 2)	+	?	+	+	+	+	+
Garber, A. (2009) (LEAD-3 Mono)	+	?	+	+	+	+	+
Gerstein, H.C. (2019) (REWIND)	+	?	+	+	+	+	+
Gerstein, H.C. (2021) (AMPLITUDE-O)	+	?	+	+	+	+	+
Heerspink, H.J.L. (2020) (DAPA-CKD)	+	?	+	+	+	+	+
Hernandez, A.F. (2018) (Harmony Outcomes)	+	?	+	+	+	+	+
Herrington, W.G. (2023) (EMPA-KIDNEY)	+	?	+	+	+	+	+
Holman, R.R. (2017) (EXSCEL)	+	?	+	+	+	+	+
Home, P.D. (2017) (HARMONY 1-NCT00849056)	+	?	+	+	?	+	+
Home, P.D. (2017) (HARMONY 3-NCT00838903)	+	?	+	+	+	+	+
Home, P.D. (2017) (HARMONY 5-NCT00839527)	+	?	+	+	+	+	+
Januzzi, J.L. Jr. (2017) (CR017014)	+	?	+	+	+	+	+
Jastreboff, A.M. (2022) (SURMOUNT-1)	+	?	+	+	+	+	+
Kadowaki, T. (2022) (STEP 6)	+	?	+	+	+	+	+

Kaku, K. (2018) (SUSTAIN)	+	?	-	-	+	+	+
Kellerer, M. (2022) (SUSTAIN 11)	+	?	-	-	+	+	+
Lincoff, A.M. (2023) (SELECT)	+	?	+	+	+	+	+
Lingvay, I. (2019) (SUSTAIN 8)	+	?	+	+	+	+	+
Lock, J.P. (2021) (BEST) (NCT02558296)	+	?	+	+	?	?	?
Marso, S.P. (2016) (LEADER)	+	?	+	+	+	+	+
Marso, S.P. (2016) (SUSTAIN-6)	+	?	+	+	+	+	+
McMurray, J.J.V. (2019) (DAPA-HF)	+	?	+	+	+	+	+
Neal, B. (2017) (CANVAS)	+	?	+	+	+	+	+
Neal, B. (2017) (CANVAS-R)	+	?	+	+	+	+	+
Packer, M. (2020) (EMPEROR-Reduced)	+	?	+	+	+	+	+
Perkovic, V. (2019) (CREDENCE)	+	?	+	-	+	+	+
Pfeffer, M.A. (2015) (ELIXA)	+	?	+	+	+	+	+
Pi-Sunyer, X. (2015) (SCALE) (before 56 weeks)	+	?	+	+	+	+	+
Ridderstrale, M. (2014) (EMPA-REG H2H-SU)	+	?	+	+	+	+	+
Rodbard, H.W. (2019) (PIONEER 2)	+	?	-	-	+	+	+
Rubino, D. (2021) (STEP 4)	+	?	+	+	+	+	+
Solomon, S.D. (2022) (DELIVER)	+	?	+	+	+	+	+
Voors, A.A. (2022) (EMPULSE)	+	?	+	+	+	+	+
Wadden, T.A. (2021) (STEP-3)	+	?	+	+	+	+	+
Wadden, T.A. (2023) (SURMOUNT-3)	+	?	+	+	+	+	+
Wang, J. (2019) (AWARD-CHN2)	+	?	+	+	+	+	+
Weinstock, R.S. (2015) (AWARD-5)	+	?	+	+	+	+	+
Wilding, J.P. (2012)	+	?	+	+	+	+	+
Wiviott, S.D. (2019) (DECLARE-TIMI 58)	+	?	+	+	+	+	+
Zinman, B. (2015) (EMPA-REG OUTCOME)	+	?	+	+	+	+	+

Table S1: PRISMA 2020 checklist of the current network meta-analysis

Section and Topic	Item #	Checklist item	Page where item is reported
**TITLE**			
Title	1	Identify the report as a systematic review.	1
**ABSTRACT**			
Abstract	2	See the PRISMA 2020 for Abstracts checklist.	7-8
**INTRODUCTION**			
Rationale	3	Describe the rationale for the review in the context of existing knowledge.	9-10
Objectives	4	Provide an explicit statement of the objective(s) or question(s) the review addresses.	9-10
**METHODS**			
Eligibility criteria	5	Specify the inclusion and exclusion criteria for the review and how studies were grouped for the syntheses.	11-12
Information sources	6	Specify all databases, registers, websites, organisations, reference lists and other sources searched or consulted to identify studies. Specify the date when each source was last searched or consulted.	11-12
Search strategy	7	Present the full search strategies for all databases, registers and websites, including any filters and limits used.	11-12
Selection process	8	Specify the methods used to decide whether a study met the inclusion criteria of the review, including how many reviewers screened each record and each report retrieved, whether they worked independently, and if applicable, details of automation tools used in the process.	11-12
Data collection process	9	Specify the methods used to collect data from reports, including how many reviewers collected data from each report, whether they worked independently, any processes for obtaining or confirming data from study investigators, and if applicable, details of automation tools used in the process.	11-12
Data items	10a	List and define all outcomes for which data were sought. Specify whether all results that were compatible with each outcome domain in each study were sought (e.g. for all measures, time points, analyses), and if not, the methods used to decide which results to collect.	12-13
	10b	List and define all other variables for which data were sought (e.g. participant and intervention characteristics, funding sources). Describe any assumptions made about any missing or unclear information.	12-13
Study risk of bias assessment	11	Specify the methods used to assess risk of bias in the included studies, including details of the tool(s) used, how many reviewers assessed each study and whether they worked independently, and if applicable, details of automation tools used in the process.	12-13
Effect measures	12	Specify for each outcome the effect measure(s) (e.g. risk ratio, mean difference) used in the synthesis or presentation of results.	12-13
Synthesis methods	13a	Describe the processes used to decide which studies were eligible for each synthesis (e.g. tabulating the study intervention characteristics and comparing against the planned groups for each synthesis (item #5)).	12-13
	13b	Describe any methods required to prepare the data for presentation or synthesis, such as handling of missing summary statistics, or data conversions.	13-15
	13c	Describe any methods used to tabulate or visually display results of individual studies and syntheses.	13-15
	13d	Describe any methods used to synthesize results and provide a rationale for the choice(s). If meta-analysis was performed, describe the model(s), method(s) to identify the presence and extent of statistical heterogeneity, and software package(s) used.	13-15
	13e	Describe any methods used to explore possible causes of heterogeneity among study results (e.g. subgroup analysis, meta-regression).	13-15
	13f	Describe any sensitivity analyses conducted to assess robustness of the synthesized results.	13-15

Section and Topic	Item #	Checklist item	Page where item is reported
Reporting bias assessment	14	Describe any methods used to assess risk of bias due to missing results in a synthesis (arising from reporting biases).	13-15
Certainty assessment	15	Describe any methods used to assess certainty (or confidence) in the body of evidence for an outcome.	13-15
**RESULTS**			
Study selection	16a	Describe the results of the search and selection process, from the number of records identified in the search to the number of studies included in the review, ideally using a flow diagram.	16-17, Fig 1, eTab 2
	16b	Cite studies that might appear to meet the inclusion criteria, but which were excluded, and explain why they were excluded.	16-17, eTab 3
Study characteristics	17	Cite each included study and present its characteristics.	16-17, eTab 4
Risk of bias in studies	18	Present assessments of risk of bias for each included study.	16-17, eFig 7
Results of individual studies	19	For all outcomes, present, for each study: (a) summary statistics for each group (where appropriate) and (b) an effect estimate and its precision (e.g. confidence/credible interval), ideally using structured tables or plots.	16-17, eFig 3
Results of syntheses	20a	For each synthesis, briefly summarise the characteristics and risk of bias among contributing studies.	17-18, Fig 2
	20b	Present results of all statistical syntheses conducted. If meta-analysis was done, present for each the summary estimate and its precision (e.g. confidence/credible interval) and measures of statistical heterogeneity. If comparing groups, describe the direction of the effect.	17-18, Fig 3
	20c	Present results of all investigations of possible causes of heterogeneity among study results.	17-18, eTab 7
	20d	Present results of all sensitivity analyses conducted to assess the robustness of the synthesized results.	17-18
Reporting biases	21	Present assessments of risk of bias due to missing results (arising from reporting biases) for each synthesis assessed.	17-18, eFig 7
Certainty of evidence	22	Present assessments of certainty (or confidence) in the body of evidence for each outcome assessed.	17-18
**DISCUSSION**			
Discussion	23a	Provide a general interpretation of the results in the context of other evidence.	19-21
	23b	Discuss any limitations of the evidence included in the review.	21-22
	23c	Discuss any limitations of the review processes used.	21-22
	23d	Discuss implications of the results for practice, policy, and future research.	23
**OTHER INFORMATION**			
Registration and protocol	24a	Provide registration information for the review, including register name and registration number, or state that the review was not registered.	8
	24b	Indicate where the review protocol can be accessed, or state that a protocol was not prepared.	8
	24c	Describe and explain any amendments to information provided at registration or in the protocol.	8
Support	25	Describe sources of financial or non-financial support for the review, and the role of the funders or sponsors in the review.	24
Competing interests	26	Declare any competing interests of review authors.	24
Availability of data,	27	Report which of the following are publicly available and where they can be found: template data collection forms; data extracted from included	24

Section and Topic	Item #	Checklist item	Page where item is reported
code and other materials		studies; data used for all analyses; analytic code; any other materials used in the review.	

The current checklist followed the latest PRISMA 2020 guideline [1].

Table S2: Keyword used in each database and search results

Database	Keyword	Filter	Date	Result
PubMed	(intestine obstruction OR intestinal obstruction OR bowel obstruction OR ileus) AND (glucagon-like peptide-1 receptor agonist OR Sodium Glucose Cotransporter 2 Inhibitor OR lixisenatide OR orforglipron OR exenatide OR semaglutide OR liraglutide OR albiglutide OR dulaglutide OR tirzepatide OR bexagliflozin OR canagliflozin OR dapagliflozin OR empagliflozin OR ertugliflozin OR ipragliflozin OR luseogliflozin OR remogliflozin OR sergliflozin OR sotagliflozin OR tofogliflozin OR henagliflozin OR janagliflozin OR mizagliflozin OR velagliflozin OR enavogliflozin OR licogliflozin OR rongliflozin)	N/A	2025/01/21	21
ClinicalKey	(intestine obstruction OR intestinal obstruction OR bowel obstruction OR ileus) AND (glucagon-like peptide-1 receptor agonist OR Sodium Glucose Cotransporter 2 Inhibitor OR lixisenatide OR orforglipron OR exenatide OR semaglutide OR liraglutide OR albiglutide OR dulaglutide OR tirzepatide OR bexagliflozin OR canagliflozin OR dapagliflozin OR empagliflozin OR ertugliflozin OR ipragliflozin OR luseogliflozin OR remogliflozin OR sergliflozin OR sotagliflozin OR tofogliflozin OR henagliflozin OR janagliflozin OR mizagliflozin OR velagliflozin OR enavogliflozin OR licogliflozin OR rongliflozin)	N/A	2025/01/21	844
Cochrane CENTRAL	(intestine obstruction OR intestinal obstruction OR bowel obstruction OR ileus) AND (glucagon-like peptide-1 receptor agonist OR Sodium Glucose Cotransporter 2 Inhibitor OR lixisenatide OR orforglipron OR exenatide OR semaglutide OR liraglutide OR albiglutide OR dulaglutide OR tirzepatide OR bexagliflozin OR canagliflozin OR dapagliflozin OR empagliflozin OR ertugliflozin OR ipragliflozin OR luseogliflozin OR remogliflozin OR	N/A	2025/01/21	5

	sergliflozin OR sotagliflozin OR tofogliflozin OR henagliflozin OR janagliflozin OR mizagliflozin OR velagliflozin OR enavogliflozin OR licogliflozin OR rongliflozin)			
Embase	(intestine obstruction OR intestinal obstruction OR bowel obstruction OR ileus) AND (glucagon-like peptide-1 receptor agonist OR Sodium Glucose Cotransporter 2 Inhibitor) AND (random OR randomized OR randomised)	N/A	2025/01/21	37
ProQuest	(intestine obstruction OR intestinal obstruction OR bowel obstruction OR ileus) AND (glucagon-like peptide-1 receptor agonist OR Sodium Glucose Cotransporter 2 Inhibitor) AND (random OR randomized OR randomised)	N/A	2025/01/21	679
ScienceDirect	(intestine obstruction OR intestinal obstruction OR bowel obstruction OR ileus) AND (glucagon-like peptide-1 receptor agonist OR Sodium Glucose Cotransporter 2 Inhibitor) AND (random OR randomized OR randomised)	N/A	2025/01/21	1320
Web of Science	(intestine obstruction OR intestinal obstruction OR bowel obstruction OR ileus) AND (glucagon-like peptide-1 receptor agonist OR Sodium Glucose Cotransporter 2 Inhibitor) AND (random OR randomized OR randomised)	N/A	2025/01/21	1
ClinicalTrials.gov	(intestine obstruction OR intestinal obstruction OR bowel obstruction OR ileus) AND (glucagon-like peptide-1 receptor agonist OR Sodium Glucose Cotransporter 2 Inhibitor OR lixisenatide OR orforglipron OR exenatide OR semaglutide OR liraglutide OR albiglutide OR dulaglutide OR tirzepatide OR bexagliflozin OR canagliflozin OR dapagliflozin OR empagliflozin OR ertugliflozin OR ipragliflozin OR luseogliflozin OR remogliflozin OR sergliflozin OR sotagliflozin OR tofogliflozin OR henagliflozin OR janagliflozin OR mizagliflozin OR velagliflozin OR enavogliflozin OR licogliflozin OR rongliflozin)	N/A	2025/01/21	0

Abbreviation: N/A: not applied

Table S3: Excluded studies and reason

Reason	Numbers	References
A trend of difference in racial composition	1	[2]
Animal study	1	[3]
Case report	5	[4-8]
Not randomized controlled trial	9	[9-17]
Not related to GLP1-1 receptor agonists or SGLT2 inhibitors	2	[18,19]
Not report target outcome	88	[20-107]
Review article	3	[108-110]
Specifically use Sulfonylurea as control, which would affect intestinal transit in animal model	1	[111]

Table S4: Characteristics of the included studies

Study name	Baseline illness	Comparison	Subjects	Mean age (year)	Female (%)	Treatment duration	ClinicalTrials.gov	Route	Category	Country
Aronne, L.J. (2024) (SURMOUNT-4)[112]	patients with obesity	Tirzepatide 10-15mg<br>Placebo	335<br>335	49.0±13.0<br>48.0±12.0	70.4<br>70.7	36 weeks	NCT04660643	injection	GLP-1 agonist	Multiple countries
Cherney, D.Z.I.(2023) (SOTA-CKD3)[113]	patients with type 2 diabetes mellitus and chronic kidney disease	Sotagliflozin<br>Placebo	527<br>260	69.5±7.9<br>69.3±8.1	44.0<br>42.7	26 weeks	NCT03242252	oral	SGLT2 inhibitor	Multiple countries
Herrington, W.G. (2023) (EMPA-KIDNEY)[114]	patients with renal failure	Empagliflozin 10mg<br>Placebo	3304<br>3305	63.9±13.9<br>63.8±13.9	33.2<br>33.1	104 weeks	NCT03594110	oral	SGLT2 inhibitor	Multiple countries
Lincoff, A.M. (2023) (SELECT)[115]	patients with obesity	Inject Semaglutide 2.4 mg<br>Placebo	8803<br>8801	61.6±8.9<br>61.6±8.8	27.8<br>27.5	104 weeks	NCT03574597	injection	GLP-1 agonist	Multiple countries
Wadden, T.A. (2023) (SURMOUNT-3)[116]	patients with obesity	Tirzepatide 10-15mg<br>Placebo	287<br>292	45.4±12.6<br>45.7±11.8	63.1<br>62.7	84 weeks	NCT04657016	injection	GLP-1 agonist	Multiple countries
Jastreboff, A.M. (2022) (SURMOUNT-1)[117]	patients with obesity	Tirzepatide 5mg<br>Tirzepatide 10mg<br>Tirzepatide 15mg<br>Placebo	630<br>636<br>630<br>643	45.6±12.7<br>44.7±12.4<br>44.9±12.3<br>44.4±12.5	67.6<br>67.1<br>67.5<br>67.8	72 weeks	NCT04184622	injection	GLP-1 agonist	Multiple countries
Kadowaki, T. (2022) (STEP 6)[118]	patients with overweight or obesity with/without type 2 diabetes mellitus	Inject semaglutide 1.7 mg<br>Inject semaglutide 2.4 mg<br>Placebo	101<br>199<br>101	51.0±10.0<br>52.0±12.0<br>50.0±9.0	36.6<br>42.7<br>25.7	68 weeks	NCT03811574	injection	GLP-1 agonist	Multiple countries
Kellerer, M. (2022) (SUSTAIN 11)[119]	patients with type 2 diabetes mellitus	Inject semaglutide 1.0 mg<br>Control with insulin glargine and aspart	874<br>874	60.8±9.4<br>61.5±9.5	49.1<br>48.6	52 weeks	NCT03689374	injection	GLP-1 agonist	Multiple countries

Solomon, S.D. (2022) (DELIVER)[120]	patients with stabilized heart failure	Dapagliflozin 10mg<br>Placebo	3131<br>3132	71.8±9.6<br>71.5±9.5	43.6<br>44.2	120 weeks	NCT03619213	oral	SGLT2 inhibitor	Multiple countries
Voors, A.A. (2022) (EMPULSE)[121]	patients with acute heart failure and dyspnea	Empagliflozin 10mg<br>Placebo	265<br>265	71.0<br>70.0	32.5<br>35.1	13 weeks	NCT04157751	oral	SGLT2 inhibitor	Multiple countries
Anker, S.D. (2021) (EMPEROR-Preserved)[122]	patients with heart failure with preserved ejection fraction	Empagliflozin 10mg<br>Placebo	2997<br>2991	71.8±9.3<br>71.9±9.6	44.6<br>44.7	156 weeks	NCT03057951	oral	SGLT2 inhibitor	Multiple countries
Bhatt, D.L. (2021) (SCORED)[123]	patients with type 2 diabetes mellitus and chronic kidney disease	Sotagliflozin 200-400mg<br>Placebo	5292<br>5292	68.4±8.4<br>68.2±8.4	44.3<br>45.5	116 weeks	NCT03315143	oral	SGLT2 inhibitor	Multiple countries
Bhatt, D.L. (2021) (SOLOIST-WHF)[124]	patients with type 2 diabetes mellitus and heart failure	Sotagliflozin 200mg<br>Placebo	608<br>614	68.6±9.5<br>69.3±8.8	32.6<br>34.9	36 weeks	NCT03521934	oral	SGLT2 inhibitor	Multiple countries
Davies, M. (2021) (STEP 2)[125]	patients with type 2 diabetes mellitus and obesity	Inject semaglutide 1.0 mg<br>Inject Semaglutide 2.4 mg<br>Placebo	403<br>404<br>403	56.0±10.0<br>55.0±11.0<br>55.0±11.0	50.4<br>55.2<br>47.1	68 weeks	NCT03552757	injection	GLP-1 agonist	Multiple countries
Gerstein, H.C. (2021) (AMPLITUDE-O)[126]	patients with type 2 diabetes mellitus	Efpeglenatide 4 mg<br>Efpeglenatide 6 mg<br>Placebo	1359<br>1358<br>1359	64.6±8.2<br>64.7±8.2<br>64.4±8.3	32.5<br>35.6<br>30.8	104 weeks	NCT03496298	injection	GLP-1 agonist	Multiple countries
Lock, J.P. (2021) (BEST) (NCT02558296)[127]	patients with type 2 diabetes mellitus	Bexagliflozin 20mg<br>Placebo	1132<br>567	64.4±7.9<br>64.6±8.0	30.1<br>31.2	52 weeks	NCT02558296	oral	SGLT2 inhibitor	Multiple countries
Rubino, D. (2021) (STEP 4)[128]	patients with overweight or obesity	Inject semaglutide 2.4 mg<br>Placebo	535<br>268	47.0±12.0<br>46.0±12.0	80.2<br>76.5	68 weeks	NCT03548987	injection	GLP-1 agonist	Multiple countries
Wadden, T.A. (2021) (STEP-3)[129]	patients with obesity	Inject semaglutide 2.4 mg<br>Placebo	407<br>204	46.0±13.0<br>46.0±13.0	77.4<br>88.2	68 weeks	NCT03611582	injection	GLP-1 agonist	Multiple countries

Cannon, C.P. (2020) (VERTIS CV)[130]	patients with type 2 diabetes mellitus	Ertugliflozin 5 mg<br>Ertugliflozin 15 mg<br>Placebo	2752<br>2747<br>2747	64.3±8.2<br>64.4±8.0<br>64.4±8.0	29.1<br>30.3<br>30.7	182 weeks	NCT01986881	oral	SGLT2 inhibitor	Multiple countries
Heerspink, H.J.L. (2020) (DAPA-CKD)[131]	patients with renal failure	Dapagliflozin 10mg<br>Placebo	2152<br>2152	61.8±12.1<br>61.9±12.1	32.9<br>33.3	125 weeks	NCT03036150	oral	SGLT2 inhibitor	Multiple countries
Packer, M. (2020) (EMPEROR-Reduced)[132]	patients with chronic heart failure	Empagliflozin 10mg<br>Placebo	1863<br>1867	67.2±10.8<br>66.5±11.2	23.5<br>24.4	64 weeks	NCT03057977	oral	SGLT2 inhibitor	Multiple countries
Gerstein, H.C. (2019) (REWIND)[133]	patients with type 2 diabetes mellitus	Dulaglutide 1.5 mg<br>Placebo	4949<br>4952	66.2±6.5<br>66.2±6.5	46.6<br>46.1	281 weeks	NCT01394952	injection	GLP-1 agonist	Multiple countries
Lingvay, I. (2019) (SUSTAIN 8)[134]	patients with type 2 diabetes mellitus	Inject semaglutide 1.0 mg<br>Canagliflozin 300 mg	394<br>394	55.7±11.1<br>57.5±10.7	43.4<br>49.0	52 weeks	NCT03136484	oral/injection	GLP-1 agonist/SGLT2 inhibitor	Multiple countries
McMurray, J.J.V. (2019) (DAPA-HF)[135]	patients with stabilized heart failure	Dapagliflozin 10mg<br>Placebo	2373<br>2371	66.2±11.0<br>66.5±10.8	23.8<br>23.0	73 weeks	NCT03036124	oral	SGLT2 inhibitor	Multiple countries
Perkovic, V. (2019) (CREDENCE)[136]	patients with type 2 diabetes mellitus and nephropathy	Canagliflozin 100 mg<br>Placebo	2202<br>2199	62.9±9.2<br>63.2±9.2	34.6<br>33.3	130 weeks	NCT02065791	oral	SGLT2 inhibitor	Multiple countries
Rodbard, H.W. (2019) (PIONEER 2)[137]	patients with type 2 diabetes mellitus	Semaglutide 14mg<br>Empagliflozin 25mg	411<br>410	57.0±10.0<br>58.0±10.0	49.9<br>49.0	52 weeks	NCT02863328	oral	GLP-1 agonist/SGLT2 inhibitor	Multiple countries
Wang, J. (2019) (AWARD-CHN2)[138]	patients with type 2 diabetes mellitus	Dulaglutide 0.75-1.5 mg<br>Placebo with insulin glargine	505<br>250	54.8±9.8<br>55.4±9.2	45.0<br>44.4	52 weeks	NCT01648582	injection	GLP-1 agonist	Multiple countries
Wiviott, S.D. (2019) (DECLARE-TIMI 58)[139]	patients with atherosclerotic vascular disease	Dapagliflozin 10mg<br>Placebo	8582<br>8578	63.9±6.8<br>64.0±6.8	36.9<br>37.9	206 weeks	NCT01730534	oral	SGLT2 inhibitor	Multiple countries
Buse, J.B. (2018) (inTandem1)[140]	patients with type 1 diabetes mellitus	Sotagliflozin<br>Placebo	525<br>268	46.5±13.3<br>45.2±12.72	53.1<br>48.9	52 weeks	NCT02384941	oral	SGLT2 inhibitor	Multiple countries

Hernandez, A.F. (2018) (Harmony Outcomes)[141]	patients with type 2 diabetes mellitus	Albiglutide 30-50 mg<br>Placebo	4731<br>4732	64.1±8.7<br>64.2±8.7	30.2<br>31.0	86 weeks	NCT02465515	injection	GLP-1 agonist	Multiple countries
Kaku, K. (2018) (SUSTAIN)[142]	patients with type 2 diabetes mellitus	Inject semaglutide 0.5 mg<br>Inject semaglutide 1.0 mg<br>Control with standard care	239<br>241<br>121	58.0±10.6<br>58.7±10.2<br>59.2±10.1	30.5<br>27.8<br>25.8	61 weeks	NCT02207374	injection	GLP-1 agonist	Multiple countries
Ahren, B. (2017) (SUSTAIN 2)[143]	patients with type 2 diabetes mellitus	Inject semaglutide 1.0 mg<br>Inject semaglutide 0.5 mg<br>Placebo	409<br>409<br>407	56.0±9.4<br>54.8±10.2<br>54.6±10.4	49.9<br>50.6<br>51.1	56 weeks	NCT01930188	injection	GLP-1 agonist	Multiple countries
Aroda, V.R. (2017) (SUSTAIN 4)[144]	patients with type 2 diabetes mellitus	Inject semaglutide 0.5 mg<br>Inject semaglutide 1.0 mg<br>Control with insulin glargine	362<br>360<br>360	56.5±10.3<br>56.7±10.4<br>56.2±10.6	45.6<br>49.4<br>45.8	30 weeks	NCT02128932	injection	GLP-1 agonist	Multiple countries
Holman, R.R. (2017) (EXSCEL)[145]	patients with type 2 diabetes mellitus	Exenatide 2mg<br>Placebo	7356<br>7396	61.8±9.4<br>61.9±9.4	38.0<br>38.0	166 weeks	NCT01144338	injection	GLP-1 agonist	Multiple countries
Home, P.D. (2017) (HARMONY 1-NCT00849056)[146]	patients with type 2 diabetes mellitus	Albiglutide 30 mg<br>Placebo	150<br>151	55.2±10.0<br>54.9±9.4	38.7<br>41.7	52 weeks	NCT00849056	injection	GLP-1 agonist	Multiple countries
Home, P.D. (2017) (HARMONY 3-NCT00838903)[146]	patients with type 2 diabetes mellitus	Albiglutide 30 mg<br>Placebo	302<br>101	54.3±10.1<br>56.1±10.0	55.3<br>50.5	104 weeks	NCT00838903	injection	GLP-1 agonist	Multiple countries
Home, P.D. (2017) (HARMONY 5-NCT00839527)[146]	patients with type 2 diabetes mellitus	Albiglutide 30-50 mg<br>Placebo	271<br>115	54.5±9.5<br>55.7±9.6	50.2<br>39.1	156 weeks	NCT00839527	injection	GLP-1 agonist	Multiple countries

Januzzi, J.L. Jr. (2017) (CR017014)[147]	patients with type 2 diabetes mellitus	Canagliflozin 100 mg<br>Canagliflozin 300 mg<br>Placebo	241<br>236<br>237	64.3±6.5<br>63.4±6.0<br>63.2±6.2	48.5<br>45.3<br>39.7	104 weeks	NCT01106651	oral	SGLT2 inhibitor	Multiple countries
Neal, B. (2017) (CANVAS)[148]	patients with type 2 diabetes mellitus	Canagliflozin 100 mg<br>Canagliflozin 300 mg<br>Placebo	1445<br>1443<br>1442	62.2±8.0<br>62.8±8.1<br>62.3±7.9	33.5<br>34.6<br>33.7	126 weeks	NCT01032629	oral	SGLT2 inhibitor	Multiple countries
Neal, B. (2017) (CANVAS-R)[148]	patients with type 2 diabetes mellitus	Canagliflozin 300 mg<br>Placebo	2907<br>2905	63.9±8.4<br>64.0±8.3	36.2<br>38.2	126 weeks	NCT01989754	oral	SGLT2 inhibitor	Multiple countries
Marso, S.P. (2016) (LEADER)[149]	patients with type 2 diabetes mellitus	Liraglutide 1.8mg<br>Placebo	4668<br>4672	64.2±7.2<br>64.4±7.2	35.5<br>36.0	198 weeks	NCT01179048	injection	GLP-1 agonist	Multiple countries
Marso, S.P. (2016) (SUSTAIN-6)[150]	patients with type 2 diabetes mellitus	Inject semaglutide 0.5 mg<br>Inject semaglutide 1.0 mg<br>Placebo	826<br>822<br>1649	NA	40.1<br>37.0<br>40.0	109 weeks	NCT01720446	injection	GLP-1 agonist	Multiple countries
Pfeffer, M.A. (2015) (ELIXA)[151]	patients with type 2 diabetes mellitus and recent acute coronary syndrome	Lixisenatide 20ug<br>Placebo	3034<br>3034	59.9±9.7<br>60.6±9.6	30.4<br>30.9	100 weeks	NCT01147250	injection	GLP-1 agonist	Multiple countries
Pi-Sunyer, X. (2015) (SCALE) (before 56 weeks)[152]	patients with obesity	Liraglutide 3.0mg<br>Placebo	2487<br>1244	45.2±12.1<br>45.0±12.0	78.7<br>78.1	56 weeks	NCT01272219	injection	GLP-1 agonist	Multiple countries
Weinstock, R.S. (2015) (AWARD-5)[153]	patients with type 2 diabetes mellitus	Dulaglutide<br>Placebo or sitagliptin	710<br>177	54.0±9.9<br>54.9±9.1	55.2<br>49.2	26 weeks	NCT00734474	injection	GLP-1 agonist	Multiple countries
Zinman, B. (2015) (EMPA-REG OUTCOME)[154]	patients with type 2 diabetes mellitus	Empagliflozin 10mg<br>Empagliflozin 25mg<br>Placebo	2345<br>2342<br>2333	63.0±8.6<br>63.2±8.6<br>63.2±8.8	29.5<br>28.1<br>28.0	135 weeks	NCT01131676	oral	SGLT2 inhibitor	Multiple countries
Barnett, A.H. (2014) (EMPA-REG RENAL)[155]	patients with type 2 diabetes mellitus and kidney disease	Empagliflozin 10mg<br>Empagliflozin 25mg<br>Placebo	98<br>97<br>95	63.2±8.5<br>62.0±8.4<br>62.6±8.1	38.8<br>37.1<br>41.1	24 weeks	NCT01164501	oral	SGLT2 inhibitor	Multiple countries

Ridderstrale, M. (2014) (EMPA-REG H2H-SU)[156]	patients with type 2 diabetes mellitus and moderate-to-severe chronic kidney disease	Empagliflozin 25mg<br>Control with glimepiride	765<br>780	56.2±10.3<br>55.7±10.4	43.5<br>46.0	104 weeks	NCT01167881	oral	SGLT2 inhibitor	Multiple countries
Wilding, J.P. (2012)[157]	patients with type 2 diabetes mellitus and moderate-to-severe chronic kidney disease	Dapagliflozin 2.5 mg<br>Dapagliflozin 5 mg<br>Dapagliflozin 10 mg<br>Placebo	202<br>211<br>194<br>193	59.8±7.6<br>59.3±7.9<br>59.3±8.8<br>58.8±8.6	50.5<br>52.6<br>55.2<br>50.8	104 weeks	NCT00673231	oral	SGLT2 inhibitor	Multiple countries
Garber, A. (2009) (LEAD-3 Mono)[158]	patients with type 2 diabetes mellitus	Liraglutide 1.2-1.8 mg<br>Placebo with glimepiride	498<br>248	52.9±10.9<br>53.4±10.9	47.8<br>53.6	104 weeks	NCT00294723	injection	GLP-1 agonist	Multiple countries

Abbreviations: GLP-1 agonist: glucagon-like peptide-1 agonist; NA: not available; SGLT2 inhibitor: sodium–glucose cotransporter 2 inhibitor

Table S5: League table of NMA of acceptability: drop-out rate

Tirzepatide	.	.	.	.	.	.	.	.	.	.	.	.	.	.	*0.44 [0.35; 0.55]
1.10 [0.50; 2.42]	Oral_ semaglutide	.	.	.	.	.	.	.	.	.	*0.46 [0.22; 0.98]	.	.	.	.
*0.67 [0.50; 0.89]	0.61 [0.28; 1.32]	Canagliflozin	.	.	.	0.80 [0.44; 1.47]	.	.	.	.	.	.	.	.	*0.66 [0.55; 0.80]
*0.63 [0.46; 0.84]	0.57 [0.26; 1.24]	0.94 [0.71; 1.23]	Albiglutide	.	.	.	.	.	.	.	.	.	.	.	*0.71 [0.58; 0.86]
*0.58 [0.44; 0.76]	0.52 [0.24; 1.13]	0.87 [0.68; 1.10]	0.93 [0.72; 1.19]	Liraglutide	.	.	.	.	.	.	.	.	.	.	*0.76 [0.66; 0.89]
*0.56 [0.41; 0.78]	0.51 [0.23; 1.12]	0.84 [0.63; 1.13]	0.90 [0.66; 1.22]	0.97 [0.74; 1.28]	Dapagliflozin	.	.	.	.	.	.	.	.	.	*0.79 [0.62; 0.99]
*0.54 [0.42; 0.71]	0.49 [0.23; 1.06]	0.81 [0.65; 1.02]	0.87 [0.68; 1.12]	0.94 [0.76; 1.16]	0.97 [0.73; 1.28]	Inject_ semaglutide	.	.	.	.	.	.	.	.	*0.81 [0.70; 0.94]
*0.55 [0.39; 0.78]	0.50 [0.22; 1.10]	0.82 [0.59; 1.13]	0.88 [0.63; 1.22]	0.95 [0.69; 1.29]	0.97 [0.68; 1.39]	1.01 [0.74; 1.37]	Dulaglutide	.	.	.	.	.	.	.	0.81 [0.62; 1.06]
*0.52 [0.40; 0.67]	0.47 [0.22; 1.01]	*0.77 [0.61; 0.98]	0.82 [0.64; 1.06]	0.89 [0.72; 1.10]	0.92 [0.70; 1.21]	0.95 [0.77; 1.17]	0.94 [0.69; 1.28]	Sotagliflozin	.	.	.	.	.	.	*0.86 [0.74; 0.99]
*0.51 [0.29; 0.89]	0.46 [0.18; 1.15]	0.76 [0.43; 1.32]	0.81 [0.46; 1.42]	0.87 [0.50; 1.51]	0.90 [0.51; 1.59]	0.93 [0.54; 1.60]	0.92 [0.51; 1.66]	0.98 [0.57; 1.69]	Bexagliflozin	.	.	.	.	.	0.88 [0.52; 1.48]
*0.51 [0.37; 0.70]	0.46 [0.21; 1.02]	0.77 [0.57; 1.03]	0.82 [0.60; 1.11]	0.88 [0.67; 1.17]	0.91 [0.66; 1.26]	0.94 [0.71; 1.23]	0.93 [0.66; 1.33]	0.99 [0.75; 1.30]	1.01 [0.57; 1.80]	Exenatide	.	.	.	.	0.86 [0.69; 1.09]
*0.51 [0.40; 0.65]	*0.46 [0.22; 0.98]	*0.76 [0.62; 0.94]	0.82 [0.66; 1.02]	0.88 [0.74; 1.05]	0.91 [0.71; 1.17]	0.94 [0.79; 1.11]	0.93 [0.70; 1.24]	0.99 [0.83; 1.18]	1.01 [0.59; 1.72]	1.00 [0.78; 1.28]	Empagliflozin	.	.	.	*0.87 [0.79; 0.95]
*0.47 [0.35; 0.64]	*0.43 [0.20; 0.94]	*0.71 [0.54; 0.93]	0.76 [0.57; 1.01]	0.82 [0.63; 1.06]	0.84 [0.61; 1.15]	0.87 [0.67; 1.12]	0.86 [0.61; 1.21]	0.92 [0.71; 1.18]	0.94 [0.53; 1.65]	0.92 [0.68; 1.26]	0.93 [0.74; 1.16]	Ertugliflozin	.	.	0.94 [0.76; 1.15]
*0.46 [0.32; 0.68]	*0.42 [0.19; 0.95]	0.69 [0.48; 1.00]	0.74 [0.51; 1.08]	0.80 [0.57; 1.14]	0.82 [0.56; 1.22]	0.85 [0.60; 1.21]	0.85 [0.56; 1.28]	0.90 [0.64; 1.27]	0.92 [0.50; 1.70]	0.91 [0.61; 1.34]	0.91 [0.66; 1.26]	0.98 [0.67; 1.43]	Lixisenatide	.	0.95 [0.70; 1.30]
*0.45 [0.33; 0.62]	*0.41 [0.19; 0.90]	*0.68 [0.51; 0.91]	*0.72 [0.53; 0.98]	0.78 [0.59; 1.03]	0.80 [0.58; 1.11]	0.83 [0.63; 1.09]	0.83 [0.58; 1.17]	0.88 [0.67; 1.15]	0.90 [0.51; 1.59]	0.88 [0.64; 1.22]	0.89 [0.69; 1.13]	0.96 [0.70; 1.30]	0.98 [0.66; 1.44]	Efpeglenatide	0.98 [0.78; 1.23]
*0.44 [0.35; 0.55]	*0.40 [0.19; 0.85]	*0.66 [0.55; 0.80]	*0.71 [0.58; 0.86]	*0.76 [0.66; 0.89]	*0.79 [0.62; 0.99]	*0.81 [0.70; 0.94]	0.81 [0.62; 1.06]	*0.86 [0.74; 0.99]	0.88 [0.52; 1.48]	0.86 [0.69; 1.09]	*0.87 [0.79; 0.95]	0.94 [0.76; 1.15]	0.95 [0.70; 1.30]	0.98 [0.78; 1.23]	Placebo_ or_Control

Data present as OR [95%CIs]. Pairwise (upper-right portion) and network (lower-left portion) meta-analysis results are presented as estimate effect sizes for the outcome of acceptability (drop-out rate). Interventions are reported in order of mean ranking of acceptability, and outcomes are expressed as odds ratio (OR) (95% confidence intervals) (95%CIs). For the pairwise meta-analyses, OR of less than 1 indicate that the treatment specified in the row got more acceptability than that specified in the column. For the network meta-analysis (NMA), OR of less than 1 indicate that the treatment specified in the column got more acceptability than that specified in the row. Bold results marked

with * indicate statistical significance.

Abbreviation: 95%CIs: 95% confidence intervals; GLP-1 agonist: glucagon-like peptide-1 agonist; NMA: network meta-analysis; OR: odds ratio; RCT: randomized controlled trial; SGLT2 inhibitor: sodium–glucose cotransporter 2 inhibitor

Table S6A: SUCRA (Surface under the cumulative ranking) of primary outcome: intestine obstruction events

Treatment	Rank 1	Rank 2	Rank 3	Rank 4	Rank 5	Rank 6	Rank 7	Rank 8	Rank 9	Rank 10	Rank 11	Rank 12	Rank 13	Rank 14	Rank 15	Rank 16	SUCRA
Albiglutide	8.75E-05	0.0071	0.024125	0.0453625	0.063025	0.0690875	0.07725	0.1198625	0.075	0.0812875	0.0938875	0.110025	0.13245	0.0732375	0.02725	0.0009625	43.26883333
Bexagliflozin	0.0382	0.55835	0.1255375	0.0716125	0.0377875	0.026825	0.0216375	0.0194625	0.0136875	0.0130375	0.0126875	0.015325	0.017975	0.0144125	0.0130125	0.00045	82.0675
Canagliflozin	0	1.25E-05	2.50E-05	0.0002625	0.000425	0.00075	0.0013375	0.0020375	0.0041625	0.0093125	0.021125	0.0484	0.1180375	0.3735375	0.39725	0.023325	12.84558333
Dapagliflozin	2.50E-05	0.0001625	0.0016125	0.0061375	0.0166875	0.0357375	0.056875	0.072725	0.0989875	0.1194875	0.1566	0.231475	0.1403125	0.051675	0.0108	7.00E-04	35.90466667
Dulaglutide	0.0031	0.0383	0.156625	0.231575	0.200825	0.13095	0.080175	0.052725	0.0331375	0.0268875	0.0204125	0.01365	0.0074125	0.003225	0.0009875	1.25E-05	70.92866667
Efpeglenatide	0.0549125	0.0062625	0.0147625	0.0163625	0.0199875	0.0198	0.0199625	0.0181375	0.0204125	0.023575	0.0331125	0.0459125	0.0776625	0.1726125	0.4155	0.041025	24.46341667
Empagliflozin	0	3.00E-04	0.0021875	0.009625	0.023475	0.0478125	0.076575	0.0946875	0.1098125	0.1428375	0.20685	0.1442875	0.099525	0.0347875	0.0070375	2.00E-04	39.62308333
Ertugliflozin	0.0019	0.070225	0.1517375	0.1290875	0.1452625	0.1198625	0.0923	0.0498875	0.042875	0.043875	0.04285	0.0395625	0.032625	0.0289125	0.0086	0.0004375	64.3985
Exenatide	0.0001375	0.008675	0.02865	0.0529125	0.0839625	0.1225625	0.1356	0.0896	0.087975	0.0938375	0.0967125	0.08615	0.0675625	0.034275	0.0109625	0.000425	50.04358333
Inject_semaglutide	8.75E-05	0.002825	0.01415	0.04905	0.1089125	0.1372875	0.130575	0.11965	0.107475	0.1101125	0.0853875	0.0850875	0.0377375	0.0105125	0.0010125	0.0001375	52.21491667
Liraglutide	0.001625	0.191975	0.2924625	0.2316875	0.1330625	0.06965	0.0360125	0.016775	0.0097	0.00635	0.0052125	0.0030875	0.0015	0.0007125	0.0001875	0	80.42208333
Lixisenatide	0.899425	0.0248	0.0032125	0.001525	0.00105	0.000875	0.0007375	0.000725	0.000425	0.0010875	0.0010875	0.0008625	0.0017125	0.05765	0.004825	0	93.8335
Oral_semaglutide	0	0.0032625	0.0035375	0.0028625	0.0027625	0.0024	0.0013125	0.0012625	0.0012	0.0012125	0.00185	0.0029375	0.0045125	0.011325	0.030175	0.9293875	2.035666667
Placebo_or_Control	0	0	0.0001875	0.002025	0.011825	0.0663	0.1372	0.237575	0.2919875	0.159675	0.0762375	0.014775	0.0021125	1.00E-04	0	0	49.36
Sotagliflozin	5.00E-05	0.014025	0.0400875	0.0526375	0.064475	0.0715375	0.077025	0.061625	0.0638125	0.0701375	0.0869125	0.0970625	0.18555	0.07475	0.0391125	0.0012	42.642
Tirzepatide	0.00045	0.073725	0.1411	0.097275	0.086475	0.0785625	0.055425	0.0432625	0.03935	0.0972875	0.059075	0.0614	0.0733125	0.058275	0.0332875	0.0017375	55.948

Table S6B: SUCRA (Surface under the cumulative ranking) of primary outcome: intestine obstruction events in aspect of various dosage subgroup

Treatment	Rank 1	Rank 2	Rank 3	Rank 4	Rank 5	Rank 6	Rank 7	Rank 8	Rank 9	Rank 10	Rank 11	Rank 12	Rank 13	Rank 14	Rank 15	Rank 16	Rank 17	Rank 18	Rank 19	Rank 20	Rank 21	Rank 22	Rank 23	Rank 24	Rank 25	Rank 26	SUCRA
Albiglutide	0	0	0	0.0002375	0.00385	0.015725	0.0294875	0.0398625	0.0613125	0.06705	0.0642375	0.062275	0.059525	0.05605	0.060025	0.071225	0.0788125	0.0807125	0.0778875	0.0723875	0.0511125	0.029925	0.01355	0.0045	0.00025	0	44.8302
Bexagliflozin	0	0.0003125	0.0119	0.0644125	0.262825	0.2489125	0.0943375	0.0609	0.0432625	0.0305	0.0228625	0.017175	0.014425	0.0125	0.0124	0.01225	0.01315	0.013575	0.0135875	0.0136	0.0131375	0.0082125	0.0099625	0.0054875	0.0002625	5.00E-05	72.12425
Canagliflozin_high_dosage	0	0	0	0	0	5.00E-05	0.0005375	0.001375	0.0018125	0.001925	0.002025	0.0018625	0.002225	0.0033875	0.00485	0.007025	0.011125	0.0188875	0.033	0.0528	0.111625	0.2045875	0.2458125	0.2586125	0.0352	0.001275	15.25025
Canagliflozin_low_dosage	0	0	0	0	7.50E-05	0.0009375	0.00215	0.0026	0.0046875	0.00715	0.009275	0.0114875	0.0129875	0.0162125	0.022375	0.0310875	0.04195	0.0652125	0.1096375	0.15005	0.180825	0.157725	0.1303375	0.039975	0.00325	1.25E-05	24.3946
Dapagliflozin_high_dosage	0	0	0	1.25E-05	0.0001125	0.0010125	0.0032	0.008525	0.0166125	0.0297625	0.0441	0.0585	0.069175	0.0828875	0.1021125	0.1182875	0.1170375	0.1135875	0.101075	0.0711375	0.03805	0.0165125	0.0070875	0.0011	0.0001125	0	40.2102
Dapagliflozin_low_dosage	0	0	0	0	1.25E-05	0.0002125	0.0006125	0.0006875	0.0005125	7.00E-04	0.0006875	0.0017125	0.0012875	0.001125	0.0010625	0.001125	0.0013875	0.0019	0.0030625	0.0048	0.0058	0.008125	0.0185125	0.0309125	0.446475	0.4692875	3.35665
Dapagliflozin_medium_dosage	0.072425	0.1566	0.1216125	0.1152	0.1093375	0.0227625	0.011975	0.0067	0.004375	0.00385	0.0027625	0.002075	0.0019125	0.0024125	0.0025125	0.00245	0.0027875	0.003625	0.004425	0.0057125	0.0077375	0.012825	0.014075	0.117925	0.1847	0.007225	59.93795
Dulaglutide	0	0	0.00055	0.0033875	0.020475	0.059725	0.111425	0.1450625	0.1547125	0.13495	0.1012	0.072325	0.0478	0.0340125	0.029025	0.022625	0.0196	0.015025	0.0114	0.00715	0.0044125	0.002725	0.0016375	0.0006375	0.0001375	0	63.1735
Efpeglenatide_high_dosage	0	0	0.00015	0.0006125	0.0049125	0.01655	0.0145125	0.0148875	0.014975	0.0183	0.0170875	0.0168625	0.0155375	0.0168	0.0193625	0.0219375	0.02975	0.0343	0.04405	0.06065	0.0839875	0.1310625	0.1952125	0.1929875	0.0348375	0.000675	24.1422
Efpeglenatide_medium_dosage	0	0	0.000125	0.00265	0.01155	0.0280875	0.0335125	0.03265	0.031275	0.0315875	0.02795	0.0270625	0.025775	0.02545	0.027975	0.0294375	0.038	0.0459625	0.0514375	0.07345	0.0889	0.1238125	0.14915	0.076525	0.017375	3.00E-04	32.9431
Empagliflozin_high_dosage	0	0	1.25E-05	3.00E-04	0.00115	0.004	0.0134625	0.01955	0.0253125	0.0326625	0.036125	0.0413625	0.053825	0.052475	0.0608875	0.071875	0.0909875	0.0988875	0.10625	0.09935	0.08645	0.0584625	0.0310375	0.014475	0.0011	0	36.9985
Empagliflozin_low_dosage	0	0	0	3.75E-05	3.00E-04	0.0017	0.0056875	0.015475	0.0294375	0.044625	0.0623375	0.071125	0.0841875	0.0967375	0.1104	0.1156125	0.1161875	0.10215	0.07175	0.0411625	0.020425	0.00705	0.0028125	8.00E-04	0	0	43.8051
Ertugliflozin_high_dosage	0	0	0.0003625	0.0035375	0.0285875	0.0526	0.0812625	0.089975	0.08765	0.0800875	0.0663125	0.0578875	0.0507375	0.0491	0.049875	0.0542625	0.0546875	0.0507125	0.0420875	0.03705	0.0284	0.01885	0.0104875	0.0047125	0.000775	0	53.97
Ertugliflozin_low_dosage	0	6.25E-05	0.0021375	0.0220625	0.13115	0.2234125	0.1750625	0.1085375	0.06935	0.049525	0.03795	0.0285875	0.0233125	0.0213375	0.0182	0.01795	0.0153625	0.0147	0.011225	0.008725	0.0063875	0.0099125	0.004275	0.000625	0.00015	0	69.56215
Exenatide	0	0	2.50E-05	0.000525	0.004675	0.0153125	0.0294375	0.0493	0.0678375	0.0816625	0.085425	0.086075	0.0763875	0.0727	0.07455	0.0713375	0.06965	0.0671125	0.0554625	0.0391	0.030225	0.0144125	0.0063625	0.0022875	0.0001375	0	48.9102
Inject_semaglutide_high_dosage	0	0	1.25E-05	0.0001625	0.0033625	0.0131875	0.0307375	0.0519125	0.0758375	0.0955125	0.1138125	0.1014125	0.0920875	0.0848	0.0813	0.0791875	0.0642625	0.0467125	0.029525	0.01975	0.0102125	0.0048	0.001325	7.50E-05	1.25E-05	0	52.21445
Inject_semaglutide_low_dosage	0	0	0	8.75E-05	0.00275	0.0091	0.014825	0.021425	0.0211	0.0270125	0.030625	0.0306875	0.0291625	0.031375	0.0337375	0.042575	0.052125	0.0629375	0.08915	0.1011625	0.1195	0.109675	0.1077375	0.0518375	0.0114125	0	31.2781
Inject_semaglutide_medium_dosage	0	0	0	0.000175	0.01265	0.0449125	0.069175	0.0787125	0.0746875	0.0652625	0.0636375	0.058775	0.056075	0.056375	0.0513375	0.053925	0.0542	0.05475	0.05735	0.0627875	0.04305	0.0285875	0.01065	0.00285	7.50E-05	0	50.28375
Liraglutide	0	0	0.002775	0.00705	0.054225	0.15175	0.219375	0.1907375	0.13315	0.0872375	0.0543375	0.0333625	0.021375	0.0133375	0.009225	0.0066625	0.0053125	0.003775	0.002525	0.0016625	0.0010375	0.0006375	0.0002625	0.00015	3.75E-05	0	70.62435

Lixisenatide	0.1261875	0.129875	0.27175	0.2803375	0.1033375	0.0243875	0.00925	0.005775	0.004475	0.003325	0.0022	0.002125	0.001625	0.001725	0.0016	0.0023625	0.0024	0.002525	0.0025625	0.0030625	0.0043	0.0038	0.004125	0.0047125	0.00175	0.000425	88.16135
Oral_semaglutide	0	0	0	0.0002375	0.0014	0.0029	0.0013875	0.0012125	0.0011625	0.0012625	0.0008375	0.000875	0.0008625	8.00E-04	0.0007625	0.00115	0.0010875	0.0013375	0.0020375	0.002275	0.004175	0.0074875	0.0125875	0.17655	0.2568625	0.52075	3.97295
Placebo_or_Control	0	0	0	0	0	5.00E-05	0.0003875	0.002575	0.00985	0.032275	0.0812875	0.1482875	0.19185	0.207075	0.1614375	0.0971375	0.045275	0.0172375	0.0041	0.001075	1.00E-04	0	0	0	0	0	49.36425
Sotagliflozin	0	0	3.75E-05	4.00E-04	0.0039375	0.0181375	0.0291875	0.0393125	0.058625	0.0670625	0.0679	0.0642	0.0641125	0.0576125	0.0609125	0.0641375	0.070175	0.079625	0.0702625	0.06515	0.054025	0.03335	0.017525	0.0095	0.0048125	0	44.68665
Tirzepatide_high_dosage	0.272025	0.218325	0.247475	0.1471125	0.0763625	0.0120625	0.0044625	0.003175	0.001925	0.00185	0.0012625	0.000975	9.00E-04	0.0008125	0.0008375	0.001	0.0010875	0.00105	0.0012	0.00195	0.002225	9.00E-04	0.0005875	0.0004375	0	0	92.5874
Tirzepatide_low_dosage	0.16545	0.2480375	0.1692875	0.225625	0.1068375	0.0203125	0.0091	0.0064625	0.004475	0.0035875	0.0027875	0.002125	0.0022	0.0022375	0.00245	0.0025875	0.002575	0.00265	0.0027	0.00265	0.0033375	0.0060375	0.0042625	0.00195	0.000275	0	89.34345
Tirzepatide_medium_dosage	0.3639125	0.2467875	0.1717875	0.1258375	0.056125	0.0122	0.00545	0.0026125	0.0015875	0.001275	0.000975	8.00E-04	0.00065	0.0006625	0.0007875	0.0007875	0.001025	0.00105	0.00225	0.00135	0.0005625	0.000525	0.000625	0.000375	0	0	93.87445

Table S6C: SUCRA (Surface under the cumulative ranking) of acceptability: drop-out rate

Treatment	Rank 1	Rank 2	Rank 3	Rank 4	Rank 5	Rank 6	Rank 7	Rank 8	Rank 9	Rank 10	Rank 11	Rank 12	Rank 13	Rank 14	Rank 15	Rank 16	SUCRA
Albiglutide	0.0005875	0.027675	0.215025	0.256175	0.1852	0.1156875	0.0745625	0.048175	0.03005	0.0199875	0.0125	0.0080375	0.0039375	0.0018	0.0005625	3.75E-05	73.0475
Bexagliflozin	0.0041875	0.0271	0.096225	0.069275	0.0616	0.0518875	0.0541375	0.045525	0.0421125	0.042225	0.046325	0.0516	0.0552125	0.0554625	0.078575	0.21855	39.42433333
Canagliflozin	0.0011	0.0654875	0.4314625	0.2458875	0.11985	0.0644125	0.0324	0.018225	0.0103875	0.0053	0.0028	0.0016625	7.00E-04	0.00025	7.50E-05	0	80.138
Dapagliflozin	5.00E-05	0.006	0.05725	0.0926125	0.1198	0.121025	0.11315	0.1003875	0.0896375	0.0749375	0.067675	0.058925	0.043225	0.0273375	0.018275	0.0097125	54.29091667
Dulaglutide	0.0001125	0.0059875	0.052075	0.079825	0.0991125	0.1092625	0.1005375	0.091575	0.08235	0.0761375	0.072975	0.068575	0.0587625	0.042625	0.033025	0.0270625	50.05133333
Efpeglenatide	1.25E-05	0.0002875	0.002275	0.0054	0.009875	0.0158875	0.0210375	0.0285625	0.0389125	0.0494375	0.06655	0.0950125	0.1273875	0.1402125	0.179475	0.219675	19.38533333
Empagliflozin	0	0	0.000175	0.0010875	0.005775	0.019775	0.051525	0.1016	0.1604375	0.1969625	0.1946	0.15415	0.079325	0.02805	0.0059	0.0006375	38.31691667
Ertugliflozin	3.75E-05	0.000375	0.00355	0.008075	0.0149875	0.023425	0.032675	0.042675	0.0552125	0.0703125	0.094	0.12265	0.1488125	0.1356375	0.1347	0.112875	25.3655
Exenatide	1.00E-04	0.0024625	0.0186	0.0328375	0.051775	0.0689375	0.08	0.08525	0.0894625	0.0970375	0.103175	0.1061	0.0905375	0.0721	0.0586875	0.0429375	39.70641667
Inject_semaglutide	0	0.0007375	0.00755	0.0322625	0.07255	0.117	0.152125	0.159025	0.1391625	0.1174	0.089225	0.06	0.03335	0.0145125	0.004125	0.000975	50.68575
Liraglutide	0	0.004075	0.043225	0.118425	0.1844	0.1918625	0.153	0.109575	0.0736	0.05105	0.0319375	0.0212875	0.0108	0.00465	0.001625	0.0004875	62.33575
Lixisenatide	0.0001125	0.0019375	0.0115625	0.0199	0.027175	0.03325	0.0410875	0.0451375	0.0500875	0.054725	0.0659	0.0828	0.0979125	0.105325	0.1389375	0.22415	25.08816667
Oral_semaglutide	0.5988875	0.267475	0.0440125	0.0231875	0.014375	0.0099375	0.0074	0.0058875	0.0047	0.00385	0.0035625	0.0037	0.0028625	0.0027375	0.003175	0.00425	93.70283333
Placebo_or_Control	0	0	0	0	0	0	0	0	7.50E-05	0.0007375	0.0071375	0.0455125	0.163875	0.324	0.325825	0.1328375	11.25425
Sotagliflozin	0	0.0002125	0.003675	0.0138375	0.0331625	0.0575875	0.08635	0.1184	0.1338	0.1399	0.1416375	0.1199875	0.0833	0.0453	0.0170375	0.0058125	41.35658333
Tirzepatide	0.3948125	0.5901875	0.0133375	0.0012125	0.0003625	6.25E-05	1.25E-05	0	1.25E-05	0	0	0	0	0	0	0	95.85041667

Table S7A: inconsistency within the network meta-analysis of primary outcome: intestine obstruction events

	Comparison	No.Studies	NMA	Direct	Indirect	Difference	Diff_95CI_lower	Diff_95CI_upper	*p* value
1	Albiglutide:Bexagliflozin	0	1.429251017	NA	1.429251017	NA	NA	NA	NA
2	Albiglutide:Canagliflozin	0	-0.896195645	NA	-0.896195645	NA	NA	NA	NA
3	Albiglutide:Dapagliflozin	0	-0.142083497	NA	-0.142083497	NA	NA	NA	NA
4	Albiglutide:Dulaglutide	0	0.646781764	NA	0.646781764	NA	NA	NA	NA
5	Albiglutide:Efpeglenatide	0	-0.872373962	NA	-0.872373962	NA	NA	NA	NA
6	Albiglutide:Empagliflozin	0	-0.104755834	NA	-0.104755834	NA	NA	NA	NA
7	Albiglutide:Ertugliflozin	0	0.554174272	NA	0.554174272	NA	NA	NA	NA
8	Albiglutide:Exenatide	0	0.038259451	NA	0.038259451	NA	NA	NA	NA
9	Albiglutide:Inject_semaglutide	0	0.103900509	NA	0.103900509	NA	NA	NA	NA
10	Albiglutide:Liraglutide	0	0.862018454	NA	0.862018454	NA	NA	NA	NA
11	Albiglutide:Lixisenatide	0	1.14068438	NA	1.14068438	NA	NA	NA	NA
12	Albiglutide:Oral_semaglutide	0	-1.203368122	NA	-1.203368122	NA	NA	NA	NA
13	Albiglutide:Placebo_or_Control	4	0.042072092	0.042072092	NA	NA	NA	NA	NA
14	Albiglutide:Sotagliflozin	0	0.088116525	NA	0.088116525	NA	NA	NA	NA
15	Albiglutide:Tirzepatide	0	0.267418523	NA	0.267418523	NA	NA	NA	NA
16	Bexagliflozin:Canagliflozin	0	-2.325446662	NA	-2.325446662	NA	NA	NA	NA
17	Bexagliflozin:Dapagliflozin	0	-1.571334514	NA	-1.571334514	NA	NA	NA	NA
18	Bexagliflozin:Dulaglutide	0	-0.782469253	NA	-0.782469253	NA	NA	NA	NA
19	Bexagliflozin:Efpeglenatide	0	-2.301624979	NA	-2.301624979	NA	NA	NA	NA
20	Bexagliflozin:Empagliflozin	0	-1.534006851	NA	-1.534006851	NA	NA	NA	NA

21	Bexagliflozin:Ertugliflozin	0	-0.875076745	NA	-0.875076745	NA	NA	NA	NA
22	Bexagliflozin:Exenatide	0	-1.390991566	NA	-1.390991566	NA	NA	NA	NA
23	Bexagliflozin:Inject_semaglutide	0	-1.325350508	NA	-1.325350508	NA	NA	NA	NA
24	Bexagliflozin:Liraglutide	0	-0.567232563	NA	-0.567232563	NA	NA	NA	NA
25	Bexagliflozin:Lixisenatide	0	-0.288566637	NA	-0.288566637	NA	NA	NA	NA
26	Bexagliflozin:Oral_semaglutide	0	-2.632619139	NA	-2.632619139	NA	NA	NA	NA
27	Bexagliflozin:Placebo_or_Control	1	-1.387178926	-1.387178926	NA	NA	NA	NA	NA
28	Bexagliflozin:Sotagliflozin	0	-1.341134492	NA	-1.341134492	NA	NA	NA	NA
29	Bexagliflozin:Tirzepatide	0	-1.161832494	NA	-1.161832494	NA	NA	NA	NA
30	Canagliflozin:Dapagliflozin	0	0.754112147	NA	0.754112147	NA	NA	NA	NA
31	Canagliflozin:Dulaglutide	0	1.542977409	NA	1.542977409	NA	NA	NA	NA
32	Canagliflozin:Efpeglenatide	0	0.023821683	NA	0.023821683	NA	NA	NA	NA
33	Canagliflozin:Empagliflozin	0	0.791439811	NA	0.791439811	NA	NA	NA	NA
34	Canagliflozin:Ertugliflozin	0	1.450369917	NA	1.450369917	NA	NA	NA	NA
35	Canagliflozin:Exenatide	0	0.934455095	NA	0.934455095	NA	NA	NA	NA
36	Canagliflozin:Inject_semaglutide	1	1.000096153	1.096067758	0.988874288	0.10719347	-3.278590572	3.492977513	0.950521264
37	Canagliflozin:Liraglutide	0	1.758214098	NA	1.758214098	NA	NA	NA	NA
38	Canagliflozin:Lixisenatide	0	2.036880025	NA	2.036880025	NA	NA	NA	NA
39	Canagliflozin:Oral_semaglutide	0	-0.307172478	NA	-0.307172478	NA	NA	NA	NA
40	Canagliflozin:Placebo_or_Control	4	0.938267736	0.929407928	1.036601398	-0.10719347	-3.492977513	3.278590572	0.950521264
41	Canagliflozin:Sotagliflozin	0	0.98431217	NA	0.98431217	NA	NA	NA	NA
42	Canagliflozin:Tirzepatide	0	1.163614167	NA	1.163614167	NA	NA	NA	NA

43	Dapagliflozin:Dulaglutide	0	0.788865261	NA	0.788865261	NA	NA	NA	NA
44	Dapagliflozin:Efpeglenatide	0	-0.730290465	NA	-0.730290465	NA	NA	NA	NA
45	Dapagliflozin:Empagliflozin	0	0.037327664	NA	0.037327664	NA	NA	NA	NA
46	Dapagliflozin:Ertugliflozin	0	0.69625777	NA	0.69625777	NA	NA	NA	NA
47	Dapagliflozin:Exenatide	0	0.180342948	NA	0.180342948	NA	NA	NA	NA
48	Dapagliflozin:Inject_semaglutide	0	0.245984006	NA	0.245984006	NA	NA	NA	NA
49	Dapagliflozin:Liraglutide	0	1.004101951	NA	1.004101951	NA	NA	NA	NA
50	Dapagliflozin:Lixisenatide	0	1.282767877	NA	1.282767877	NA	NA	NA	NA
51	Dapagliflozin:Oral_semaglutide	0	-1.061284625	NA	-1.061284625	NA	NA	NA	NA
52	Dapagliflozin:Placebo_or_Control	5	0.184155589	0.184155589	NA	NA	NA	NA	NA
53	Dapagliflozin:Sotagliflozin	0	0.230200022	NA	0.230200022	NA	NA	NA	NA
54	Dapagliflozin:Tirzepatide	0	0.40950202	NA	0.40950202	NA	NA	NA	NA
55	Dulaglutide:Efpeglenatide	0	-1.519155726	NA	-1.519155726	NA	NA	NA	NA
56	Dulaglutide:Empagliflozin	0	-0.751537598	NA	-0.751537598	NA	NA	NA	NA
57	Dulaglutide:Ertugliflozin	0	-0.092607492	NA	-0.092607492	NA	NA	NA	NA
58	Dulaglutide:Exenatide	0	-0.608522313	NA	-0.608522313	NA	NA	NA	NA
59	Dulaglutide:Inject_semaglutide	0	-0.542881255	NA	-0.542881255	NA	NA	NA	NA
60	Dulaglutide:Liraglutide	0	0.21523669	NA	0.21523669	NA	NA	NA	NA
61	Dulaglutide:Lixisenatide	0	0.493902616	NA	0.493902616	NA	NA	NA	NA
62	Dulaglutide:Oral_semaglutide	0	-1.850149886	NA	-1.850149886	NA	NA	NA	NA
63	Dulaglutide:Placebo_or_Control	3	-0.604709673	-0.604709673	NA	NA	NA	NA	NA
64	Dulaglutide:Sotagliflozin	0	-0.558665239	NA	-0.558665239	NA	NA	NA	NA

65	Dulaglutide:Tirzepatide	0	-0.379363241	NA	-0.379363241	NA	NA	NA	NA
66	Efpeglenatide:Empagliflozin	0	0.767618128	NA	0.767618128	NA	NA	NA	NA
67	Efpeglenatide:Ertugliflozin	0	1.426548234	NA	1.426548234	NA	NA	NA	NA
68	Efpeglenatide:Exenatide	0	0.910633412	NA	0.910633412	NA	NA	NA	NA
69	Efpeglenatide:Inject_semaglutide	0	0.97627447	NA	0.97627447	NA	NA	NA	NA
70	Efpeglenatide:Liraglutide	0	1.734392416	NA	1.734392416	NA	NA	NA	NA
71	Efpeglenatide:Lixisenatide	0	2.013058342	NA	2.013058342	NA	NA	NA	NA
72	Efpeglenatide:Oral_semaglutide	0	-0.330994161	NA	-0.330994161	NA	NA	NA	NA
73	Efpeglenatide:Placebo_or_Control	1	0.914446053	0.914446053	NA	NA	NA	NA	NA
74	Efpeglenatide:Sotagliflozin	0	0.960490487	NA	0.960490487	NA	NA	NA	NA
75	Efpeglenatide:Tirzepatide	0	1.139792484	NA	1.139792484	NA	NA	NA	NA
76	Empagliflozin:Ertugliflozin	0	0.658930106	NA	0.658930106	NA	NA	NA	NA
77	Empagliflozin:Exenatide	0	0.143015284	NA	0.143015284	NA	NA	NA	NA
78	Empagliflozin:Inject_semaglutide	0	0.208656342	NA	0.208656342	NA	NA	NA	NA
79	Empagliflozin:Liraglutide	0	0.966774287	NA	0.966774287	NA	NA	NA	NA
80	Empagliflozin:Lixisenatide	0	1.245440214	NA	1.245440214	NA	NA	NA	NA
81	Empagliflozin:Oral_semaglutide	1	-1.098612289	-1.098612289	NA	NA	NA	NA	NA
82	Empagliflozin:Placebo_or_Control	7	0.146827925	0.146827925	NA	NA	NA	NA	NA
83	Empagliflozin:Sotagliflozin	0	0.192872359	NA	0.192872359	NA	NA	NA	NA
84	Empagliflozin:Tirzepatide	0	0.372174356	NA	0.372174356	NA	NA	NA	NA
85	Ertugliflozin:Exenatide	0	-0.515914822	NA	-0.515914822	NA	NA	NA	NA
86	Ertugliflozin:Inject_semaglutide	0	-0.450273764	NA	-0.450273764	NA	NA	NA	NA

87	Ertugliflozin:Liraglutide	0	0.307844181	NA	0.307844181	NA	NA	NA	NA
88	Ertugliflozin:Lixisenatide	0	0.586510108	NA	0.586510108	NA	NA	NA	NA
89	Ertugliflozin:Oral_semaglutide	0	-1.757542395	NA	-1.757542395	NA	NA	NA	NA
90	Ertugliflozin:Placebo_or_Control	1	-0.512102181	-0.512102181	NA	NA	NA	NA	NA
91	Ertugliflozin:Sotagliflozin	0	-0.466057747	NA	-0.466057747	NA	NA	NA	NA
92	Ertugliflozin:Tirzepatide	0	-0.28675575	NA	-0.28675575	NA	NA	NA	NA
93	Exenatide:Inject_semaglutide	0	0.065641058	NA	0.065641058	NA	NA	NA	NA
94	Exenatide:Liraglutide	0	0.823759003	NA	0.823759003	NA	NA	NA	NA
95	Exenatide:Lixisenatide	0	1.102424929	NA	1.102424929	NA	NA	NA	NA
96	Exenatide:Oral_semaglutide	0	-1.241627573	NA	-1.241627573	NA	NA	NA	NA
97	Exenatide:Placebo_or_Control	1	0.003812641	0.003812641	NA	NA	NA	NA	NA
98	Exenatide:Sotagliflozin	0	0.049857074	NA	0.049857074	NA	NA	NA	NA
99	Exenatide:Tirzepatide	0	0.229159072	NA	0.229159072	NA	NA	NA	NA
100	Inject_semaglutide:Liraglutide	0	0.758117945	NA	0.758117945	NA	NA	NA	NA
101	Inject_semaglutide:Lixisenatide	0	1.036783871	NA	1.036783871	NA	NA	NA	NA
102	Inject_semaglutide:Oral_semaglutide	0	-1.307268631	NA	-1.307268631	NA	NA	NA	NA
103	Inject_semaglutide:Placebo_or_Control	10	-0.061828417	-0.05946636	-0.16665983	0.10719347	-3.278590572	3.492977513	0.950521264
104	Inject_semaglutide:Sotagliflozin	0	-0.015783984	NA	-0.015783984	NA	NA	NA	NA
105	Inject_semaglutide:Tirzepatide	0	0.163518014	NA	0.163518014	NA	NA	NA	NA
106	Liraglutide:Lixisenatide	0	0.278665926	NA	0.278665926	NA	NA	NA	NA
107	Liraglutide:Oral_semaglutide	0	-2.065386576	NA	-2.065386576	NA	NA	NA	NA
108	Liraglutide:Placebo_or_Control	3	-0.819946362	-0.819946362	NA	NA	NA	NA	NA

109	Liraglutide:Sotagliflozin	0	-0.773901929	NA	-0.773901929	NA	NA	NA	NA
110	Liraglutide:Tirzepatide	0	-0.594599931	NA	-0.594599931	NA	NA	NA	NA
111	Lixisenatide:Oral_semaglutide	0	-2.344052502	NA	-2.344052502	NA	NA	NA	NA
112	Lixisenatide:Placebo_or_Control	1	-1.098612289	-1.098612289	NA	NA	NA	NA	NA
113	Lixisenatide:Sotagliflozin	0	-1.052567855	NA	-1.052567855	NA	NA	NA	NA
114	Lixisenatide:Tirzepatide	0	-0.873265857	NA	-0.873265857	NA	NA	NA	NA
115	Oral_semaglutide:Placebo_or_Control	0	1.245440214	NA	1.245440214	NA	NA	NA	NA
116	Oral_semaglutide:Sotagliflozin	0	1.291484647	NA	1.291484647	NA	NA	NA	NA
117	Oral_semaglutide:Tirzepatide	0	1.470786645	NA	1.470786645	NA	NA	NA	NA
118	Sotagliflozin:Placebo_or_Control	4	-0.046044433	-0.046044433	NA	NA	NA	NA	NA
119	Tirzepatide:Placebo_or_Control	3	-0.225346431	-0.225346431	NA	NA	NA	NA	NA
120	Sotagliflozin:Tirzepatide	0	0.179301998	NA	0.179301998	NA	NA	NA	NA

Table S7B: inconsistency within the network meta-analysis of primary outcome: intestine obstruction events in aspect of various dosage subgroup

	Comparison	No.Studies	NMA	Direct	Indirect	Difference	Diff_95CI_lower	Diff_95CI_upper	*p* value
1	Albiglutide:Bexagliflozin	0	1.429251017	NA	1.429251017	NA	NA	NA	NA
2	Albiglutide:Canagliflozin_high_dosage	0	-1.186720088	NA	-1.186720088	NA	NA	NA	NA
3	Albiglutide:Canagliflozin_low_dosage	0	-0.744542199	NA	-0.744542199	NA	NA	NA	NA
4	Albiglutide:Dapagliflozin_high_dosage	0	-0.145936237	NA	-0.145936237	NA	NA	NA	NA
5	Albiglutide:Dapagliflozin_low_dosage	0	-1.127955047	NA	-1.127955047	NA	NA	NA	NA
6	Albiglutide:Dapagliflozin_medium_dosage	0	0.023809848	NA	0.023809848	NA	NA	NA	NA
7	Albiglutide:Dulaglutide	0	0.646781764	NA	0.646781764	NA	NA	NA	NA
8	Albiglutide:Efpeglenatide_high_dosage	0	-1.055801917	NA	-1.055801917	NA	NA	NA	NA
9	Albiglutide:Efpeglenatide_medium_dosage	0	-0.648125234	NA	-0.648125234	NA	NA	NA	NA
10	Albiglutide:Empagliflozin_high_dosage	0	-0.174764775	NA	-0.174764775	NA	NA	NA	NA
11	Albiglutide:Empagliflozin_low_dosage	0	-0.097917792	NA	-0.097917792	NA	NA	NA	NA
12	Albiglutide:Ertugliflozin_high_dosage	0	0.266309934	NA	0.266309934	NA	NA	NA	NA
13	Albiglutide:Ertugliflozin_low_dosage	0	0.959821613	NA	0.959821613	NA	NA	NA	NA
14	Albiglutide:Exenatide	0	0.038259451	NA	0.038259451	NA	NA	NA	NA
15	Albiglutide:Inject_semaglutide_high_dosage	0	0.093350698	NA	0.093350698	NA	NA	NA	NA
16	Albiglutide:Inject_semaglutide_low_dosage	0	-0.353226755	NA	-0.353226755	NA	NA	NA	NA
17	Albiglutide:Inject_semaglutide_medium_dosage	0	-0.000743006	NA	-0.000743006	NA	NA	NA	NA
18	Albiglutide:Liraglutide	0	0.862018454	NA	0.862018454	NA	NA	NA	NA
19	Albiglutide:Lixisenatide	0	1.14068438	NA	1.14068438	NA	NA	NA	NA

20	Albiglutide:Oral_semaglutide	0	-1.273377064	NA	-1.273377064	NA	NA	NA	NA
21	Albiglutide:Placebo_or_Control	4	0.042072092	0.042072092	NA	NA	NA	NA	NA
22	Albiglutide:Sotagliflozin	0	0.088116525	NA	0.088116525	NA	NA	NA	NA
23	Albiglutide:Tirzepatide_high_dosage	0	1.121830719	NA	1.121830719	NA	NA	NA	NA
24	Albiglutide:Tirzepatide_low_dosage	0	1.121830719	NA	1.121830719	NA	NA	NA	NA
25	Albiglutide:Tirzepatide_medium_dosage	0	1.131301981	NA	1.131301981	NA	NA	NA	NA
26	Bexagliflozin:Canagliflozin_high_dosage	0	-2.615971105	NA	-2.615971105	NA	NA	NA	NA
27	Bexagliflozin:Canagliflozin_low_dosage	0	-2.173793216	NA	-2.173793216	NA	NA	NA	NA
28	Bexagliflozin:Dapagliflozin_high_dosage	0	-1.575187254	NA	-1.575187254	NA	NA	NA	NA
29	Bexagliflozin:Dapagliflozin_low_dosage	0	-2.557206065	NA	-2.557206065	NA	NA	NA	NA
30	Bexagliflozin:Dapagliflozin_medium_dosage	0	-1.405441169	NA	-1.405441169	NA	NA	NA	NA
31	Bexagliflozin:Dulaglutide	0	-0.782469253	NA	-0.782469253	NA	NA	NA	NA
32	Bexagliflozin:Efpeglenatide_high_dosage	0	-2.485052934	NA	-2.485052934	NA	NA	NA	NA
33	Bexagliflozin:Efpeglenatide_medium_dosage	0	-2.077376251	NA	-2.077376251	NA	NA	NA	NA
34	Bexagliflozin:Empagliflozin_high_dosage	0	-1.604015792	NA	-1.604015792	NA	NA	NA	NA
35	Bexagliflozin:Empagliflozin_low_dosage	0	-1.527168809	NA	-1.527168809	NA	NA	NA	NA
36	Bexagliflozin:Ertugliflozin_high_dosage	0	-1.162941083	NA	-1.162941083	NA	NA	NA	NA
37	Bexagliflozin:Ertugliflozin_low_dosage	0	-0.469429404	NA	-0.469429404	NA	NA	NA	NA
38	Bexagliflozin:Exenatide	0	-1.390991566	NA	-1.390991566	NA	NA	NA	NA
39	Bexagliflozin:Inject_semaglutide_high_dosage	0	-1.335900319	NA	-1.335900319	NA	NA	NA	NA
40	Bexagliflozin:Inject_semaglutide_low_dosage	0	-1.782477772	NA	-1.782477772	NA	NA	NA	NA
41	Bexagliflozin:Inject_semaglutide_medium_dosage	0	-1.429994023	NA	-1.429994023	NA	NA	NA	NA

42	Bexagliflozin:Liraglutide	0	-0.567232563	NA	-0.567232563	NA	NA	NA	NA
43	Bexagliflozin:Lixisenatide	0	-0.288566637	NA	-0.288566637	NA	NA	NA	NA
44	Bexagliflozin:Oral_semaglutide	0	-2.702628081	NA	-2.702628081	NA	NA	NA	NA
45	Bexagliflozin:Placebo_or_Control	1	-1.387178926	-1.387178926	NA	NA	NA	NA	NA
46	Bexagliflozin:Sotagliflozin	0	-1.341134492	NA	-1.341134492	NA	NA	NA	NA
47	Bexagliflozin:Tirzepatide_high_dosage	0	-0.307420298	NA	-0.307420298	NA	NA	NA	NA
48	Bexagliflozin:Tirzepatide_low_dosage	0	-0.307420298	NA	-0.307420298	NA	NA	NA	NA
49	Bexagliflozin:Tirzepatide_medium_dosage	0	-0.297949036	NA	-0.297949036	NA	NA	NA	NA
50	Canagliflozin_high_dosage:Canagliflozin_low_dosage	2	0.442177889	0.628956128	-0.096346704	0.725302832	-1.840165543	3.290771208	0.579499366
51	Canagliflozin_high_dosage:Dapagliflozin_high_dosage	0	1.040783851	NA	1.040783851	NA	NA	NA	NA
52	Canagliflozin_high_dosage:Dapagliflozin_low_dosage	0	0.05876504	NA	0.05876504	NA	NA	NA	NA
53	Canagliflozin_high_dosage:Dapagliflozin_medium_dosage	0	1.210529936	NA	1.210529936	NA	NA	NA	NA
54	Canagliflozin_high_dosage:Dulaglutide	0	1.833501852	NA	1.833501852	NA	NA	NA	NA
55	Canagliflozin_high_dosage:Efpeglenatide_high_dosage	0	0.130918171	NA	0.130918171	NA	NA	NA	NA
56	Canagliflozin_high_dosage:Efpeglenatide_medium_dosage	0	0.538594854	NA	0.538594854	NA	NA	NA	NA
57	Canagliflozin_high_dosage:Empagliflozin_high_dosage	0	1.011955313	NA	1.011955313	NA	NA	NA	NA
58	Canagliflozin_high_dosage:Empagliflozin_low_dosage	0	1.088802296	NA	1.088802296	NA	NA	NA	NA
59	Canagliflozin_high_dosage:Ertugliflozin_high_dosage	0	1.453030022	NA	1.453030022	NA	NA	NA	NA
60	Canagliflozin_high_dosage:Ertugliflozin_low_dosage	0	2.146541701	NA	2.146541701	NA	NA	NA	NA
61	Canagliflozin_high_dosage:Exenatide	0	1.224979539	NA	1.224979539	NA	NA	NA	NA
62	Canagliflozin_high_dosage:Inject_semaglutide_high_dosage	0	1.280070786	NA	1.280070786	NA	NA	NA	NA
63	Canagliflozin_high_dosage:Inject_semaglutide_low_dosage	0	0.833493333	NA	0.833493333	NA	NA	NA	NA

64	Canagliflozin_high_dosage:Inject_semaglutide_medium_dosage	1	1.185977082	1.096067758	1.211729442	-0.115661684	-3.749276233	3.517952866	0.950254116
65	Canagliflozin_high_dosage:Liraglutide	0	2.048738542	NA	2.048738542	NA	NA	NA	NA
66	Canagliflozin_high_dosage:Lixisenatide	0	2.327404468	NA	2.327404468	NA	NA	NA	NA
67	Canagliflozin_high_dosage:Oral_semaglutide	0	-0.086656976	NA	-0.086656976	NA	NA	NA	NA
68	Canagliflozin_high_dosage:Placebo_or_Control	3	1.228792179	1.077244545	1.465541213	-0.388296668	-2.740469488	1.963876152	0.746278114
69	Canagliflozin_high_dosage:Sotagliflozin	0	1.274836613	NA	1.274836613	NA	NA	NA	NA
70	Canagliflozin_high_dosage:Tirzepatide_high_dosage	0	2.308550807	NA	2.308550807	NA	NA	NA	NA
71	Canagliflozin_high_dosage:Tirzepatide_low_dosage	0	2.308550807	NA	2.308550807	NA	NA	NA	NA
72	Canagliflozin_high_dosage:Tirzepatide_medium_dosage	0	2.318022069	NA	2.318022069	NA	NA	NA	NA
73	Canagliflozin_low_dosage:Dapagliflozin_high_dosage	0	0.598605962	NA	0.598605962	NA	NA	NA	NA
74	Canagliflozin_low_dosage:Dapagliflozin_low_dosage	0	-0.383412848	NA	-0.383412848	NA	NA	NA	NA
75	Canagliflozin_low_dosage:Dapagliflozin_medium_dosage	0	0.768352047	NA	0.768352047	NA	NA	NA	NA
76	Canagliflozin_low_dosage:Dulaglutide	0	1.391323963	NA	1.391323963	NA	NA	NA	NA
77	Canagliflozin_low_dosage:Efpeglenatide_high_dosage	0	-0.311259718	NA	-0.311259718	NA	NA	NA	NA
78	Canagliflozin_low_dosage:Efpeglenatide_medium_dosage	0	0.096416965	NA	0.096416965	NA	NA	NA	NA
79	Canagliflozin_low_dosage:Empagliflozin_high_dosage	0	0.569777424	NA	0.569777424	NA	NA	NA	NA
80	Canagliflozin_low_dosage:Empagliflozin_low_dosage	0	0.646624407	NA	0.646624407	NA	NA	NA	NA
81	Canagliflozin_low_dosage:Ertugliflozin_high_dosage	0	1.010852134	NA	1.010852134	NA	NA	NA	NA
82	Canagliflozin_low_dosage:Ertugliflozin_low_dosage	0	1.704363812	NA	1.704363812	NA	NA	NA	NA
83	Canagliflozin_low_dosage:Exenatide	0	0.78280165	NA	0.78280165	NA	NA	NA	NA
84	Canagliflozin_low_dosage:Inject_semaglutide_high_dosage	0	0.837892897	NA	0.837892897	NA	NA	NA	NA
85	Canagliflozin_low_dosage:Inject_semaglutide_low_dosage	0	0.391315444	NA	0.391315444	NA	NA	NA	NA

86	Canagliflozin_low_dosage:Inject_semaglutide_medium_dosage	0	0.743799193	NA	0.743799193	NA	NA	NA	NA
87	Canagliflozin_low_dosage:Liraglutide	0	1.606560653	NA	1.606560653	NA	NA	NA	NA
88	Canagliflozin_low_dosage:Lixisenatide	0	1.885226579	NA	1.885226579	NA	NA	NA	NA
89	Canagliflozin_low_dosage:Oral_semaglutide	0	-0.528834865	NA	-0.528834865	NA	NA	NA	NA
90	Canagliflozin_low_dosage:Placebo_or_Control	3	0.786614291	0.905290104	-0.037637622	0.942927726	-2.199021282	4.084876735	0.556395926
91	Canagliflozin_low_dosage:Sotagliflozin	0	0.832658724	NA	0.832658724	NA	NA	NA	NA
92	Canagliflozin_low_dosage:Tirzepatide_high_dosage	0	1.866372918	NA	1.866372918	NA	NA	NA	NA
93	Canagliflozin_low_dosage:Tirzepatide_low_dosage	0	1.866372918	NA	1.866372918	NA	NA	NA	NA
94	Canagliflozin_low_dosage:Tirzepatide_medium_dosage	0	1.875844181	NA	1.875844181	NA	NA	NA	NA
95	Dapagliflozin_high_dosage:Dapagliflozin_low_dosage	1	-0.982018811	-1.073485339	-0.887646635	-0.185838704	-4.756577763	4.384900356	0.936484688
96	Dapagliflozin_high_dosage:Dapagliflozin_medium_dosage	0	0.169746085	NA	0.169746085	NA	NA	NA	NA
97	Dapagliflozin_high_dosage:Dulaglutide	0	0.792718001	NA	0.792718001	NA	NA	NA	NA
98	Dapagliflozin_high_dosage:Efpeglenatide_high_dosage	0	-0.90986568	NA	-0.90986568	NA	NA	NA	NA
99	Dapagliflozin_high_dosage:Efpeglenatide_medium_dosage	0	-0.502188997	NA	-0.502188997	NA	NA	NA	NA
100	Dapagliflozin_high_dosage:Empagliflozin_high_dosage	0	-0.028828538	NA	-0.028828538	NA	NA	NA	NA
101	Dapagliflozin_high_dosage:Empagliflozin_low_dosage	0	0.048018445	NA	0.048018445	NA	NA	NA	NA
102	Dapagliflozin_high_dosage:Ertugliflozin_high_dosage	0	0.412246171	NA	0.412246171	NA	NA	NA	NA
103	Dapagliflozin_high_dosage:Ertugliflozin_low_dosage	0	1.10575785	NA	1.10575785	NA	NA	NA	NA
104	Dapagliflozin_high_dosage:Exenatide	0	0.184195688	NA	0.184195688	NA	NA	NA	NA
105	Dapagliflozin_high_dosage:Inject_semaglutide_high_dosage	0	0.239286935	NA	0.239286935	NA	NA	NA	NA
106	Dapagliflozin_high_dosage:Inject_semaglutide_low_dosage	0	-0.207290518	NA	-0.207290518	NA	NA	NA	NA
107	Dapagliflozin_high_dosage:Inject_semaglutide_medium_dosage	0	0.145193231	NA	0.145193231	NA	NA	NA	NA

108	Dapagliflozin_high_dosage:Liraglutide	0	1.007954691	NA	1.007954691	NA	NA	NA	NA
109	Dapagliflozin_high_dosage:Lixisenatide	0	1.286620617	NA	1.286620617	NA	NA	NA	NA
110	Dapagliflozin_high_dosage:Oral_semaglutide	0	-1.127440827	NA	-1.127440827	NA	NA	NA	NA
111	Dapagliflozin_high_dosage:Placebo_or_Control	4	0.188008328	0.190914857	0.005076153	0.185838704	-4.384900356	4.756577763	0.936484688
112	Dapagliflozin_high_dosage:Sotagliflozin	0	0.234052762	NA	0.234052762	NA	NA	NA	NA
113	Dapagliflozin_high_dosage:Tirzepatide_high_dosage	0	1.267766956	NA	1.267766956	NA	NA	NA	NA
114	Dapagliflozin_high_dosage:Tirzepatide_low_dosage	0	1.267766956	NA	1.267766956	NA	NA	NA	NA
115	Dapagliflozin_high_dosage:Tirzepatide_medium_dosage	0	1.277238218	NA	1.277238218	NA	NA	NA	NA
116	Dapagliflozin_low_dosage:Dapagliflozin_medium_dosage	1	1.151764896	1.151764896	NA	NA	NA	NA	NA
117	Dapagliflozin_low_dosage:Dulaglutide	0	1.774736812	NA	1.774736812	NA	NA	NA	NA
118	Dapagliflozin_low_dosage:Efpeglenatide_high_dosage	0	0.07215313	NA	0.07215313	NA	NA	NA	NA
119	Dapagliflozin_low_dosage:Efpeglenatide_medium_dosage	0	0.479829813	NA	0.479829813	NA	NA	NA	NA
120	Dapagliflozin_low_dosage:Empagliflozin_high_dosage	0	0.953190272	NA	0.953190272	NA	NA	NA	NA
121	Dapagliflozin_low_dosage:Empagliflozin_low_dosage	0	1.030037256	NA	1.030037256	NA	NA	NA	NA
122	Dapagliflozin_low_dosage:Ertugliflozin_high_dosage	0	1.394264982	NA	1.394264982	NA	NA	NA	NA
123	Dapagliflozin_low_dosage:Ertugliflozin_low_dosage	0	2.08777666	NA	2.08777666	NA	NA	NA	NA
124	Dapagliflozin_low_dosage:Exenatide	0	1.166214498	NA	1.166214498	NA	NA	NA	NA
125	Dapagliflozin_low_dosage:Inject_semaglutide_high_dosage	0	1.221305745	NA	1.221305745	NA	NA	NA	NA
126	Dapagliflozin_low_dosage:Inject_semaglutide_low_dosage	0	0.774728292	NA	0.774728292	NA	NA	NA	NA
127	Dapagliflozin_low_dosage:Inject_semaglutide_medium_dosage	0	1.127212041	NA	1.127212041	NA	NA	NA	NA
128	Dapagliflozin_low_dosage:Liraglutide	0	1.989973501	NA	1.989973501	NA	NA	NA	NA
129	Dapagliflozin_low_dosage:Lixisenatide	0	2.268639428	NA	2.268639428	NA	NA	NA	NA

130	Dapagliflozin_low_dosage:Oral_semaglutide	0	-0.145422016	NA	-0.145422016	NA	NA	NA	NA
131	Dapagliflozin_low_dosage:Placebo_or_Control	1	1.170027139	1.078561492	1.264400195	-0.185838704	-4.756577763	4.384900356	0.936484688
132	Dapagliflozin_low_dosage:Sotagliflozin	0	1.216071573	NA	1.216071573	NA	NA	NA	NA
133	Dapagliflozin_low_dosage:Tirzepatide_high_dosage	0	2.249785766	NA	2.249785766	NA	NA	NA	NA
134	Dapagliflozin_low_dosage:Tirzepatide_low_dosage	0	2.249785766	NA	2.249785766	NA	NA	NA	NA
135	Dapagliflozin_low_dosage:Tirzepatide_medium_dosage	0	2.259257029	NA	2.259257029	NA	NA	NA	NA
136	Dapagliflozin_medium_dosage:Dulaglutide	0	0.622971916	NA	0.622971916	NA	NA	NA	NA
137	Dapagliflozin_medium_dosage:Efpeglenatide_high_dosage	0	-1.079611765	NA	-1.079611765	NA	NA	NA	NA
138	Dapagliflozin_medium_dosage:Efpeglenatide_medium_dosage	0	-0.671935082	NA	-0.671935082	NA	NA	NA	NA
139	Dapagliflozin_medium_dosage:Empagliflozin_high_dosage	0	-0.198574623	NA	-0.198574623	NA	NA	NA	NA
140	Dapagliflozin_medium_dosage:Empagliflozin_low_dosage	0	-0.12172764	NA	-0.12172764	NA	NA	NA	NA
141	Dapagliflozin_medium_dosage:Ertugliflozin_high_dosage	0	0.242500086	NA	0.242500086	NA	NA	NA	NA
142	Dapagliflozin_medium_dosage:Ertugliflozin_low_dosage	0	0.936011765	NA	0.936011765	NA	NA	NA	NA
143	Dapagliflozin_medium_dosage:Exenatide	0	0.014449603	NA	0.014449603	NA	NA	NA	NA
144	Dapagliflozin_medium_dosage:Inject_semaglutide_high_dosage	0	0.06954085	NA	0.06954085	NA	NA	NA	NA
145	Dapagliflozin_medium_dosage:Inject_semaglutide_low_dosage	0	-0.377036603	NA	-0.377036603	NA	NA	NA	NA
146	Dapagliflozin_medium_dosage:Inject_semaglutide_medium_dosage	0	-0.024552854	NA	-0.024552854	NA	NA	NA	NA
147	Dapagliflozin_medium_dosage:Liraglutide	0	0.838208606	NA	0.838208606	NA	NA	NA	NA
148	Dapagliflozin_medium_dosage:Lixisenatide	0	1.116874532	NA	1.116874532	NA	NA	NA	NA
149	Dapagliflozin_medium_dosage:Oral_semaglutide	0	-1.297186912	NA	-1.297186912	NA	NA	NA	NA
150	Dapagliflozin_medium_dosage:Placebo_or_Control	0	0.018262243	NA	0.018262243	NA	NA	NA	NA
151	Dapagliflozin_medium_dosage:Sotagliflozin	0	0.064306677	NA	0.064306677	NA	NA	NA	NA

152	Dapagliflozin_medium_dosage:Tirzepatide_high_dosage	0	1.098020871	NA	1.098020871	NA	NA	NA	NA
153	Dapagliflozin_medium_dosage:Tirzepatide_low_dosage	0	1.098020871	NA	1.098020871	NA	NA	NA	NA
154	Dapagliflozin_medium_dosage:Tirzepatide_medium_dosage	0	1.107492133	NA	1.107492133	NA	NA	NA	NA
155	Dulaglutide:Efpeglenatide_high_dosage	0	-1.702583681	NA	-1.702583681	NA	NA	NA	NA
156	Dulaglutide:Efpeglenatide_medium_dosage	0	-1.294906998	NA	-1.294906998	NA	NA	NA	NA
157	Dulaglutide:Empagliflozin_high_dosage	0	-0.821546539	NA	-0.821546539	NA	NA	NA	NA
158	Dulaglutide:Empagliflozin_low_dosage	0	-0.744699556	NA	-0.744699556	NA	NA	NA	NA
159	Dulaglutide:Ertugliflozin_high_dosage	0	-0.38047183	NA	-0.38047183	NA	NA	NA	NA
160	Dulaglutide:Ertugliflozin_low_dosage	0	0.313039849	NA	0.313039849	NA	NA	NA	NA
161	Dulaglutide:Exenatide	0	-0.608522313	NA	-0.608522313	NA	NA	NA	NA
162	Dulaglutide:Inject_semaglutide_high_dosage	0	-0.553431066	NA	-0.553431066	NA	NA	NA	NA
163	Dulaglutide:Inject_semaglutide_low_dosage	0	-1.000008519	NA	-1.000008519	NA	NA	NA	NA
164	Dulaglutide:Inject_semaglutide_medium_dosage	0	-0.64752477	NA	-0.64752477	NA	NA	NA	NA
165	Dulaglutide:Liraglutide	0	0.21523669	NA	0.21523669	NA	NA	NA	NA
166	Dulaglutide:Lixisenatide	0	0.493902616	NA	0.493902616	NA	NA	NA	NA
167	Dulaglutide:Oral_semaglutide	0	-1.920158828	NA	-1.920158828	NA	NA	NA	NA
168	Dulaglutide:Placebo_or_Control	3	-0.604709673	-0.604709673	NA	NA	NA	NA	NA
169	Dulaglutide:Sotagliflozin	0	-0.558665239	NA	-0.558665239	NA	NA	NA	NA
170	Dulaglutide:Tirzepatide_high_dosage	0	0.475048955	NA	0.475048955	NA	NA	NA	NA
171	Dulaglutide:Tirzepatide_low_dosage	0	0.475048955	NA	0.475048955	NA	NA	NA	NA
172	Dulaglutide:Tirzepatide_medium_dosage	0	0.484520217	NA	0.484520217	NA	NA	NA	NA
173	Efpeglenatide_high_dosage:Efpeglenatide_medium_dosage	1	0.407676683	0.407676683	NA	NA	NA	NA	NA

174	Efpeglenatide_high_dosage:Empagliflozin_high_dosage	0	0.881037142	NA	0.881037142	NA	NA	NA	NA
175	Efpeglenatide_high_dosage:Empagliflozin_low_dosage	0	0.957884125	NA	0.957884125	NA	NA	NA	NA
176	Efpeglenatide_high_dosage:Ertugliflozin_high_dosage	0	1.322111852	NA	1.322111852	NA	NA	NA	NA
177	Efpeglenatide_high_dosage:Ertugliflozin_low_dosage	0	2.01562353	NA	2.01562353	NA	NA	NA	NA
178	Efpeglenatide_high_dosage:Exenatide	0	1.094061368	NA	1.094061368	NA	NA	NA	NA
179	Efpeglenatide_high_dosage:Inject_semaglutide_high_dosage	0	1.149152615	NA	1.149152615	NA	NA	NA	NA
180	Efpeglenatide_high_dosage:Inject_semaglutide_low_dosage	0	0.702575162	NA	0.702575162	NA	NA	NA	NA
181	Efpeglenatide_high_dosage:Inject_semaglutide_medium_dosage	0	1.055058911	NA	1.055058911	NA	NA	NA	NA
182	Efpeglenatide_high_dosage:Liraglutide	0	1.917820371	NA	1.917820371	NA	NA	NA	NA
183	Efpeglenatide_high_dosage:Lixisenatide	0	2.196486297	NA	2.196486297	NA	NA	NA	NA
184	Efpeglenatide_high_dosage:Oral_semaglutide	0	-0.217575147	NA	-0.217575147	NA	NA	NA	NA
185	Efpeglenatide_high_dosage:Placebo_or_Control	1	1.097874009	1.097874009	NA	NA	NA	NA	NA
186	Efpeglenatide_high_dosage:Sotagliflozin	0	1.143918442	NA	1.143918442	NA	NA	NA	NA
187	Efpeglenatide_high_dosage:Tirzepatide_high_dosage	0	2.177632636	NA	2.177632636	NA	NA	NA	NA
188	Efpeglenatide_high_dosage:Tirzepatide_low_dosage	0	2.177632636	NA	2.177632636	NA	NA	NA	NA
189	Efpeglenatide_high_dosage:Tirzepatide_medium_dosage	0	2.187103899	NA	2.187103899	NA	NA	NA	NA
190	Efpeglenatide_medium_dosage:Empagliflozin_high_dosage	0	0.473360459	NA	0.473360459	NA	NA	NA	NA
191	Efpeglenatide_medium_dosage:Empagliflozin_low_dosage	0	0.550207443	NA	0.550207443	NA	NA	NA	NA
192	Efpeglenatide_medium_dosage:Ertugliflozin_high_dosage	0	0.914435169	NA	0.914435169	NA	NA	NA	NA
193	Efpeglenatide_medium_dosage:Ertugliflozin_low_dosage	0	1.607946847	NA	1.607946847	NA	NA	NA	NA
194	Efpeglenatide_medium_dosage:Exenatide	0	0.686384685	NA	0.686384685	NA	NA	NA	NA
195	Efpeglenatide_medium_dosage:Inject_semaglutide_high_dosage	0	0.741475932	NA	0.741475932	NA	NA	NA	NA

196	Efpeglenatide_medium_dosage:Inject_semaglutide_low_dosage	0	0.294898479	NA	0.294898479	NA	NA	NA	NA
197	Efpeglenatide_medium_dosage:Inject_semaglutide_medium_dosage	0	0.647382228	NA	0.647382228	NA	NA	NA	NA
198	Efpeglenatide_medium_dosage:Liraglutide	0	1.510143688	NA	1.510143688	NA	NA	NA	NA
199	Efpeglenatide_medium_dosage:Lixisenatide	0	1.788809615	NA	1.788809615	NA	NA	NA	NA
200	Efpeglenatide_medium_dosage:Oral_semaglutide	0	-0.62525183	NA	-0.62525183	NA	NA	NA	NA
201	Efpeglenatide_medium_dosage:Placebo_or_Control	1	0.690197326	0.690197326	NA	NA	NA	NA	NA
202	Efpeglenatide_medium_dosage:Sotagliflozin	0	0.736241759	NA	0.736241759	NA	NA	NA	NA
203	Efpeglenatide_medium_dosage:Tirzepatide_high_dosage	0	1.769955953	NA	1.769955953	NA	NA	NA	NA
204	Efpeglenatide_medium_dosage:Tirzepatide_low_dosage	0	1.769955953	NA	1.769955953	NA	NA	NA	NA
205	Efpeglenatide_medium_dosage:Tirzepatide_medium_dosage	0	1.779427216	NA	1.779427216	NA	NA	NA	NA
206	Empagliflozin_high_dosage:Empagliflozin_low_dosage	2	0.076846983	0.180777994	-0.134210771	0.314988765	-1.804722608	2.434700138	0.770859873
207	Empagliflozin_high_dosage:Ertugliflozin_high_dosage	0	0.44107471	NA	0.44107471	NA	NA	NA	NA
208	Empagliflozin_high_dosage:Ertugliflozin_low_dosage	0	1.134586388	NA	1.134586388	NA	NA	NA	NA
209	Empagliflozin_high_dosage:Exenatide	0	0.213024226	NA	0.213024226	NA	NA	NA	NA
210	Empagliflozin_high_dosage:Inject_semaglutide_high_dosage	0	0.268115473	NA	0.268115473	NA	NA	NA	NA
211	Empagliflozin_high_dosage:Inject_semaglutide_low_dosage	0	-0.17846198	NA	-0.17846198	NA	NA	NA	NA
212	Empagliflozin_high_dosage:Inject_semaglutide_medium_dosage	0	0.174021769	NA	0.174021769	NA	NA	NA	NA
213	Empagliflozin_high_dosage:Liraglutide	0	1.036783229	NA	1.036783229	NA	NA	NA	NA
214	Empagliflozin_high_dosage:Lixisenatide	0	1.315449155	NA	1.315449155	NA	NA	NA	NA
215	Empagliflozin_high_dosage:Oral_semaglutide	1	-1.098612289	-1.098612289	NA	NA	NA	NA	NA
216	Empagliflozin_high_dosage:Placebo_or_Control	3	0.216836867	0.092642545	0.80337523	-0.710732685	-3.227867128	1.806401758	0.579981852
217	Empagliflozin_high_dosage:Sotagliflozin	0	0.2628813	NA	0.2628813	NA	NA	NA	NA

218	Empagliflozin_high_dosage:Tirzepatide_high_dosage	0	1.296595494	NA	1.296595494	NA	NA	NA	NA
219	Empagliflozin_high_dosage:Tirzepatide_low_dosage	0	1.296595494	NA	1.296595494	NA	NA	NA	NA
220	Empagliflozin_high_dosage:Tirzepatide_medium_dosage	0	1.306066757	NA	1.306066757	NA	NA	NA	NA
221	Empagliflozin_low_dosage:Ertugliflozin_high_dosage	0	0.364227726	NA	0.364227726	NA	NA	NA	NA
222	Empagliflozin_low_dosage:Ertugliflozin_low_dosage	0	1.057739405	NA	1.057739405	NA	NA	NA	NA
223	Empagliflozin_low_dosage:Exenatide	0	0.136177243	NA	0.136177243	NA	NA	NA	NA
224	Empagliflozin_low_dosage:Inject_semaglutide_high_dosage	0	0.19126849	NA	0.19126849	NA	NA	NA	NA
225	Empagliflozin_low_dosage:Inject_semaglutide_low_dosage	0	-0.255308963	NA	-0.255308963	NA	NA	NA	NA
226	Empagliflozin_low_dosage:Inject_semaglutide_medium_dosage	0	0.097174786	NA	0.097174786	NA	NA	NA	NA
227	Empagliflozin_low_dosage:Liraglutide	0	0.959936246	NA	0.959936246	NA	NA	NA	NA
228	Empagliflozin_low_dosage:Lixisenatide	0	1.238602172	NA	1.238602172	NA	NA	NA	NA
229	Empagliflozin_low_dosage:Oral_semaglutide	0	-1.175459272	NA	-1.175459272	NA	NA	NA	NA
230	Empagliflozin_low_dosage:Placebo_or_Control	5	0.139989883	0.112547472	1.335245216	-1.222697744	-5.164799345	2.719403857	0.543247073
231	Empagliflozin_low_dosage:Sotagliflozin	0	0.186034317	NA	0.186034317	NA	NA	NA	NA
232	Empagliflozin_low_dosage:Tirzepatide_high_dosage	0	1.219748511	NA	1.219748511	NA	NA	NA	NA
233	Empagliflozin_low_dosage:Tirzepatide_low_dosage	0	1.219748511	NA	1.219748511	NA	NA	NA	NA
234	Empagliflozin_low_dosage:Tirzepatide_medium_dosage	0	1.229219773	NA	1.229219773	NA	NA	NA	NA
235	Ertugliflozin_high_dosage:Ertugliflozin_low_dosage	1	0.693511678	0.693511678	NA	NA	NA	NA	NA
236	Ertugliflozin_high_dosage:Exenatide	0	-0.228050484	NA	-0.228050484	NA	NA	NA	NA
237	Ertugliflozin_high_dosage:Inject_semaglutide_high_dosage	0	-0.172959237	NA	-0.172959237	NA	NA	NA	NA
238	Ertugliflozin_high_dosage:Inject_semaglutide_low_dosage	0	-0.61953669	NA	-0.61953669	NA	NA	NA	NA
239	Ertugliflozin_high_dosage:Inject_semaglutide_medium_dosage	0	-0.267052941	NA	-0.267052941	NA	NA	NA	NA

240	Ertugliflozin_high_dosage:Liraglutide	0	0.595708519	NA	0.595708519	NA	NA	NA	NA
241	Ertugliflozin_high_dosage:Lixisenatide	0	0.874374446	NA	0.874374446	NA	NA	NA	NA
242	Ertugliflozin_high_dosage:Oral_semaglutide	0	-1.539686998	NA	-1.539686998	NA	NA	NA	NA
243	Ertugliflozin_high_dosage:Placebo_or_Control	1	-0.224237843	-0.224237843	NA	NA	NA	NA	NA
244	Ertugliflozin_high_dosage:Sotagliflozin	0	-0.178193409	NA	-0.178193409	NA	NA	NA	NA
245	Ertugliflozin_high_dosage:Tirzepatide_high_dosage	0	0.855520784	NA	0.855520784	NA	NA	NA	NA
246	Ertugliflozin_high_dosage:Tirzepatide_low_dosage	0	0.855520784	NA	0.855520784	NA	NA	NA	NA
247	Ertugliflozin_high_dosage:Tirzepatide_medium_dosage	0	0.864992047	NA	0.864992047	NA	NA	NA	NA
248	Ertugliflozin_low_dosage:Exenatide	0	-0.921562162	NA	-0.921562162	NA	NA	NA	NA
249	Ertugliflozin_low_dosage:Inject_semaglutide_high_dosage	0	-0.866470915	NA	-0.866470915	NA	NA	NA	NA
250	Ertugliflozin_low_dosage:Inject_semaglutide_low_dosage	0	-1.313048368	NA	-1.313048368	NA	NA	NA	NA
251	Ertugliflozin_low_dosage:Inject_semaglutide_medium_dosage	0	-0.960564619	NA	-0.960564619	NA	NA	NA	NA
252	Ertugliflozin_low_dosage:Liraglutide	0	-0.097803159	NA	-0.097803159	NA	NA	NA	NA
253	Ertugliflozin_low_dosage:Lixisenatide	0	0.180862767	NA	0.180862767	NA	NA	NA	NA
254	Ertugliflozin_low_dosage:Oral_semaglutide	0	-2.233198677	NA	-2.233198677	NA	NA	NA	NA
255	Ertugliflozin_low_dosage:Placebo_or_Control	1	-0.917749521	-0.917749521	NA	NA	NA	NA	NA
256	Ertugliflozin_low_dosage:Sotagliflozin	0	-0.871705088	NA	-0.871705088	NA	NA	NA	NA
257	Ertugliflozin_low_dosage:Tirzepatide_high_dosage	0	0.162009106	NA	0.162009106	NA	NA	NA	NA
258	Ertugliflozin_low_dosage:Tirzepatide_low_dosage	0	0.162009106	NA	0.162009106	NA	NA	NA	NA
259	Ertugliflozin_low_dosage:Tirzepatide_medium_dosage	0	0.171480369	NA	0.171480369	NA	NA	NA	NA
260	Exenatide:Inject_semaglutide_high_dosage	0	0.055091247	NA	0.055091247	NA	NA	NA	NA
261	Exenatide:Inject_semaglutide_low_dosage	0	-0.391486206	NA	-0.391486206	NA	NA	NA	NA

262	Exenatide:Inject_semaglutide_medium_dosage	0	-0.039002457	NA	-0.039002457	NA	NA	NA	NA
263	Exenatide:Liraglutide	0	0.823759003	NA	0.823759003	NA	NA	NA	NA
264	Exenatide:Lixisenatide	0	1.102424929	NA	1.102424929	NA	NA	NA	NA
265	Exenatide:Oral_semaglutide	0	-1.311636515	NA	-1.311636515	NA	NA	NA	NA
266	Exenatide:Placebo_or_Control	1	0.003812641	0.003812641	NA	NA	NA	NA	NA
267	Exenatide:Sotagliflozin	0	0.049857074	NA	0.049857074	NA	NA	NA	NA
268	Exenatide:Tirzepatide_high_dosage	0	1.083571268	NA	1.083571268	NA	NA	NA	NA
269	Exenatide:Tirzepatide_low_dosage	0	1.083571268	NA	1.083571268	NA	NA	NA	NA
270	Exenatide:Tirzepatide_medium_dosage	0	1.093042531	NA	1.093042531	NA	NA	NA	NA
271	Inject_semaglutide_high_dosage:Inject_semaglutide_low_dosage	0	-0.446577453	NA	-0.446577453	NA	NA	NA	NA
272	Inject_semaglutide_high_dosage:Inject_semaglutide_medium_dosage	1	-0.094093704	1.098612289	-0.300862976	1.399475265	-2.070709635	4.869660165	0.42927957
273	Inject_semaglutide_high_dosage:Liraglutide	0	0.768667756	NA	0.768667756	NA	NA	NA	NA
274	Inject_semaglutide_high_dosage:Lixisenatide	0	1.047333682	NA	1.047333682	NA	NA	NA	NA
275	Inject_semaglutide_high_dosage:Oral_semaglutide	0	-1.366727762	NA	-1.366727762	NA	NA	NA	NA
276	Inject_semaglutide_high_dosage:Placebo_or_Control	5	-0.051278606	-0.08640009	1.313075175	-1.399475265	-4.869660165	2.070709635	0.42927957
277	Inject_semaglutide_high_dosage:Sotagliflozin	0	-0.005234173	NA	-0.005234173	NA	NA	NA	NA
278	Inject_semaglutide_high_dosage:Tirzepatide_high_dosage	0	1.028480021	NA	1.028480021	NA	NA	NA	NA
279	Inject_semaglutide_high_dosage:Tirzepatide_low_dosage	0	1.028480021	NA	1.028480021	NA	NA	NA	NA
280	Inject_semaglutide_high_dosage:Tirzepatide_medium_dosage	0	1.037951284	NA	1.037951284	NA	NA	NA	NA
281	Inject_semaglutide_low_dosage:Inject_semaglutide_medium_dosage	4	0.352483749	0.253360394	0.639427433	-0.386067039	-3.423439295	2.651305217	0.803266096
282	Inject_semaglutide_low_dosage:Liraglutide	0	1.215245209	NA	1.215245209	NA	NA	NA	NA
283	Inject_semaglutide_low_dosage:Lixisenatide	0	1.493911136	NA	1.493911136	NA	NA	NA	NA

284	Inject_semaglutide_low_dosage:Oral_semaglutide	0	-0.920150309	NA	-0.920150309	NA	NA	NA	NA
285	Inject_semaglutide_low_dosage:Placebo_or_Control	3	0.395298847	0.399134267	0.387493531	0.011640736	-2.86052553	2.883807002	0.993661968
286	Inject_semaglutide_low_dosage:Sotagliflozin	0	0.44134328	NA	0.44134328	NA	NA	NA	NA
287	Inject_semaglutide_low_dosage:Tirzepatide_high_dosage	0	1.475057474	NA	1.475057474	NA	NA	NA	NA
288	Inject_semaglutide_low_dosage:Tirzepatide_low_dosage	0	1.475057474	NA	1.475057474	NA	NA	NA	NA
289	Inject_semaglutide_low_dosage:Tirzepatide_medium_dosage	0	1.484528737	NA	1.484528737	NA	NA	NA	NA
290	Inject_semaglutide_medium_dosage:Liraglutide	0	0.86276146	NA	0.86276146	NA	NA	NA	NA
291	Inject_semaglutide_medium_dosage:Lixisenatide	0	1.141427386	NA	1.141427386	NA	NA	NA	NA
292	Inject_semaglutide_medium_dosage:Oral_semaglutide	0	-1.272634058	NA	-1.272634058	NA	NA	NA	NA
293	Inject_semaglutide_medium_dosage:Placebo_or_Control	4	0.042815098	0.230067853	-0.241399979	0.471467832	-1.854959922	2.797895587	0.691219073
294	Inject_semaglutide_medium_dosage:Sotagliflozin	0	0.088859531	NA	0.088859531	NA	NA	NA	NA
295	Inject_semaglutide_medium_dosage:Tirzepatide_high_dosage	0	1.122573725	NA	1.122573725	NA	NA	NA	NA
296	Inject_semaglutide_medium_dosage:Tirzepatide_low_dosage	0	1.122573725	NA	1.122573725	NA	NA	NA	NA
297	Inject_semaglutide_medium_dosage:Tirzepatide_medium_dosage	0	1.132044988	NA	1.132044988	NA	NA	NA	NA
298	Liraglutide:Lixisenatide	0	0.278665926	NA	0.278665926	NA	NA	NA	NA
299	Liraglutide:Oral_semaglutide	0	-2.135395518	NA	-2.135395518	NA	NA	NA	NA
300	Liraglutide:Placebo_or_Control	3	-0.819946362	-0.819946362	NA	NA	NA	NA	NA
301	Liraglutide:Sotagliflozin	0	-0.773901929	NA	-0.773901929	NA	NA	NA	NA
302	Liraglutide:Tirzepatide_high_dosage	0	0.259812265	NA	0.259812265	NA	NA	NA	NA
303	Liraglutide:Tirzepatide_low_dosage	0	0.259812265	NA	0.259812265	NA	NA	NA	NA
304	Liraglutide:Tirzepatide_medium_dosage	0	0.269283528	NA	0.269283528	NA	NA	NA	NA
305	Lixisenatide:Oral_semaglutide	0	-2.414061444	NA	-2.414061444	NA	NA	NA	NA

306	Lixisenatide:Placebo_or_Control	1	-1.098612289	-1.098612289	NA	NA	NA	NA	NA
307	Lixisenatide:Sotagliflozin	0	-1.052567855	NA	-1.052567855	NA	NA	NA	NA
308	Lixisenatide:Tirzepatide_high_dosage	0	-0.018853661	NA	-0.018853661	NA	NA	NA	NA
309	Lixisenatide:Tirzepatide_low_dosage	0	-0.018853661	NA	-0.018853661	NA	NA	NA	NA
310	Lixisenatide:Tirzepatide_medium_dosage	0	-0.009382399	NA	-0.009382399	NA	NA	NA	NA
311	Oral_semaglutide:Placebo_or_Control	0	1.315449155	NA	1.315449155	NA	NA	NA	NA
312	Oral_semaglutide:Sotagliflozin	0	1.361493589	NA	1.361493589	NA	NA	NA	NA
313	Oral_semaglutide:Tirzepatide_high_dosage	0	2.395207783	NA	2.395207783	NA	NA	NA	NA
314	Oral_semaglutide:Tirzepatide_low_dosage	0	2.395207783	NA	2.395207783	NA	NA	NA	NA
315	Oral_semaglutide:Tirzepatide_medium_dosage	0	2.404679045	NA	2.404679045	NA	NA	NA	NA
316	Sotagliflozin:Placebo_or_Control	4	-0.046044433	-0.046044433	NA	NA	NA	NA	NA
317	Tirzepatide_high_dosage:Placebo_or_Control	1	-1.079758627	-1.079758627	NA	NA	NA	NA	NA
318	Tirzepatide_low_dosage:Placebo_or_Control	1	-1.079758627	-1.079758627	NA	NA	NA	NA	NA
319	Tirzepatide_medium_dosage:Placebo_or_Control	1	-1.08922989	-1.08922989	NA	NA	NA	NA	NA
320	Sotagliflozin:Tirzepatide_high_dosage	0	1.033714194	NA	1.033714194	NA	NA	NA	NA
321	Sotagliflozin:Tirzepatide_low_dosage	0	1.033714194	NA	1.033714194	NA	NA	NA	NA
322	Sotagliflozin:Tirzepatide_medium_dosage	0	1.043185456	NA	1.043185456	NA	NA	NA	NA
323	Tirzepatide_high_dosage:Tirzepatide_low_dosage	0	0	NA	0	NA	NA	NA	NA
324	Tirzepatide_high_dosage:Tirzepatide_medium_dosage	0	0.009471263	NA	0.009471263	NA	NA	NA	NA
325	Tirzepatide_low_dosage:Tirzepatide_medium_dosage	0	0.009471263	NA	0.009471263	NA	NA	NA	NA

Table S7C: inconsistency within the network meta-analysis of acceptability: drop-out rate

	Comparison	No.Studies	NMA	Direct	Indirect	Difference	Diff_95CI_lower	Diff_95CI_upper	*p* value
1	Albiglutide:Bexagliflozin	0	-0.214206286	NA	-0.214206286	NA	NA	NA	NA
2	Albiglutide:Canagliflozin	0	0.06632405	NA	0.06632405	NA	NA	NA	NA
3	Albiglutide:Dapagliflozin	0	-0.106001699	NA	-0.106001699	NA	NA	NA	NA
4	Albiglutide:Dulaglutide	0	-0.13325246	NA	-0.13325246	NA	NA	NA	NA
5	Albiglutide:Efpeglenatide	0	-0.323625032	NA	-0.323625032	NA	NA	NA	NA
6	Albiglutide:Empagliflozin	0	-0.202371738	NA	-0.202371738	NA	NA	NA	NA
7	Albiglutide:Ertugliflozin	0	-0.279780415	NA	-0.279780415	NA	NA	NA	NA
8	Albiglutide:Exenatide	0	-0.201112322	NA	-0.201112322	NA	NA	NA	NA
9	Albiglutide:Inject_semaglutide	0	-0.138371925	NA	-0.138371925	NA	NA	NA	NA
10	Albiglutide:Liraglutide	0	-0.077538442	NA	-0.077538442	NA	NA	NA	NA
11	Albiglutide:Lixisenatide	0	-0.298413057	NA	-0.298413057	NA	NA	NA	NA
12	Albiglutide:Oral_semaglutide	0	0.568267059	NA	0.568267059	NA	NA	NA	NA
13	Albiglutide:Placebo_or_Control	4	-0.346641598	-0.346641598	NA	NA	NA	NA	NA
14	Albiglutide:Sotagliflozin	0	-0.192949245	NA	-0.192949245	NA	NA	NA	NA
15	Albiglutide:Tirzepatide	0	0.468853277	NA	0.468853277	NA	NA	NA	NA
16	Bexagliflozin:Canagliflozin	0	0.280530335	NA	0.280530335	NA	NA	NA	NA
17	Bexagliflozin:Dapagliflozin	0	0.108204587	NA	0.108204587	NA	NA	NA	NA
18	Bexagliflozin:Dulaglutide	0	0.080953825	NA	0.080953825	NA	NA	NA	NA
19	Bexagliflozin:Efpeglenatide	0	-0.109418746	NA	-0.109418746	NA	NA	NA	NA
20	Bexagliflozin:Empagliflozin	0	0.011834547	NA	0.011834547	NA	NA	NA	NA

21	Bexagliflozin:Ertugliflozin	0	-0.065574129	NA	-0.065574129	NA	NA	NA	NA
22	Bexagliflozin:Exenatide	0	0.013093963	NA	0.013093963	NA	NA	NA	NA
23	Bexagliflozin:Inject_semaglutide	0	0.07583436	NA	0.07583436	NA	NA	NA	NA
24	Bexagliflozin:Liraglutide	0	0.136667844	NA	0.136667844	NA	NA	NA	NA
25	Bexagliflozin:Lixisenatide	0	-0.084206771	NA	-0.084206771	NA	NA	NA	NA
26	Bexagliflozin:Oral_semaglutide	0	0.782473344	NA	0.782473344	NA	NA	NA	NA
27	Bexagliflozin:Placebo_or_Control	1	-0.132435312	-0.132435312	NA	NA	NA	NA	NA
28	Bexagliflozin:Sotagliflozin	0	0.021257041	NA	0.021257041	NA	NA	NA	NA
29	Bexagliflozin:Tirzepatide	0	0.683059563	NA	0.683059563	NA	NA	NA	NA
30	Canagliflozin:Dapagliflozin	0	-0.172325749	NA	-0.172325749	NA	NA	NA	NA
31	Canagliflozin:Dulaglutide	0	-0.19957651	NA	-0.19957651	NA	NA	NA	NA
32	Canagliflozin:Efpeglenatide	0	-0.389949082	NA	-0.389949082	NA	NA	NA	NA
33	Canagliflozin:Empagliflozin	0	-0.268695788	NA	-0.268695788	NA	NA	NA	NA
34	Canagliflozin:Ertugliflozin	0	-0.346104465	NA	-0.346104465	NA	NA	NA	NA
35	Canagliflozin:Exenatide	0	-0.267436372	NA	-0.267436372	NA	NA	NA	NA
36	Canagliflozin:Inject_semaglutide	1	-0.204695975	-0.218326419	-0.202425464	-0.015900955	-0.666013181	0.634211271	0.961765386
37	Canagliflozin:Liraglutide	0	-0.143862491	NA	-0.143862491	NA	NA	NA	NA
38	Canagliflozin:Lixisenatide	0	-0.364737106	NA	-0.364737106	NA	NA	NA	NA
39	Canagliflozin:Oral_semaglutide	0	0.501943009	NA	0.501943009	NA	NA	NA	NA
40	Canagliflozin:Placebo_or_Control	4	-0.412965648	-0.411560904	-0.427461859	0.015900955	-0.634211271	0.666013181	0.961765386
41	Canagliflozin:Sotagliflozin	0	-0.259273294	NA	-0.259273294	NA	NA	NA	NA
42	Canagliflozin:Tirzepatide	0	0.402529227	NA	0.402529227	NA	NA	NA	NA

43	Dapagliflozin:Dulaglutide	0	-0.027250761	NA	-0.027250761	NA	NA	NA	NA
44	Dapagliflozin:Efpeglenatide	0	-0.217623333	NA	-0.217623333	NA	NA	NA	NA
45	Dapagliflozin:Empagliflozin	0	-0.096370039	NA	-0.096370039	NA	NA	NA	NA
46	Dapagliflozin:Ertugliflozin	0	-0.173778716	NA	-0.173778716	NA	NA	NA	NA
47	Dapagliflozin:Exenatide	0	-0.095110623	NA	-0.095110623	NA	NA	NA	NA
48	Dapagliflozin:Inject_semaglutide	0	-0.032370226	NA	-0.032370226	NA	NA	NA	NA
49	Dapagliflozin:Liraglutide	0	0.028463257	NA	0.028463257	NA	NA	NA	NA
50	Dapagliflozin:Lixisenatide	0	-0.192411358	NA	-0.192411358	NA	NA	NA	NA
51	Dapagliflozin:Oral_semaglutide	0	0.674268758	NA	0.674268758	NA	NA	NA	NA
52	Dapagliflozin:Placebo_or_Control	5	-0.240639899	-0.240639899	NA	NA	NA	NA	NA
53	Dapagliflozin:Sotagliflozin	0	-0.086947546	NA	-0.086947546	NA	NA	NA	NA
54	Dapagliflozin:Tirzepatide	0	0.574854976	NA	0.574854976	NA	NA	NA	NA
55	Dulaglutide:Efpeglenatide	0	-0.190372572	NA	-0.190372572	NA	NA	NA	NA
56	Dulaglutide:Empagliflozin	0	-0.069119278	NA	-0.069119278	NA	NA	NA	NA
57	Dulaglutide:Ertugliflozin	0	-0.146527955	NA	-0.146527955	NA	NA	NA	NA
58	Dulaglutide:Exenatide	0	-0.067859862	NA	-0.067859862	NA	NA	NA	NA
59	Dulaglutide:Inject_semaglutide	0	-0.005119465	NA	-0.005119465	NA	NA	NA	NA
60	Dulaglutide:Liraglutide	0	0.055714019	NA	0.055714019	NA	NA	NA	NA
61	Dulaglutide:Lixisenatide	0	-0.165160596	NA	-0.165160596	NA	NA	NA	NA
62	Dulaglutide:Oral_semaglutide	0	0.701519519	NA	0.701519519	NA	NA	NA	NA
63	Dulaglutide:Placebo_or_Control	3	-0.213389138	-0.213389138	NA	NA	NA	NA	NA
64	Dulaglutide:Sotagliflozin	0	-0.059696784	NA	-0.059696784	NA	NA	NA	NA

65	Dulaglutide:Tirzepatide	0	0.602105737	NA	0.602105737	NA	NA	NA	NA
66	Efpeglenatide:Empagliflozin	0	0.121253294	NA	0.121253294	NA	NA	NA	NA
67	Efpeglenatide:Ertugliflozin	0	0.043844617	NA	0.043844617	NA	NA	NA	NA
68	Efpeglenatide:Exenatide	0	0.12251271	NA	0.12251271	NA	NA	NA	NA
69	Efpeglenatide:Inject_semaglutide	0	0.185253107	NA	0.185253107	NA	NA	NA	NA
70	Efpeglenatide:Liraglutide	0	0.24608659	NA	0.24608659	NA	NA	NA	NA
71	Efpeglenatide:Lixisenatide	0	0.025211975	NA	0.025211975	NA	NA	NA	NA
72	Efpeglenatide:Oral_semaglutide	0	0.891892091	NA	0.891892091	NA	NA	NA	NA
73	Efpeglenatide:Placebo_or_Control	1	-0.023016566	-0.023016566	NA	NA	NA	NA	NA
74	Efpeglenatide:Sotagliflozin	0	0.130675787	NA	0.130675787	NA	NA	NA	NA
75	Efpeglenatide:Tirzepatide	0	0.792478309	NA	0.792478309	NA	NA	NA	NA
76	Empagliflozin:Ertugliflozin	0	-0.077408677	NA	-0.077408677	NA	NA	NA	NA
77	Empagliflozin:Exenatide	0	0.001259416	NA	0.001259416	NA	NA	NA	NA
78	Empagliflozin:Inject_semaglutide	0	0.063999813	NA	0.063999813	NA	NA	NA	NA
79	Empagliflozin:Liraglutide	0	0.124833297	NA	0.124833297	NA	NA	NA	NA
80	Empagliflozin:Lixisenatide	0	-0.096041318	NA	-0.096041318	NA	NA	NA	NA
81	Empagliflozin:Oral_semaglutide	1	0.770638797	0.770638797	NA	NA	NA	NA	NA
82	Empagliflozin:Placebo_or_Control	7	-0.14426986	-0.14426986	NA	NA	NA	NA	NA
83	Empagliflozin:Sotagliflozin	0	0.009422494	NA	0.009422494	NA	NA	NA	NA
84	Empagliflozin:Tirzepatide	0	0.671225015	NA	0.671225015	NA	NA	NA	NA
85	Ertugliflozin:Exenatide	0	0.078668093	NA	0.078668093	NA	NA	NA	NA
86	Ertugliflozin:Inject_semaglutide	0	0.14140849	NA	0.14140849	NA	NA	NA	NA

87	Ertugliflozin:Liraglutide	0	0.202241974	NA	0.202241974	NA	NA	NA	NA
88	Ertugliflozin:Lixisenatide	0	-0.018632642	NA	-0.018632642	NA	NA	NA	NA
89	Ertugliflozin:Oral_semaglutide	0	0.848047474	NA	0.848047474	NA	NA	NA	NA
90	Ertugliflozin:Placebo_or_Control	1	-0.066861183	-0.066861183	NA	NA	NA	NA	NA
91	Ertugliflozin:Sotagliflozin	0	0.08683117	NA	0.08683117	NA	NA	NA	NA
92	Ertugliflozin:Tirzepatide	0	0.748633692	NA	0.748633692	NA	NA	NA	NA
93	Exenatide:Inject_semaglutide	0	0.062740397	NA	0.062740397	NA	NA	NA	NA
94	Exenatide:Liraglutide	0	0.123573881	NA	0.123573881	NA	NA	NA	NA
95	Exenatide:Lixisenatide	0	-0.097300734	NA	-0.097300734	NA	NA	NA	NA
96	Exenatide:Oral_semaglutide	0	0.769379381	NA	0.769379381	NA	NA	NA	NA
97	Exenatide:Placebo_or_Control	1	-0.145529276	-0.145529276	NA	NA	NA	NA	NA
98	Exenatide:Sotagliflozin	0	0.008163078	NA	0.008163078	NA	NA	NA	NA
99	Exenatide:Tirzepatide	0	0.669965599	NA	0.669965599	NA	NA	NA	NA
100	Inject_semaglutide:Liraglutide	0	0.060833484	NA	0.060833484	NA	NA	NA	NA
101	Inject_semaglutide:Lixisenatide	0	-0.160041131	NA	-0.160041131	NA	NA	NA	NA
102	Inject_semaglutide:Oral_semaglutide	0	0.706638984	NA	0.706638984	NA	NA	NA	NA
103	Inject_semaglutide:Placebo_or_Control	10	-0.208269673	-0.20913544	-0.193234485	-0.015900955	-0.666013181	0.634211271	0.961765386
104	Inject_semaglutide:Sotagliflozin	0	-0.054577319	NA	-0.054577319	NA	NA	NA	NA
105	Inject_semaglutide:Tirzepatide	0	0.607225202	NA	0.607225202	NA	NA	NA	NA
106	Liraglutide:Lixisenatide	0	-0.220874615	NA	-0.220874615	NA	NA	NA	NA
107	Liraglutide:Oral_semaglutide	0	0.6458055	NA	0.6458055	NA	NA	NA	NA
108	Liraglutide:Placebo_or_Control	3	-0.269103157	-0.269103157	NA	NA	NA	NA	NA

109	Liraglutide:Sotagliflozin	0	-0.115410803	NA	-0.115410803	NA	NA	NA	NA
110	Liraglutide:Tirzepatide	0	0.546391718	NA	0.546391718	NA	NA	NA	NA
111	Lixisenatide:Oral_semaglutide	0	0.866680115	NA	0.866680115	NA	NA	NA	NA
112	Lixisenatide:Placebo_or_Control	1	-0.048228542	-0.048228542	NA	NA	NA	NA	NA
113	Lixisenatide:Sotagliflozin	0	0.105463812	NA	0.105463812	NA	NA	NA	NA
114	Lixisenatide:Tirzepatide	0	0.767266333	NA	0.767266333	NA	NA	NA	NA
115	Oral_semaglutide:Placebo_or_Control	0	-0.914908657	NA	-0.914908657	NA	NA	NA	NA
116	Oral_semaglutide:Sotagliflozin	0	-0.761216303	NA	-0.761216303	NA	NA	NA	NA
117	Oral_semaglutide:Tirzepatide	0	-0.099413782	NA	-0.099413782	NA	NA	NA	NA
118	Sotagliflozin:Placebo_or_Control	4	-0.153692353	-0.153692353	NA	NA	NA	NA	NA
119	Tirzepatide:Placebo_or_Control	3	-0.815494875	-0.815494875	NA	NA	NA	NA	NA
120	Sotagliflozin:Tirzepatide	0	0.661802522	NA	0.661802522	NA	NA	NA	NA

Abbreviation: 95%CIs: 95% confidence intervals; GLP-1 agonist: glucagon-like peptide-1 agonist; NA: not applicable; NMA: network meta-analysis; OR: odds ratio; RCT: randomized controlled trial; SGLT2 inhibitor: sodium–glucose cotransporter 2 inhibitor

Table S8A: GRADE of primary outcome: intestine obstruction events

	Comparison	No.Studies	Direct		Indirect		NMA	
			Estimate	Rate	Estimate	Rate	Estimate	Rate
1	Albiglutide:Bexagliflozin	0			1.429251017	⊕⊕◯◯ Low	1.429251017	⊕⊕◯◯ Low
2	Albiglutide:Canagliflozin	0			-0.896195645	⊕⊕◯◯ Low	-0.896195645	⊕⊕◯◯ Low
3	Albiglutide:Dapagliflozin	0			-0.142083497	⊕⊕◯◯ Low	-0.142083497	⊕⊕◯◯ Low
4	Albiglutide:Dulaglutide	0			0.646781764	⊕⊕◯◯ Low	0.646781764	⊕⊕◯◯ Low
5	Albiglutide:Efpeglenatide	0			-0.872373962	⊕⊕◯◯ Low	-0.872373962	⊕⊕◯◯ Low
6	Albiglutide:Empagliflozin	0			-0.104755834	⊕⊕◯◯ Low	-0.104755834	⊕⊕◯◯ Low
7	Albiglutide:Ertugliflozin	0			0.554174272	⊕⊕◯◯ Low	0.554174272	⊕⊕◯◯ Low
8	Albiglutide:Exenatide	0			0.038259451	⊕⊕◯◯ Low	0.038259451	⊕⊕◯◯ Low
9	Albiglutide:Inject_semaglutide	0			0.103900509	⊕⊕◯◯ Low	0.103900509	⊕⊕◯◯ Low
10	Albiglutide:Liraglutide	0			0.862018454	⊕⊕◯◯ Low	0.862018454	⊕⊕◯◯ Low
11	Albiglutide:Lixisenatide	0			1.14068438	⊕⊕◯◯ Low	1.14068438	⊕⊕◯◯ Low
12	Albiglutide:Oral_semaglutide	0			-1.203368122	⊕⊕◯◯ Low	-1.203368122	⊕⊕◯◯ Low
13	Albiglutide:Placebo_or_Control	4	0.042072092	⊕⊕⊕⊕ High			0.042072092	⊕⊕⊕◯ Medium
14	Albiglutide:Sotagliflozin	0			0.088116525	⊕⊕◯◯ Low	0.088116525	⊕⊕◯◯ Low
15	Albiglutide:Tirzepatide	0			0.267418523	⊕⊕◯◯ Low	0.267418523	⊕⊕◯◯ Low
16	Bexagliflozin:Canagliflozin	0			-2.325446662	⊕⊕◯◯ Low	-2.325446662	⊕⊕◯◯ Low
17	Bexagliflozin:Dapagliflozin	0			-1.571334514	⊕⊕◯◯ Low	-1.571334514	⊕⊕◯◯ Low
18	Bexagliflozin:Dulaglutide	0			-0.782469253	⊕⊕◯◯ Low	-0.782469253	⊕⊕◯◯ Low

19	Bexagliflozin:Efpeglenatide	0			-2.301624979	⊕⊕◯◯ Low	-2.301624979	⊕⊕◯◯ Low
20	Bexagliflozin:Empagliflozin	0			-1.534006851	⊕⊕◯◯ Low	-1.534006851	⊕⊕◯◯ Low
21	Bexagliflozin:Ertugliflozin	0			-0.875076745	⊕⊕◯◯ Low	-0.875076745	⊕⊕◯◯ Low
22	Bexagliflozin:Exenatide	0			-1.390991566	⊕⊕◯◯ Low	-1.390991566	⊕⊕◯◯ Low
23	Bexagliflozin:Inject_semaglutide	0			-1.325350508	⊕⊕◯◯ Low	-1.325350508	⊕⊕◯◯ Low
24	Bexagliflozin:Liraglutide	0			-0.567232563	⊕⊕◯◯ Low	-0.567232563	⊕⊕◯◯ Low
25	Bexagliflozin:Lixisenatide	0			-0.288566637	⊕⊕◯◯ Low	-0.288566637	⊕⊕◯◯ Low
26	Bexagliflozin:Oral_semaglutide	0			-2.632619139	⊕⊕◯◯ Low	-2.632619139	⊕⊕◯◯ Low
27	Bexagliflozin:Placebo_or_Control	1	-1.387178926	⊕⊕⊕⊕ High			-1.387178926	⊕⊕⊕◯ Medium
28	Bexagliflozin:Sotagliflozin	0			-1.341134492	⊕⊕◯◯ Low	-1.341134492	⊕⊕◯◯ Low
29	Bexagliflozin:Tirzepatide	0			-1.161832494	⊕⊕◯◯ Low	-1.161832494	⊕⊕◯◯ Low
30	Canagliflozin:Dapagliflozin	0			0.754112147	⊕⊕◯◯ Low	0.754112147	⊕⊕◯◯ Low
31	Canagliflozin:Dulaglutide	0			1.542977409	⊕⊕◯◯ Low	1.542977409	⊕⊕◯◯ Low
32	Canagliflozin:Efpeglenatide	0			0.023821683	⊕⊕◯◯ Low	0.023821683	⊕⊕◯◯ Low
33	Canagliflozin:Empagliflozin	0			0.791439811	⊕⊕◯◯ Low	0.791439811	⊕⊕◯◯ Low
34	Canagliflozin:Ertugliflozin	0			1.450369917	⊕⊕◯◯ Low	1.450369917	⊕⊕◯◯ Low
35	Canagliflozin:Exenatide	0			0.934455095	⊕⊕◯◯ Low	0.934455095	⊕⊕◯◯ Low
36	Canagliflozin:Inject_semaglutide	1	1.096067758	⊕⊕⊕⊕ High	0.988874288	⊕⊕⊕⊕ High	1.000096153	⊕⊕⊕⊕ High
37	Canagliflozin:Liraglutide	0			1.758214098	⊕⊕◯◯ Low	1.758214098	⊕⊕◯◯ Low
38	Canagliflozin:Lixisenatide	0			2.036880025	⊕⊕◯◯ Low	2.036880025	⊕⊕◯◯ Low
39	Canagliflozin:Oral_semaglutide	0			-0.307172478	⊕⊕◯◯ Low	-0.307172478	⊕⊕◯◯ Low

40	Canagliflozin:Placebo_or_Control	4	0.929407928	⊕⊕⊕⊕ High	1.036601398	⊕⊕⊕⊕ High	0.938267736	⊕⊕⊕⊕ High
41	Canagliflozin:Sotagliflozin	0			0.98431217	⊕⊕◯◯ Low	0.98431217	⊕⊕◯◯ Low
42	Canagliflozin:Tirzepatide	0			1.163614167	⊕⊕◯◯ Low	1.163614167	⊕⊕◯◯ Low
43	Dapagliflozin:Dulaglutide	0			0.788865261	⊕⊕◯◯ Low	0.788865261	⊕⊕◯◯ Low
44	Dapagliflozin:Efpeglenatide	0			-0.730290465	⊕⊕◯◯ Low	-0.730290465	⊕⊕◯◯ Low
45	Dapagliflozin:Empagliflozin	0			0.037327664	⊕⊕◯◯ Low	0.037327664	⊕⊕◯◯ Low
46	Dapagliflozin:Ertugliflozin	0			0.69625777	⊕⊕◯◯ Low	0.69625777	⊕⊕◯◯ Low
47	Dapagliflozin:Exenatide	0			0.180342948	⊕⊕◯◯ Low	0.180342948	⊕⊕◯◯ Low
48	Dapagliflozin:Inject_semaglutide	0			0.245984006	⊕⊕◯◯ Low	0.245984006	⊕⊕◯◯ Low
49	Dapagliflozin:Liraglutide	0			1.004101951	⊕⊕◯◯ Low	1.004101951	⊕⊕◯◯ Low
50	Dapagliflozin:Lixisenatide	0			1.282767877	⊕⊕◯◯ Low	1.282767877	⊕⊕◯◯ Low
51	Dapagliflozin:Oral_semaglutide	0			-1.061284625	⊕⊕◯◯ Low	-1.061284625	⊕⊕◯◯ Low
52	Dapagliflozin:Placebo_or_Control	5	0.184155589	⊕⊕⊕⊕ High			0.184155589	⊕⊕⊕◯ Medium
53	Dapagliflozin:Sotagliflozin	0			0.230200022	⊕⊕◯◯ Low	0.230200022	⊕⊕◯◯ Low
54	Dapagliflozin:Tirzepatide	0			0.40950202	⊕⊕◯◯ Low	0.40950202	⊕⊕◯◯ Low
55	Dulaglutide:Efpeglenatide	0			-1.519155726	⊕⊕◯◯ Low	-1.519155726	⊕⊕◯◯ Low
56	Dulaglutide:Empagliflozin	0			-0.751537598	⊕⊕◯◯ Low	-0.751537598	⊕⊕◯◯ Low
57	Dulaglutide:Ertugliflozin	0			-0.092607492	⊕⊕◯◯ Low	-0.092607492	⊕⊕◯◯ Low
58	Dulaglutide:Exenatide	0			-0.608522313	⊕⊕◯◯ Low	-0.608522313	⊕⊕◯◯ Low
59	Dulaglutide:Inject_semaglutide	0			-0.542881255	⊕⊕◯◯ Low	-0.542881255	⊕⊕◯◯ Low
60	Dulaglutide:Liraglutide	0			0.21523669	⊕⊕◯◯ Low	0.21523669	⊕⊕◯◯ Low

61	Dulaglutide:Lixisenatide	0			0.493902616	⊕⊕◯◯ Low	0.493902616	⊕⊕◯◯ Low
62	Dulaglutide:Oral_semaglutide	0			-1.850149886	⊕⊕◯◯ Low	-1.850149886	⊕⊕◯◯ Low
63	Dulaglutide:Placebo_or_Control	3	-0.604709673	⊕⊕⊕⊕ High			-0.604709673	⊕⊕⊕◯ Medium
64	Dulaglutide:Sotagliflozin	0			-0.558665239	⊕⊕◯◯ Low	-0.558665239	⊕⊕◯◯ Low
65	Dulaglutide:Tirzepatide	0			-0.379363241	⊕⊕◯◯ Low	-0.379363241	⊕⊕◯◯ Low
66	Efpeglenatide:Empagliflozin	0			0.767618128	⊕⊕◯◯ Low	0.767618128	⊕⊕◯◯ Low
67	Efpeglenatide:Ertugliflozin	0			1.426548234	⊕⊕◯◯ Low	1.426548234	⊕⊕◯◯ Low
68	Efpeglenatide:Exenatide	0			0.910633412	⊕⊕◯◯ Low	0.910633412	⊕⊕◯◯ Low
69	Efpeglenatide:Inject_semaglutide	0			0.97627447	⊕⊕◯◯ Low	0.97627447	⊕⊕◯◯ Low
70	Efpeglenatide:Liraglutide	0			1.734392416	⊕⊕◯◯ Low	1.734392416	⊕⊕◯◯ Low
71	Efpeglenatide:Lixisenatide	0			2.013058342	⊕⊕◯◯ Low	2.013058342	⊕⊕◯◯ Low
72	Efpeglenatide:Oral_semaglutide	0			-0.330994161	⊕⊕◯◯ Low	-0.330994161	⊕⊕◯◯ Low
73	Efpeglenatide:Placebo_or_Control	1	0.914446053	⊕⊕⊕⊕ High			0.914446053	⊕⊕⊕◯ Medium
74	Efpeglenatide:Sotagliflozin	0			0.960490487	⊕⊕◯◯ Low	0.960490487	⊕⊕◯◯ Low
75	Efpeglenatide:Tirzepatide	0			1.139792484	⊕⊕◯◯ Low	1.139792484	⊕⊕◯◯ Low
76	Empagliflozin:Ertugliflozin	0			0.658930106	⊕⊕◯◯ Low	0.658930106	⊕⊕◯◯ Low
77	Empagliflozin:Exenatide	0			0.143015284	⊕⊕◯◯ Low	0.143015284	⊕⊕◯◯ Low
78	Empagliflozin:Inject_semaglutide	0			0.208656342	⊕⊕◯◯ Low	0.208656342	⊕⊕◯◯ Low
79	Empagliflozin:Liraglutide	0			0.966774287	⊕⊕◯◯ Low	0.966774287	⊕⊕◯◯ Low
80	Empagliflozin:Lixisenatide	0			1.245440214	⊕⊕◯◯ Low	1.245440214	⊕⊕◯◯ Low
81	Empagliflozin:Oral_semaglutide	1	-1.098612289	⊕⊕⊕⊕ High			-1.098612289	⊕⊕⊕◯ Medium

82	Empagliflozin:Placebo_or_Control	7	0.146827925	⊕⊕⊕⊕ High			0.146827925	⊕⊕⊕◯ Medium
83	Empagliflozin:Sotagliflozin	0			0.192872359	⊕⊕◯◯ Low	0.192872359	⊕⊕◯◯ Low
84	Empagliflozin:Tirzepatide	0			0.372174356	⊕⊕◯◯ Low	0.372174356	⊕⊕◯◯ Low
85	Ertugliflozin:Exenatide	0			-0.515914822	⊕⊕◯◯ Low	-0.515914822	⊕⊕◯◯ Low
86	Ertugliflozin:Inject_semaglutide	0			-0.450273764	⊕⊕◯◯ Low	-0.450273764	⊕⊕◯◯ Low
87	Ertugliflozin:Liraglutide	0			0.307844181	⊕⊕◯◯ Low	0.307844181	⊕⊕◯◯ Low
88	Ertugliflozin:Lixisenatide	0			0.586510108	⊕⊕◯◯ Low	0.586510108	⊕⊕◯◯ Low
89	Ertugliflozin:Oral_semaglutide	0			-1.757542395	⊕⊕◯◯ Low	-1.757542395	⊕⊕◯◯ Low
90	Ertugliflozin:Placebo_or_Control	1	-0.512102181	⊕⊕⊕⊕ High			-0.512102181	⊕⊕⊕◯ Medium
91	Ertugliflozin:Sotagliflozin	0			-0.466057747	⊕⊕◯◯ Low	-0.466057747	⊕⊕◯◯ Low
92	Ertugliflozin:Tirzepatide	0			-0.28675575	⊕⊕◯◯ Low	-0.28675575	⊕⊕◯◯ Low
93	Exenatide:Inject_semaglutide	0			0.065641058	⊕⊕◯◯ Low	0.065641058	⊕⊕◯◯ Low
94	Exenatide:Liraglutide	0			0.823759003	⊕⊕◯◯ Low	0.823759003	⊕⊕◯◯ Low
95	Exenatide:Lixisenatide	0			1.102424929	⊕⊕◯◯ Low	1.102424929	⊕⊕◯◯ Low
96	Exenatide:Oral_semaglutide	0			-1.241627573	⊕⊕◯◯ Low	-1.241627573	⊕⊕◯◯ Low
97	Exenatide:Placebo_or_Control	1	0.003812641	⊕⊕⊕⊕ High			0.003812641	⊕⊕⊕◯ Medium
98	Exenatide:Sotagliflozin	0			0.049857074	⊕⊕◯◯ Low	0.049857074	⊕⊕◯◯ Low
99	Exenatide:Tirzepatide	0			0.229159072	⊕⊕◯◯ Low	0.229159072	⊕⊕◯◯ Low
100	Inject_semaglutide:Liraglutide	0			0.758117945	⊕⊕◯◯ Low	0.758117945	⊕⊕◯◯ Low
101	Inject_semaglutide:Lixisenatide	0			1.036783871	⊕⊕◯◯ Low	1.036783871	⊕⊕◯◯ Low
102	Inject_semaglutide:Oral_semaglutide	0			-1.307268631	⊕⊕◯◯ Low	-1.307268631	⊕⊕◯◯ Low

103	Inject_semaglutide:Placebo_or_Control	10	-0.05946636	⊕⊕⊕⊕ High	-0.16665983	⊕⊕⊕⊕ High	-0.061828417	⊕⊕⊕⊕ High
104	Inject_semaglutide:Sotagliflozin	0			-0.015783984	⊕⊕◯◯ Low	-0.015783984	⊕⊕◯◯ Low
105	Inject_semaglutide:Tirzepatide	0			0.163518014	⊕⊕◯◯ Low	0.163518014	⊕⊕◯◯ Low
106	Liraglutide:Lixisenatide	0			0.278665926	⊕⊕◯◯ Low	0.278665926	⊕⊕◯◯ Low
107	Liraglutide:Oral_semaglutide	0			-2.065386576	⊕⊕◯◯ Low	-2.065386576	⊕⊕◯◯ Low
108	Liraglutide:Placebo_or_Control	3	-0.819946362	⊕⊕⊕⊕ High			-0.819946362	⊕⊕⊕◯ Medium
109	Liraglutide:Sotagliflozin	0			-0.773901929	⊕⊕◯◯ Low	-0.773901929	⊕⊕◯◯ Low
110	Liraglutide:Tirzepatide	0			-0.594599931	⊕⊕◯◯ Low	-0.594599931	⊕⊕◯◯ Low
111	Lixisenatide:Oral_semaglutide	0			-2.344052502	⊕⊕◯◯ Low	-2.344052502	⊕⊕◯◯ Low
112	Lixisenatide:Placebo_or_Control	1	-1.098612289	⊕⊕⊕⊕ High			-1.098612289	⊕⊕⊕◯ Medium
113	Lixisenatide:Sotagliflozin	0			-1.052567855	⊕⊕◯◯ Low	-1.052567855	⊕⊕◯◯ Low
114	Lixisenatide:Tirzepatide	0			-0.873265857	⊕⊕◯◯ Low	-0.873265857	⊕⊕◯◯ Low
115	Oral_semaglutide:Placebo_or_Control	0			1.245440214	⊕⊕◯◯ Low	1.245440214	⊕⊕◯◯ Low
116	Oral_semaglutide:Sotagliflozin	0			1.291484647	⊕⊕◯◯ Low	1.291484647	⊕⊕◯◯ Low
117	Oral_semaglutide:Tirzepatide	0			1.470786645	⊕⊕◯◯ Low	1.470786645	⊕⊕◯◯ Low
118	Sotagliflozin:Placebo_or_Control	4	-0.046044433	⊕⊕⊕⊕ High			-0.046044433	⊕⊕⊕◯ Medium
119	Tirzepatide:Placebo_or_Control	3	-0.225346431	⊕⊕⊕⊕ High			-0.225346431	⊕⊕⊕◯ Medium
120	Sotagliflozin:Tirzepatide	0			0.179301998	⊕⊕◯◯ Low	0.179301998	⊕⊕◯◯ Low

Table S8B: GRADE of primary outcome: intestine obstruction events in aspect of various dosage subgroup

	Comparison	No.Studies	Direct		Indirect		NMA	
			Estimate	Rate	Estimate	Rate	Estimate	Rate
1	Albiglutide:Bexagliflozin	0			1.429251017	⊕⊕◯◯ Low	1.429251017	⊕⊕◯◯ Low
2	Albiglutide:Canagliflozin_high_dosage	0			-1.186720088	⊕⊕◯◯ Low	-1.186720088	⊕⊕◯◯ Low
3	Albiglutide:Canagliflozin_low_dosage	0			-0.744542199	⊕⊕◯◯ Low	-0.744542199	⊕⊕◯◯ Low
4	Albiglutide:Dapagliflozin_high_dosage	0			-0.145936237	⊕⊕◯◯ Low	-0.145936237	⊕⊕◯◯ Low
5	Albiglutide:Dapagliflozin_low_dosage	0			-1.127955047	⊕⊕◯◯ Low	-1.127955047	⊕⊕◯◯ Low
6	Albiglutide:Dapagliflozin_medium_dosage	0			0.023809848	⊕⊕◯◯ Low	0.023809848	⊕⊕◯◯ Low
7	Albiglutide:Dulaglutide	0			0.646781764	⊕⊕◯◯ Low	0.646781764	⊕⊕◯◯ Low
8	Albiglutide:Efpeglenatide_high_dosage	0			-1.055801917	⊕⊕◯◯ Low	-1.055801917	⊕⊕◯◯ Low
9	Albiglutide:Efpeglenatide_medium_dosage	0			-0.648125234	⊕⊕◯◯ Low	-0.648125234	⊕⊕◯◯ Low
10	Albiglutide:Empagliflozin_high_dosage	0			-0.174764775	⊕⊕◯◯ Low	-0.174764775	⊕⊕◯◯ Low
11	Albiglutide:Empagliflozin_low_dosage	0			-0.097917792	⊕⊕◯◯ Low	-0.097917792	⊕⊕◯◯ Low
12	Albiglutide:Ertugliflozin_high_dosage	0			0.266309934	⊕⊕◯◯ Low	0.266309934	⊕⊕◯◯ Low
13	Albiglutide:Ertugliflozin_low_dosage	0			0.959821613	⊕⊕◯◯ Low	0.959821613	⊕⊕◯◯ Low
14	Albiglutide:Exenatide	0			0.038259451	⊕⊕◯◯ Low	0.038259451	⊕⊕◯◯ Low
15	Albiglutide:Inject_semaglutide_high_dosage	0			0.093350698	⊕⊕◯◯ Low	0.093350698	⊕⊕◯◯ Low
16	Albiglutide:Inject_semaglutide_low_dosage	0			-0.353226755	⊕⊕◯◯ Low	-0.353226755	⊕⊕◯◯ Low
17	Albiglutide:Inject_semaglutide_medium_dosage	0			-0.000743006	⊕⊕◯◯ Low	-0.000743006	⊕⊕◯◯ Low
18	Albiglutide:Liraglutide	0			0.862018454	⊕⊕◯◯ Low	0.862018454	⊕⊕◯◯ Low

19	Albiglutide:Lixisenatide	0			1.14068438	⊕⊕◯◯ Low	1.14068438	⊕⊕◯◯ Low
20	Albiglutide:Oral_semaglutide	0			-1.273377064	⊕⊕◯◯ Low	-1.273377064	⊕⊕◯◯ Low
21	Albiglutide:Placebo_or_Control	4	0.042072092	⊕⊕⊕⊕ High			0.042072092	⊕⊕⊕◯ Medium
22	Albiglutide:Sotagliflozin	0			0.088116525	⊕⊕◯◯ Low	0.088116525	⊕⊕◯◯ Low
23	Albiglutide:Tirzepatide_high_dosage	0			1.121830719	⊕⊕◯◯ Low	1.121830719	⊕⊕◯◯ Low
24	Albiglutide:Tirzepatide_low_dosage	0			1.121830719	⊕⊕◯◯ Low	1.121830719	⊕⊕◯◯ Low
25	Albiglutide:Tirzepatide_medium_dosage	0			1.131301981	⊕⊕◯◯ Low	1.131301981	⊕⊕◯◯ Low
26	Bexagliflozin:Canagliflozin_high_dosage	0			-2.615971105	⊕⊕◯◯ Low	-2.615971105	⊕⊕◯◯ Low
27	Bexagliflozin:Canagliflozin_low_dosage	0			-2.173793216	⊕⊕◯◯ Low	-2.173793216	⊕⊕◯◯ Low
28	Bexagliflozin:Dapagliflozin_high_dosage	0			-1.575187254	⊕⊕◯◯ Low	-1.575187254	⊕⊕◯◯ Low
29	Bexagliflozin:Dapagliflozin_low_dosage	0			-2.557206065	⊕⊕◯◯ Low	-2.557206065	⊕⊕◯◯ Low
30	Bexagliflozin:Dapagliflozin_medium_dosage	0			-1.405441169	⊕⊕◯◯ Low	-1.405441169	⊕⊕◯◯ Low
31	Bexagliflozin:Dulaglutide	0			-0.782469253	⊕⊕◯◯ Low	-0.782469253	⊕⊕◯◯ Low
32	Bexagliflozin:Efpeglenatide_high_dosage	0			-2.485052934	⊕⊕◯◯ Low	-2.485052934	⊕⊕◯◯ Low
33	Bexagliflozin:Efpeglenatide_medium_dosage	0			-2.077376251	⊕⊕◯◯ Low	-2.077376251	⊕⊕◯◯ Low
34	Bexagliflozin:Empagliflozin_high_dosage	0			-1.604015792	⊕⊕◯◯ Low	-1.604015792	⊕⊕◯◯ Low
35	Bexagliflozin:Empagliflozin_low_dosage	0			-1.527168809	⊕⊕◯◯ Low	-1.527168809	⊕⊕◯◯ Low
36	Bexagliflozin:Ertugliflozin_high_dosage	0			-1.162941083	⊕⊕◯◯ Low	-1.162941083	⊕⊕◯◯ Low
37	Bexagliflozin:Ertugliflozin_low_dosage	0			-0.469429404	⊕⊕◯◯ Low	-0.469429404	⊕⊕◯◯ Low
38	Bexagliflozin:Exenatide	0			-1.390991566	⊕⊕◯◯ Low	-1.390991566	⊕⊕◯◯ Low
39	Bexagliflozin:Inject_semaglutide_high_dosage	0			-1.335900319	⊕⊕◯◯ Low	-1.335900319	⊕⊕◯◯ Low

40	Bexagliflozin:Inject_semaglutide_low_dosage	0			-1.782477772	⊕⊕◯◯ Low	-1.782477772	⊕⊕◯◯ Low
41	Bexagliflozin:Inject_semaglutide_medium_dosage	0			-1.429994023	⊕⊕◯◯ Low	-1.429994023	⊕⊕◯◯ Low
42	Bexagliflozin:Liraglutide	0			-0.567232563	⊕⊕◯◯ Low	-0.567232563	⊕⊕◯◯ Low
43	Bexagliflozin:Lixisenatide	0			-0.288566637	⊕⊕◯◯ Low	-0.288566637	⊕⊕◯◯ Low
44	Bexagliflozin:Oral_semaglutide	0			-2.702628081	⊕⊕◯◯ Low	-2.702628081	⊕⊕◯◯ Low
45	Bexagliflozin:Placebo_or_Control	1	-1.387178926	⊕⊕⊕⊕ High			-1.387178926	⊕⊕⊕◯ Medium
46	Bexagliflozin:Sotagliflozin	0			-1.341134492	⊕⊕◯◯ Low	-1.341134492	⊕⊕◯◯ Low
47	Bexagliflozin:Tirzepatide_high_dosage	0			-0.307420298	⊕⊕◯◯ Low	-0.307420298	⊕⊕◯◯ Low
48	Bexagliflozin:Tirzepatide_low_dosage	0			-0.307420298	⊕⊕◯◯ Low	-0.307420298	⊕⊕◯◯ Low
49	Bexagliflozin:Tirzepatide_medium_dosage	0			-0.297949036	⊕⊕◯◯ Low	-0.297949036	⊕⊕◯◯ Low
50	Canagliflozin_high_dosage:Canagliflozin_low_dosage	2	0.628956128	⊕⊕⊕⊕ High	-0.096346704	⊕⊕⊕◯ Medium	0.442177889	⊕⊕⊕⊕ High
51	Canagliflozin_high_dosage:Dapagliflozin_high_dosage	0			1.040783851	⊕⊕◯◯ Low	1.040783851	⊕⊕◯◯ Low
52	Canagliflozin_high_dosage:Dapagliflozin_low_dosage	0			0.05876504	⊕⊕◯◯ Low	0.05876504	⊕⊕◯◯ Low
53	Canagliflozin_high_dosage:Dapagliflozin_medium_dosage	0			1.210529936	⊕⊕◯◯ Low	1.210529936	⊕⊕◯◯ Low
54	Canagliflozin_high_dosage:Dulaglutide	0			1.833501852	⊕⊕◯◯ Low	1.833501852	⊕⊕◯◯ Low
55	Canagliflozin_high_dosage:Efpeglenatide_high_dosage	0			0.130918171	⊕⊕◯◯ Low	0.130918171	⊕⊕◯◯ Low
56	Canagliflozin_high_dosage:Efpeglenatide_medium_dosage	0			0.538594854	⊕⊕◯◯ Low	0.538594854	⊕⊕◯◯ Low
57	Canagliflozin_high_dosage:Empagliflozin_high_dosage	0			1.011955313	⊕⊕◯◯ Low	1.011955313	⊕⊕◯◯ Low
58	Canagliflozin_high_dosage:Empagliflozin_low_dosage	0			1.088802296	⊕⊕◯◯ Low	1.088802296	⊕⊕◯◯ Low
59	Canagliflozin_high_dosage:Ertugliflozin_high_dosage	0			1.453030022	⊕⊕◯◯ Low	1.453030022	⊕⊕◯◯ Low
60	Canagliflozin_high_dosage:Ertugliflozin_low_dosage	0			2.146541701	⊕⊕◯◯ Low	2.146541701	⊕⊕◯◯ Low

61	Canagliflozin_high_dosage:Exenatide	0			1.224979539	⊕⊕◯◯ Low	1.224979539	⊕⊕◯◯ Low
62	Canagliflozin_high_dosage:Inject_semaglutide_high_dosage	0			1.280070786	⊕⊕◯◯ Low	1.280070786	⊕⊕◯◯ Low
63	Canagliflozin_high_dosage:Inject_semaglutide_low_dosage	0			0.833493333	⊕⊕◯◯ Low	0.833493333	⊕⊕◯◯ Low
64	Canagliflozin_high_dosage:Inject_semaglutide_medium_dosage	1	1.096067758	⊕⊕⊕⊕ High	1.211729442	⊕⊕⊕⊕ High	1.185977082	⊕⊕⊕⊕ High
65	Canagliflozin_high_dosage:Liraglutide	0			2.048738542	⊕⊕◯◯ Low	2.048738542	⊕⊕◯◯ Low
66	Canagliflozin_high_dosage:Lixisenatide	0			2.327404468	⊕⊕◯◯ Low	2.327404468	⊕⊕◯◯ Low
67	Canagliflozin_high_dosage:Oral_semaglutide	0			-0.086656976	⊕⊕◯◯ Low	-0.086656976	⊕⊕◯◯ Low
68	Canagliflozin_high_dosage:Placebo_or_Control	3	1.077244545	⊕⊕⊕⊕ High	1.465541213	⊕⊕⊕⊕ High	1.228792179	⊕⊕⊕⊕ High
69	Canagliflozin_high_dosage:Sotagliflozin	0			1.274836613	⊕⊕◯◯ Low	1.274836613	⊕⊕◯◯ Low
70	Canagliflozin_high_dosage:Tirzepatide_high_dosage	0			2.308550807	⊕⊕◯◯ Low	2.308550807	⊕⊕◯◯ Low
71	Canagliflozin_high_dosage:Tirzepatide_low_dosage	0			2.308550807	⊕⊕◯◯ Low	2.308550807	⊕⊕◯◯ Low
72	Canagliflozin_high_dosage:Tirzepatide_medium_dosage	0			2.318022069	⊕⊕◯◯ Low	2.318022069	⊕⊕◯◯ Low
73	Canagliflozin_low_dosage:Dapagliflozin_high_dosage	0			0.598605962	⊕⊕◯◯ Low	0.598605962	⊕⊕◯◯ Low
74	Canagliflozin_low_dosage:Dapagliflozin_low_dosage	0			-0.383412848	⊕⊕◯◯ Low	-0.383412848	⊕⊕◯◯ Low
75	Canagliflozin_low_dosage:Dapagliflozin_medium_dosage	0			0.768352047	⊕⊕◯◯ Low	0.768352047	⊕⊕◯◯ Low
76	Canagliflozin_low_dosage:Dulaglutide	0			1.391323963	⊕⊕◯◯ Low	1.391323963	⊕⊕◯◯ Low
77	Canagliflozin_low_dosage:Efpeglenatide_high_dosage	0			-0.311259718	⊕⊕◯◯ Low	-0.311259718	⊕⊕◯◯ Low
78	Canagliflozin_low_dosage:Efpeglenatide_medium_dosage	0			0.096416965	⊕⊕◯◯ Low	0.096416965	⊕⊕◯◯ Low
79	Canagliflozin_low_dosage:Empagliflozin_high_dosage	0			0.569777424	⊕⊕◯◯ Low	0.569777424	⊕⊕◯◯ Low
80	Canagliflozin_low_dosage:Empagliflozin_low_dosage	0			0.646624407	⊕⊕◯◯ Low	0.646624407	⊕⊕◯◯ Low
81	Canagliflozin_low_dosage:Ertugliflozin_high_dosage	0			1.010852134	⊕⊕◯◯ Low	1.010852134	⊕⊕◯◯ Low

82	Canagliflozin_low_dosage:Ertugliflozin_low_dosage	0			1.704363812	⊕⊕◯◯ Low	1.704363812	⊕⊕◯◯ Low
83	Canagliflozin_low_dosage:Exenatide	0			0.78280165	⊕⊕◯◯ Low	0.78280165	⊕⊕◯◯ Low
84	Canagliflozin_low_dosage:Inject_semaglutide_high_dosage	0			0.837892897	⊕⊕◯◯ Low	0.837892897	⊕⊕◯◯ Low
85	Canagliflozin_low_dosage:Inject_semaglutide_low_dosage	0			0.391315444	⊕⊕◯◯ Low	0.391315444	⊕⊕◯◯ Low
86	Canagliflozin_low_dosage:Inject_semaglutide_medium_dosage	0			0.743799193	⊕⊕◯◯ Low	0.743799193	⊕⊕◯◯ Low
87	Canagliflozin_low_dosage:Liraglutide	0			1.606560653	⊕⊕◯◯ Low	1.606560653	⊕⊕◯◯ Low
88	Canagliflozin_low_dosage:Lixisenatide	0			1.885226579	⊕⊕◯◯ Low	1.885226579	⊕⊕◯◯ Low
89	Canagliflozin_low_dosage:Oral_semaglutide	0			-0.528834865	⊕⊕◯◯ Low	-0.528834865	⊕⊕◯◯ Low
90	Canagliflozin_low_dosage:Placebo_or_Control	3	0.905290104	⊕⊕⊕⊕ High	-0.037637622	⊕⊕⊕◯ Medium	0.786614291	⊕⊕⊕⊕ High
91	Canagliflozin_low_dosage:Sotagliflozin	0			0.832658724	⊕⊕◯◯ Low	0.832658724	⊕⊕◯◯ Low
92	Canagliflozin_low_dosage:Tirzepatide_high_dosage	0			1.866372918	⊕⊕◯◯ Low	1.866372918	⊕⊕◯◯ Low
93	Canagliflozin_low_dosage:Tirzepatide_low_dosage	0			1.866372918	⊕⊕◯◯ Low	1.866372918	⊕⊕◯◯ Low
94	Canagliflozin_low_dosage:Tirzepatide_medium_dosage	0			1.875844181	⊕⊕◯◯ Low	1.875844181	⊕⊕◯◯ Low
95	Dapagliflozin_high_dosage:Dapagliflozin_low_dosage	1	-1.073485339	⊕⊕⊕⊕ High	-0.887646635	⊕⊕⊕⊕ High	-0.982018811	⊕⊕⊕⊕ High
96	Dapagliflozin_high_dosage:Dapagliflozin_medium_dosage	0			0.169746085	⊕⊕◯◯ Low	0.169746085	⊕⊕◯◯ Low
97	Dapagliflozin_high_dosage:Dulaglutide	0			0.792718001	⊕⊕◯◯ Low	0.792718001	⊕⊕◯◯ Low
98	Dapagliflozin_high_dosage:Efpeglenatide_high_dosage	0			-0.90986568	⊕⊕◯◯ Low	-0.90986568	⊕⊕◯◯ Low
99	Dapagliflozin_high_dosage:Efpeglenatide_medium_dosage	0			-0.502188997	⊕⊕◯◯ Low	-0.502188997	⊕⊕◯◯ Low
100	Dapagliflozin_high_dosage:Empagliflozin_high_dosage	0			-0.028828538	⊕⊕◯◯ Low	-0.028828538	⊕⊕◯◯ Low
101	Dapagliflozin_high_dosage:Empagliflozin_low_dosage	0			0.048018445	⊕⊕◯◯ Low	0.048018445	⊕⊕◯◯ Low
102	Dapagliflozin_high_dosage:Ertugliflozin_high_dosage	0			0.412246171	⊕⊕◯◯ Low	0.412246171	⊕⊕◯◯ Low

103	Dapagliflozin_high_dosage:Ertugliflozin_low_dosage	0			1.10575785	⊕⊕◯◯ Low	1.10575785	⊕⊕◯◯ Low
104	Dapagliflozin_high_dosage:Exenatide	0			0.184195688	⊕⊕◯◯ Low	0.184195688	⊕⊕◯◯ Low
105	Dapagliflozin_high_dosage:Inject_semaglutide_high_dosage	0			0.239286935	⊕⊕◯◯ Low	0.239286935	⊕⊕◯◯ Low
106	Dapagliflozin_high_dosage:Inject_semaglutide_low_dosage	0			-0.207290518	⊕⊕◯◯ Low	-0.207290518	⊕⊕◯◯ Low
107	Dapagliflozin_high_dosage:Inject_semaglutide_medium_dosage	0			0.145193231	⊕⊕◯◯ Low	0.145193231	⊕⊕◯◯ Low
108	Dapagliflozin_high_dosage:Liraglutide	0			1.007954691	⊕⊕◯◯ Low	1.007954691	⊕⊕◯◯ Low
109	Dapagliflozin_high_dosage:Lixisenatide	0			1.286620617	⊕⊕◯◯ Low	1.286620617	⊕⊕◯◯ Low
110	Dapagliflozin_high_dosage:Oral_semaglutide	0			-1.127440827	⊕⊕◯◯ Low	-1.127440827	⊕⊕◯◯ Low
111	Dapagliflozin_high_dosage:Placebo_or_Control	4	0.190914857	⊕⊕⊕⊕ High	0.005076153	⊕⊕⊕⊕ High	0.188008328	⊕⊕⊕⊕ High
112	Dapagliflozin_high_dosage:Sotagliflozin	0			0.234052762	⊕⊕◯◯ Low	0.234052762	⊕⊕◯◯ Low
113	Dapagliflozin_high_dosage:Tirzepatide_high_dosage	0			1.267766956	⊕⊕◯◯ Low	1.267766956	⊕⊕◯◯ Low
114	Dapagliflozin_high_dosage:Tirzepatide_low_dosage	0			1.267766956	⊕⊕◯◯ Low	1.267766956	⊕⊕◯◯ Low
115	Dapagliflozin_high_dosage:Tirzepatide_medium_dosage	0			1.277238218	⊕⊕◯◯ Low	1.277238218	⊕⊕◯◯ Low
116	Dapagliflozin_low_dosage:Dapagliflozin_medium_dosage	1	1.151764896	⊕⊕⊕⊕ High			1.151764896	⊕⊕⊕◯ Medium
117	Dapagliflozin_low_dosage:Dulaglutide	0			1.774736812	⊕⊕◯◯ Low	1.774736812	⊕⊕◯◯ Low
118	Dapagliflozin_low_dosage:Efpeglenatide_high_dosage	0			0.07215313	⊕⊕◯◯ Low	0.07215313	⊕⊕◯◯ Low
119	Dapagliflozin_low_dosage:Efpeglenatide_medium_dosage	0			0.479829813	⊕⊕◯◯ Low	0.479829813	⊕⊕◯◯ Low
120	Dapagliflozin_low_dosage:Empagliflozin_high_dosage	0			0.953190272	⊕⊕◯◯ Low	0.953190272	⊕⊕◯◯ Low
121	Dapagliflozin_low_dosage:Empagliflozin_low_dosage	0			1.030037256	⊕⊕◯◯ Low	1.030037256	⊕⊕◯◯ Low
122	Dapagliflozin_low_dosage:Ertugliflozin_high_dosage	0			1.394264982	⊕⊕◯◯ Low	1.394264982	⊕⊕◯◯ Low
123	Dapagliflozin_low_dosage:Ertugliflozin_low_dosage	0			2.08777666	⊕⊕◯◯ Low	2.08777666	⊕⊕◯◯ Low

124	Dapagliflozin_low_dosage:Exenatide	0			1.166214498	⊕⊕◯◯ Low	1.166214498	⊕⊕◯◯ Low
125	Dapagliflozin_low_dosage:Inject_semaglutide_high_dosage	0			1.221305745	⊕⊕◯◯ Low	1.221305745	⊕⊕◯◯ Low
126	Dapagliflozin_low_dosage:Inject_semaglutide_low_dosage	0			0.774728292	⊕⊕◯◯ Low	0.774728292	⊕⊕◯◯ Low
127	Dapagliflozin_low_dosage:Inject_semaglutide_medium_dosage	0			1.127212041	⊕⊕◯◯ Low	1.127212041	⊕⊕◯◯ Low
128	Dapagliflozin_low_dosage:Liraglutide	0			1.989973501	⊕⊕◯◯ Low	1.989973501	⊕⊕◯◯ Low
129	Dapagliflozin_low_dosage:Lixisenatide	0			2.268639428	⊕⊕◯◯ Low	2.268639428	⊕⊕◯◯ Low
130	Dapagliflozin_low_dosage:Oral_semaglutide	0			-0.145422016	⊕⊕◯◯ Low	-0.145422016	⊕⊕◯◯ Low
131	Dapagliflozin_low_dosage:Placebo_or_Control	1	1.078561492	⊕⊕⊕⊕ High	1.264400195	⊕⊕⊕⊕ High	1.170027139	⊕⊕⊕⊕ High
132	Dapagliflozin_low_dosage:Sotagliflozin	0			1.216071573	⊕⊕◯◯ Low	1.216071573	⊕⊕◯◯ Low
133	Dapagliflozin_low_dosage:Tirzepatide_high_dosage	0			2.249785766	⊕⊕◯◯ Low	2.249785766	⊕⊕◯◯ Low
134	Dapagliflozin_low_dosage:Tirzepatide_low_dosage	0			2.249785766	⊕⊕◯◯ Low	2.249785766	⊕⊕◯◯ Low
135	Dapagliflozin_low_dosage:Tirzepatide_medium_dosage	0			2.259257029	⊕⊕◯◯ Low	2.259257029	⊕⊕◯◯ Low
136	Dapagliflozin_medium_dosage:Dulaglutide	0			0.622971916	⊕⊕◯◯ Low	0.622971916	⊕⊕◯◯ Low
137	Dapagliflozin_medium_dosage:Efpeglenatide_high_dosage	0			-1.079611765	⊕⊕◯◯ Low	-1.079611765	⊕⊕◯◯ Low
138	Dapagliflozin_medium_dosage:Efpeglenatide_medium_dosage	0			-0.671935082	⊕⊕◯◯ Low	-0.671935082	⊕⊕◯◯ Low
139	Dapagliflozin_medium_dosage:Empagliflozin_high_dosage	0			-0.198574623	⊕⊕◯◯ Low	-0.198574623	⊕⊕◯◯ Low
140	Dapagliflozin_medium_dosage:Empagliflozin_low_dosage	0			-0.12172764	⊕⊕◯◯ Low	-0.12172764	⊕⊕◯◯ Low
141	Dapagliflozin_medium_dosage:Ertugliflozin_high_dosage	0			0.242500086	⊕⊕◯◯ Low	0.242500086	⊕⊕◯◯ Low
142	Dapagliflozin_medium_dosage:Ertugliflozin_low_dosage	0			0.936011765	⊕⊕◯◯ Low	0.936011765	⊕⊕◯◯ Low
143	Dapagliflozin_medium_dosage:Exenatide	0			0.014449603	⊕⊕◯◯ Low	0.014449603	⊕⊕◯◯ Low
144	Dapagliflozin_medium_dosage:Inject_semaglutide_high_dosage	0			0.06954085	⊕⊕◯◯ Low	0.06954085	⊕⊕◯◯ Low

145	Dapagliflozin_medium_dosage:Inject_semaglutide_low_dosage	0	-0.377036603	⊕⊕◯◯	Low	-0.377036603	⊕⊕◯◯	Low
146	Dapagliflozin_medium_dosage:Inject_semaglutide_medium_dosage	0	-0.024552854	⊕⊕◯◯	Low	-0.024552854	⊕⊕◯◯	Low
147	Dapagliflozin_medium_dosage:Liraglutide	0	0.838208606	⊕⊕◯◯	Low	0.838208606	⊕⊕◯◯	Low
148	Dapagliflozin_medium_dosage:Lixisenatide	0	1.116874532	⊕⊕◯◯	Low	1.116874532	⊕⊕◯◯	Low
149	Dapagliflozin_medium_dosage:Oral_semaglutide	0	-1.297186912	⊕⊕◯◯	Low	-1.297186912	⊕⊕◯◯	Low
150	Dapagliflozin_medium_dosage:Placebo_or_Control	0	0.018262243	⊕⊕◯◯	Low	0.018262243	⊕⊕◯◯	Low
151	Dapagliflozin_medium_dosage:Sotagliflozin	0	0.064306677	⊕⊕◯◯	Low	0.064306677	⊕⊕◯◯	Low
152	Dapagliflozin_medium_dosage:Tirzepatide_high_dosage	0	1.098020871	⊕⊕◯◯	Low	1.098020871	⊕⊕◯◯	Low
153	Dapagliflozin_medium_dosage:Tirzepatide_low_dosage	0	1.098020871	⊕⊕◯◯	Low	1.098020871	⊕⊕◯◯	Low
154	Dapagliflozin_medium_dosage:Tirzepatide_medium_dosage	0	1.107492133	⊕⊕◯◯	Low	1.107492133	⊕⊕◯◯	Low
155	Dulaglutide:Efpeglenatide_high_dosage	0	-1.702583681	⊕⊕◯◯	Low	-1.702583681	⊕⊕◯◯	Low
156	Dulaglutide:Efpeglenatide_medium_dosage	0	-1.294906998	⊕⊕◯◯	Low	-1.294906998	⊕⊕◯◯	Low
157	Dulaglutide:Empagliflozin_high_dosage	0	-0.821546539	⊕⊕◯◯	Low	-0.821546539	⊕⊕◯◯	Low
158	Dulaglutide:Empagliflozin_low_dosage	0	-0.744699556	⊕⊕◯◯	Low	-0.744699556	⊕⊕◯◯	Low
159	Dulaglutide:Ertugliflozin_high_dosage	0	-0.38047183	⊕⊕◯◯	Low	-0.38047183	⊕⊕◯◯	Low
160	Dulaglutide:Ertugliflozin_low_dosage	0	0.313039849	⊕⊕◯◯	Low	0.313039849	⊕⊕◯◯	Low
161	Dulaglutide:Exenatide	0	-0.608522313	⊕⊕◯◯	Low	-0.608522313	⊕⊕◯◯	Low
162	Dulaglutide:Inject_semaglutide_high_dosage	0	-0.553431066	⊕⊕◯◯	Low	-0.553431066	⊕⊕◯◯	Low
163	Dulaglutide:Inject_semaglutide_low_dosage	0	-1.000008519	⊕⊕◯◯	Low	-1.000008519	⊕⊕◯◯	Low
164	Dulaglutide:Inject_semaglutide_medium_dosage	0	-0.64752477	⊕⊕◯◯	Low	-0.64752477	⊕⊕◯◯	Low
165	Dulaglutide:Liraglutide	0	0.21523669	⊕⊕◯◯	Low	0.21523669	⊕⊕◯◯	Low

166	Dulaglutide:Lixisenatide	0			0.493902616	⊕⊕◯◯ Low	0.493902616	⊕⊕◯◯ Low
167	Dulaglutide:Oral_semaglutide	0			-1.920158828	⊕⊕◯◯ Low	-1.920158828	⊕⊕◯◯ Low
168	Dulaglutide:Placebo_or_Control	3	-0.604709673	⊕⊕⊕⊕ High			-0.604709673	⊕⊕⊕◯ Medium
169	Dulaglutide:Sotagliflozin	0			-0.558665239	⊕⊕◯◯ Low	-0.558665239	⊕⊕◯◯ Low
170	Dulaglutide:Tirzepatide_high_dosage	0			0.475048955	⊕⊕◯◯ Low	0.475048955	⊕⊕◯◯ Low
171	Dulaglutide:Tirzepatide_low_dosage	0			0.475048955	⊕⊕◯◯ Low	0.475048955	⊕⊕◯◯ Low
172	Dulaglutide:Tirzepatide_medium_dosage	0			0.484520217	⊕⊕◯◯ Low	0.484520217	⊕⊕◯◯ Low
173	Efpeglenatide_high_dosage:Efpeglenatide_medium_dosage	1	0.407676683	⊕⊕⊕⊕ High			0.407676683	⊕⊕⊕◯ Medium
174	Efpeglenatide_high_dosage:Empagliflozin_high_dosage	0			0.881037142	⊕⊕◯◯ Low	0.881037142	⊕⊕◯◯ Low
175	Efpeglenatide_high_dosage:Empagliflozin_low_dosage	0			0.957884125	⊕⊕◯◯ Low	0.957884125	⊕⊕◯◯ Low
176	Efpeglenatide_high_dosage:Ertugliflozin_high_dosage	0			1.322111852	⊕⊕◯◯ Low	1.322111852	⊕⊕◯◯ Low
177	Efpeglenatide_high_dosage:Ertugliflozin_low_dosage	0			2.01562353	⊕⊕◯◯ Low	2.01562353	⊕⊕◯◯ Low
178	Efpeglenatide_high_dosage:Exenatide	0			1.094061368	⊕⊕◯◯ Low	1.094061368	⊕⊕◯◯ Low
179	Efpeglenatide_high_dosage:Inject_semaglutide_high_dosage	0			1.149152615	⊕⊕◯◯ Low	1.149152615	⊕⊕◯◯ Low
180	Efpeglenatide_high_dosage:Inject_semaglutide_low_dosage	0			0.702575162	⊕⊕◯◯ Low	0.702575162	⊕⊕◯◯ Low
181	Efpeglenatide_high_dosage:Inject_semaglutide_medium_dosage	0			1.055058911	⊕⊕◯◯ Low	1.055058911	⊕⊕◯◯ Low
182	Efpeglenatide_high_dosage:Liraglutide	0			1.917820371	⊕⊕◯◯ Low	1.917820371	⊕⊕◯◯ Low
183	Efpeglenatide_high_dosage:Lixisenatide	0			2.196486297	⊕⊕◯◯ Low	2.196486297	⊕⊕◯◯ Low
184	Efpeglenatide_high_dosage:Oral_semaglutide	0			-0.217575147	⊕⊕◯◯ Low	-0.217575147	⊕⊕◯◯ Low
185	Efpeglenatide_high_dosage:Placebo_or_Control	1	1.097874009	⊕⊕⊕⊕ High			1.097874009	⊕⊕⊕◯ Medium
186	Efpeglenatide_high_dosage:Sotagliflozin	0			1.143918442	⊕⊕◯◯ Low	1.143918442	⊕⊕◯◯ Low

187	Efpeglenatide_high_dosage:Tirzepatide_high_dosage	0			2.177632636	⊕⊕◯◯ Low	2.177632636	⊕⊕◯◯ Low
188	Efpeglenatide_high_dosage:Tirzepatide_low_dosage	0			2.177632636	⊕⊕◯◯ Low	2.177632636	⊕⊕◯◯ Low
189	Efpeglenatide_high_dosage:Tirzepatide_medium_dosage	0			2.187103899	⊕⊕◯◯ Low	2.187103899	⊕⊕◯◯ Low
190	Efpeglenatide_medium_dosage:Empagliflozin_high_dosage	0			0.473360459	⊕⊕◯◯ Low	0.473360459	⊕⊕◯◯ Low
191	Efpeglenatide_medium_dosage:Empagliflozin_low_dosage	0			0.550207443	⊕⊕◯◯ Low	0.550207443	⊕⊕◯◯ Low
192	Efpeglenatide_medium_dosage:Ertugliflozin_high_dosage	0			0.914435169	⊕⊕◯◯ Low	0.914435169	⊕⊕◯◯ Low
193	Efpeglenatide_medium_dosage:Ertugliflozin_low_dosage	0			1.607946847	⊕⊕◯◯ Low	1.607946847	⊕⊕◯◯ Low
194	Efpeglenatide_medium_dosage:Exenatide	0			0.686384685	⊕⊕◯◯ Low	0.686384685	⊕⊕◯◯ Low
195	Efpeglenatide_medium_dosage:Inject_semaglutide_high_dosage	0			0.741475932	⊕⊕◯◯ Low	0.741475932	⊕⊕◯◯ Low
196	Efpeglenatide_medium_dosage:Inject_semaglutide_low_dosage	0			0.294898479	⊕⊕◯◯ Low	0.294898479	⊕⊕◯◯ Low
197	Efpeglenatide_medium_dosage:Inject_semaglutide_medium_dosage	0			0.647382228	⊕⊕◯◯ Low	0.647382228	⊕⊕◯◯ Low
198	Efpeglenatide_medium_dosage:Liraglutide	0			1.510143688	⊕⊕◯◯ Low	1.510143688	⊕⊕◯◯ Low
199	Efpeglenatide_medium_dosage:Lixisenatide	0			1.788809615	⊕⊕◯◯ Low	1.788809615	⊕⊕◯◯ Low
200	Efpeglenatide_medium_dosage:Oral_semaglutide	0			-0.62525183	⊕⊕◯◯ Low	-0.62525183	⊕⊕◯◯ Low
201	Efpeglenatide_medium_dosage:Placebo_or_Control	1	0.690197326	⊕⊕⊕⊕ High			0.690197326	⊕⊕⊕◯ Medium
202	Efpeglenatide_medium_dosage:Sotagliflozin	0			0.736241759	⊕⊕◯◯ Low	0.736241759	⊕⊕◯◯ Low
203	Efpeglenatide_medium_dosage:Tirzepatide_high_dosage	0			1.769955953	⊕⊕◯◯ Low	1.769955953	⊕⊕◯◯ Low
204	Efpeglenatide_medium_dosage:Tirzepatide_low_dosage	0			1.769955953	⊕⊕◯◯ Low	1.769955953	⊕⊕◯◯ Low
205	Efpeglenatide_medium_dosage:Tirzepatide_medium_dosage	0			1.779427216	⊕⊕◯◯ Low	1.779427216	⊕⊕◯◯ Low
206	Empagliflozin_high_dosage:Empagliflozin_low_dosage	2	0.180777994	⊕⊕⊕⊕ High	-0.134210771	⊕⊕⊕◯ Medium	0.076846983	⊕⊕⊕⊕ High
207	Empagliflozin_high_dosage:Ertugliflozin_high_dosage	0			0.44107471	⊕⊕◯◯ Low	0.44107471	⊕⊕◯◯ Low

208	Empagliflozin_high_dosage:Ertugliflozin_low_dosage	0			1.134586388	⊕⊕◯◯ Low	1.134586388	⊕⊕◯◯ Low
209	Empagliflozin_high_dosage:Exenatide	0			0.213024226	⊕⊕◯◯ Low	0.213024226	⊕⊕◯◯ Low
210	Empagliflozin_high_dosage:Inject_semaglutide_high_dosage	0			0.268115473	⊕⊕◯◯ Low	0.268115473	⊕⊕◯◯ Low
211	Empagliflozin_high_dosage:Inject_semaglutide_low_dosage	0			-0.17846198	⊕⊕◯◯ Low	-0.17846198	⊕⊕◯◯ Low
212	Empagliflozin_high_dosage:Inject_semaglutide_medium_dosage	0			0.174021769	⊕⊕◯◯ Low	0.174021769	⊕⊕◯◯ Low
213	Empagliflozin_high_dosage:Liraglutide	0			1.036783229	⊕⊕◯◯ Low	1.036783229	⊕⊕◯◯ Low
214	Empagliflozin_high_dosage:Lixisenatide	0			1.315449155	⊕⊕◯◯ Low	1.315449155	⊕⊕◯◯ Low
215	Empagliflozin_high_dosage:Oral_semaglutide	1	-1.098612289	⊕⊕⊕⊕ High			-1.098612289	⊕⊕⊕◯ Medium
216	Empagliflozin_high_dosage:Placebo_or_Control	3	0.092642545	⊕⊕⊕⊕ High	0.80337523	⊕⊕⊕⊕ High	0.216836867	⊕⊕⊕⊕ High
217	Empagliflozin_high_dosage:Sotagliflozin	0			0.2628813	⊕⊕◯◯ Low	0.2628813	⊕⊕◯◯ Low
218	Empagliflozin_high_dosage:Tirzepatide_high_dosage	0			1.296595494	⊕⊕◯◯ Low	1.296595494	⊕⊕◯◯ Low
219	Empagliflozin_high_dosage:Tirzepatide_low_dosage	0			1.296595494	⊕⊕◯◯ Low	1.296595494	⊕⊕◯◯ Low
220	Empagliflozin_high_dosage:Tirzepatide_medium_dosage	0			1.306066757	⊕⊕◯◯ Low	1.306066757	⊕⊕◯◯ Low
221	Empagliflozin_low_dosage:Ertugliflozin_high_dosage	0			0.364227726	⊕⊕◯◯ Low	0.364227726	⊕⊕◯◯ Low
222	Empagliflozin_low_dosage:Ertugliflozin_low_dosage	0			1.057739405	⊕⊕◯◯ Low	1.057739405	⊕⊕◯◯ Low
223	Empagliflozin_low_dosage:Exenatide	0			0.136177243	⊕⊕◯◯ Low	0.136177243	⊕⊕◯◯ Low
224	Empagliflozin_low_dosage:Inject_semaglutide_high_dosage	0			0.19126849	⊕⊕◯◯ Low	0.19126849	⊕⊕◯◯ Low
225	Empagliflozin_low_dosage:Inject_semaglutide_low_dosage	0			-0.255308963	⊕⊕◯◯ Low	-0.255308963	⊕⊕◯◯ Low
226	Empagliflozin_low_dosage:Inject_semaglutide_medium_dosage	0			0.097174786	⊕⊕◯◯ Low	0.097174786	⊕⊕◯◯ Low
227	Empagliflozin_low_dosage:Liraglutide	0			0.959936246	⊕⊕◯◯ Low	0.959936246	⊕⊕◯◯ Low
228	Empagliflozin_low_dosage:Lixisenatide	0			1.238602172	⊕⊕◯◯ Low	1.238602172	⊕⊕◯◯ Low

229	Empagliflozin_low_dosage:Oral_semaglutide	0			-1.175459272	⊕⊕◯◯ Low	-1.175459272	⊕⊕◯◯ Low
230	Empagliflozin_low_dosage:Placebo_or_Control	5	0.112547472	⊕⊕⊕⊕ High	1.335245216	⊕⊕⊕⊕ High	0.139989883	⊕⊕⊕⊕ High
231	Empagliflozin_low_dosage:Sotagliflozin	0			0.186034317	⊕⊕◯◯ Low	0.186034317	⊕⊕◯◯ Low
232	Empagliflozin_low_dosage:Tirzepatide_high_dosage	0			1.219748511	⊕⊕◯◯ Low	1.219748511	⊕⊕◯◯ Low
233	Empagliflozin_low_dosage:Tirzepatide_low_dosage	0			1.219748511	⊕⊕◯◯ Low	1.219748511	⊕⊕◯◯ Low
234	Empagliflozin_low_dosage:Tirzepatide_medium_dosage	0			1.229219773	⊕⊕◯◯ Low	1.229219773	⊕⊕◯◯ Low
235	Ertugliflozin_high_dosage:Ertugliflozin_low_dosage	1	0.693511678	⊕⊕⊕⊕ High			0.693511678	⊕⊕⊕◯ Medium
236	Ertugliflozin_high_dosage:Exenatide	0			-0.228050484	⊕⊕◯◯ Low	-0.228050484	⊕⊕◯◯ Low
237	Ertugliflozin_high_dosage:Inject_semaglutide_high_dosage	0			-0.172959237	⊕⊕◯◯ Low	-0.172959237	⊕⊕◯◯ Low
238	Ertugliflozin_high_dosage:Inject_semaglutide_low_dosage	0			-0.61953669	⊕⊕◯◯ Low	-0.61953669	⊕⊕◯◯ Low
239	Ertugliflozin_high_dosage:Inject_semaglutide_medium_dosage	0			-0.267052941	⊕⊕◯◯ Low	-0.267052941	⊕⊕◯◯ Low
240	Ertugliflozin_high_dosage:Liraglutide	0			0.595708519	⊕⊕◯◯ Low	0.595708519	⊕⊕◯◯ Low
241	Ertugliflozin_high_dosage:Lixisenatide	0			0.874374446	⊕⊕◯◯ Low	0.874374446	⊕⊕◯◯ Low
242	Ertugliflozin_high_dosage:Oral_semaglutide	0			-1.539686998	⊕⊕◯◯ Low	-1.539686998	⊕⊕◯◯ Low
243	Ertugliflozin_high_dosage:Placebo_or_Control	1	-0.224237843	⊕⊕⊕⊕ High			-0.224237843	⊕⊕⊕◯ Medium
244	Ertugliflozin_high_dosage:Sotagliflozin	0			-0.178193409	⊕⊕◯◯ Low	-0.178193409	⊕⊕◯◯ Low
245	Ertugliflozin_high_dosage:Tirzepatide_high_dosage	0			0.855520784	⊕⊕◯◯ Low	0.855520784	⊕⊕◯◯ Low
246	Ertugliflozin_high_dosage:Tirzepatide_low_dosage	0			0.855520784	⊕⊕◯◯ Low	0.855520784	⊕⊕◯◯ Low
247	Ertugliflozin_high_dosage:Tirzepatide_medium_dosage	0			0.864992047	⊕⊕◯◯ Low	0.864992047	⊕⊕◯◯ Low
248	Ertugliflozin_low_dosage:Exenatide	0			-0.921562162	⊕⊕◯◯ Low	-0.921562162	⊕⊕◯◯ Low
249	Ertugliflozin_low_dosage:Inject_semaglutide_high_dosage	0			-0.866470915	⊕⊕◯◯ Low	-0.866470915	⊕⊕◯◯ Low

250	Ertugliflozin_low_dosage:Inject_semaglutide_low_dosage	0			-1.313048368	⊕⊕◯◯ Low	-1.313048368	⊕⊕◯◯ Low
251	Ertugliflozin_low_dosage:Inject_semaglutide_medium_dosage	0			-0.960564619	⊕⊕◯◯ Low	-0.960564619	⊕⊕◯◯ Low
252	Ertugliflozin_low_dosage:Liraglutide	0			-0.097803159	⊕⊕◯◯ Low	-0.097803159	⊕⊕◯◯ Low
253	Ertugliflozin_low_dosage:Lixisenatide	0			0.180862767	⊕⊕◯◯ Low	0.180862767	⊕⊕◯◯ Low
254	Ertugliflozin_low_dosage:Oral_semaglutide	0			-2.233198677	⊕⊕◯◯ Low	-2.233198677	⊕⊕◯◯ Low
255	Ertugliflozin_low_dosage:Placebo_or_Control	1	-0.917749521	⊕⊕⊕⊕ High			-0.917749521	⊕⊕⊕◯ Medium
256	Ertugliflozin_low_dosage:Sotagliflozin	0			-0.871705088	⊕⊕◯◯ Low	-0.871705088	⊕⊕◯◯ Low
257	Ertugliflozin_low_dosage:Tirzepatide_high_dosage	0			0.162009106	⊕⊕◯◯ Low	0.162009106	⊕⊕◯◯ Low
258	Ertugliflozin_low_dosage:Tirzepatide_low_dosage	0			0.162009106	⊕⊕◯◯ Low	0.162009106	⊕⊕◯◯ Low
259	Ertugliflozin_low_dosage:Tirzepatide_medium_dosage	0			0.171480369	⊕⊕◯◯ Low	0.171480369	⊕⊕◯◯ Low
260	Exenatide:Inject_semaglutide_high_dosage	0			0.055091247	⊕⊕◯◯ Low	0.055091247	⊕⊕◯◯ Low
261	Exenatide:Inject_semaglutide_low_dosage	0			-0.391486206	⊕⊕◯◯ Low	-0.391486206	⊕⊕◯◯ Low
262	Exenatide:Inject_semaglutide_medium_dosage	0			-0.039002457	⊕⊕◯◯ Low	-0.039002457	⊕⊕◯◯ Low
263	Exenatide:Liraglutide	0			0.823759003	⊕⊕◯◯ Low	0.823759003	⊕⊕◯◯ Low
264	Exenatide:Lixisenatide	0			1.102424929	⊕⊕◯◯ Low	1.102424929	⊕⊕◯◯ Low
265	Exenatide:Oral_semaglutide	0			-1.311636515	⊕⊕◯◯ Low	-1.311636515	⊕⊕◯◯ Low
266	Exenatide:Placebo_or_Control	1	0.003812641	⊕⊕⊕⊕ High			0.003812641	⊕⊕⊕◯ Medium
267	Exenatide:Sotagliflozin	0			0.049857074	⊕⊕◯◯ Low	0.049857074	⊕⊕◯◯ Low
268	Exenatide:Tirzepatide_high_dosage	0			1.083571268	⊕⊕◯◯ Low	1.083571268	⊕⊕◯◯ Low
269	Exenatide:Tirzepatide_low_dosage	0			1.083571268	⊕⊕◯◯ Low	1.083571268	⊕⊕◯◯ Low
270	Exenatide:Tirzepatide_medium_dosage	0			1.093042531	⊕⊕◯◯ Low	1.093042531	⊕⊕◯◯ Low

271	Inject_semaglutide_high_dosage:Inject_semaglutide_low_dosage	0			-0.446577453	⊕⊕◯◯ Low	-0.446577453	⊕⊕◯◯ Low
272	Inject_semaglutide_high_dosage:Inject_semaglutide_medium_dosage	1	1.098612289	⊕⊕⊕⊕ High	-0.300862976	⊕⊕⊕◯ Medium	-0.094093704	⊕⊕⊕⊕ High
273	Inject_semaglutide_high_dosage:Liraglutide	0			0.768667756	⊕⊕◯◯ Low	0.768667756	⊕⊕◯◯ Low
274	Inject_semaglutide_high_dosage:Lixisenatide	0			1.047333682	⊕⊕◯◯ Low	1.047333682	⊕⊕◯◯ Low
275	Inject_semaglutide_high_dosage:Oral_semaglutide	0			-1.366727762	⊕⊕◯◯ Low	-1.366727762	⊕⊕◯◯ Low
276	Inject_semaglutide_high_dosage:Placebo_or_Control	5	-0.08640009	⊕⊕⊕⊕ High	1.313075175	⊕⊕⊕◯ Medium	-0.051278606	⊕⊕⊕⊕ High
277	Inject_semaglutide_high_dosage:Sotagliflozin	0			-0.005234173	⊕⊕◯◯ Low	-0.005234173	⊕⊕◯◯ Low
278	Inject_semaglutide_high_dosage:Tirzepatide_high_dosage	0			1.028480021	⊕⊕◯◯ Low	1.028480021	⊕⊕◯◯ Low
279	Inject_semaglutide_high_dosage:Tirzepatide_low_dosage	0			1.028480021	⊕⊕◯◯ Low	1.028480021	⊕⊕◯◯ Low
280	Inject_semaglutide_high_dosage:Tirzepatide_medium_dosage	0			1.037951284	⊕⊕◯◯ Low	1.037951284	⊕⊕◯◯ Low
281	Inject_semaglutide_low_dosage:Inject_semaglutide_medium_dosage	4	0.253360394	⊕⊕⊕⊕ High	0.639427433	⊕⊕⊕⊕ High	0.352483749	⊕⊕⊕⊕ High
282	Inject_semaglutide_low_dosage:Liraglutide	0			1.215245209	⊕⊕◯◯ Low	1.215245209	⊕⊕◯◯ Low
283	Inject_semaglutide_low_dosage:Lixisenatide	0			1.493911136	⊕⊕◯◯ Low	1.493911136	⊕⊕◯◯ Low
284	Inject_semaglutide_low_dosage:Oral_semaglutide	0			-0.920150309	⊕⊕◯◯ Low	-0.920150309	⊕⊕◯◯ Low
285	Inject_semaglutide_low_dosage:Placebo_or_Control	3	0.399134267	⊕⊕⊕⊕ High	0.387493531	⊕⊕⊕⊕ High	0.395298847	⊕⊕⊕⊕ High
286	Inject_semaglutide_low_dosage:Sotagliflozin	0			0.44134328	⊕⊕◯◯ Low	0.44134328	⊕⊕◯◯ Low
287	Inject_semaglutide_low_dosage:Tirzepatide_high_dosage	0			1.475057474	⊕⊕◯◯ Low	1.475057474	⊕⊕◯◯ Low
288	Inject_semaglutide_low_dosage:Tirzepatide_low_dosage	0			1.475057474	⊕⊕◯◯ Low	1.475057474	⊕⊕◯◯ Low
289	Inject_semaglutide_low_dosage:Tirzepatide_medium_dosage	0			1.484528737	⊕⊕◯◯ Low	1.484528737	⊕⊕◯◯ Low
290	Inject_semaglutide_medium_dosage:Liraglutide	0			0.86276146	⊕⊕◯◯ Low	0.86276146	⊕⊕◯◯ Low
291	Inject_semaglutide_medium_dosage:Lixisenatide	0			1.141427386	⊕⊕◯◯ Low	1.141427386	⊕⊕◯◯ Low

292	Inject_semaglutide_medium_dosage:Oral_semaglutide	0			-1.272634058	⊕⊕◯◯ Low	-1.272634058	⊕⊕◯◯ Low
293	Inject_semaglutide_medium_dosage:Placebo_or_Control	4	0.230067853	⊕⊕⊕⊕ High	-0.241399979	⊕⊕⊕◯ Medium	0.042815098	⊕⊕⊕⊕ High
294	Inject_semaglutide_medium_dosage:Sotagliflozin	0			0.088859531	⊕⊕◯◯ Low	0.088859531	⊕⊕◯◯ Low
295	Inject_semaglutide_medium_dosage:Tirzepatide_high_dosage	0			1.122573725	⊕⊕◯◯ Low	1.122573725	⊕⊕◯◯ Low
296	Inject_semaglutide_medium_dosage:Tirzepatide_low_dosage	0			1.122573725	⊕⊕◯◯ Low	1.122573725	⊕⊕◯◯ Low
297	Inject_semaglutide_medium_dosage:Tirzepatide_medium_dosage	0			1.132044988	⊕⊕◯◯ Low	1.132044988	⊕⊕◯◯ Low
298	Liraglutide:Lixisenatide	0			0.278665926	⊕⊕◯◯ Low	0.278665926	⊕⊕◯◯ Low
299	Liraglutide:Oral_semaglutide	0			-2.135395518	⊕⊕◯◯ Low	-2.135395518	⊕⊕◯◯ Low
300	Liraglutide:Placebo_or_Control	3	-0.819946362	⊕⊕⊕⊕ High			-0.819946362	⊕⊕⊕◯ Medium
301	Liraglutide:Sotagliflozin	0			-0.773901929	⊕⊕◯◯ Low	-0.773901929	⊕⊕◯◯ Low
302	Liraglutide:Tirzepatide_high_dosage	0			0.259812265	⊕⊕◯◯ Low	0.259812265	⊕⊕◯◯ Low
303	Liraglutide:Tirzepatide_low_dosage	0			0.259812265	⊕⊕◯◯ Low	0.259812265	⊕⊕◯◯ Low
304	Liraglutide:Tirzepatide_medium_dosage	0			0.269283528	⊕⊕◯◯ Low	0.269283528	⊕⊕◯◯ Low
305	Lixisenatide:Oral_semaglutide	0			-2.414061444	⊕⊕◯◯ Low	-2.414061444	⊕⊕◯◯ Low
306	Lixisenatide:Placebo_or_Control	1	-1.098612289	⊕⊕⊕⊕ High			-1.098612289	⊕⊕⊕◯ Medium
307	Lixisenatide:Sotagliflozin	0			-1.052567855	⊕⊕◯◯ Low	-1.052567855	⊕⊕◯◯ Low
308	Lixisenatide:Tirzepatide_high_dosage	0			-0.018853661	⊕⊕◯◯ Low	-0.018853661	⊕⊕◯◯ Low
309	Lixisenatide:Tirzepatide_low_dosage	0			-0.018853661	⊕⊕◯◯ Low	-0.018853661	⊕⊕◯◯ Low
310	Lixisenatide:Tirzepatide_medium_dosage	0			-0.009382399	⊕⊕◯◯ Low	-0.009382399	⊕⊕◯◯ Low
311	Oral_semaglutide:Placebo_or_Control	0			1.315449155	⊕⊕◯◯ Low	1.315449155	⊕⊕◯◯ Low
312	Oral_semaglutide:Sotagliflozin	0			1.361493589	⊕⊕◯◯ Low	1.361493589	⊕⊕◯◯ Low

313	Oral_semaglutide:Tirzepatide_high_dosage	0			2.395207783	⊕⊕◯◯ Low	2.395207783	⊕⊕◯◯ Low
314	Oral_semaglutide:Tirzepatide_low_dosage	0			2.395207783	⊕⊕◯◯ Low	2.395207783	⊕⊕◯◯ Low
315	Oral_semaglutide:Tirzepatide_medium_dosage	0			2.404679045	⊕⊕◯◯ Low	2.404679045	⊕⊕◯◯ Low
316	Sotagliflozin:Placebo_or_Control	4	-0.046044433	⊕⊕⊕⊕ High			-0.046044433	⊕⊕⊕◯ Medium
317	Tirzepatide_high_dosage:Placebo_or_Control	1	-1.079758627	⊕⊕⊕⊕ High			-1.079758627	⊕⊕⊕◯ Medium
318	Tirzepatide_low_dosage:Placebo_or_Control	1	-1.079758627	⊕⊕⊕⊕ High			-1.079758627	⊕⊕⊕◯ Medium
319	Tirzepatide_medium_dosage:Placebo_or_Control	1	-1.08922989	⊕⊕⊕⊕ High			-1.08922989	⊕⊕⊕◯ Medium
320	Sotagliflozin:Tirzepatide_high_dosage	0			1.033714194	⊕⊕◯◯ Low	1.033714194	⊕⊕◯◯ Low
321	Sotagliflozin:Tirzepatide_low_dosage	0			1.033714194	⊕⊕◯◯ Low	1.033714194	⊕⊕◯◯ Low
322	Sotagliflozin:Tirzepatide_medium_dosage	0			1.043185456	⊕⊕◯◯ Low	1.043185456	⊕⊕◯◯ Low
323	Tirzepatide_high_dosage:Tirzepatide_low_dosage	0			0	⊕⊕◯◯ Low	0	⊕⊕◯◯ Low
324	Tirzepatide_high_dosage:Tirzepatide_medium_dosage	0			0.009471263	⊕⊕◯◯ Low	0.009471263	⊕⊕◯◯ Low
325	Tirzepatide_low_dosage:Tirzepatide_medium_dosage	0			0.009471263	⊕⊕◯◯ Low	0.009471263	⊕⊕◯◯ Low

Table S8C: GRADE of acceptability: drop-out rate

	Comparison	No.Studies	Direct		Indirect		NMA	
			Estimate	Rate	Estimate	Rate	Estimate	Rate
1	Albiglutide:Bexagliflozin	0			-0.214206286	⊕⊕◯◯ Low	-0.214206286	⊕⊕◯◯ Low
2	Albiglutide:Canagliflozin	0			0.06632405	⊕⊕◯◯ Low	0.06632405	⊕⊕◯◯ Low
3	Albiglutide:Dapagliflozin	0			-0.106001699	⊕⊕◯◯ Low	-0.106001699	⊕⊕◯◯ Low
4	Albiglutide:Dulaglutide	0			-0.13325246	⊕⊕◯◯ Low	-0.13325246	⊕⊕◯◯ Low
5	Albiglutide:Efpeglenatide	0			-0.323625032	⊕⊕◯◯ Low	-0.323625032	⊕⊕◯◯ Low
6	Albiglutide:Empagliflozin	0			-0.202371738	⊕⊕◯◯ Low	-0.202371738	⊕⊕◯◯ Low
7	Albiglutide:Ertugliflozin	0			-0.279780415	⊕⊕◯◯ Low	-0.279780415	⊕⊕◯◯ Low
8	Albiglutide:Exenatide	0			-0.201112322	⊕⊕◯◯ Low	-0.201112322	⊕⊕◯◯ Low
9	Albiglutide:Inject_semaglutide	0			-0.138371925	⊕⊕◯◯ Low	-0.138371925	⊕⊕◯◯ Low
10	Albiglutide:Liraglutide	0			-0.077538442	⊕⊕◯◯ Low	-0.077538442	⊕⊕◯◯ Low
11	Albiglutide:Lixisenatide	0			-0.298413057	⊕⊕◯◯ Low	-0.298413057	⊕⊕◯◯ Low
12	Albiglutide:Oral_semaglutide	0			0.568267059	⊕⊕◯◯ Low	0.568267059	⊕⊕◯◯ Low
13	Albiglutide:Placebo_or_Control	4	-0.346641598	⊕⊕⊕⊕ High			-0.346641598	⊕⊕⊕◯ Medium
14	Albiglutide:Sotagliflozin	0			-0.192949245	⊕⊕◯◯ Low	-0.192949245	⊕⊕◯◯ Low
15	Albiglutide:Tirzepatide	0			0.468853277	⊕⊕◯◯ Low	0.468853277	⊕⊕◯◯ Low
16	Bexagliflozin:Canagliflozin	0			0.280530335	⊕⊕◯◯ Low	0.280530335	⊕⊕◯◯ Low
17	Bexagliflozin:Dapagliflozin	0			0.108204587	⊕⊕◯◯ Low	0.108204587	⊕⊕◯◯ Low
18	Bexagliflozin:Dulaglutide	0			0.080953825	⊕⊕◯◯ Low	0.080953825	⊕⊕◯◯ Low

19	Bexagliflozin:Efpeglenatide	0			-0.109418746	⊕⊕◯◯ Low	-0.109418746	⊕⊕◯◯ Low
20	Bexagliflozin:Empagliflozin	0			0.011834547	⊕⊕◯◯ Low	0.011834547	⊕⊕◯◯ Low
21	Bexagliflozin:Ertugliflozin	0			-0.065574129	⊕⊕◯◯ Low	-0.065574129	⊕⊕◯◯ Low
22	Bexagliflozin:Exenatide	0			0.013093963	⊕⊕◯◯ Low	0.013093963	⊕⊕◯◯ Low
23	Bexagliflozin:Inject_semaglutide	0			0.07583436	⊕⊕◯◯ Low	0.07583436	⊕⊕◯◯ Low
24	Bexagliflozin:Liraglutide	0			0.136667844	⊕⊕◯◯ Low	0.136667844	⊕⊕◯◯ Low
25	Bexagliflozin:Lixisenatide	0			-0.084206771	⊕⊕◯◯ Low	-0.084206771	⊕⊕◯◯ Low
26	Bexagliflozin:Oral_semaglutide	0			0.782473344	⊕⊕◯◯ Low	0.782473344	⊕⊕◯◯ Low
27	Bexagliflozin:Placebo_or_Control	1	-0.132435312	⊕⊕⊕⊕ High			-0.132435312	⊕⊕⊕◯ Medium
28	Bexagliflozin:Sotagliflozin	0			0.021257041	⊕⊕◯◯ Low	0.021257041	⊕⊕◯◯ Low
29	Bexagliflozin:Tirzepatide	0			0.683059563	⊕⊕◯◯ Low	0.683059563	⊕⊕◯◯ Low
30	Canagliflozin:Dapagliflozin	0			-0.172325749	⊕⊕◯◯ Low	-0.172325749	⊕⊕◯◯ Low
31	Canagliflozin:Dulaglutide	0			-0.19957651	⊕⊕◯◯ Low	-0.19957651	⊕⊕◯◯ Low
32	Canagliflozin:Efpeglenatide	0			-0.389949082	⊕⊕◯◯ Low	-0.389949082	⊕⊕◯◯ Low
33	Canagliflozin:Empagliflozin	0			-0.268695788	⊕⊕◯◯ Low	-0.268695788	⊕⊕◯◯ Low
34	Canagliflozin:Ertugliflozin	0			-0.346104465	⊕⊕◯◯ Low	-0.346104465	⊕⊕◯◯ Low
35	Canagliflozin:Exenatide	0			-0.267436372	⊕⊕◯◯ Low	-0.267436372	⊕⊕◯◯ Low
36	Canagliflozin:Inject_semaglutide	1	-0.218326419	⊕⊕⊕⊕ High	-0.202425464	⊕⊕⊕⊕ High	-0.204695975	⊕⊕⊕⊕ High
37	Canagliflozin:Liraglutide	0			-0.143862491	⊕⊕◯◯ Low	-0.143862491	⊕⊕◯◯ Low
38	Canagliflozin:Lixisenatide	0			-0.364737106	⊕⊕◯◯ Low	-0.364737106	⊕⊕◯◯ Low
39	Canagliflozin:Oral_semaglutide	0			0.501943009	⊕⊕◯◯ Low	0.501943009	⊕⊕◯◯ Low

40	Canagliflozin:Placebo_or_Control	4	-0.411560904	⊕⊕⊕⊕ High	-0.427461859	⊕⊕⊕⊕ High	-0.412965648	⊕⊕⊕⊕ High
41	Canagliflozin:Sotagliflozin	0			-0.259273294	⊕⊕◯◯ Low	-0.259273294	⊕⊕◯◯ Low
42	Canagliflozin:Tirzepatide	0			0.402529227	⊕⊕◯◯ Low	0.402529227	⊕⊕◯◯ Low
43	Dapagliflozin:Dulaglutide	0			-0.027250761	⊕⊕◯◯ Low	-0.027250761	⊕⊕◯◯ Low
44	Dapagliflozin:Efpeglenatide	0			-0.217623333	⊕⊕◯◯ Low	-0.217623333	⊕⊕◯◯ Low
45	Dapagliflozin:Empagliflozin	0			-0.096370039	⊕⊕◯◯ Low	-0.096370039	⊕⊕◯◯ Low
46	Dapagliflozin:Ertugliflozin	0			-0.173778716	⊕⊕◯◯ Low	-0.173778716	⊕⊕◯◯ Low
47	Dapagliflozin:Exenatide	0			-0.095110623	⊕⊕◯◯ Low	-0.095110623	⊕⊕◯◯ Low
48	Dapagliflozin:Inject_semaglutide	0			-0.032370226	⊕⊕◯◯ Low	-0.032370226	⊕⊕◯◯ Low
49	Dapagliflozin:Liraglutide	0			0.028463257	⊕⊕◯◯ Low	0.028463257	⊕⊕◯◯ Low
50	Dapagliflozin:Lixisenatide	0			-0.192411358	⊕⊕◯◯ Low	-0.192411358	⊕⊕◯◯ Low
51	Dapagliflozin:Oral_semaglutide	0			0.674268758	⊕⊕◯◯ Low	0.674268758	⊕⊕◯◯ Low
52	Dapagliflozin:Placebo_or_Control	5	-0.240639899	⊕⊕⊕⊕ High			-0.240639899	⊕⊕⊕◯ Medium
53	Dapagliflozin:Sotagliflozin	0			-0.086947546	⊕⊕◯◯ Low	-0.086947546	⊕⊕◯◯ Low
54	Dapagliflozin:Tirzepatide	0			0.574854976	⊕⊕◯◯ Low	0.574854976	⊕⊕◯◯ Low
55	Dulaglutide:Efpeglenatide	0			-0.190372572	⊕⊕◯◯ Low	-0.190372572	⊕⊕◯◯ Low
56	Dulaglutide:Empagliflozin	0			-0.069119278	⊕⊕◯◯ Low	-0.069119278	⊕⊕◯◯ Low
57	Dulaglutide:Ertugliflozin	0			-0.146527955	⊕⊕◯◯ Low	-0.146527955	⊕⊕◯◯ Low
58	Dulaglutide:Exenatide	0			-0.067859862	⊕⊕◯◯ Low	-0.067859862	⊕⊕◯◯ Low
59	Dulaglutide:Inject_semaglutide	0			-0.005119465	⊕⊕◯◯ Low	-0.005119465	⊕⊕◯◯ Low
60	Dulaglutide:Liraglutide	0			0.055714019	⊕⊕◯◯ Low	0.055714019	⊕⊕◯◯ Low

61	Dulaglutide:Lixisenatide	0			-0.165160596	⊕⊕◯◯ Low	-0.165160596	⊕⊕◯◯ Low
62	Dulaglutide:Oral_semaglutide	0			0.701519519	⊕⊕◯◯ Low	0.701519519	⊕⊕◯◯ Low
63	Dulaglutide:Placebo_or_Control	3	-0.213389138	⊕⊕⊕⊕ High			-0.213389138	⊕⊕⊕◯ Medium
64	Dulaglutide:Sotagliflozin	0			-0.059696784	⊕⊕◯◯ Low	-0.059696784	⊕⊕◯◯ Low
65	Dulaglutide:Tirzepatide	0			0.602105737	⊕⊕◯◯ Low	0.602105737	⊕⊕◯◯ Low
66	Efpeglenatide:Empagliflozin	0			0.121253294	⊕⊕◯◯ Low	0.121253294	⊕⊕◯◯ Low
67	Efpeglenatide:Ertugliflozin	0			0.043844617	⊕⊕◯◯ Low	0.043844617	⊕⊕◯◯ Low
68	Efpeglenatide:Exenatide	0			0.12251271	⊕⊕◯◯ Low	0.12251271	⊕⊕◯◯ Low
69	Efpeglenatide:Inject_semaglutide	0			0.185253107	⊕⊕◯◯ Low	0.185253107	⊕⊕◯◯ Low
70	Efpeglenatide:Liraglutide	0			0.24608659	⊕⊕◯◯ Low	0.24608659	⊕⊕◯◯ Low
71	Efpeglenatide:Lixisenatide	0			0.025211975	⊕⊕◯◯ Low	0.025211975	⊕⊕◯◯ Low
72	Efpeglenatide:Oral_semaglutide	0			0.891892091	⊕⊕◯◯ Low	0.891892091	⊕⊕◯◯ Low
73	Efpeglenatide:Placebo_or_Control	1	-0.023016566	⊕⊕⊕⊕ High			-0.023016566	⊕⊕⊕◯ Medium
74	Efpeglenatide:Sotagliflozin	0			0.130675787	⊕⊕◯◯ Low	0.130675787	⊕⊕◯◯ Low
75	Efpeglenatide:Tirzepatide	0			0.792478309	⊕⊕◯◯ Low	0.792478309	⊕⊕◯◯ Low
76	Empagliflozin:Ertugliflozin	0			-0.077408677	⊕⊕◯◯ Low	-0.077408677	⊕⊕◯◯ Low
77	Empagliflozin:Exenatide	0			0.001259416	⊕⊕◯◯ Low	0.001259416	⊕⊕◯◯ Low
78	Empagliflozin:Inject_semaglutide	0			0.063999813	⊕⊕◯◯ Low	0.063999813	⊕⊕◯◯ Low
79	Empagliflozin:Liraglutide	0			0.124833297	⊕⊕◯◯ Low	0.124833297	⊕⊕◯◯ Low
80	Empagliflozin:Lixisenatide	0			-0.096041318	⊕⊕◯◯ Low	-0.096041318	⊕⊕◯◯ Low
81	Empagliflozin:Oral_semaglutide	1	0.770638797	⊕⊕⊕⊕ High			0.770638797	⊕⊕⊕◯ Medium

82	Empagliflozin:Placebo_or_Control	7	-0.14426986	⊕⊕⊕⊕ High			-0.14426986	⊕⊕⊕◯ Medium
83	Empagliflozin:Sotagliflozin	0			0.009422494	⊕⊕◯◯ Low	0.009422494	⊕⊕◯◯ Low
84	Empagliflozin:Tirzepatide	0			0.671225015	⊕⊕◯◯ Low	0.671225015	⊕⊕◯◯ Low
85	Ertugliflozin:Exenatide	0			0.078668093	⊕⊕◯◯ Low	0.078668093	⊕⊕◯◯ Low
86	Ertugliflozin:Inject_semaglutide	0			0.14140849	⊕⊕◯◯ Low	0.14140849	⊕⊕◯◯ Low
87	Ertugliflozin:Liraglutide	0			0.202241974	⊕⊕◯◯ Low	0.202241974	⊕⊕◯◯ Low
88	Ertugliflozin:Lixisenatide	0			-0.018632642	⊕⊕◯◯ Low	-0.018632642	⊕⊕◯◯ Low
89	Ertugliflozin:Oral_semaglutide	0			0.848047474	⊕⊕◯◯ Low	0.848047474	⊕⊕◯◯ Low
90	Ertugliflozin:Placebo_or_Control	1	-0.066861183	⊕⊕⊕⊕ High			-0.066861183	⊕⊕⊕◯ Medium
91	Ertugliflozin:Sotagliflozin	0			0.08683117	⊕⊕◯◯ Low	0.08683117	⊕⊕◯◯ Low
92	Ertugliflozin:Tirzepatide	0			0.748633692	⊕⊕◯◯ Low	0.748633692	⊕⊕◯◯ Low
93	Exenatide:Inject_semaglutide	0			0.062740397	⊕⊕◯◯ Low	0.062740397	⊕⊕◯◯ Low
94	Exenatide:Liraglutide	0			0.123573881	⊕⊕◯◯ Low	0.123573881	⊕⊕◯◯ Low
95	Exenatide:Lixisenatide	0			-0.097300734	⊕⊕◯◯ Low	-0.097300734	⊕⊕◯◯ Low
96	Exenatide:Oral_semaglutide	0			0.769379381	⊕⊕◯◯ Low	0.769379381	⊕⊕◯◯ Low
97	Exenatide:Placebo_or_Control	1	-0.145529276	⊕⊕⊕⊕ High			-0.145529276	⊕⊕⊕◯ Medium
98	Exenatide:Sotagliflozin	0			0.008163078	⊕⊕◯◯ Low	0.008163078	⊕⊕◯◯ Low
99	Exenatide:Tirzepatide	0			0.669965599	⊕⊕◯◯ Low	0.669965599	⊕⊕◯◯ Low
100	Inject_semaglutide:Liraglutide	0			0.060833484	⊕⊕◯◯ Low	0.060833484	⊕⊕◯◯ Low
101	Inject_semaglutide:Lixisenatide	0			-0.160041131	⊕⊕◯◯ Low	-0.160041131	⊕⊕◯◯ Low
102	Inject_semaglutide:Oral_semaglutide	0			0.706638984	⊕⊕◯◯ Low	0.706638984	⊕⊕◯◯ Low

103	Inject_semaglutide:Placebo_or_Control	10	-0.20913544	⊕⊕⊕⊕ High	-0.193234485	⊕⊕⊕⊕ High	-0.208269673	⊕⊕⊕⊕ High
104	Inject_semaglutide:Sotagliflozin	0			-0.054577319	⊕⊕◯◯ Low	-0.054577319	⊕⊕◯◯ Low
105	Inject_semaglutide:Tirzepatide	0			0.607225202	⊕⊕◯◯ Low	0.607225202	⊕⊕◯◯ Low
106	Liraglutide:Lixisenatide	0			-0.220874615	⊕⊕◯◯ Low	-0.220874615	⊕⊕◯◯ Low
107	Liraglutide:Oral_semaglutide	0			0.6458055	⊕⊕◯◯ Low	0.6458055	⊕⊕◯◯ Low
108	Liraglutide:Placebo_or_Control	3	-0.269103157	⊕⊕⊕⊕ High			-0.269103157	⊕⊕⊕◯ Medium
109	Liraglutide:Sotagliflozin	0			-0.115410803	⊕⊕◯◯ Low	-0.115410803	⊕⊕◯◯ Low
110	Liraglutide:Tirzepatide	0			0.546391718	⊕⊕◯◯ Low	0.546391718	⊕⊕◯◯ Low
111	Lixisenatide:Oral_semaglutide	0			0.866680115	⊕⊕◯◯ Low	0.866680115	⊕⊕◯◯ Low
112	Lixisenatide:Placebo_or_Control	1	-0.048228542	⊕⊕⊕⊕ High			-0.048228542	⊕⊕⊕◯ Medium
113	Lixisenatide:Sotagliflozin	0			0.105463812	⊕⊕◯◯ Low	0.105463812	⊕⊕◯◯ Low
114	Lixisenatide:Tirzepatide	0			0.767266333	⊕⊕◯◯ Low	0.767266333	⊕⊕◯◯ Low
115	Oral_semaglutide:Placebo_or_Control	0			-0.914908657	⊕⊕◯◯ Low	-0.914908657	⊕⊕◯◯ Low
116	Oral_semaglutide:Sotagliflozin	0			-0.761216303	⊕⊕◯◯ Low	-0.761216303	⊕⊕◯◯ Low
117	Oral_semaglutide:Tirzepatide	0			-0.099413782	⊕⊕◯◯ Low	-0.099413782	⊕⊕◯◯ Low
118	Sotagliflozin:Placebo_or_Control	4	-0.153692353	⊕⊕⊕⊕ High			-0.153692353	⊕⊕⊕◯ Medium
119	Tirzepatide:Placebo_or_Control	3	-0.815494875	⊕⊕⊕⊕ High			-0.815494875	⊕⊕⊕◯ Medium
120	Sotagliflozin:Tirzepatide	0			0.661802522	⊕⊕◯◯ Low	0.661802522	⊕⊕◯◯ Low

Reference list of supplement tables:

1. Page, M.J.; McKenzie, J.E.; Bossuyt, P.M.; Boutron, I.; Hoffmann, T.C.; Mulrow, C.D.; Shamseer, L.; Tetzlaff, J.M.; Akl, E.A.; Brennan, S.E.; et al. The PRISMA 2020 statement: an updated guideline for reporting systematic reviews. *Bmj* **2021**, *372*, n71, doi:10.1136/bmj.n71.
2. Stenlof, K.; Cefalu, W.T.; Kim, K.A.; Jodar, E.; Alba, M.; Edwards, R.; Tong, C.; Canovatchel, W.; Meininger, G. Long-term efficacy and safety of canagliflozin monotherapy in patients with type 2 diabetes inadequately controlled with diet and exercise: findings from the 52-week CANTATA-M study. *Curr Med Res Opin* **2014**, *30*, 163-175, doi:10.1185/03007995.2013.850066.
3. Nozu, T.; Miyagishi, S.; Ishioh, M.; Takakusaki, K.; Okumura, T. Phlorizin attenuates postoperative gastric ileus in rats. *Neurogastroenterology and motility : the official journal of the European Gastrointestinal Motility Society* **2023**, *35*, e14659, doi:10.1111/nmo.14659.
4. Morgenthaler, L.; DePietro, R. Case Report: Small Bowel Obstruction After Starting Tirzepatide (Mounjaro). *Am Fam Physician* **2024**, *110*, 562.
5. Itoh, Y.; Tani, M.; Takahashi, R.; Yamamoto, K. Food-induced small bowel obstruction observed in a patient with inappropriate use of semaglutide. *Diabetol Int* **2024**, *15*, 850-854, doi:10.1007/s13340-024-00751-4.
6. Echeverria, P.; Saa, J.; Paz, Y.M.L.D. Emphysematous Kidney Related to the Use of Empagliflozin in a Diabetic Woman. *AACE Clin Case Rep* **2023**, *9*, 136-139, doi:10.1016/j.aace.2023.06.001.
7. Babajide, O.; K, C.N.; Solaimanzadeh, I.; Shiferaw-Deribe, Z. Case Report of Acute Pancreatitis Associated With Combination Treatment of Dulaglutide and Glipizide. *Cureus* **2022**, *14*, e20938, doi:10.7759/cureus.20938.
8. Lee, I.H.; Ahn, D.J. Dapagliflozin-associated euglycemic diabetic ketoacidosis in a patient with type 2 diabetes mellitus: A case report. *Medicine* **2020**, *99*, e20228, doi:10.1097/MD.0000000000020228.
9. Rashid, Z.; Woldesenbet, S.; Khalil, M.; Altaf, A.; Kawashima, J.; Mumtaz, K.; Pawlik, T.M. Impact of Preoperative Glucagon-Like Peptide-1

Receptor Agonist on Outcomes Following Major Surgery. *World J Surg* **2025**, doi:10.1002/wjs.12484.
10. Nielsen, J.; Friedman, S.; Norgard, B.M.; Knudsen, T.; Kjeldsen, J.; Wod, M. Glucagon-Like Peptide 1 Receptor Agonists Are Not Associated With an Increased Risk of Ileus or Intestinal Obstruction in Patients with Inflammatory Bowel Disease-A Danish Nationwide Cohort Study. *Inflamm Bowel Dis* **2024**, doi:10.1093/ibd/izae276.
11. Ding, P.; Gao, Z.; Gorenflo, M.P.; Xu, R. GLP-1 Receptor Agonists and Risk of Paralytic Ileus: A drug-target Mendelian Randomization Study. *medRxiv* **2024**, doi:10.1101/2024.10.17.24315627.
12. Du, Y.; Zhang, M.; Wang, Z.; Hu, M.; Xie, D.; Wang, X.; Guo, Z.; Zhu, J.; Zhang, W.; Luo, Z.; et al. A real-world disproportionality analysis of semaglutide: Post-marketing pharmacovigilance data. *J Diabetes Investig* **2024**, *15*, 1422-1433, doi:10.1111/jdi.14229.
13. Klonoff, D.C.; Kim, S.H.; Galindo, R.J.; Joseph, J.I.; Garrett, V.; Gombar, S.; Aaron, R.E.; Tian, T.; Kerr, D. Risks of peri- and postoperative complications with glucagon-like peptide-1 receptor agonists. *Diabetes Obes Metab* **2024**, *26*, 3128-3136, doi:10.1111/dom.15636.
14. Li, Z.; Zhang, X.; Chen, H.; Zeng, H.; Wu, J.; Wang, Y.; Ma, N.; Lan, J.; Zhang, Y.; Niu, H.; et al. Empagliflozin in children with glycogen storage disease-associated inflammatory bowel disease: a prospective, single-arm, open-label clinical trial. *Scientific reports* **2024**, *14*, 8630, doi:10.1038/s41598-024-59320-z.
15. Ueda, P.; Wintzell, V.; Melbye, M.; Eliasson, B.; Soderling, J.; Gudbjornsdottir, S.; Hveem, K.; Jonasson, C.; Svanstrom, H.; Hviid, A.; et al. Use of DPP4 Inhibitors and GLP-1 Receptor Agonists and Risk of Intestinal Obstruction: Scandinavian Cohort Study. *Clinical gastroenterology and hepatology : the official clinical practice journal of the American Gastroenterological Association* **2024**, *22*, 1226-1237 e1214, doi:10.1016/j.cgh.2023.08.034.
16. Wu, T.; Zhang, Y.; Shi, Y.; Yu, K.; Zhao, M.; Liu, S.; Zhao, Z. Safety of Glucagon-Like Peptide-1 Receptor Agonists: A Real-World Study Based on the US FDA Adverse Event Reporting System Database. *Clinical drug investigation* **2022**, *42*, 965-975, doi:10.1007/s40261-022-01202-1.
17. Faillie, J.L.; Yin, H.; Yu, O.H.Y.; Herrero, A.; Altwegg, R.; Renoux, C.; Azoulay, L. Incretin-Based Drugs and Risk of Intestinal Obstruction Among Patients With Type 2 Diabetes. *Clin Pharmacol Ther* **2022**, *111*, 272-282, doi:10.1002/cpt.2430.
18. Bennett, D.; Dave, S.; Sakaguchi, M.; Chang, C.H.; Dolin, P. Association between therapy with dipeptidyl peptidase-4 (DPP-4) inhibitors

and risk of ileus: a cohort study. *Diabetol Int* **2016**, *7*, 375-383, doi:10.1007/s13340-016-0261-3.
19. Falken, Y.; Webb, D.L.; Abraham-Nordling, M.; Kressner, U.; Hellstrom, P.M.; Naslund, E. Intravenous ghrelin accelerates postoperative gastric emptying and time to first bowel movement in humans. *Neurogastroenterology and motility : the official journal of the European Gastrointestinal Motility Society* **2013**, *25*, 474-480, doi:10.1111/nmo.12098.
20. Lavalle-Gonzalez, F.J.; Januszewicz, A.; Davidson, J.; Tong, C.; Qiu, R.; Canovatchel, W.; Meininger, G. Efficacy and safety of canagliflozin compared with placebo and sitagliptin in patients with type 2 diabetes on background metformin monotherapy: a randomised trial. *Diabetologia* **2013**, *56*, 2582-2592, doi:10.1007/s00125-013-3039-1.
21. Rosenstock, J.; Raccah, D.; Koranyi, L.; Maffei, L.; Boka, G.; Miossec, P.; Gerich, J.E. Efficacy and safety of lixisenatide once daily versus exenatide twice daily in type 2 diabetes inadequately controlled on metformin: a 24-week, randomized, open-label, active-controlled study (GetGoal-X). *Diabetes Care* **2013**, *36*, 2945-2951, doi:10.2337/dc12-2709.
22. Charbonnel, B.; Steinberg, H.; Eymard, E.; Xu, L.; Thakkar, P.; Prabhu, V.; Davies, M.J.; Engel, S.S. Efficacy and safety over 26 weeks of an oral treatment strategy including sitagliptin compared with an injectable treatment strategy with liraglutide in patients with type 2 diabetes mellitus inadequately controlled on metformin: a randomised clinical trial. *Diabetologia* **2013**, *56*, 1503-1511, doi:10.1007/s00125-013-2905-1.
23. Gallwitz, B.; Guzman, J.; Dotta, F.; Guerci, B.; Simo, R.; Basson, B.R.; Festa, A.; Kiljanski, J.; Sapin, H.; Trautmann, M.; et al. Exenatide twice daily versus glimepiride for prevention of glycaemic deterioration in patients with type 2 diabetes with metformin failure (EUREXA): an open-label, randomised controlled trial. *Lancet* **2012**, *379*, 2270-2278, doi:10.1016/S0140-6736(12)60479-6.
24. Gallwitz, B.; Bohmer, M.; Segiet, T.; Molle, A.; Milek, K.; Becker, B.; Helsberg, K.; Petto, H.; Peters, N.; Bachmann, O. Exenatide twice daily versus premixed insulin aspart 70/30 in metformin-treated patients with type 2 diabetes: a randomized 26-week study on glycemic control and hypoglycemia. *Diabetes Care* **2011**, *34*, 604-606, doi:10.2337/dc10-1900.
25. Bailey, C.J.; Gross, J.L.; Pieters, A.; Bastien, A.; List, J.F. Effect of dapagliflozin in patients with type 2 diabetes who have inadequate glycaemic control with metformin: a randomised, double-blind, placebo-controlled trial. *Lancet* **2010**, *375*, 2223-2233, doi:10.1016/S0140-6736(10)60407-2.

26. Buse, J.B.; Rosenstock, J.; Sesti, G.; Schmidt, W.E.; Montanya, E.; Brett, J.H.; Zychma, M.; Blonde, L.; Group, L.-S. Liraglutide once a day versus exenatide twice a day for type 2 diabetes: a 26-week randomised, parallel-group, multinational, open-label trial (LEAD-6). *Lancet* **2009**, *374*, 39-47, doi:10.1016/S0140-6736(09)60659-0.
27. Nauck, M.; Frid, A.; Hermansen, K.; Shah, N.S.; Tankova, T.; Mitha, I.H.; Zdravkovic, M.; During, M.; Matthews, D.R.; Group, L.-S. Efficacy and safety comparison of liraglutide, glimepiride, and placebo, all in combination with metformin, in type 2 diabetes: the LEAD (liraglutide effect and action in diabetes)-2 study. *Diabetes Care* **2009**, *32*, 84-90, doi:10.2337/dc08-1355.
28. Cefalu, W.T.; Leiter, L.A.; de Bruin, T.W.; Gause-Nilsson, I.; Sugg, J.; Parikh, S.J. Dapagliflozin's Effects on Glycemia and Cardiovascular Risk Factors in High-Risk Patients With Type 2 Diabetes: A 24-Week, Multicenter, Randomized, Double-Blind, Placebo-Controlled Study With a 28-Week Extension. *Diabetes Care* **2015**, *38*, 1218-1227, doi:10.2337/dc14-0315.
29. Weissman, P.N.; Carr, M.C.; Ye, J.; Cirkel, D.T.; Stewart, M.; Perry, C.; Pratley, R. HARMONY 4: randomised clinical trial comparing once-weekly albiglutide and insulin glargine in patients with type 2 diabetes inadequately controlled with metformin with or without sulfonylurea. *Diabetologia* **2014**, *57*, 2475-2484, doi:10.1007/s00125-014-3360-3.
30. Dungan, K.M.; Povedano, S.T.; Forst, T.; Gonzalez, J.G.; Atisso, C.; Sealls, W.; Fahrbach, J.L. Once-weekly dulaglutide versus once-daily liraglutide in metformin-treated patients with type 2 diabetes (AWARD-6): a randomised, open-label, phase 3, non-inferiority trial. *Lancet* **2014**, *384*, 1349-1357, doi:10.1016/S0140-6736(14)60976-4.
31. Rosenstock, J.; Fonseca, V.A.; Gross, J.L.; Ratner, R.E.; Ahren, B.; Chow, F.C.; Yang, F.; Miller, D.; Johnson, S.L.; Stewart, M.W.; et al. Advancing basal insulin replacement in type 2 diabetes inadequately controlled with insulin glargine plus oral agents: a comparison of adding albiglutide, a weekly GLP-1 receptor agonist, versus thrice-daily prandial insulin lispro. *Diabetes Care* **2014**, *37*, 2317-2325, doi:10.2337/dc14-0001.
32. Wysham, C.; Blevins, T.; Arakaki, R.; Colon, G.; Garcia, P.; Atisso, C.; Kuhstoss, D.; Lakshmanan, M. Efficacy and safety of dulaglutide added onto pioglitazone and metformin versus exenatide in type 2 diabetes in a randomized controlled trial (AWARD-1). *Diabetes Care* **2014**, *37*, 2159-2167, doi:10.2337/dc13-2760.
33. Umpierrez, G.; Tofe Povedano, S.; Perez Manghi, F.; Shurzinske, L.; Pechtner, V. Efficacy and safety of dulaglutide monotherapy versus

metformin in type 2 diabetes in a randomized controlled trial (AWARD-3). *Diabetes Care* **2014**, *37*, 2168-2176, doi:10.2337/dc13-2759.
34. Pratley, R.E.; Nauck, M.A.; Barnett, A.H.; Feinglos, M.N.; Ovalle, F.; Harman-Boehm, I.; Ye, J.; Scott, R.; Johnson, S.; Stewart, M.; et al. Once-weekly albiglutide versus once-daily liraglutide in patients with type 2 diabetes inadequately controlled on oral drugs (HARMONY 7): a randomised, open-label, multicentre, non-inferiority phase 3 study. *Lancet Diabetes Endocrinol* **2014**, *2*, 289-297, doi:10.1016/S2213-8587(13)70214-6.
35. Roden, M.; Weng, J.; Eilbracht, J.; Delafont, B.; Kim, G.; Woerle, H.J.; Broedl, U.C.; investigators, E.-R.M.t. Empagliflozin monotherapy with sitagliptin as an active comparator in patients with type 2 diabetes: a randomised, double-blind, placebo-controlled, phase 3 trial. *Lancet Diabetes Endocrinol* **2013**, *1*, 208-219, doi:10.1016/S2213-8587(13)70084-6.
36. Polidori, D.; Mari, A.; Ferrannini, E. Canagliflozin, a sodium glucose co-transporter 2 inhibitor, improves model-based indices of beta cell function in patients with type 2 diabetes. *Diabetologia* **2014**, *57*, 891-901, doi:10.1007/s00125-014-3196-x.
37. Ferrannini, E.; Berk, A.; Hantel, S.; Pinnetti, S.; Hach, T.; Woerle, H.J.; Broedl, U.C. Long-term safety and efficacy of empagliflozin, sitagliptin, and metformin: an active-controlled, parallel-group, randomized, 78-week open-label extension study in patients with type 2 diabetes. *Diabetes Care* **2013**, *36*, 4015-4021, doi:10.2337/dc13-0663.
38. Investigators, F.-S.T. Glucose Variability in a 26-Week Randomized Comparison of Mealtime Treatment With Rapid-Acting Insulin Versus GLP-1 Agonist in Participants With Type 2 Diabetes at High Cardiovascular Risk. *Diabetes Care* **2016**, *39*, 973-981, doi:10.2337/dc15-2782.
39. Leiter, L.A.; Cefalu, W.T.; de Bruin, T.W.; Xu, J.; Parikh, S.; Johnsson, E.; Gause-Nilsson, I. Long-term maintenance of efficacy of dapagliflozin in patients with type 2 diabetes mellitus and cardiovascular disease. *Diabetes Obes Metab* **2016**, *18*, 766-774, doi:10.1111/dom.12666.
40. Dungan, K.M.; Weitgasser, R.; Perez Manghi, F.; Pintilei, E.; Fahrbach, J.L.; Jiang, H.H.; Shell, J.; Robertson, K.E. A 24-week study to evaluate the efficacy and safety of once-weekly dulaglutide added on to glimepiride in type 2 diabetes (AWARD-8). *Diabetes Obes Metab* **2016**, *18*, 475-482, doi:10.1111/dom.12634.
41. Davies, M.J.; Bain, S.C.; Atkin, S.L.; Rossing, P.; Scott, D.; Shamkhalova, M.S.; Bosch-Traberg, H.; Syren, A.; Umpierrez, G.E. Efficacy and

Safety of Liraglutide Versus Placebo as Add-on to Glucose-Lowering Therapy in Patients With Type 2 Diabetes and Moderate Renal Impairment (LIRA-RENAL): A Randomized Clinical Trial. *Diabetes Care* **2016**, *39*, 222-230, doi:10.2337/dc14-2883.
42. Nauck, M.A.; Stewart, M.W.; Perkins, C.; Jones-Leone, A.; Yang, F.; Perry, C.; Reinhardt, R.R.; Rendell, M. Efficacy and safety of once-weekly GLP-1 receptor agonist albiglutide (HARMONY 2): 52 week primary endpoint results from a randomised, placebo-controlled trial in patients with type 2 diabetes mellitus inadequately controlled with diet and exercise. *Diabetologia* **2016**, *59*, 266-274, doi:10.1007/s00125-015-3795-1.
43. Davies, M.J.; Bergenstal, R.; Bode, B.; Kushner, R.F.; Lewin, A.; Skjoth, T.V.; Andreasen, A.H.; Jensen, C.B.; DeFronzo, R.A.; Group, N.N.S. Efficacy of Liraglutide for Weight Loss Among Patients With Type 2 Diabetes: The SCALE Diabetes Randomized Clinical Trial. *Jama* **2015**, *314*, 687-699, doi:10.1001/jama.2015.9676.
44. Mathieu, C.; Ranetti, A.E.; Li, D.; Ekholm, E.; Cook, W.; Hirshberg, B.; Chen, H.; Hansen, L.; Iqbal, N. Randomized, Double-Blind, Phase 3 Trial of Triple Therapy With Dapagliflozin Add-on to Saxagliptin Plus Metformin in Type 2 Diabetes. *Diabetes Care* **2015**, *38*, 2009-2017, doi:10.2337/dc15-0779.
45. Kovacs, C.S.; Seshiah, V.; Merker, L.; Christiansen, A.V.; Roux, F.; Salsali, A.; Kim, G.; Stella, P.; Woerle, H.J.; Broedl, U.C.; et al. Empagliflozin as Add-on Therapy to Pioglitazone With or Without Metformin in Patients With Type 2 Diabetes Mellitus. *Clin Ther* **2015**, *37*, 1773-1788 e1771, doi:10.1016/j.clinthera.2015.05.511.
46. Giorgino, F.; Benroubi, M.; Sun, J.H.; Zimmermann, A.G.; Pechtner, V. Efficacy and Safety of Once-Weekly Dulaglutide Versus Insulin Glargine in Patients With Type 2 Diabetes on Metformin and Glimepiride (AWARD-2). *Diabetes Care* **2015**, *38*, 2241-2249, doi:10.2337/dc14-1625.
47. Blonde, L.; Jendle, J.; Gross, J.; Woo, V.; Jiang, H.; Fahrbach, J.L.; Milicevic, Z. Once-weekly dulaglutide versus bedtime insulin glargine, both in combination with prandial insulin lispro, in patients with type 2 diabetes (AWARD-4): a randomised, open-label, phase 3, non-inferiority study. *Lancet* **2015**, *385*, 2057-2066, doi:10.1016/S0140-6736(15)60936-9.
48. Ludvik, B.; Frias, J.P.; Tinahones, F.J.; Wainstein, J.; Jiang, H.; Robertson, K.E.; Garcia-Perez, L.E.; Woodward, D.B.; Milicevic, Z. Dulaglutide as add-on therapy to SGLT2 inhibitors in patients with inadequately controlled type 2 diabetes (AWARD-10): a 24-week, randomised,

double-blind, placebo-controlled trial. *Lancet Diabetes Endocrinol* **2018**, *6*, 370-381, doi:10.1016/S2213-8587(18)30023-8.
49. Aronson, R.; Frias, J.; Goldman, A.; Darekar, A.; Lauring, B.; Terra, S.G. Long-term efficacy and safety of ertugliflozin monotherapy in patients with inadequately controlled T2DM despite diet and exercise: VERTIS MONO extension study. *Diabetes Obes Metab* **2018**, *20*, 1453-1460, doi:10.1111/dom.13251.
50. Pratley, R.E.; Aroda, V.R.; Lingvay, I.; Ludemann, J.; Andreassen, C.; Navarria, A.; Viljoen, A.; investigators, S. Semaglutide versus dulaglutide once weekly in patients with type 2 diabetes (SUSTAIN 7): a randomised, open-label, phase 3b trial. *Lancet Diabetes Endocrinol* **2018**, *6*, 275-286, doi:10.1016/S2213-8587(18)30024-X.
51. Ahmann, A.J.; Capehorn, M.; Charpentier, G.; Dotta, F.; Henkel, E.; Lingvay, I.; Holst, A.G.; Annett, M.P.; Aroda, V.R. Efficacy and Safety of Once-Weekly Semaglutide Versus Exenatide ER in Subjects With Type 2 Diabetes (SUSTAIN 3): A 56-Week, Open-Label, Randomized Clinical Trial. *Diabetes Care* **2018**, *41*, 258-266, doi:10.2337/dc17-0417.
52. Grunberger, G.; Camp, S.; Johnson, J.; Huyck, S.; Terra, S.G.; Mancuso, J.P.; Jiang, Z.W.; Golm, G.; Engel, S.S.; Lauring, B. Ertugliflozin in Patients with Stage 3 Chronic Kidney Disease and Type 2 Diabetes Mellitus: The VERTIS RENAL Randomized Study. *Diabetes Ther* **2018**, *9*, 49-66, doi:10.1007/s13300-017-0337-5.
53. Yu, M.; Brunt, K.V.; Milicevic, Z.; Varnado, O.; Boye, K.S. Patient-reported Outcomes in Patients with Type 2 Diabetes Treated with Dulaglutide Added to Titrated Insulin Glargine (AWARD-9). *Clin Ther* **2017**, *39*, 2284-2295, doi:10.1016/j.clinthera.2017.10.002.
54. Meneilly, G.S.; Roy-Duval, C.; Alawi, H.; Dailey, G.; Bellido, D.; Trescoli, C.; Manrique Hurtado, H.; Guo, H.; Pilorget, V.; Perfetti, R.; et al. Lixisenatide Therapy in Older Patients With Type 2 Diabetes Inadequately Controlled on Their Current Antidiabetic Treatment: The GetGoal-O Randomized Trial. *Diabetes Care* **2017**, *40*, 485-493, doi:10.2337/dc16-2143.
55. Mellander, A.; Billger, M.; Johnsson, E.; Traff, A.K.; Yoshida, S.; Johnsson, K. Hypersensitivity Events, Including Potentially Hypersensitivity-Related Skin Events, with Dapagliflozin in Patients with Type 2 Diabetes Mellitus: A Pooled Analysis. *Clinical drug investigation* **2016**, *36*, 925-933, doi:10.1007/s40261-016-0438-3.
56. Hadjadj, S.; Rosenstock, J.; Meinicke, T.; Woerle, H.J.; Broedl, U.C. Initial Combination of Empagliflozin and Metformin in Patients With Type 2 Diabetes. *Diabetes Care* **2016**, *39*, 1718-1728, doi:10.2337/dc16-0522.

57. Nauck, M.; Rizzo, M.; Johnson, A.; Bosch-Traberg, H.; Madsen, J.; Cariou, B. Once-Daily Liraglutide Versus Lixisenatide as Add-on to Metformin in Type 2 Diabetes: A 26-Week Randomized Controlled Clinical Trial. *Diabetes Care* **2016**, *39*, 1501-1509, doi:10.2337/dc15-2479.
58. Aroda, V.R.; Rosenstock, J.; Terauchi, Y.; Altuntas, Y.; Lalic, N.M.; Morales Villegas, E.C.; Jeppesen, O.K.; Christiansen, E.; Hertz, C.L.; Haluzik, M.; et al. PIONEER 1: Randomized Clinical Trial of the Efficacy and Safety of Oral Semaglutide Monotherapy in Comparison With Placebo in Patients With Type 2 Diabetes. *Diabetes Care* **2019**, *42*, 1724-1732, doi:10.2337/dc19-0749.
59. Pratley, R.; Amod, A.; Hoff, S.T.; Kadowaki, T.; Lingvay, I.; Nauck, M.; Pedersen, K.B.; Saugstrup, T.; Meier, J.J.; investigators, P. Oral semaglutide versus subcutaneous liraglutide and placebo in type 2 diabetes (PIONEER 4): a randomised, double-blind, phase 3a trial. *Lancet* **2019**, *394*, 39-50, doi:10.1016/S0140-6736(19)31271-1.
60. Husain, M.; Birkenfeld, A.L.; Donsmark, M.; Dungan, K.; Eliaschewitz, F.G.; Franco, D.R.; Jeppesen, O.K.; Lingvay, I.; Mosenzon, O.; Pedersen, S.D.; et al. Oral Semaglutide and Cardiovascular Outcomes in Patients with Type 2 Diabetes. *N Engl J Med* **2019**, *381*, 841-851, doi:10.1056/NEJMoa1901118.
61. Rosenstock, J.; Allison, D.; Birkenfeld, A.L.; Blicher, T.M.; Deenadayalan, S.; Jacobsen, J.B.; Serusclat, P.; Violante, R.; Watada, H.; Davies, M.; et al. Effect of Additional Oral Semaglutide vs Sitagliptin on Glycated Hemoglobin in Adults With Type 2 Diabetes Uncontrolled With Metformin Alone or With Sulfonylurea: The PIONEER 3 Randomized Clinical Trial. *Jama* **2019**, *321*, 1466-1480, doi:10.1001/jama.2019.2942.
62. Gallo, S.; Charbonnel, B.; Goldman, A.; Shi, H.; Huyck, S.; Darekar, A.; Lauring, B.; Terra, S.G. Long-term efficacy and safety of ertugliflozin in patients with type 2 diabetes mellitus inadequately controlled with metformin monotherapy: 104-week VERTIS MET trial. *Diabetes Obes Metab* **2019**, *21*, 1027-1036, doi:10.1111/dom.13631.
63. Coskun, T.; Sloop, K.W.; Loghin, C.; Alsina-Fernandez, J.; Urva, S.; Bokvist, K.B.; Cui, X.; Briere, D.A.; Cabrera, O.; Roell, W.C.; et al. LY3298176, a novel dual GIP and GLP-1 receptor agonist for the treatment of type 2 diabetes mellitus: From discovery to clinical proof of concept. *Mol Metab* **2018**, *18*, 3-14, doi:10.1016/j.molmet.2018.09.009.
64. Frias, J.P.; Nauck, M.A.; Van, J.; Kutner, M.E.; Cui, X.; Benson, C.; Urva, S.; Gimeno, R.E.; Milicevic, Z.; Robins, D.; et al. Efficacy and safety

of LY3298176, a novel dual GIP and GLP-1 receptor agonist, in patients with type 2 diabetes: a randomised, placebo-controlled and active comparator-controlled phase 2 trial. *Lancet* **2018**, *392*, 2180-2193, doi:10.1016/S0140-6736(18)32260-8.

65. O'Neil, P.M.; Birkenfeld, A.L.; McGowan, B.; Mosenzon, O.; Pedersen, S.D.; Wharton, S.; Carson, C.G.; Jepsen, C.H.; Kabisch, M.; Wilding, J.P.H. Efficacy and safety of semaglutide compared with liraglutide and placebo for weight loss in patients with obesity: a randomised, double-blind, placebo and active controlled, dose-ranging, phase 2 trial. *Lancet* **2018**, *392*, 637-649, doi:10.1016/S0140-6736(18)31773-2.
66. Danne, T.; Cariou, B.; Banks, P.; Brandle, M.; Brath, H.; Franek, E.; Kushner, J.A.; Lapuerta, P.; McGuire, D.K.; Peters, A.L.; et al. HbA(1c) and Hypoglycemia Reductions at 24 and 52 Weeks With Sotagliflozin in Combination With Insulin in Adults With Type 1 Diabetes: The European inTandem2 Study. *Diabetes Care* **2018**, *41*, 1981-1990, doi:10.2337/dc18-0342.
67. Tuttle, K.R.; Lakshmanan, M.C.; Rayner, B.; Busch, R.S.; Zimmermann, A.G.; Woodward, D.B.; Botros, F.T. Dulaglutide versus insulin glargine in patients with type 2 diabetes and moderate-to-severe chronic kidney disease (AWARD-7): a multicentre, open-label, randomised trial. *Lancet Diabetes Endocrinol* **2018**, *6*, 605-617, doi:10.1016/S2213-8587(18)30104-9.
68. Del Prato, S.; Kahn, S.E.; Pavo, I.; Weerakkody, G.J.; Yang, Z.; Doupis, J.; Aizenberg, D.; Wynne, A.G.; Riesmeyer, J.S.; Heine, R.J.; et al. Tirzepatide versus insulin glargine in type 2 diabetes and increased cardiovascular risk (SURPASS-4): a randomised, open-label, parallel-group, multicentre, phase 3 trial. *Lancet* **2021**, *398*, 1811-1824, doi:10.1016/S0140-6736(21)02188-7.
69. Ludvik, B.; Giorgino, F.; Jodar, E.; Frias, J.P.; Fernandez Lando, L.; Brown, K.; Bray, R.; Rodriguez, A. Once-weekly tirzepatide versus once-daily insulin degludec as add-on to metformin with or without SGLT2 inhibitors in patients with type 2 diabetes (SURPASS-3): a randomised, open-label, parallel-group, phase 3 trial. *Lancet* **2021**, *398*, 583-598, doi:10.1016/S0140-6736(21)01443-4.
70. Kosiborod, M.N.; Esterline, R.; Furtado, R.H.M.; Oscarsson, J.; Gasparyan, S.B.; Koch, G.G.; Martinez, F.; Mukhtar, O.; Verma, S.; Chopra, V.; et al. Dapagliflozin in patients with cardiometabolic risk factors hospitalised with COVID-19 (DARE-19): a randomised, double-blind, placebo-controlled, phase 3 trial. *Lancet Diabetes Endocrinol* **2021**, *9*, 586-594, doi:10.1016/S2213-8587(21)00180-7.
71. Rosenstock, J.; Wysham, C.; Frias, J.P.; Kaneko, S.; Lee, C.J.; Fernandez Lando, L.; Mao, H.; Cui, X.; Karanikas, C.A.; Thieu, V.T. Efficacy and safety of a novel dual GIP and GLP-1 receptor agonist tirzepatide in patients with type 2 diabetes (SURPASS-1): a double-blind,

randomised, phase 3 trial. *Lancet* **2021**, *398*, 143-155, doi:10.1016/S0140-6736(21)01324-6.
72. Frias, J.P.; Davies, M.J.; Rosenstock, J.; Perez Manghi, F.C.; Fernandez Lando, L.; Bergman, B.K.; Liu, B.; Cui, X.; Brown, K.; Investigators, S.-. Tirzepatide versus Semaglutide Once Weekly in Patients with Type 2 Diabetes. *N Engl J Med* **2021**, *385*, 503-515, doi:10.1056/NEJMoa2107519.
73. Wilding, J.P.H.; Batterham, R.L.; Calanna, S.; Davies, M.; Van Gaal, L.F.; Lingvay, I.; McGowan, B.M.; Rosenstock, J.; Tran, M.T.D.; Wadden, T.A.; et al. Once-Weekly Semaglutide in Adults with Overweight or Obesity. *N Engl J Med* **2021**, *384*, 989-1002, doi:10.1056/NEJMoa2032183.
74. Stack, A.G.; Han, D.; Goldwater, R.; Johansson, S.; Dronamraju, N.; Oscarsson, J.; Johnsson, E.; Parkinson, J.; Erlandsson, F. Dapagliflozin Added to Verinurad Plus Febuxostat Further Reduces Serum Uric Acid in Hyperuricemia: The QUARTZ Study. *J Clin Endocrinol Metab* **2021**, *106*, e2347-e2356, doi:10.1210/clinem/dgaa748.
75. Mullins, R.J.; Mustapic, M.; Chia, C.W.; Carlson, O.; Gulyani, S.; Tran, J.; Li, Y.; Mattson, M.P.; Resnick, S.; Egan, J.M.; et al. A Pilot Study of Exenatide Actions in Alzheimer's Disease. *Curr Alzheimer Res* **2019**, *16*, 741-752, doi:10.2174/1567205016666190913155950.
76. Pieber, T.R.; Bode, B.; Mertens, A.; Cho, Y.M.; Christiansen, E.; Hertz, C.L.; Wallenstein, S.O.R.; Buse, J.B.; investigators, P. Efficacy and safety of oral semaglutide with flexible dose adjustment versus sitagliptin in type 2 diabetes (PIONEER 7): a multicentre, open-label, randomised, phase 3a trial. *Lancet Diabetes Endocrinol* **2019**, *7*, 528-539, doi:10.1016/S2213-8587(19)30194-9.
77. Mosenzon, O.; Blicher, T.M.; Rosenlund, S.; Eriksson, J.W.; Heller, S.; Hels, O.H.; Pratley, R.; Sathyapalan, T.; Desouza, C.; Investigators, P. Efficacy and safety of oral semaglutide in patients with type 2 diabetes and moderate renal impairment (PIONEER 5): a placebo-controlled, randomised, phase 3a trial. *Lancet Diabetes Endocrinol* **2019**, *7*, 515-527, doi:10.1016/S2213-8587(19)30192-5.
78. Kadowaki, T.; Chin, R.; Ozeki, A.; Imaoka, T.; Ogawa, Y. Safety and efficacy of tirzepatide as an add-on to single oral antihyperglycaemic medication in patients with type 2 diabetes in Japan (SURPASS J-combo): a multicentre, randomised, open-label, parallel-group, phase 3 trial. *Lancet Diabetes Endocrinol* **2022**, *10*, 634-644, doi:10.1016/S2213-8587(22)00187-5.
79. Wada, T.; Mori-Anai, K.; Takahashi, A.; Matsui, T.; Inagaki, M.; Iida, M.; Maruyama, K.; Tsuda, H. Effect of canagliflozin on the decline of estimated glomerular filtration rate in chronic kidney disease patients with type 2 diabetes mellitus: A multicenter, randomized, double-

blind, placebo-controlled, parallel-group, phase III study in Japan. *J Diabetes Investig* **2022**, *13*, 1981-1989, doi:10.1111/jdi.13888.
80. Frias, J.P.; Choi, J.; Rosenstock, J.; Popescu, L.; Niemoeller, E.; Muehlen-Bartmer, I.; Baek, S. Efficacy and Safety of Once-Weekly Efpeglenatide Monotherapy Versus Placebo in Type 2 Diabetes: The AMPLITUDE-M Randomized Controlled Trial. *Diabetes Care* **2022**, *45*, 1592-1600, doi:10.2337/dc21-2656.
81. Tuttle, K.R.; Levin, A.; Nangaku, M.; Kadowaki, T.; Agarwal, R.; Hauske, S.J.; Elsasser, A.; Ritter, I.; Steubl, D.; Wanner, C.; et al. Safety of Empagliflozin in Patients With Type 2 Diabetes and Chronic Kidney Disease: Pooled Analysis of Placebo-Controlled Clinical Trials. *Diabetes Care* **2022**, *45*, 1445-1452, doi:10.2337/dc21-2034.
82. Heise, T.; Mari, A.; DeVries, J.H.; Urva, S.; Li, J.; Pratt, E.J.; Coskun, T.; Thomas, M.K.; Mather, K.J.; Haupt, A.; et al. Effects of subcutaneous tirzepatide versus placebo or semaglutide on pancreatic islet function and insulin sensitivity in adults with type 2 diabetes: a multicentre, randomised, double-blind, parallel-arm, phase 1 clinical trial. *Lancet Diabetes Endocrinol* **2022**, *10*, 418-429, doi:10.1016/S2213-8587(22)00085-7.
83. Fox, C.K.; Clark, J.M.; Rudser, K.D.; Ryder, J.R.; Gross, A.C.; Nathan, B.M.; Sunni, M.; Dengel, D.R.; Billington, C.J.; Bensignor, M.O.; et al. Exenatide for weight-loss maintenance in adolescents with severe obesity: A randomized, placebo-controlled trial. *Obesity (Silver Spring)* **2022**, *30*, 1105-1115, doi:10.1002/oby.23395.
84. Spertus, J.A.; Birmingham, M.C.; Nassif, M.; Damaraju, C.V.; Abbate, A.; Butler, J.; Lanfear, D.E.; Lingvay, I.; Kosiborod, M.N.; Januzzi, J.L. The SGLT2 inhibitor canagliflozin in heart failure: the CHIEF-HF remote, patient-centered randomized trial. *Nat Med* **2022**, *28*, 809-813, doi:10.1038/s41591-022-01703-8.
85. Dahl, D.; Onishi, Y.; Norwood, P.; Huh, R.; Bray, R.; Patel, H.; Rodriguez, A. Effect of Subcutaneous Tirzepatide vs Placebo Added to Titrated Insulin Glargine on Glycemic Control in Patients With Type 2 Diabetes: The SURPASS-5 Randomized Clinical Trial. *Jama* **2022**, *327*, 534-545, doi:10.1001/jama.2022.0078.
86. Rubino, D.M.; Greenway, F.L.; Khalid, U.; O'Neil, P.M.; Rosenstock, J.; Sorrig, R.; Wadden, T.A.; Wizert, A.; Garvey, W.T.; Investigators, S. Effect of Weekly Subcutaneous Semaglutide vs Daily Liraglutide on Body Weight in Adults With Overweight or Obesity Without Diabetes: The STEP 8 Randomized Clinical Trial. *Jama* **2022**, *327*, 138-150, doi:10.1001/jama.2021.23619.

87. Rodgers, M.; Migdal, A.L.; Rodriguez, T.G.; Chen, Z.Z.; Nath, A.K.; Gerszten, R.E.; Kasid, N.; Toschi, E.; Tripaldi, J.; Heineman, B.; et al. Weight Loss Outcomes Among Early High Responders to Exenatide Treatment: A Randomized, Placebo Controlled Study in Overweight and Obese Women. *Front Endocrinol (Lausanne)* **2021**, *12*, 742873, doi:10.3389/fendo.2021.742873.
88. Rosenstock, J.; Frias, J.P.; Rodbard, H.W.; Tofe, S.; Sears, E.; Huh, R.; Fernandez Lando, L.; Patel, H. Tirzepatide vs Insulin Lispro Added to Basal Insulin in Type 2 Diabetes: The SURPASS-6 Randomized Clinical Trial. *Jama* **2023**, *330*, 1631-1640, doi:10.1001/jama.2023.20294.
89. Garvey, W.T.; Frias, J.P.; Jastreboff, A.M.; le Roux, C.W.; Sattar, N.; Aizenberg, D.; Mao, H.; Zhang, S.; Ahmad, N.N.; Bunck, M.C.; et al. Tirzepatide once weekly for the treatment of obesity in people with type 2 diabetes (SURMOUNT-2): a double-blind, randomised, multicentre, placebo-controlled, phase 3 trial. *Lancet* **2023**, *402*, 613-626, doi:10.1016/S0140-6736(23)01200-X.
90. Frias, J.P.; Hsia, S.; Eyde, S.; Liu, R.; Ma, X.; Konig, M.; Kazda, C.; Mather, K.J.; Haupt, A.; Pratt, E.; et al. Efficacy and safety of oral orforglipron in patients with type 2 diabetes: a multicentre, randomised, dose-response, phase 2 study. *Lancet* **2023**, *402*, 472-483, doi:10.1016/S0140-6736(23)01302-8.
91. Feng, P.; Sheng, X.; Ji, Y.; Urva, S.; Wang, F.; Miller, S.; Qian, C.; An, Z.; Cui, Y. A Phase 1 Multiple Dose Study of Tirzepatide in Chinese Patients with Type 2 Diabetes. *Adv Ther* **2023**, *40*, 3434-3445, doi:10.1007/s12325-023-02536-8.
92. Gao, L.; Lee, B.W.; Chawla, M.; Kim, J.; Huo, L.; Du, L.; Huang, Y.; Ji, L. Tirzepatide versus insulin glargine as second-line or third-line therapy in type 2 diabetes in the Asia-Pacific region: the SURPASS-AP-Combo trial. *Nat Med* **2023**, *29*, 1500-1510, doi:10.1038/s41591-023-02344-1.
93. Buse, J.B.; Nordahl Christensen, H.; Harty, B.J.; Mitchell, J.; Soule, B.P.; Zacherle, E.; Cziraky, M.; Willey, V.J. Study design and baseline profile for adults with type 2 diabetes in the once-weekly subcutaneous SEmaglutide randomized PRAgmatic (SEPRA) trial. *BMJ Open Diabetes Res Care* **2023**, *11*, doi:10.1136/bmjdrc-2022-003206.
94. Aroda, V.R.; Frias, J.P.; Ji, L.; Niemoeller, E.; Nguyen-Pascal, M.L.; Denkel, K.; Espinasse, M.; Guo, H.; Baek, S.; Choi, J.; et al. Efficacy and safety of once-weekly efpeglenatide in people with suboptimally controlled type 2 diabetes: The AMPLITUDE-D, AMPLITUDE-L and AMPLITUDE-S randomized controlled trials. *Diabetes Obes Metab* **2023**, *25*, 2084-2095, doi:10.1111/dom.15079.
95. Ji, L.; Lu, Y.; Li, Q.; Fu, L.; Luo, Y.; Lei, T.; Li, L.; Ye, S.; Shi, B.; Li, X.; et al. Efficacy and safety of empagliflozin in combination with insulin in

Chinese patients with type 2 diabetes and insufficient glycaemic control: A phase III, randomized, double-blind, placebo-controlled, parallel study. *Diabetes Obes Metab* **2023**, *25*, 1839-1848, doi:10.1111/dom.15041.
96. Garvey, W.T.; Batterham, R.L.; Bhatta, M.; Buscemi, S.; Christensen, L.N.; Frias, J.P.; Jodar, E.; Kandler, K.; Rigas, G.; Wadden, T.A.; et al. Two-year effects of semaglutide in adults with overweight or obesity: the STEP 5 trial. *Nat Med* **2022**, *28*, 2083-2091, doi:10.1038/s41591-022-02026-4.
97. Inagaki, N.; Takeuchi, M.; Oura, T.; Imaoka, T.; Seino, Y. Efficacy and safety of tirzepatide monotherapy compared with dulaglutide in Japanese patients with type 2 diabetes (SURPASS J-mono): a double-blind, multicentre, randomised, phase 3 trial. *Lancet Diabetes Endocrinol* **2022**, *10*, 623-633, doi:10.1016/S2213-8587(22)00188-7.
98. Bliddal, H.; Bays, H.; Czernichow, S.; Udden Hemmingsson, J.; Hjelmesaeth, J.; Hoffmann Morville, T.; Koroleva, A.; Skov Neergaard, J.; Velez Sanchez, P.; Wharton, S.; et al. Once-Weekly Semaglutide in Persons with Obesity and Knee Osteoarthritis. *N Engl J Med* **2024**, *391*, 1573-1583, doi:10.1056/NEJMoa2403664.
99. Zhao, L.; Cheng, Z.; Lu, Y.; Liu, M.; Chen, H.; Zhang, M.; Wang, R.; Yuan, Y.; Li, X. Tirzepatide for Weight Reduction in Chinese Adults With Obesity: The SURMOUNT-CN Randomized Clinical Trial. *Jama* **2024**, *332*, 551-560, doi:10.1001/jama.2024.9217.
100. Natale, P.; Tunnicliffe, D.J.; Toyama, T.; Palmer, S.C.; Saglimbene, V.M.; Ruospo, M.; Gargano, L.; Stallone, G.; Gesualdo, L.; Strippoli, G.F. Sodium-glucose co-transporter protein 2 (SGLT2) inhibitors for people with chronic kidney disease and diabetes. *The Cochrane database of systematic reviews* **2024**, *5*, CD015588, doi:10.1002/14651858.CD015588.pub2.
101. Dei Cas, A.; Micheli, M.M.; Aldigeri, R.; Gardini, S.; Ferrari-Pellegrini, F.; Perini, M.; Messa, G.; Antonini, M.; Spigoni, V.; Cinquegrani, G.; et al. Long-acting exenatide does not prevent cognitive decline in mild cognitive impairment: a proof-of-concept clinical trial. *Journal of endocrinological investigation* **2024**, *47*, 2339-2349, doi:10.1007/s40618-024-02320-7.
102. Mu, Y.; Bao, X.; Eliaschewitz, F.G.; Hansen, M.R.; Kim, B.T.; Koroleva, A.; Ma, R.C.W.; Yang, T.; Zu, N.; Liu, M.; et al. Efficacy and safety of once weekly semaglutide 2.4 mg for weight management in a predominantly east Asian population with overweight or obesity (STEP 7): a double-blind, multicentre, randomised controlled trial. *Lancet Diabetes Endocrinol* **2024**, *12*, 184-195, doi:10.1016/S2213-8587(23)00388-1.

103. Lee, B.W.; Cho, Y.M.; Kim, S.G.; Ko, S.H.; Lim, S.; Dahaoui, A.; Jeong, J.S.; Lim, H.J.; Yu, J.M. Efficacy and Safety of Once-Weekly Semaglutide Versus Once-Daily Sitagliptin as Metformin Add-on in a Korean Population with Type 2 Diabetes. *Diabetes Ther* **2024**, *15*, 547-563, doi:10.1007/s13300-023-01515-0.
104. Tuttle, K.R.; Hauske, S.J.; Canziani, M.E.; Caramori, M.L.; Cherney, D.; Cronin, L.; Heerspink, H.J.L.; Hugo, C.; Nangaku, M.; Rotter, R.C.; et al. Efficacy and safety of aldosterone synthase inhibition with and without empagliflozin for chronic kidney disease: a randomised, controlled, phase 2 trial. *Lancet* **2024**, *403*, 379-390, doi:10.1016/S0140-6736(23)02408-X.
105. SURMOUNT-J. A Study of Tirzepatide (LY3298176) in Participants With Obesity Disease (SURMOUNT-J). Available online: https://clinicaltrials.gov/study/NCT04844918?cond=NCT04844918&rank=1 (accessed on 2024/10/28).
106. Wason, S. Efficacy and Safety of Sotagliflozin Versus Placebo in Participants With Type 2 Diabetes Mellitus Who Have Inadequate Glycemic Control While Taking Insulin Alone or With Other Oral Antidiabetic Agents (SOTA-INS). Available online: https://clinicaltrials.gov/study/NCT03285594?cond=NCT03285594&rank=1 (accessed on 2024/10/28).
107. Wason, S. Efficacy and Bone Safety of Sotagliflozin 400 and 200 mg Versus Placebo in Participants With Type 2 Diabetes Mellitus Who Have Inadequate Glycemic Control (SOTA-BONE). Available online: https://clinicaltrials.gov/study/NCT03386344?cond=NCT03386344&rank=1 (accessed on 2024/10/28).
108. Goron, A.R.; Connolly, C.; Valdez-Sinon, A.N.; Hesson, A.; Helou, C.; Kirschen, G.W. Anti-Hyperglycemic Medication Management in the Perioperative Setting: A Review and Illustrative Case of an Adverse Effect of GLP-1 Receptor Agonist. *J Clin Med* **2024**, *13*, doi:10.3390/jcm13206259.
109. Jalleh, R.J.; Plummer, M.P.; Marathe, C.S.; Umapathysivam, M.M.; Quast, D.R.; Rayner, C.K.; Jones, K.L.; Wu, T.; Horowitz, M.; Nauck, M.A. Clinical Consequences of Delayed Gastric Emptying With GLP-1 Receptor Agonists and Tirzepatide. *J Clin Endocrinol Metab* **2024**, *110*, 1-15, doi:10.1210/clinem/dgae719.
110. Stanton, E.W.; Manasyan, A.; Banerjee, R.; Hong, K.; Koesters, E.; Daar, D.A. Glucagon-Like Peptide-1 Agonists: A Practical Overview for Plastic and Reconstructive Surgeons. *Ann Plast Surg* **2025**, *94*, 121-127, doi:10.1097/SAP.0000000000004089.
111. Schernthaner, G.; Gross, J.L.; Rosenstock, J.; Guarisco, M.; Fu, M.; Yee, J.; Kawaguchi, M.; Canovatchel, W.; Meininger, G. Canagliflozin

compared with sitagliptin for patients with type 2 diabetes who do not have adequate glycemic control with metformin plus sulfonylurea: a 52-week randomized trial. *Diabetes Care* **2013**, *36*, 2508-2515, doi:10.2337/dc12-2491.
112. Aronne, L.J.; Sattar, N.; Horn, D.B.; Bays, H.E.; Wharton, S.; Lin, W.Y.; Ahmad, N.N.; Zhang, S.; Liao, R.; Bunck, M.C.; et al. Continued Treatment With Tirzepatide for Maintenance of Weight Reduction in Adults With Obesity: The SURMOUNT-4 Randomized Clinical Trial. *Jama* **2024**, *331*, 38-48, doi:10.1001/jama.2023.24945.
113. Cherney, D.Z.I.; Ferrannini, E.; Umpierrez, G.E.; Peters, A.L.; Rosenstock, J.; Powell, D.R.; Davies, M.J.; Banks, P.; Agarwal, R. Efficacy and safety of sotagliflozin in patients with type 2 diabetes and stage 3 chronic kidney disease. *Diabetes Obes Metab* **2023**, *25*, 1646-1657, doi:10.1111/dom.15019.
114. The, E.-K.C.G.; Herrington, W.G.; Staplin, N.; Wanner, C.; Green, J.B.; Hauske, S.J.; Emberson, J.R.; Preiss, D.; Judge, P.; Mayne, K.J.; et al. Empagliflozin in Patients with Chronic Kidney Disease. *N Engl J Med* **2023**, *388*, 117-127, doi:10.1056/NEJMoa2204233.
115. Lincoff, A.M.; Brown-Frandsen, K.; Colhoun, H.M.; Deanfield, J.; Emerson, S.S.; Esbjerg, S.; Hardt-Lindberg, S.; Hovingh, G.K.; Kahn, S.E.; Kushner, R.F.; et al. Semaglutide and Cardiovascular Outcomes in Obesity without Diabetes. *N Engl J Med* **2023**, *389*, 2221-2232, doi:10.1056/NEJMoa2307563.
116. Wadden, T.A.; Chao, A.M.; Machineni, S.; Kushner, R.; Ard, J.; Srivastava, G.; Halpern, B.; Zhang, S.; Chen, J.; Bunck, M.C.; et al. Tirzepatide after intensive lifestyle intervention in adults with overweight or obesity: the SURMOUNT-3 phase 3 trial. *Nat Med* **2023**, *29*, 2909-2918, doi:10.1038/s41591-023-02597-w.
117. Jastreboff, A.M.; Aronne, L.J.; Ahmad, N.N.; Wharton, S.; Connery, L.; Alves, B.; Kiyosue, A.; Zhang, S.; Liu, B.; Bunck, M.C.; et al. Tirzepatide Once Weekly for the Treatment of Obesity. *N Engl J Med* **2022**, *387*, 205-216, doi:10.1056/NEJMoa2206038.
118. Kadowaki, T.; Isendahl, J.; Khalid, U.; Lee, S.Y.; Nishida, T.; Ogawa, W.; Tobe, K.; Yamauchi, T.; Lim, S.; investigators, S. Semaglutide once a week in adults with overweight or obesity, with or without type 2 diabetes in an east Asian population (STEP 6): a randomised, double-blind, double-dummy, placebo-controlled, phase 3a trial. *Lancet Diabetes Endocrinol* **2022**, *10*, 193-206, doi:10.1016/S2213-8587(22)00008-0.
119. Kellerer, M.; Kaltoft, M.S.; Lawson, J.; Nielsen, L.L.; Strojek, K.; Tabak, O.; Jacob, S. Effect of once-weekly semaglutide versus thrice-daily

insulin aspart, both as add-on to metformin and optimized insulin glargine treatment in participants with type 2 diabetes (SUSTAIN 11): A randomized, open-label, multinational, phase 3b trial. *Diabetes Obes Metab* **2022**, *24*, 1788-1799, doi:10.1111/dom.14765.
120. Solomon, S.D.; McMurray, J.J.V.; Claggett, B.; de Boer, R.A.; DeMets, D.; Hernandez, A.F.; Inzucchi, S.E.; Kosiborod, M.N.; Lam, C.S.P.; Martinez, F.; et al. Dapagliflozin in Heart Failure with Mildly Reduced or Preserved Ejection Fraction. *N Engl J Med* **2022**, *387*, 1089-1098, doi:10.1056/NEJMoa2206286.
121. Voors, A.A.; Angermann, C.E.; Teerlink, J.R.; Collins, S.P.; Kosiborod, M.; Biegus, J.; Ferreira, J.P.; Nassif, M.E.; Psotka, M.A.; Tromp, J.; et al. The SGLT2 inhibitor empagliflozin in patients hospitalized for acute heart failure: a multinational randomized trial. *Nat Med* **2022**, *28*, 568-574, doi:10.1038/s41591-021-01659-1.
122. Anker, S.D.; Butler, J.; Filippatos, G.; Ferreira, J.P.; Bocchi, E.; Bohm, M.; Brunner-La Rocca, H.P.; Choi, D.J.; Chopra, V.; Chuquiure-Valenzuela, E.; et al. Empagliflozin in Heart Failure with a Preserved Ejection Fraction. *N Engl J Med* **2021**, *385*, 1451-1461, doi:10.1056/NEJMoa2107038.
123. Bhatt, D.L.; Szarek, M.; Pitt, B.; Cannon, C.P.; Leiter, L.A.; McGuire, D.K.; Lewis, J.B.; Riddle, M.C.; Inzucchi, S.E.; Kosiborod, M.N.; et al. Sotagliflozin in Patients with Diabetes and Chronic Kidney Disease. *N Engl J Med* **2021**, *384*, 129-139, doi:10.1056/NEJMoa2030186.
124. Bhatt, D.L.; Szarek, M.; Steg, P.G.; Cannon, C.P.; Leiter, L.A.; McGuire, D.K.; Lewis, J.B.; Riddle, M.C.; Voors, A.A.; Metra, M.; et al. Sotagliflozin in Patients with Diabetes and Recent Worsening Heart Failure. *N Engl J Med* **2021**, *384*, 117-128, doi:10.1056/NEJMoa2030183.
125. Davies, M.; Faerch, L.; Jeppesen, O.K.; Pakseresht, A.; Pedersen, S.D.; Perreault, L.; Rosenstock, J.; Shimomura, I.; Viljoen, A.; Wadden, T.A.; et al. Semaglutide 2.4 mg once a week in adults with overweight or obesity, and type 2 diabetes (STEP 2): a randomised, double-blind, double-dummy, placebo-controlled, phase 3 trial. *Lancet* **2021**, *397*, 971-984, doi:10.1016/S0140-6736(21)00213-0.
126. Gerstein, H.C.; Sattar, N.; Rosenstock, J.; Ramasundarahettige, C.; Pratley, R.; Lopes, R.D.; Lam, C.S.P.; Khurmi, N.S.; Heenan, L.; Del Prato, S.; et al. Cardiovascular and Renal Outcomes with Efpeglenatide in Type 2 Diabetes. *N Engl J Med* **2021**, *385*, 896-907, doi:10.1056/NEJMoa2108269.
127. Lock, J.P. Bexagliflozin Efficacy and Safety Trial (BEST). Available online:

https://clinicaltrials.gov/study/NCT02558296?cond=NCT02558296&rank=1 (accessed on 2024/10/28).
128. Rubino, D.; Abrahamsson, N.; Davies, M.; Hesse, D.; Greenway, F.L.; Jensen, C.; Lingvay, I.; Mosenzon, O.; Rosenstock, J.; Rubio, M.A.; et al. Effect of Continued Weekly Subcutaneous Semaglutide vs Placebo on Weight Loss Maintenance in Adults With Overweight or Obesity: The STEP 4 Randomized Clinical Trial. *Jama* **2021**, *325*, 1414-1425, doi:10.1001/jama.2021.3224.
129. Wadden, T.A.; Bailey, T.S.; Billings, L.K.; Davies, M.; Frias, J.P.; Koroleva, A.; Lingvay, I.; O'Neil, P.M.; Rubino, D.M.; Skovgaard, D.; et al. Effect of Subcutaneous Semaglutide vs Placebo as an Adjunct to Intensive Behavioral Therapy on Body Weight in Adults With Overweight or Obesity: The STEP 3 Randomized Clinical Trial. *Jama* **2021**, *325*, 1403-1413, doi:10.1001/jama.2021.1831.
130. Cannon, C.P.; Pratley, R.; Dagogo-Jack, S.; Mancuso, J.; Huyck, S.; Masiukiewicz, U.; Charbonnel, B.; Frederich, R.; Gallo, S.; Cosentino, F.; et al. Cardiovascular Outcomes with Ertugliflozin in Type 2 Diabetes. *N Engl J Med* **2020**, *383*, 1425-1435, doi:10.1056/NEJMoa2004967.
131. Heerspink, H.J.L.; Stefansson, B.V.; Correa-Rotter, R.; Chertow, G.M.; Greene, T.; Hou, F.F.; Mann, J.F.E.; McMurray, J.J.V.; Lindberg, M.; Rossing, P.; et al. Dapagliflozin in Patients with Chronic Kidney Disease. *N Engl J Med* **2020**, *383*, 1436-1446, doi:10.1056/NEJMoa2024816.
132. Packer, M.; Anker, S.D.; Butler, J.; Filippatos, G.; Pocock, S.J.; Carson, P.; Januzzi, J.; Verma, S.; Tsutsui, H.; Brueckmann, M.; et al. Cardiovascular and Renal Outcomes with Empagliflozin in Heart Failure. *N Engl J Med* **2020**, *383*, 1413-1424, doi:10.1056/NEJMoa2022190.
133. Gerstein, H.C.; Colhoun, H.M.; Dagenais, G.R.; Diaz, R.; Lakshmanan, M.; Pais, P.; Probstfield, J.; Riesmeyer, J.S.; Riddle, M.C.; Ryden, L.; et al. Dulaglutide and cardiovascular outcomes in type 2 diabetes (REWIND): a double-blind, randomised placebo-controlled trial. *Lancet* **2019**, *394*, 121-130, doi:10.1016/S0140-6736(19)31149-3.
134. Lingvay, I.; Catarig, A.M.; Frias, J.P.; Kumar, H.; Lausvig, N.L.; le Roux, C.W.; Thielke, D.; Viljoen, A.; McCrimmon, R.J. Efficacy and safety of once-weekly semaglutide versus daily canagliflozin as add-on to metformin in patients with type 2 diabetes (SUSTAIN 8): a double-blind, phase 3b, randomised controlled trial. *Lancet Diabetes Endocrinol* **2019**, *7*, 834-844, doi:10.1016/S2213-8587(19)30311-0.
135. McMurray, J.J.V.; Solomon, S.D.; Inzucchi, S.E.; Kober, L.; Kosiborod, M.N.; Martinez, F.A.; Ponikowski, P.; Sabatine, M.S.; Anand, I.S.; Belohlavek, J.; et al. Dapagliflozin in Patients with Heart Failure and Reduced Ejection Fraction. *N Engl J Med* **2019**, *381*, 1995-2008,

doi:10.1056/NEJMoa1911303.
136. Perkovic, V.; Jardine, M.J.; Neal, B.; Bompoint, S.; Heerspink, H.J.L.; Charytan, D.M.; Edwards, R.; Agarwal, R.; Bakris, G.; Bull, S.; et al. Canagliflozin and Renal Outcomes in Type 2 Diabetes and Nephropathy. *N Engl J Med* **2019**, *380*, 2295-2306, doi:10.1056/NEJMoa1811744.
137. Rodbard, H.W.; Rosenstock, J.; Canani, L.H.; Deerochanawong, C.; Gumprecht, J.; Lindberg, S.O.; Lingvay, I.; Sondergaard, A.L.; Treppendahl, M.B.; Montanya, E.; et al. Oral Semaglutide Versus Empagliflozin in Patients With Type 2 Diabetes Uncontrolled on Metformin: The PIONEER 2 Trial. *Diabetes Care* **2019**, *42*, 2272-2281, doi:10.2337/dc19-0883.
138. Wang, J.; Li, H.Q.; Xu, X.H.; Kong, X.C.; Sun, R.; Jing, T.; Ye, L.; Su, X.F.; Ma, J.H. The Effects of Once-Weekly Dulaglutide and Insulin Glargine on Glucose Fluctuation in Poorly Oral-Antidiabetic Controlled Patients with Type 2 Diabetes Mellitus. *Biomed Res Int* **2019**, *2019*, 2682657, doi:10.1155/2019/2682657.
139. Wiviott, S.D.; Raz, I.; Bonaca, M.P.; Mosenzon, O.; Kato, E.T.; Cahn, A.; Silverman, M.G.; Zelniker, T.A.; Kuder, J.F.; Murphy, S.A.; et al. Dapagliflozin and Cardiovascular Outcomes in Type 2 Diabetes. *N Engl J Med* **2019**, *380*, 347-357, doi:10.1056/NEJMoa1812389.
140. Buse, J.B.; Garg, S.K.; Rosenstock, J.; Bailey, T.S.; Banks, P.; Bode, B.W.; Danne, T.; Kushner, J.A.; Lane, W.S.; Lapuerta, P.; et al. Sotagliflozin in Combination With Optimized Insulin Therapy in Adults With Type 1 Diabetes: The North American inTandem1 Study. *Diabetes Care* **2018**, *41*, 1970-1980, doi:10.2337/dc18-0343.
141. Hernandez, A.F.; Green, J.B.; Janmohamed, S.; D'Agostino, R.B., Sr.; Granger, C.B.; Jones, N.P.; Leiter, L.A.; Rosenberg, A.E.; Sigmon, K.N.; Somerville, M.C.; et al. Albiglutide and cardiovascular outcomes in patients with type 2 diabetes and cardiovascular disease (Harmony Outcomes): a double-blind, randomised placebo-controlled trial. *Lancet* **2018**, *392*, 1519-1529, doi:10.1016/S0140-6736(18)32261-X.
142. Kaku, K.; Yamada, Y.; Watada, H.; Abiko, A.; Nishida, T.; Zacho, J.; Kiyosue, A. Safety and efficacy of once-weekly semaglutide vs additional oral antidiabetic drugs in Japanese people with inadequately controlled type 2 diabetes: A randomized trial. *Diabetes Obes Metab* **2018**, *20*, 1202-1212, doi:10.1111/dom.13218.
143. Ahren, B.; Masmiquel, L.; Kumar, H.; Sargin, M.; Karsbol, J.D.; Jacobsen, S.H.; Chow, F. Efficacy and safety of once-weekly semaglutide versus once-daily sitagliptin as an add-on to metformin, thiazolidinediones, or both, in patients with type 2 diabetes (SUSTAIN 2): a 56-

week, double-blind, phase 3a, randomised trial. *Lancet Diabetes Endocrinol* **2017**, *5*, 341-354, doi:10.1016/S2213-8587(17)30092-X.
144. Aroda, V.R.; Bain, S.C.; Cariou, B.; Piletic, M.; Rose, L.; Axelsen, M.; Rowe, E.; DeVries, J.H. Efficacy and safety of once-weekly semaglutide versus once-daily insulin glargine as add-on to metformin (with or without sulfonylureas) in insulin-naive patients with type 2 diabetes (SUSTAIN 4): a randomised, open-label, parallel-group, multicentre, multinational, phase 3a trial. *Lancet Diabetes Endocrinol* **2017**, *5*, 355-366, doi:10.1016/S2213-8587(17)30085-2.
145. Holman, R.R.; Bethel, M.A.; Mentz, R.J.; Thompson, V.P.; Lokhnygina, Y.; Buse, J.B.; Chan, J.C.; Choi, J.; Gustavson, S.M.; Iqbal, N.; et al. Effects of Once-Weekly Exenatide on Cardiovascular Outcomes in Type 2 Diabetes. *N Engl J Med* **2017**, *377*, 1228-1239, doi:10.1056/NEJMoa1612917.
146. Home, P.D.; Ahren, B.; Reusch, J.E.B.; Rendell, M.; Weissman, P.N.; Cirkel, D.T.; Miller, D.; Ambery, P.; Carr, M.C.; Nauck, M.A. Three-year data from 5 HARMONY phase 3 clinical trials of albiglutide in type 2 diabetes mellitus: Long-term efficacy with or without rescue therapy. *Diabetes Res Clin Pract* **2017**, *131*, 49-60, doi:10.1016/j.diabres.2017.06.013.
147. Januzzi, J.L., Jr.; Butler, J.; Jarolim, P.; Sattar, N.; Vijapurkar, U.; Desai, M.; Davies, M.J. Effects of Canagliflozin on Cardiovascular Biomarkers in Older Adults With Type 2 Diabetes. *J Am Coll Cardiol* **2017**, *70*, 704-712, doi:10.1016/j.jacc.2017.06.016.
148. Neal, B.; Perkovic, V.; Mahaffey, K.W.; de Zeeuw, D.; Fulcher, G.; Erondu, N.; Shaw, W.; Law, G.; Desai, M.; Matthews, D.R.; et al. Canagliflozin and Cardiovascular and Renal Events in Type 2 Diabetes. *N Engl J Med* **2017**, *377*, 644-657, doi:10.1056/NEJMoa1611925.
149. Marso, S.P.; Daniels, G.H.; Brown-Frandsen, K.; Kristensen, P.; Mann, J.F.; Nauck, M.A.; Nissen, S.E.; Pocock, S.; Poulter, N.R.; Ravn, L.S.; et al. Liraglutide and Cardiovascular Outcomes in Type 2 Diabetes. *N Engl J Med* **2016**, *375*, 311-322, doi:10.1056/NEJMoa1603827.
150. Marso, S.P.; Bain, S.C.; Consoli, A.; Eliaschewitz, F.G.; Jodar, E.; Leiter, L.A.; Lingvay, I.; Rosenstock, J.; Seufert, J.; Warren, M.L.; et al. Semaglutide and Cardiovascular Outcomes in Patients with Type 2 Diabetes. *N Engl J Med* **2016**, *375*, 1834-1844, doi:10.1056/NEJMoa1607141.
151. Pfeffer, M.A.; Claggett, B.; Diaz, R.; Dickstein, K.; Gerstein, H.C.; Kober, L.V.; Lawson, F.C.; Ping, L.; Wei, X.; Lewis, E.F.; et al. Lixisenatide in Patients with Type 2 Diabetes and Acute Coronary Syndrome. *N Engl J Med* **2015**, *373*, 2247-2257, doi:10.1056/NEJMoa1509225.
152. Pi-Sunyer, X.; Astrup, A.; Fujioka, K.; Greenway, F.; Halpern, A.; Krempf, M.; Lau, D.C.; le Roux, C.W.; Violante Ortiz, R.; Jensen, C.B.; et al.

A Randomized, Controlled Trial of 3.0 mg of Liraglutide in Weight Management. *N Engl J Med* **2015**, *373*, 11-22, doi:10.1056/NEJMoa1411892.
153. Weinstock, R.S.; Guerci, B.; Umpierrez, G.; Nauck, M.A.; Skrivanek, Z.; Milicevic, Z. Safety and efficacy of once-weekly dulaglutide versus sitagliptin after 2 years in metformin-treated patients with type 2 diabetes (AWARD-5): a randomized, phase III study. *Diabetes Obes Metab* **2015**, *17*, 849-858, doi:10.1111/dom.12479.
154. Zinman, B.; Wanner, C.; Lachin, J.M.; Fitchett, D.; Bluhmki, E.; Hantel, S.; Mattheus, M.; Devins, T.; Johansen, O.E.; Woerle, H.J.; et al. Empagliflozin, Cardiovascular Outcomes, and Mortality in Type 2 Diabetes. *N Engl J Med* **2015**, *373*, 2117-2128, doi:10.1056/NEJMoa1504720.
155. Barnett, A.H.; Mithal, A.; Manassie, J.; Jones, R.; Rattunde, H.; Woerle, H.J.; Broedl, U.C.; investigators, E.-R.R.t. Efficacy and safety of empagliflozin added to existing antidiabetes treatment in patients with type 2 diabetes and chronic kidney disease: a randomised, double-blind, placebo-controlled trial. *Lancet Diabetes Endocrinol* **2014**, *2*, 369-384, doi:10.1016/S2213-8587(13)70208-0.
156. Ridderstrale, M.; Andersen, K.R.; Zeller, C.; Kim, G.; Woerle, H.J.; Broedl, U.C.; investigators, E.-R.H.H.S.t. Comparison of empagliflozin and glimepiride as add-on to metformin in patients with type 2 diabetes: a 104-week randomised, active-controlled, double-blind, phase 3 trial. *Lancet Diabetes Endocrinol* **2014**, *2*, 691-700, doi:10.1016/S2213-8587(14)70120-2.
157. Wilding, J.P.; Woo, V.; Soler, N.G.; Pahor, A.; Sugg, J.; Rohwedder, K.; Parikh, S.; Dapagliflozin 006 Study, G. Long-term efficacy of dapagliflozin in patients with type 2 diabetes mellitus receiving high doses of insulin: a randomized trial. *Ann Intern Med* **2012**, *156*, 405-415, doi:10.7326/0003-4819-156-6-201203200-00003.
158. Garber, A.; Henry, R.; Ratner, R.; Garcia-Hernandez, P.A.; Rodriguez-Pattzi, H.; Olvera-Alvarez, I.; Hale, P.M.; Zdravkovic, M.; Bode, B.; Group, L.-S. Liraglutide versus glimepiride monotherapy for type 2 diabetes (LEAD-3 Mono): a randomised, 52-week, phase III, double-blind, parallel-treatment trial. *Lancet* **2009**, *373*, 473-481, doi:10.1016/S0140-6736(08)61246-5.
